# Almost All Antipsychotics Result in Weight Gain: A Meta-Analysis

**DOI:** 10.1371/journal.pone.0094112

**Published:** 2014-04-24

**Authors:** Maarten Bak, Annemarie Fransen, Jouke Janssen, Jim van Os, Marjan Drukker

**Affiliations:** 1 Maastricht University Medical Centre, South Limburg Mental Health Research and Teaching Network, EURON, Maastricht, The Netherlands; 2 Maxima Medical Centre Dep. of gynaecology, Veldhoven, The Netherlands; 3 King's College London, King's Health Partners, Department of Psychosis Studies, Institute of Psychiatry, London, United Kingdom; Baylor College of Medicine, United States of America

## Abstract

**Introduction:**

Antipsychotics (AP) induce weight gain. However, reviews and meta-analyses generally are restricted to second generation antipsychotics (SGA) and do not stratify for duration of AP use. It is hypothesised that patients gain more weight if duration of AP use is longer.

**Method:**

A meta-analysis was conducted of clinical trials of AP that reported weight change. Outcome measures were body weight change, change in BMI and clinically relevant weight change (7% weight gain or loss). Duration of AP-use was stratified as follows: ≤6 weeks, 6–16 weeks, 16–38 weeks and >38 weeks. Forest plots stratified by AP as well as by duration of use were generated and results were summarised in figures.

**Results:**

307 articles met inclusion criteria. The majority were AP switch studies. Almost all AP showed a degree of weight gain after prolonged use, except for amisulpride, aripiprazole and ziprasidone, for which prolonged exposure resulted in negligible weight change. The level of weight gain per AP varied from discrete to severe. Contrary to expectations, switch of AP did not result in weight loss for amisulpride, aripiprazole or ziprasidone. In AP-naive patients, weight gain was much more pronounced for all AP.

**Conclusion:**

Given prolonged exposure, virtually all AP are associated with weight gain. The rational of switching AP to achieve weight reduction may be overrated. In AP-naive patients, weight gain is more pronounced.

## Introduction

Weight gain resulting in overweight and more particularly obesity is a growing problem worldwide. Overweight and particularly obesity predicts cardiovascular risk, metabolic syndrome (MS) and diabetes mellitus type 2 (DM-II) [1]–[5] as well as an increased risk for cancer [6],[7].

In general, the life expectancy of patients with Severe Mental Illness (SMI) is reduced compared with the general population [8]. In SMI patients, overweight and obesity are more prevalent compared to the general population [9], whilst risk of developing cardiovascular diseases is substantially increased [9], [10]. People with a diagnosis of schizophrenia have a 2–3 times increased standard mortality ratio, for all causes of death [11]–[13]. Compared to the general population, the risk of developing cardiovascular illness is doubled and more than five times higher for endocrine disease [12], [14]. For people with bipolar disorder, the standard mortality rate (SMR) due to cardiovascular disease is 2, and for unipolar depression the SMR is 1.5 in men and 1.6 in women [15]. Long term use of antipsychotics (AP) is associated with increased mortality risk in people with SMI [16], [17]. In general, it is concluded that AP add to the increased mortality risk of people with SMI either through direct cardio toxic effects or by impacting on weight gain [8], [18].

In 1999, Allison [19] showed that most AP are associated with an increase in body weight and this was the starting point for the present meta-analysis, given that after this date, systematic attention to weight gain in trials became the norm. In addition, Allison [19] suggested to study clinically significant weight gain and weight loss and provided a definition. Subsequent meta-analyses confirmed the finding that most AP contributes to weight gain [20]–[26]. Particularly clozapine and olanzapine were associated with severe weight gain, whereas aripiprazole and ziprasidone appeared almost weight neutral [5], [20], [23], [24], [26]–[31]. The meta-analysis by Tarricone and colleagues is of special interest as this study showed that in AP naive patients, BMI increases with duration of AP use [26].

Duration of AP use was studied in only two meta-analyses [24], [26]. The study by Parsons and colleagues contrasted a short duration (4–12 weeks) with a long duration (around 52 weeks). The study by Tarricone and colleagues included 11 studies in AP-naive patients who were prescribed an AP, defining three periods of AP exposure (4–8 wks, 10–12 wks and 24–48 wks). Both studies showed that long term use of AP was associated with more weight gain compared with short term use. These studies did not differentiate individual AP.

Several factors explain weight gain due to AP and the impact of duration of AP use on bodyweight. AP medication induces changes in appetite and food intake, most likely because of interaction with serotonergic [32], histaminergic [33] and dopaminergic [34] neurotransmitter systems inducing increase in appetite and food intake. Therefore, the effects on weight and Body Mass Index (BMI) likely will progress with time. Duration of AP use thus is thought to constitute an important factor contributing to weight gain [24], [26]. In addition, certain diagnoses like schizophrenia and to lesser extent bipolar disorder have been associated with a higher level of metabolic dysregulation [32] and weight gain may be more substantial in this group of patients.

The study by Allison [19] recalculated the data towards a 10 weeks period and in the study by Leucht and colleagues [35] only studies shorter than 12 weeks were included. These studies ignore the importance of duration of AP use. Changes in body weight are usually more prominent after prolonged exposure to an AP. So, there is an urgent need to summarise studies stratified by duration of exposure.

Studies in drug-naive patients are more informative than switch studies, as weight outcomes are not influenced by the level of overweight due to a previous AP, thus allowing for assessment of an effect that can be attributed to a specific AP. Two previous meta-analyses have published data in AP-naive patients. In first-episode schizophrenia patients, weight gain was more prominent compared to chronic patients [24]. Second, BMI increases after first exposure to AP from more than 1 BMI point after 4–8 weeks, to almost 4 BMI points after 24–48 weeks [26]. However, because studies in drug-naive patients starting an AP are rare, the present meta-analysis examines both the total group and the subgroup of studies in restricted to drug-naive patients. When drawing conclusions, it should be considered that results pertaining to drug-naive patients are more likely to reflect the true extent impact of weight change induced by AP than results from a meta-analysis combining switch studies and studies in AP-naive patients.

The various systematic reviews and meta-analyses described above addressed the association between a selection of antipsychotics and weight gain. However, none of the previous reviews intended to include all randomised controlled trials, irrespective of diagnosis or dosage of all antipsychotics including data on weight change across all durations of treatment. Meta-analyses almost exclusively focused on schizophrenia and related psychoses or bipolar disorder, whereas AP are used in many patients with other diagnostic categories such as anxiety disorders, depression, dementia, personality disorders or Tourette's Syndrome. Therefore, a more comprehensive approach is required. A complete overview of all AP will enhance the understanding of the clinical impact of weight change for each AP separately. Additionally, AP are generally used long-time and, therefore, duration of AP exposure is a factor of interest associated with potential weight gain over time. Only two previous meta-analyses included this factor [24], [26]. Finally, as already mentioned above, meta-analyses on AP naive patients are very rare. Therefore, the present meta-analysis aims to assess crude weight changes after the start of an antipsychotic or after the switch to another antipsychotic, including all antipsychotics ever examined in a randomised controlled trial (RCT).

The study by Allison [19] launched the interest in weight change and metabolic problems as important side effects of antipsychotics. After this study, interest in metabolic changes due to AP gradually increased, leading to presentation of data on metabolic changes, including changes of body weight, in medication studies. The present meta-analysis additionally included proportion of clinically relevant weight gain and weight loss as well as durations of follow-up exceeding one year. The search in the present paper was limited to articles published after January 1999. Before 1999, there was no systematic consensus to assess body weight, BMI or 7% weight gain or loss.

Whether all AP result in weight gain remains unclear, as the majority of the studies are restricted to the most prescribed SGAs or haloperidol as comparator [19]–[21], [24]–[26], [34]. Previous meta-analyses and reviews did not focus on FGA with the exception of haloperidol, or treated FGA as a single homogenous group. Generally, it is suggested that FGA are weight neutral, but at the time these drugs were launched studies did not include weight change as an outcome. Another problem is that outcome of studies and meta-analyses are contaminated by two factors: (i) mix of study duration (short and long term studies) whereas effects on weight are delayed and (ii) no distinction is made between first episode of drug naive patients and chronic patients [20].

The recent meta-analysis by Leucht et al [35] included 15 AP of which only haloperidol was a FGA compound. A refined statistical method (network analysis) allowed for mutual comparison between AP and shows that haloperidol has the least impact weight gain. Leucht and colleagues only included weight change as an outcome, not BMI change or 7% weight gain of 7% weight loss. The result showed that olanzapine was associated with the most gain in weight. However the authors did not control for duration of AP use effects. In addition, the meta-analyses did not include variables BMI change nor the proportion of subjects with 7% weight increase or 7% weight loss [35]. Finally, as mentioned above previous meta-analyses studied predominantly schizophrenia and related psychoses and bipolar disorders. This emphasizes the need for comprehensive analyses including all data on weight change per AP available.

### Hypothesis/study goals

The present study assessed absolute changes in body weight and BMI as well as the proportion of subjects with more than seven per cent increase or decrease in body weight after the start of a specific AP. Second, body weight change, change in BMI and change in >7% weight increase (or clinically relevant weight gain) or >7% weight loss (or clinically relevant weight loss) in AP-naive patients were examined as a function of duration of AP exposure, allowing for assessment of possible progress with duration of AP exposure.

## Method

### Data sources

The meta-analysis was conducted and reported according to recommendations of the Meta-analysis of Observational Studies in Epidemiology (MOOSE) group [36]. A review protocol was construed following the MOOSE guidelines. This was not published but only for internal use of this study.

A PubMed and Embase search was conducted for articles on metabolic side effect profiles of antipsychotic medication. The search term used was: ((“weight gain” OR “BMI” OR “7% weight”) AND (chlorpromazine OR haloperidol OR bromperidol OR fluphenazine OR zuclopenthixol OR pentixol OR flupentixol OR levopromazine OR perphenazine OR pimozide OR penfluridol OR sulpiride OR amisulpride OR amoxapine OR asenapine OR aripiprazole OR blonanserine OR clozapine OR iloperidone OR melperone OR olanzapine OR risperidone OR paliperidone OR quetiapine OR sertindole OR lurasidone OR ziprasidone)) NOT (addition OR additive OR adjunctive OR augmentation OR lithium OR valproate OR carbamazepine OR metformin OR topiramate OR ramelteon OR rimonabant OR modafinil OR sibutramine OR genetics OR pharmacokinetics OR vomiting OR nausea OR review OR “cognitive behavioural therapy” OR “cognitive behavioral therapy” OR delirium OR steroids OR ropinirole OR sleep OR “brain volume”)

*Limits Activated:* Humans, Clinical Trial, Randomized Controlled Trial, Clinical Trial, Phase IV, Controlled Clinical Trial, English, German, All Adult: 18+ years, Publication Date from 1999/01/01 to 2011/12/31.

### Inclusion criteria and study evaluation

The aim of the search was to identify randomised controlled studies (RCT) or controlled clinical trials where subjects were randomised into various AP intervention groups. The identified outcome was absolute change in weight, BMI (continuous) or 7% weight loss or 7% weight increase. Studies were included if they compared two or more AP or AP versus placebo. There were no restrictions with regard to diagnosis, age, dosage of AP or duration of AP exposure.

The inclusion criteria were:

1. Weight gain (continuous), BMI (continuous) or 7% weight loss or 7% weight increase.
2. Age 18 years or older
3. Minimum follow-up of one week
4. Data available for AP treatment and weight change
5. Randomised controlled trial, controlled clinical trial or clinical trial or phase IV clinical trial with adequate control group with intention to treat.
6. 01-01-1999/12-31-2011

Excluded were studies designed to influence weight gain in patients with eating disorders such as anorexia or bulimia nervosa and studies involving somatic causes of weight change irrespective of the medication (e.g. delirium). Very short term or acute antipsychotic interventions, rapid tranquilisation, or brain imaging studies used for assessing AP impact on brain morphology or brain function were excluded. In these studies, antipsychotic interventions were very brief (ranging from a single dose to a 7-day regimen). These studies are excluded as they are not expected to show a clear change on body weight. Additionally, evaluation of weight change in short term interventions is often evaluated in case of treatment of transient confusion or delirium which is complicated by underlying somatic illness that may explain body weight change directly and therefore represents a biased assessment of AP-impact on weight change. Also excluded were studies on specific (non-) pharmacologic interventions to reduce weight such as medication augmentation strategies, dietary programs, psycho-education or cognitive behavioural therapy (CBT). Systematic reviews, meta-analyses, case reports and poster presentations are also excluded.

Quality assessment was based on items given in the MOOSE checklist, which summarises recommendations of an expert panel for reporting meta-analyses and systematic reviews of observational studies [36]. Methodological issues evaluated with the checklist were the presence of a clearly focused study question, an appropriate study type, an adequate recruitment of patients and controls, an unbiased measurement of outcomes, the identification of and statistical control of important confounding factors, the completeness of follow-up and the precision of estimates.

All papers were reviewed by two independent researchers (MB and AF or JJ), who studied the papers closely on methodology and outcome measures based on the MOOSE checklist criteria. In case of doubt, papers were discussed and consensus was reached.

The search strategy initially was limited to PubMed. After this search was completed, including the screening of papers and data entry, the same strategy was applied to EMBASE. First authors were contacted in case of missing information. Pharmaceutical companies were contacted for unpublished data or papers not cited in Pubmed or Embase. In case of papers that were not present in the University Library, authors were contacted to provide the requested article.

### Search strategy

**Figure 1:**
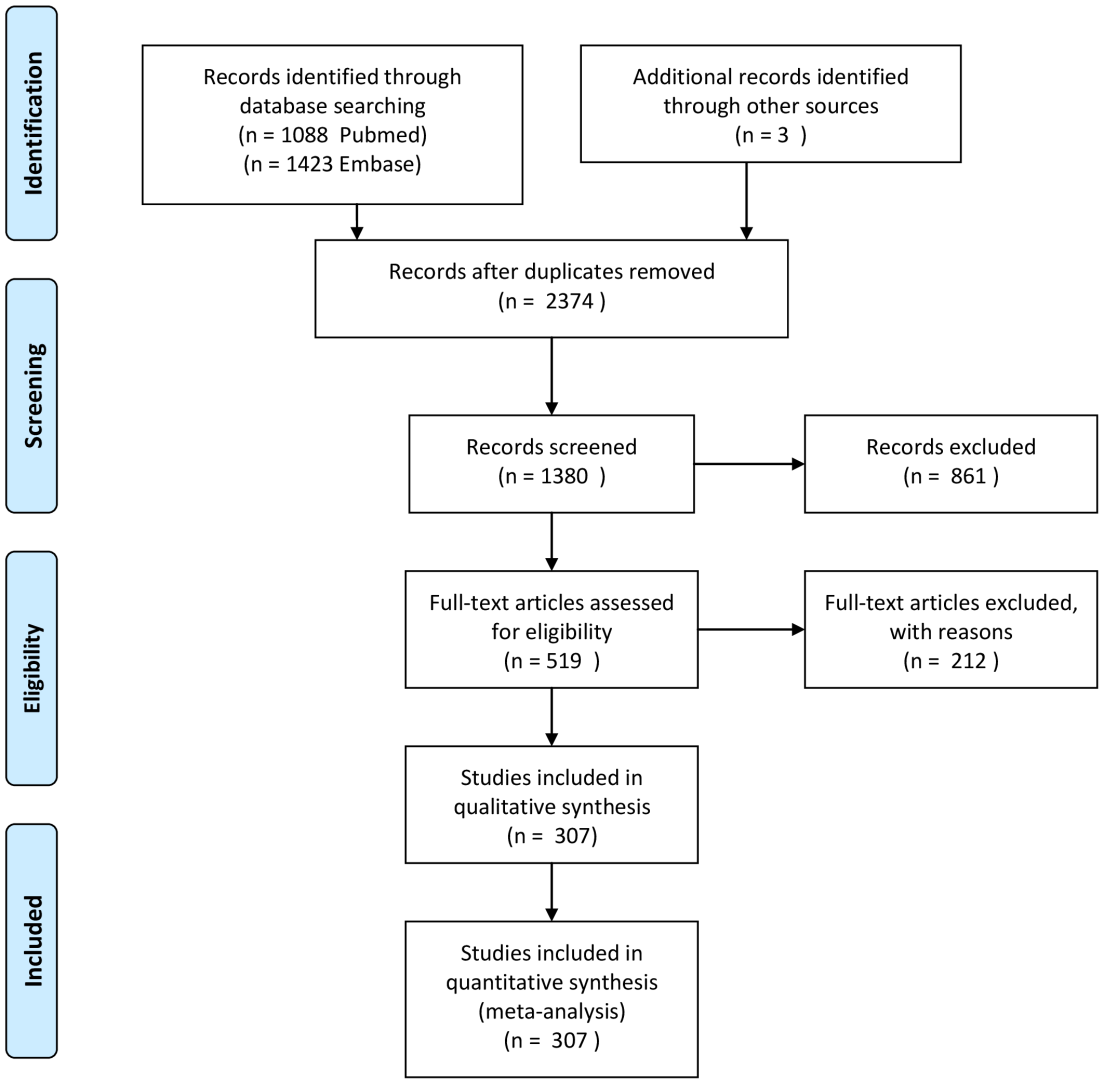
Prisma Checklist flow diagram.

The PubMed search yielded 1088 citations. The Embase search yielded 1423 citations. After removing duplicates between Pubmed en Embase 2374 papers remained. Screening papers resulted in exclusion of papers if the study did not meet the inclusion criteria despite the limits activated, e.g. rapid tranquillisation studies, reviews or meta-analyses, case reports, weight intervention studies, studies with duration of one week or brain morphology studies examining the effect of a single dose of medication, and left 1380 articles. Of the studies eligible for more detailed evaluation. Full text article screening resulted in rejection of papers because of incomplete data, absence of crude data, study or data redundancy or failure to provide data per antipsychotic (an exception was made for articles presenting data as FGA or SGA, rather than the specific AP) overviews, risk assessment studies, case reports or cross-sectional studies and resulted in 519 papers. After qualitative assessment 307 papers were selected and used for data extraction (See Figure 1 Prisma Checklist flow diagram). One paper was treated as two separate studies, as it presented two separate data sets in a single paper [37].

### Data extraction

Data from RCT's were extracted if based on intention-to-treat analysis. Before data entry, lbs units were converted to kg.

### Duration of exposure categories

In order to calculate the association between duration of antipsychotic use and gain in body weight, four exposure categories were defined: short term (≤6 weeks), medium short term (6–16 weeks), medium term (16–38 weeks) and long term (>38 weeks).

### Outcomes

Four outcome measures were defined: (i) body weight gain in kilogram's (kg), (ii) BMI, and (iii) 7% proportion of weight gain or (iv) weight loss after starting an AP. The 7% weight gain or weight loss represents the cut-off for clinically relevant weight change. The association between an AP and any of the four outcomes (weight change, change in BMI, proportion >7% weight gain, and >7% weight loss) was only presented if data of more than one study was available. Rates were transformed [ln(proportion/(1-proportion))], in order to avoid negative numbers in the confidence intervals (CI) (0 is lowest valid value in rates).

In case weight change or BMI change were not presented in the original paper, weight change or BMI change were calculated by subtracting end of study body weight or BMI post-baseline study body weight or BMI (body weight baseline - end body weight or baseline BMI - end BMI). As in this instance standard errors were not available, these were estimated using the formulas below:
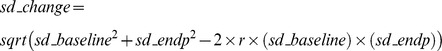


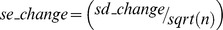
in which:

r = correlation between weight at baseline and weight at follow up

sd_change = estimated standard deviation of weight change scores

sd_baseline = standard deviation of baseline weight

sd_endp = standard deviation of endpoint weights

se_change = estimated standard error of weight change

n = number of subjects per study.

r was estimated using data from a local longitudinal register of medication use in relation to somatic parameters [38] (data available July 2006–September 2012) as follows: weight change →6–16 weeks: 0.96 (n = 220); 16–38 weeks: 0.95 (n = 241); 38–260 weeks 0.93 (n = 961); BMI change →6–16 weeks: 0.96 (n = 212); 16–38 weeks (n = 240): 0.96; 38–260 weeks: 0.92 (n = 936). The r for duration of ≤6 weeks was also conservatively set at 0.96, as the longitudinal register had relatively few observations for this duration (n = 11) and in theory r increases when duration decreases.

### Statistical analysis

All analyses were performed using Stata 12 [39]. In order to examine the four outcomes per antipsychotic for each duration of exposure category, the Stata command *metan* [40] generated forest plots including pooled estimates (absolute changes) with their corresponding 95% confidence interval (95% CI). This was repeated including only studies with drug-naive patients. This same procedure was performed for the rates, but because of the transformation of the rates before analyses, the R-program was used to make forest plots of the back-transformed results [41].

The computation of summary effects was carried out under the random-effects model, in which Tau was estimated using the DerSimonian-Laird method. Heterogeneity analyses were carried out using the chi-square, I-square, and Tau-square statistics. Tau-square estimates the total amount of variability (heterogeneity) among the effect sizes, but does not differentiate between sources. Heterogeneity may be due to random or systematic differences between the estimated effect sizes. I-square estimates the proportion of the total variability in the effect size estimates that is due to heterogeneity among the true effects.

The present analyses aim to test whether changes in weight, BMI or the proportion of 7% weight gain and weight loss are statistically significant. The present paper also presents figures per AP for each outcome measure. These figures are for descriptive purposes only. Using the present methods of analysis, comparisons between interventions (including placebo) or between exposure durations ignores clustering in the data (given more than one intervention group extracted per article and given the fact that intervention groups are clustered because of the randomisation).

In addition, in a subset of antipsychotic compounds with sufficient data available, a meta-regression analysis was performed to test whether duration of AP use was a modifier.

## Results

### Weight change per type of antipsychotic for each duration of exposure category

**Figure 2:**
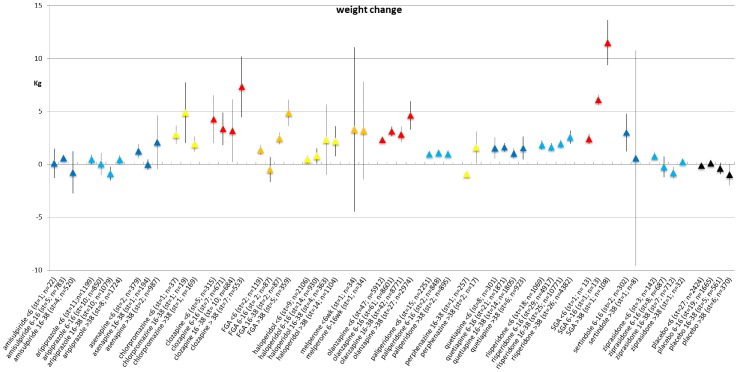
Weight change (in kg) per period per antipsychotic medication.

Of the 307 included studies, a total of 257 studies reported results on weight change (603 records in the meta-analysis data). In table S1 the number of reporting papers per AP are given for the outcomes weight change, BMI change, percentage >7% weight gain, percentage >7% weight loss. Figure 2 shows the mean change per antipsychotic per duration of exposure category. More details are presented in see table S2 and forest plots S1–S16 in File S1 and File S2. For some AP, only 1 study was available, or data could not be used (sd or se could not be calculated) – these were therefore not included in the meta-analyses. The excluded AP and their weight change are amoxapine 1.05 kg (<6 wk, n = 22) [42], blonanserine 1.29 kg, sd = 3.48 (6–16 wk, n = 92) [43], fluphenazine −2.6 (16–38 wk, n = 9) [44], iloperidone 2.6, sd = 3.7 (<6 wk, n = 1239) [45], levomepromazine 4.1 (16–38 wk, n = 19) [46], lurasidone 0.9 kg (<6 wk, n = 90) [47], pimozide 2.9 (6–16 wk, n = 24) [48] and zuclopentixol 0.6 (6–16 wk, n = 19) [49]. The I-square of the included studies varied strongly, ranging from 10.3%–99.8% (in case 4 or more studies were included in the analysis), indicating little heterogeneity to very strong heterogeneity.

Most AP showed a statistically significant change in weight post-baseline, with the exception of amisulpride, aripiprazole, asenapine, sertindole, ziprasidone and placebo which showed no statistically significant weight change. However, these results are crude outcomes regarding weight change and therefore merely suggestive for differences in the magnitude of weight change per AP. Although comparison between APs is not tested, the crude data suggest that clozapine and olanzapine show the most severe weight gain post-baseline, while FGA, for example haloperidol, are also associated with significant weight gain. Even over the shortest exposure period of ≤6 weeks, an increase in body weight post-baseline was evident for most AP (Table 1).

**Table 1:** Metaregression of weight changes per period.

Period	aripiprazole	asenapine	clozapine	FGA	haloperidol	olanzapine	quetiapine	risperidone	ziprasidone	placebo
≤6 wk*	0	0	0	0	0	0	0	0	0	0
6–16 wk	−0.46 *−1.78–0.85*		−2.37 *−6.93–2.19*	−2.19 *−6.63–2.25*	−0.25 *−2.50–1.99*	0.472 *−0.16–1.60*	0.05 *−1.26–1.36*	−0.58 *−1.57–0.72*	−0.97 *−3.08–1.13*	0.25 *−0.14–0.64*
16–38 wk	**−1.43 ** ***−2.75–−0.12***	−1.25 *−5.98–3.48*	−3.81 *−8.18–0.55*		2.75 *−0.58–6.08*	0.26*−0.68–1.20*	−0.54*−1.94–0.86*	−0,03 −1.03–0.96	−1.68 *−3.78–0.41*	−0.26 *−0.81–0.28*
>38 wk	−0.20 *−1.64–1.24*	0.74 *−3.24–4.72*	1.09 *−3.47–5.66*	2.79 *−1.12–6.70*	1.81 *−0.53–4.15*	**1.74 ** ***0.50–2.99***	−0.85 *−2.56–0.87*	0.37 −0.63–1.38	−0.50 *−3.67–2.68*	**−1.08 ** ***−1.88–−0.29***

The coeffecient indicates the changes of weight compared with the constant (period 1).

* period 1 (≤6 wk) is the reference category.

Data in italics indicate 95% confidence interval.

The data in **bold** indicate significant difference in weight change of reference category.

The increase in weight was significantly greater in exposure period 4 (>38 weeks) then in exposure period 1 (0–6 weeks) for FGA and olanzapine (see table S2) and forest plots S1–S16 in File S1 and File S2). For example, in the analysis of olanzapine, subjects gained 1.74 kilogram more weight (95% CI 0.50–2.99, p = 0.006) in exposure period 4 (>38 weeks) than in exposure period 1 (≤6 weeks). On the other hand, in the placebo group, patients lost weight in exposure period 4 and this was significantly different from the weight change in exposure period 1. Other AP did not show statistically significant changes in body weight over the consecutive exposure periods compared with exposure period 1.

### Weight change in AP-naive patients for each duration of exposure category

**Figure 3:**
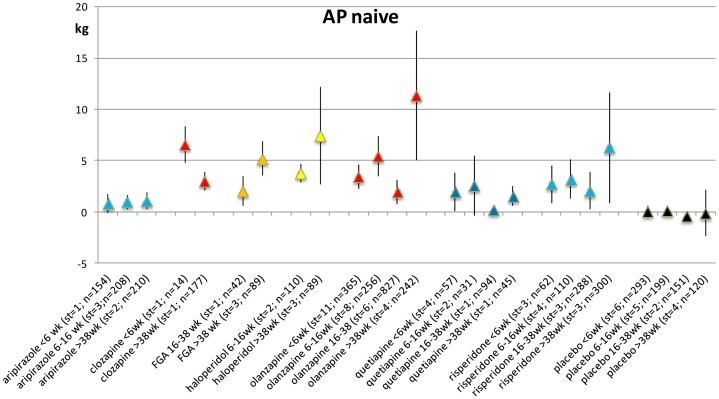
Weight change (kg) per period only including AP-naive samples.

Weight change post-baseline in AP-naive patients was limited to 39 studies, yielding 90 records. Data were available only for aripiprazole, chlorpromazine, clozapine, FGA, haloperidol, olanzapine, perphenazine, quetiapine, risperidone, SGA, sulpiride, ziprasidone and the placebo group. Figure 3 shows the weight change of the various AP within the group of AP-naive patients. Most associations between AP and weight gain were statistically significant at all exposure periods. For sulpiride, only 1 record was available (1.86, se 0.45; >38 wk, n = 162) and therefore not presented in the figure (Figure 3) [50]. For more detailed information see Table S3 and Forest plots S17–S24 in File S3). The short term period (≤6 weeks) showed substantial and statistically significant weight gain, olanzapine 3.42 kg, quetiapine 1.91 kg, risperidone 2.68 kg. I-square varied between 63.9% and 98.6% for meta-analysis. Weight was increased over time. Studies with 4 or more studies are presented.

For exposure period 4 (>38 weeks), patients receiving olanzapine gained significantly more weight than in exposure period 1 (See Table 2 and Table S3).

**Table 2:** Metaregression of weight changes per period in drug-naive patients.

Period	aripiprazole	olanzapine	Quetiapine	risperidone	placebo
≤6 wk*	0	0	0	0	0
6–16 wk	0.15 (*−1.55–1.27*)	1.30 (*−2.34–4.93*)	0.09 (*−6.47–6.65*)	−1.26 (*−6.83–4.32*)	0.24 (*−0.69–1.17*)
16–38 wk		−1.19 (*−5.00–2.63*)	−2.34 (*−9.95–5.27*)	−1.30 (−6.83–−4.23)	−0.38(*−1.48–0.71*)
>38 wk		**5.41 (** ***0.17–6.13*** **)**	−0.98 (*−8.70–6.74*)	2.31 (*−3.38–7.91*)	−0.36 (*−2.0–1.29*)

The coeffecient indicates the changes of weight compared with the constant (period 1).

*constant is period 1 that serves as reference in change.

Data in italics indicate 95% confidence interval.

The outcome in **bold** indicate significant difference in weight change of reference category.

### BMI change per duration of exposure category

**Figure 4:**
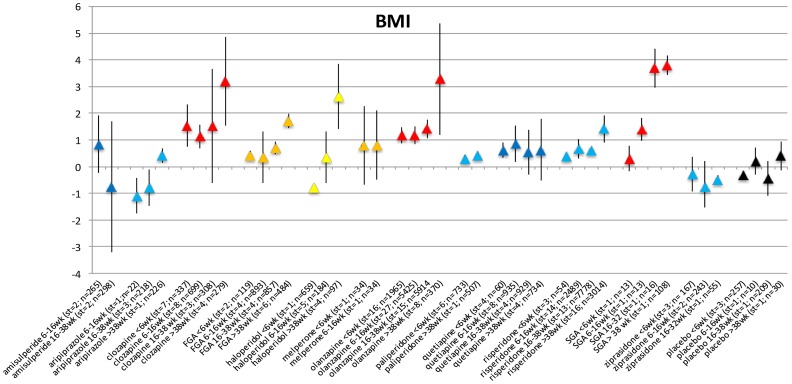
BMI change per period.

Ninety-one studies reported results on BMI change (227 records in the meta-analysis data). BMI increased over time after the start of a specific AP (Figure 4). Not all changes were statistically significant, likely due to the relatively low number of studies for each separate antipsychotic (Table S4 and Forest plots S25–S35 in File S4).

### BMI changes in AP-naive patients per duration of exposure category

**Figure 5:**
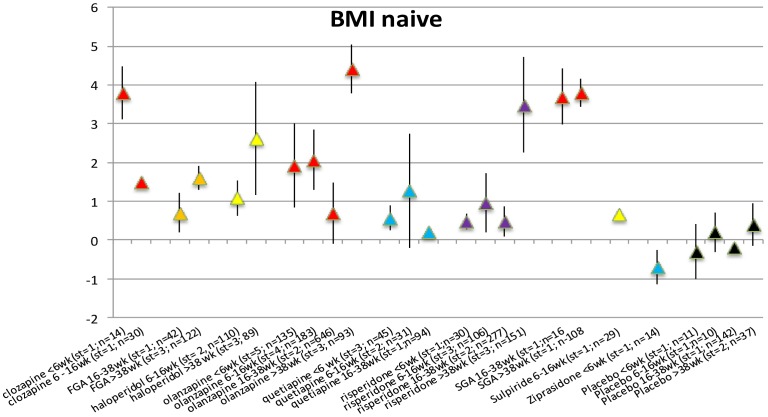
BMI change in AP naive patients per time period.

The number of studies reporting data on BMI change in AP-naive patients was limited to 18 studies with 51 records. All included AP showed a statistically significant increase in BMI (Figure 5). For quetiapine (6–16 weeks) and ziprasidone (<6 weeks), only a single exposure period was available in the data. Placebo did not result in increase of BMI over consecutive periods (Table S5 and Forest plots S36–S44 in File S5).

### 7% weight gain per duration of exposure category

**Figure 6:**
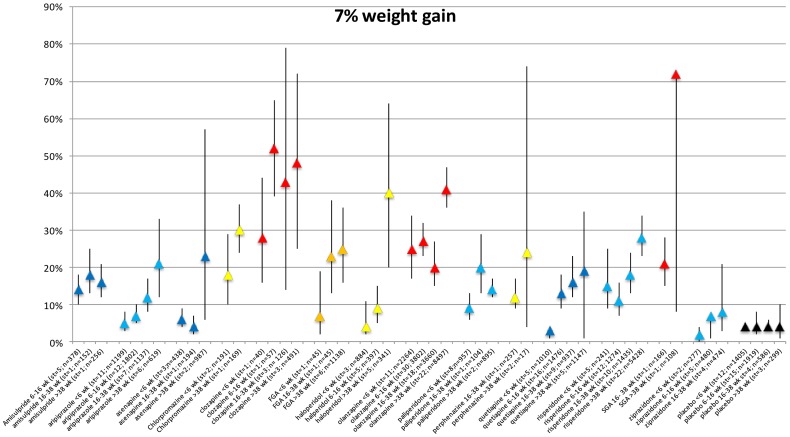
Proportion of weight increase per antipsychotic per time period.

There were 126 studies reporting on proportional weight gain (319 records in the meta-analysis data). The proportion of patients gaining more than 7% weight expanded with duration of AP use for each AP (Figure 6). The exception is the placebo group proportional weight increase remained constant at 4% during all exposure periods (Table S6 and Forest plots S44a–S58d in File S6 and File S7).

### 7% weight gain in AP-naive patients per duration of exposure category

**Figure 7:**
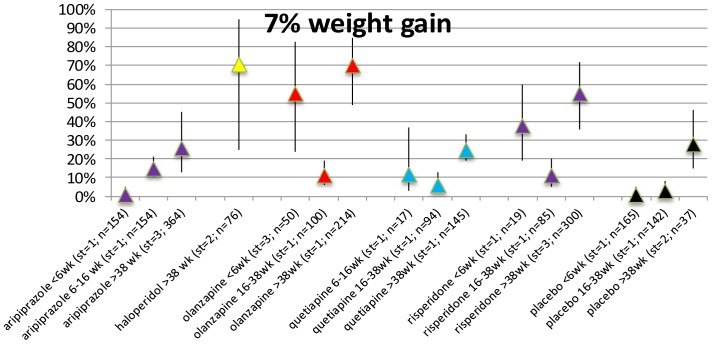
Proportion of weight increase in AP naïve.

The number of papers that presented data of 7% weight gain in antipsychotic naive patients is limited (11 studies with 32 records). For almost all included AP the proportion of subjects with clinically relevant weight gain is statistically significant (Figure 7). Apart from the short-term exposure period (≤6 weeks) treatment with aripiprazole resulted in an elevated number of subjects with clinically relevant weight gain at each duration of exposure category. The proportion of subjects gaining weight is also statistically significant in the placebo group after >38 weeks. For more detailed information see Table S7 Forest plots S59a–S64c in File S8.

### 7% weight loss per duration of exposure category

**Figure 8:**
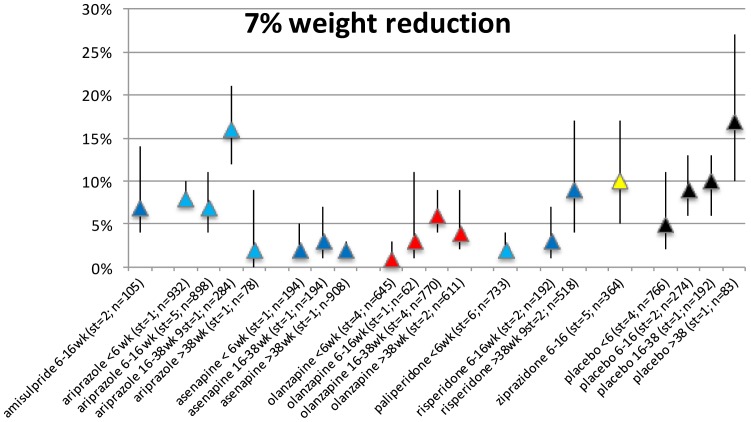
Proportion of weight reduction.

Twenty-four studies (representing 53 records) reported on proportional weight loss. Only data for amisulpride, aripiprazole, asenapine, olanzapine, paliperidone, ziprasidone and placebo were available (for 1 or more exposure periods, see Figure 8). Results showed that a statistically significant proportion of the patients had clinically relevant weight loss after the start of any of these AP, but visual inspection did not show a duration-response pattern (Table S8 and Forest plots S65–S72 in File S9).

## Discussion

### Main findings

This meta-analysis presents four outcome measures: (i) body weight change, (ii) BMI change, (iii) proportion of clinically relevant weight gain and (iv) proportion of clinically relevant weight loss for an extensive number of AP and within a subgroup of AP naive patients as well, simultaneously in one paper, The main result was that almost all AP showed a mean increase in body weight, BMI and a clinically relevant proportion of weight gain with increased duration of AP use, except for amisulpride, aripiprazole and ziprasidone which were weight neutral with duration of AP use. The AP-naive subgroup showed more robust increases of mean weight gain and BMI with duration of AP use. The proportion clinically of weight gain in the AP naive was at least 20% for all AP. In contrast, the outcome measure ‘proportion of clinically relevant weight loss’ showed a modest weight loss of around 10% for all AP studies, except aripiprazole showed clinically relevant weight loss in excess of 15%. Unfortunately, in the subgroup of AP-naive patients the proportion of clinically relevant weight loss could not be analysed because of lack of data. In conclusion, the present results add to the existing knowledge, showing that (i) duration of AP use is a modifying factor; (ii) that there are also AP users who lose weight; (iii) that AP switch to metabolically more neutral compounds may not result in weight loss in all cases; and (iv) that AP naive patients are more vulnerable to weight gain. This meets the criticism of Alvares-Jimemez that AP naive patients or first episode patients need to be studied separately from chronic patients [20].

The meta-regression analyses suggested that increased exposure to AP over time is associated with increased weight gain. Indicating that duration of AP exposure may be regarded as a causal factor, contributing to weight gain. Inspection of figure 1 to 8 suggests that other AP also display duration-response associations with weight gain.

### Perspectives

Although studies in AP-naive patients are more informative on weight gain induced by a specific AP, only one previous meta-analysis addressed the issue of weight change after start of an AP in AP-naive patients [26]. An increase in body weight and BMI for the combined group of all AP in AP-naive patients with schizophrenia over three duration categories of AP use was reported: 4–8 weeks, 10–12 weeks and 24–48 weeks [26]. This is in agreement with the present meta-analysis that showed that duration of AP use in AP naive patients resulted in weight gain. This result confirms also the direct impact of AP on weight gain.

Switching to an AP like amisulpride, aripiprazole or ziprasidone may not result in weight loss in all cases, as the mean weight change post-baseline according to this meta-analysis is neutral. On the other hand, the outcome “proportion of patients with weight loss” suggests that a considerable proportion of patients on aripiprazole, amisulpride or ziprasidone show significant weight loss after switching AP medication. However, this needs to be put into perspective: (i) the fact that the mean body weight change is neutral for these AP indicates that a comparable proportion will show weight gain, as shown in the outcome “proportion of clinically relevant weight gain” and (ii) this is based on the crude data, which are only suggestive, and may not be used for a direct comparison of AP's.

The consequence is that switching to another AP should be planned with care involving monitoring and evaluation of at least body weight and BMI, among other metabolic parameters [51]. Of interest is the outcome of proportion of clinically relevant weight loss (7% weight loss). It sheds light on the mean changes in body weight, as treatment with amisulpride, aripiprazole or ziprasidone resulted in a higher proportion of patients with clinically relevant weight loss, compared to other AP. Combining mean weight change, the proportion of clinically relevant weight gain and weight loss, offers a more precise impact of AP on body weight. The fact that treatment with aripiprazole resulted in only marginal mean weight loss after a switch, but occasioned a high percentage of patients losing >7% of their body weight indicates that a comparable proportion must experience >7% weight gain. The same is seen for olanzapine. Olanzapine shows a mean increase in body weight over the various duration categories, but even for this AP, a small proportion of patients showed clinically relevant weight loss.

Previous systematic reviews and meta-analyses reported that clozapine and olanzapine induce most severe weight gain [19], [20], [24], [26]. Amisulpride, aripiprazole and ziprasidone were weight neutral and may even result in some weight reduction [52]. A direct comparison between AP to calculate differences between the various AP was only presented by Rummel-Kluge, performing a head-to-head comparison [25] Leucht and colleagues [35] also showed that (i) all AP are associated with at least some weight gain compared with placebo and (ii) that olanzapine and clozapine have the most profound impact on weight gain. These two studies uniquely allow for a direct comparison between AP. However, it should be kept in mind that the study by Leucht and colleagues was restricted to patients with a diagnosis of schizophrenia, with a follow-up time of 4–12 weeks, and only presenting a single weight related outcome (measure mean body weight change). The short period of AP use and the diagnostic restrictions may explain some of the differences found between this study and the study by Leucht and colleagues [35].

The weight gain post-baseline for most AP in this study may not seem severe. Several factors may explain this finding. First, all diagnostic categories were included in the analyses. In most of the earlier meta-analyses, inclusion was restricted to severe mental illness, schizophrenia or bipolar disorders. Patients with SMI have an increased risk for metabolic problems like obesity [9], [53], [54]. Within the group of SMI, the risk for weight gain is more enhanced for schizophrenia than for bipolar disorder [55], [56]. Additionally, the level of weight change is predicted by baseline BMI. A low baseline BMI (≤27.5) results in a greater weight increase compared to high baseline BMI (>27.5) levels [57]. As most studies presented in the current study are switch studies, and the reason for a switch often is AP-related obesity, this would explain the relatively low impact by AP on weight change in the current study, most patient groups having a BMI>27.5. On the other hand, a continuous increase in body weight was observed over time, implying that switching from one AP to another AP has limited effect on body weight, even for AP like aripiprazole or amisulpride. Only ziprasidone may result in some weight loss. This issues needs to be addressed in more detail given its clinical importance.

A previous meta-analysis suggested that switching from higher to lower metabolic risk AP as a way of managing metabolic side should be conducted with care, considering the effect on psychopathology and other side effects [51]. Our findings also shed light on the effect of switching from so-called high to low metabolic risk AP. A proportion of patients may indeed benefit and lose weight. However, prolonged duration of AP use, the mean weight did not change. The principal message is that switching to an AP with a different metabolic risk profile does not always result in losing body weight. Psychiatrists should keep in mind that switching antipsychotics requires monitoring and evaluation [51] and may benefit from concurrent non-pharmacological interventions [58], [59].

### Methodological issues

In the present systematic review, only RCTs were included. In our view this is a legitimate choice because the RCT study design is generally accepted as gold standard [60]. However, some AP were not, or only once examined in an RCT. In addition, the RCT design also has its drawbacks. The patient group in an RCT is kept artificially homogeneous; all patients with comorbidity, using other medications or presenting with substance use problems tend to be excluded. Drop-out due to probable weight problems may also bias the results. Therefore, only ITT analyses were included, as a best possible correction procedure in the analyses. These factors impact on the generalizability of the results. In real life clinical practice therapeutic effects of the tested medications may be different and side-effects like weight changes may also be different because of co-medication and other factors. For these two reasons, future meta-analyses on various antipsychotics including and comparing, both RCTs and observational studies would be a welcome addition to the present meta-analysis. In addition, a follow-up meta-analysis, using the present data set, need to address direct comparison of weight change between AP and modifying factors, using modern analysis techniques like network analysis.

Despite the fact that various systematic reviews have been published before, this systematic review is the only meta-analysis that did not exclude any AP *a priori*. In addition, a clinically intuitive exposure period was used to assess the association between duration of AP intake and weight change. In spite of these advantages, several limitations apply.

First, despite the inclusion of 307 articles, the results for each AP separately were often based on very limited numbers of articles - one to three. This was occasioned by grouping length of AP use in 4 exposure periods. Data thus were particularly sparse for AP-naive groups. This calls for a careful interpretation of results. On the other hand, the results of various outcome measures all point in the same direction.

Second, the aim of the present meta-analysis was to test whether weight changes are significantly different from the null for each AP across the 4 exposure periods. For the purpose of analysis, in case of multiple outcomes per study, the last outcome assessment per exposure period (≤6 weeks, 6–16 weeks, 16–38 weeks, >38 weeks) per AP was selected, to avoid clustering in the data (also see statistical analysis).

Third, the definition of the 4 exposure categories is based on the average duration of exposure of the studies in the meta-analysis. Although the periods are chosen around the most common time frames presented in the studies, the demarcation is arbitrary. Most studies have fixed periods, however a substantial number of publications used average duration of study. This may have resulted in some measure of regression to the mean.

Fourth, weight gain in groups treated with AP could be the result of other medications like antidepressants or mood stabilisers. This problem is not present, as in studies that entered this meta-analysis, all other medications did not change during the study period, except the AP studied and control medication. Studies on weight change intervention were excluded.

Fifth, not all AP were included. Publications on older AP rarely describe data on the adverse event of weight change. Further, AP with a single reference, blonanserine, fluphenazine, levopromethazine, lurasidone, melperone, pimozide and zuclopentixol, similarly could not be included. Only papers published since 1999 were included, the year the meta-analysis by Allison [19] was published. This paper represented the start of a growing interest in metabolic side effects of AP, particularly weight gain. The current meta-analysis was originally designed as an extension of the Allison paper [19]. Unfortunately, first generation AP were mainly studied before 1999 and, therefore, information on weight is scarcer for some FGA.

Furthermore, in a large number of studies neither standard deviation nor standard errors of the continuous outcomes (weight change, BMI change) were available and standard error, therefore, was estimated using a formula (see methods section). Sensitivity analyses were performed to assess the impact of this on the final results (weight change and BMI change), assuming a worst case scenario (using the present data, it was possible to calculate the correlation between pre and post assessment if a study presented variances of both pre and post assessment as well as change score; in these studies the lowest correlation was 0.85 and this correlation was entered in the worst case scenario). Results of these sensitivity analyses were very similar to the original results (results available upon request). Sensitivity analyses removing all estimated standard errors were not informative because too few studies remained (results available upon request).

Sixth, although only RCTs using the intention-to-treat principle were included, some of the included studies had a long-term follow-up after ending the study. Because these long-term results were very important for the research question, we did include these data, despite the fact that they were not per analysed intention-to-treat. This means that for the long-term results bias, due to drop-out after weight gain is largest. In spite of this, weight gain in the long-term studies was largest. Therefore, contrary to the expected direction of results with bias, we found that AP were associated with weight gain and that weight gain was larger over time.

Finally, this study did not address the issue of differences in weight change across various diagnostic groups. Indeed some reviews address this issue although restricted to only schizophrenia and bipolar disorder [27], [61]. As mentioned in the introduction section, AP are more widely used and studied for various diagnoses other than schizophrenia of bipolar disorder. In this meta-analysis, the number of studies that only include either schizophrenia or bipolar disorder is limited. The diagnostic categories included in studies mostly pertain to combinations of various psychiatric diagnoses of schizophrenia, schizophreniform disorder, schizoaffective disorder, bipolar disorder, or depression. Given the complexity of the current study and the importance of the modifying factor diagnosis, a separate study to address this issue is required, but beyond the scope of the current analysis. Results reported in the present study provide a comprehensive overview of weight changes in all studies, but the reader has to keep in mind that associations may be stronger in specific diagnoses and weaker in others this meta-analysis. Therefore, interpretation might be done with caution considering the potential influence of diagnosis or AP doses on weight change.

## Conclusion

All AP show weight gain over time. The increase in weight varies per AP and per duration of exposure category, both in switch studies and in studies of AP-naive patients. The initial weight increase at ≤6 weeks is most important, as patients will not lose weight afterwards. The vast majority of the studies included are switch studies. This analysis does not suggest that switching AP is likely to result in weight reduction in the long term. Additionally, in AP-naive patients the short term weight gain is substantial for all AP, although the number of studies with AP-naive patients was limited. More work in AP-naive patients is of interest, particularly in FGA. Apart from haloperidol and chlorpromazine, FGA are poorly studied with respect to their metabolic effects. Lastly, given that haloperidol is not weight neutral, it is questionable whether it can serve as a good comparator AP in studies.

### List of studies per year of publication

1999 [62]–[68]; 2000 [69]–[73]; 2001 [74]–[90]; 2002 [37], [91]–[106]; 2003 [107]–[132]; 2004 [133]–[157]; 2005 [158]–[191]; 2006 [46], [192]–[218]; 2007 [219]–[254]; 2008 [45], [49], [255]–[286]; 2009 [287]–[317]; 2010 [43], [318]–[338]; 2011 [50], [339]–[359]

## Supporting Information

Checklist S1 **Prisma Checklist Meta-analysis.**
(DOC)

File S1 **Forest Plots S1–S8 Weight changes per exposure category.**
(ZIP)

File S2 **Forets Plots S9–S16 Weight changes per exposure category.**
(ZIP)

File S3 **Forest plots S17–S24. Weight changes in AP naives per exposure category.**
(ZIP)

File S4 **Forest Plots S25–S35. Change of BMI per exposure category.**
(ZIP)

File S5 **Forest Plots S36–S43. Changes of BMI in AP naives per exposure category.**
(ZIP)

File S6 **Forest Plots S44–S51d - Proportion (7%) of weight gain per exposure category.**
(ZIP)

File S7 **Forest Plots S52–S58 - Proportion (7%) of weight gain per exposure category.**
(ZIP)

File S8 **Forest plots S59a–S64c. Proportion (7%) of weight increase in AP naives per exposure category.**
(ZIP)

File S9 **Forest Plots S65–S72d. Proportion of 7% weight loss per exposure category.**
(ZIP)

Table S1 **Number of studies reporting on each of the antipsychotics (switch studies and drug naive separately).**
(DOCX)

Table S2 **Weight changes per exposure category.**
(DOCX)

Table S3 **Weight changes in AP naives per exposure category.**
(DOCX)

Table S4 **Change of BMI per exposure category.**
(DOCX)

Table S5 **Change of BMI in AP naives per exposure category.**
(DOCX)

Table S6 **Proportion (7%) of weight gain per exposure category.**
(DOCX)

Table S7 **Proportion (7%) of weight increase in AP naives per exposure category.**
(DOCX)

Table S8 **Proportion of 7% weight loss per exposure category.**
(DOCX)

## References

[1] De Hert M, van Winkel R, Van Eyck D, Hanssens L, Wampers M, et al (2006) Prevalence of diabetes, metabolic syndrome and metabolic abnormalities in schizophrenia over the course of the illness: a cross-sectional study. Clin Pract Epidemol Ment Health 2: 14. 10.1186/1745-0179-2-14 PMC1533826 16803620

[2] Henderson DC (2008) Managing weight gain and metabolic issues in patients treated with atypical antipsychotics. J Clin Psychiatry 69: e04. 18363448 10.4088/jcp.0208e04

[3] Huxley R, Mendis S, Zheleznyakov E, Reddy S, Chan J (2010) Body mass index, waist circumference and waist:hip ratio as predictors of cardiovascular risk–a review of the literature. Eur J Clin Nutr 64: 16–22. 19654593 10.1038/ejcn.2009.68

[4] Meltzer HY (2001) Putting metabolic side effects into perspective: risks versus benefits of atypical antipsychotics. J Clin Psychiatry 62 Suppl 27: 35–39 discussion 40-31. 11806488

[5] Newcomer JW, Haupt DW (2006) The metabolic effects of antipsychotic medications. Can J Psychiatry 51: 480–491. 16933585 10.1177/070674370605100803

[6] Beary M, Hodgson R, Wildgust HJ (2012) A critical review of major mortality risk factors for all-cause mortality in first-episode schizophrenia: clinical and research implications. J Psychopharmacol 26: 52–61. 22465947 10.1177/0269881112440512

[7] Seidell JC (2010) Waist circumference and waist/hip ratio in relation to all-cause mortality, cancer and sleep apnea. Eur J Clin Nutr 64: 35–41. 19639001 10.1038/ejcn.2009.71

[8] Fleischhacker WW, Cetkovich-Bakmas M, De Hert M, Hennekens CH, Lambert M, et al (2008) Comorbid somatic illnesses in patients with severe mental disorders: clinical, policy, and research challenges. J Clin Psychiatry 69: 514–519. 18370570 10.4088/jcp.v69n0401

[9] Holt RI, Peveler RC (2009) Obesity, serious mental illness and antipsychotic drugs. Diabetes Obes Metab 11: 665–679. 19476478 10.1111/j.1463-1326.2009.01038.x

[10] Newcomer JW (2007) Antipsychotic medications: metabolic and cardiovascular risk. J Clin Psychiatry 68 Suppl 4: 8–13. 17539694

[11] Lahti M, Tiihonen J, Wildgust H, Beary M, Hodgson R, et al (2012) Cardiovascular morbidity, mortality and pharmacotherapy in patients with schizophrenia. Psychol Med 1–11. 10.1017/S0033291712000396 22405504

[12] Saha S, Chant D, McGrath J (2007) A systematic review of mortality in schizophrenia: is the differential mortality gap worsening over time? Arch Gen Psychiatry 64: 1123–1131. 17909124 10.1001/archpsyc.64.10.1123

[13] Tiihonen J, Lonnqvist J, Wahlbeck K, Klaukka T, Niskanen L, et al (2009) 11-year follow-up of mortality in patients with schizophrenia: a population-based cohort study (FIN11 study). Lancet 374: 620–627. 19595447 10.1016/S0140-6736(09)60742-X

[14] Enger C, Weatherby L, Reynolds RF, Glasser DB, Walker AM (2004) Serious cardiovascular events and mortality among patients with schizophrenia. J Nerv Ment Dis 192: 19–27. 14718772 10.1097/01.nmd.0000105996.62105.07

[15] Osby U, Brandt L, Correia N, Ekbom A, Sparen P (2001) Excess mortality in bipolar and unipolar disorder in Sweden. Arch Gen Psychiatry 58: 844–850. 11545667 10.1001/archpsyc.58.9.844

[16] Tiihonen K, Ouwehand AC, Rautonen N (2010) Effect of overweight on gastrointestinal microbiology and immunology: correlation with blood biomarkers. Br J Nutr 103: 1070–1078. 19930761 10.1017/S0007114509992807

[17] Weinmann S, Read J, Aderhold V (2009) Influence of antipsychotics on mortality in schizophrenia: systematic review. Schizophr Res 113: 1–11. 19524406 10.1016/j.schres.2009.05.018

[18] Newcomer JW (2009) Comparing the safety and efficacy of atypical antipsychotics in psychiatric patients with comorbid medical illnesses. J Clin Psychiatry 70 Suppl 3: 30–36. 19570499 10.4088/JCP.7075su1c.05

[19] Allison DB, Mentore JL, Heo M, Chandler LP, Cappelleri JC, et al (1999) Antipsychotic-induced weight gain: a comprehensive research synthesis. Am J Psychiatry 156: 1686–1696. 10553730 10.1176/ajp.156.11.1686

[20] Alvarez-Jimenez M, Gonzalez-Blanch C, Crespo-Facorro B, Hetrick S, Rodriguez-Sanchez JM, et al (2008) Antipsychotic-induced weight gain in chronic and first-episode psychotic disorders: a systematic critical reappraisal. CNS Drugs 22: 547–562. 18547125 10.2165/00023210-200822070-00002

[21] Jones B, Basson BR, Walker DJ, Crawford AM, Kinon BJ (2001) Weight change and atypical antipsychotic treatment in patients with schizophrenia. J Clin Psychiatry 62 Suppl 2: 41–44. 11232752

[22] Klemp M, Tvete IF, Skomedal T, Gaasemyr J, Natvig B, et al (2011) A review and Bayesian meta-analysis of clinical efficacy and adverse effects of 4 atypical neuroleptic drugs compared with haloperidol and placebo. J Clin Psychopharmacol 31: 698–704. 22020356 10.1097/JCP.0b013e31823657d9

[23] McIntyre RS, McCann SM, Kennedy SH (2001) Antipsychotic metabolic effects: weight gain, diabetes mellitus, and lipid abnormalities. Can J Psychiatry 46: 273–281. 11320682 10.1177/070674370104600308

[24] Parsons B, Allison DB, Loebel A, Williams K, Giller E, et al (2009) Weight effects associated with antipsychotics: a comprehensive database analysis. Schizophr Res 110: 103–110. 19321312 10.1016/j.schres.2008.09.025

[25] Rummel-Kluge C, Komossa K, Schwarz S, Hunger H, Schmid F, et al (2010) Second-Generation Antipsychotic Drugs and Extrapyramidal Side Effects: A Systematic Review and Meta-analysis of Head-to-Head Comparisons. Schizophr Bull 10.1093/schbul/sbq042 PMC3245581 20513652

[26] Tarricone I, Ferrari Gozzi B, Serretti A, Grieco D, Berardi D (2010) Weight gain in antipsychotic-naive patients: a review and meta-analysis. Psychol Med 40: 187–200. 19656426 10.1017/S0033291709990407

[27] Allison DB, Newcomer JW, Dunn AL, Blumenthal JA, Fabricatore AN, et al (2009) Obesity among those with mental disorders: a National Institute of Mental Health meeting report. Am J Prev Med 36: 341–350. 19285199 10.1016/j.amepre.2008.11.020

[28] Citrome L, Blonde L, Damatarca C (2005) Metabolic issues in patients with severe mental illness. South Med J 98: 714–720. 16108240 10.1097/01.smj.0000167621.49292.11

[29] Gentile S (2009) Contributing factors to weight gain during long-term treatment with second-generation antipsychotics. A systematic appraisal and clinical implications. Obes Rev 10: 527–542. 19460111 10.1111/j.1467-789X.2009.00589.x

[30] Johnsen E, Jorgensen HA (2008) Effectiveness of second generation antipsychotics: a systematic review of randomized trials. BMC Psychiatry 8: 31. 18439263 10.1186/1471-244X-8-31 PMC2386457

[31] Leucht S, Kissling W, Davis JM (2009) Second-generation antipsychotics for schizophrenia: can we resolve the conflict? Psychol Med 39: 1591–1602. 19335931 10.1017/S0033291709005455

[32] Starrenburg FC, Bogers JP (2009) How can antipsychotics cause Diabetes Mellitus? Insights based on receptor-binding profiles, humoral factors and transporter proteins. Eur Psychiatry 24: 164–170. 19285836 10.1016/j.eurpsy.2009.01.001

[33] Kim DH, Maneen MJ, Stahl SM (2009) Building a better antipsychotic: receptor targets for the treatment of multiple symptom dimensions of schizophrenia. Neurotherapeutics 6: 78–85. 19110200 10.1016/j.nurt.2008.10.020 PMC5084257

[34] Panariello F, De Luca V, de Bartolomeis A (2011) Wegt gain, schizophrenia and antispychotics: New findings from animal model and pharmacogenomic studies. Schizophrenia Research & Treatment. pp. 16. 10.1155/2011/459284 PMC3440684 22988505

[35] Leucht S, Cipriani A, Spineli L, Mavridis D, Orey D, et al (2013) Comparative efficacy and tolerability of 15 antipsychotic drugs in schizophrenia: a multiple-treatments meta-analysis. Lancet 382: 951–962. 23810019 10.1016/S0140-6736(13)60733-3

[36] Stroup DF, Berlin JA, Morton SC, Olkin I, Williamson GD, et al (2000) Meta-analysis of observational studies in epidemiology: a proposal for reporting. Meta-analysis Of Observational Studies in Epidemiology (MOOSE) group. JAMA 283: 2008–2012. 10789670 10.1001/jama.283.15.2008

[37] Breier A, Sutton VK, Feldman PD, Kadam DL, Ferchland I, et al (2002) Olanzapine in the treatment of dopamimetic-induced psychosis in patients with Parkinson's disease. Biol Psychiatry 52: 438–445. 12242060 10.1016/s0006-3223(02)01392-6

[38] Drukker M, Bak M, Campo J, Driessen G, Van Os J, et al (2010) The cumulative needs for care monitor: a unique monitoring system in the south of the Netherlands. Soc Psychiatry Psychiatr Epidemiol 45: 475–485. 19572089 10.1007/s00127-009-0088-3 PMC2834763

[39] Statacorp (2012) Statistical Software: release 12. College Station, TX: Stata Corporation.

[40] Bradburn MJ, Deeks JJ, Altman JJ (2009) Metan - a command for meta-analysis in Stata. In: ed SJAC, editor. Meta-analysis in Stata: An updated collection from the Stata Journal. College Station, Texas: Stata Press Publications.

[41] R Core Team (2013) R: A Language and Environment for Statistical Computing. In: Computing RFfS, editor. Vienna, Austria.

[42] Apiquian R, Fresan A, Ulloa RE, de la Fuente-Sandoval C, Herrera-Estrella M, et al (2005) Amoxapine as an atypical antipsychotic: a comparative study vs risperidone. Neuropsychopharmacology 30: 2236–2244. 15956984 10.1038/sj.npp.1300796

[43] Yang J, Bahk WM, Cho HS, Jeon YW, Jon DI, et al (2010) Efficacy and tolerability of Blonanserin in the patients with schizophrenia: a randomized, double-blind, risperidone-compared trial. Clin Neuropharmacol 33: 169–175. 20661022 10.1097/WNF.0b013e3181dcda50

[44] Conley RR, Kelly DL, Nelson MW, Richardson CM, Feldman S, et al (2005) Risperidone, quetiapine, and fluphenazine in the treatment of patients with therapy-refractory schizophrenia. Clin Neuropharmacol 28: 163–168. 16062094 10.1097/01.wnf.0000172993.89879.0f

[45] Kane JM, Lauriello J, Laska E, Di Marino M, Wolfgang CD (2008) Long-term efficacy and safety of iloperidone: results from 3 clinical trials for the treatment of schizophrenia. J Clin Psychopharmacol 28: S29–35. 18334910 10.1097/JCP.0b013e318169cca7

[46] Lal S, Thavundayil JX, Nair NP, Annable L, Ng Ying Kin NM, et al (2006) Levomepromazine versus chlorpromazine in treatment-resistant schizophrenia: a double-blind randomized trial. J Psychiatry Neurosci 31: 271–279. 16862245 PMC1488906

[47] Potkin SG, Ogasa M, Cucchiaro J, Loebel A (2011) Double-blind comparison of the safety and efficacy of lurasidone and ziprasidone in clinically stable outpatients with schizophrenia or schizoaffective disorder. Schizophr Res 132: 101–107. 21889878 10.1016/j.schres.2011.04.008

[48] Bruggeman R, van der Linden C, Buitelaar JK, Gericke GS, Hawkridge SM, et al (2001) Risperidone versus pimozide in Tourette's disorder: a comparative double-blind parallel-group study. J Clin Psychiatry 62: 50–56. 10.4088/jcp.v62n0111 11235929

[49] Haessler F, Glaser T, Pap AF, Diefenbacher A, Rets O (2008) A double-blind placebo-controlled discontinuation study of zuclopenthixol for the theratment of agressive and disruptive behaviours in adults with mental retardation: Seondary parameter analyses. Pharmacopsychiatry 41: 232–239. 19067260 10.1055/s-0028-1082072

[50] Guo X, Fang M, Zhai J, Wang B, Wang C, et al (2011) Effectiveness of maintenance treatments with atypical and typical antipsychotics in stable schizophrenia with early stage: 1-year naturalistic study. Psychopharmacology (Berl) 216: 475–484. 21369751 10.1007/s00213-011-2242-3

[51] Newcomer JW, Weiden PJ, Buchanan RW (2013) Switching antipsychotic medications to reduce adverse event burden in schizophrenia: establishing evidence-based practice. J Clin Psychiatry 74: 1108–1120. 24330898 10.4088/JCP.12028ah1

[52] Newcomer JW (2004) Metabolic risk during antipsychotic treatment. Clin Ther 26: 1936–1946. 15823759 10.1016/j.clinthera.2004.12.003

[53] Dickerson FB, Brown CH, Kreyenbuhl JA, Fang L, Goldberg RW, et al (2006) Obesity among individuals with serious mental illness. Acta psychiatrica Scandinavica 113: 306–313. 16638075 10.1111/j.1600-0447.2005.00637.x

[54] McElroy SL (2009) Obesity in patients with severe mental illness: overview and management. The Journal of clinical psychiatry 70 Suppl 3: 12–21. 10.4088/JCP.7075su1c.03 19570497

[55] De Hert M, Schreurs V, Vancampfort D, Van Winkel R (2009) Metabolic syndrome in people with schizophrenia: a review. World psychiatry : official journal of the World Psychiatric Association 8: 15–22. 10.1002/j.2051-5545.2009.tb00199.x PMC2656262 19293950

[56] Mitchell AJ, Vancampfort D, Sweers K, van Winkel R, Yu W, et al (2013) Prevalence of metabolic syndrome and metabolic abnormalities in schizophrenia and related disorders–a systematic review and meta-analysis. Schizophr Bull 39: 306–318. 22207632 10.1093/schbul/sbr148 PMC3576174

[57] Bushe CJ, Slooff CJ, Haddad PM, Karagianis JL (2013) Weight change by baseline BMI from three-year observational data: findings from the Worldwide Schizophrenia Outpatient Health Outcomes Database. Journal of psychopharmacology 10.1177/0269881112473789 23343595

[58] Alvarez-Jimenez M, Hetrick SE, Gonzalez-Blanch C, Gleeson JF, McGorry PD (2008) Non-pharmacological management of antipsychotic-induced weight gain: systematic review and meta-analysis of randomised controlled trials. Br J Psychiatry 193: 101–107. 18669990 10.1192/bjp.bp.107.042853

[59] Daumit GL, Dickerson FB, Appel LJ (2013) Weight loss in persons with serious mental illness. N Engl J Med 369: 486–487. 10.1056/NEJMc1306994 23902501

[60] Rothman KJ, Greensland S (1998) Modern Epidemiology. Philidelphia: Lippinscott-Raven.

[61] De Hert M, Detraux J, van Winkel R, Yu W, Correll CU (2012) Metabolic and cardiovascular adverse effects associated with antipsychotic drugs. Nat Rev Endocrinol 8: 114–126. 10.1038/nrendo.2011.156 22009159

[62] Osser DN, Najarian DM, Dufresne RL (1999) Olanzapine increases weight and serum triglyceride levels. J Clin Psychiatry 60: 767–770. 10584766 10.4088/jcp.v60n1109

[63] Peuskens J, Bech P, Moller HJ, Bale R, Fleurot O, et al (1999) Amisulpride vs. risperidone in the treatment of acute exacerbations of schizophrenia. Amisulpride study group. Psychiatry Res 88: 107–117. 10622347 10.1016/s0165-1781(99)00075-x

[64] Revicky DA, Genduso LA, Hamilton AH, Ganzoczy D, Beasly CM Jr (1999) Olanzapine versus haloperidol in thetreatment of schizophrenia and other psychotic disorders: Quality of life and clinical outcomes of a randomized clinical trial. Quality of Life Research 8: 417–426. 10474283 10.1023/a:1008958925848

[65] Schulz SC, Camlin KL, Berry SA, Jesberger JA (1999) Olanzapine safety and efficacy in patients with borderline personality disorder and comorbid dysthymia. Biol Psychiatry 46: 1429–1435. 10578457 10.1016/s0006-3223(99)00128-6

[66] Spivak B, Musin E, Mester R, Gonen N, Talmon Y, et al (1999) The effect of long-term antipsychotic treatment on the body weight of patients suffering from chronic schizophrenia: clozapine versus classical antipsychotic agents. Int Clin Psychopharmacol 14: 229–232. 10468315 10.1097/00004850-199907000-00004

[67] Tran PV, Tollefson GD, Sanger TM, Lu Y, Berg PH, et al (1999) Olanzapine versus haloperidol in the treatment of schizoaffective disorder. Acute and long-term therapy. Br J Psychiatry 174: 15–22. 10211146 10.1192/bjp.174.1.15

[68] Wirshing DA, Wirshing WC, Kysar L, Berisford MA, Goldstein D, et al (1999) Novel antipsychotics: comparison of weight gain liabilities. J Clin Psychiatry 60: 358–363. 10401912

[69] Guille C, Sachs GS, Ghaemi SN (2000) A naturalistic comparison of clozapine, risperidone, and olanzapine in the treatment of bipolar disorder. J Clin Psychiatry 61: 638–642. 11030483 10.4088/jcp.v61n0907

[70] Henderson DC, Cagliero E, Gray C, Nasrallah RA, Hayden DL, et al (2000) Clozapine, diabetes mellitus, weight gain, and lipid abnormalities: A five-year naturalistic study. Am J Psychiatry 157: 975–981. 10831479 10.1176/appi.ajp.157.6.975

[71] Kinon BJ, Basson BR, Gilmore JA, Malcolm S, Stauffer VL (2000) Strategies for switching from conventional antipsychotic drugs or risperidone to olanzapine. J Clin Psychiatry 61: 833–840. 11105736 10.4088/jcp.v61n1105

[72] Tariot PN, Salzman C, Yeung PP, Pultz J, Rak IW (2000) Long-Term use of quetiapine in elderly patients with psychotic disorders. Clin Ther 22: 1068–1084. 11048905 10.1016/s0149-2918(00)80085-5

[73] Tohen M, Jacobs TG, Grundy SL, McElroy SL, Banov MC, et al (2000) Efficacy of olanzapine in acute bipolar mania: a double-blind, placebo-controlled study. The Olanzipine HGGW Study Group. Arch Gen Psychiatry 57: 841–849. 10986547 10.1001/archpsyc.57.9.841

[74] Azorin JM, Spiegel R, Remington G, Vanelle JM, Pere JJ, et al (2001) A double-blind comparative study of clozapine and risperidone in the management of severe chronic schizophrenia. Am J Psychiatry 158: 1305–1313. 11481167 10.1176/appi.ajp.158.8.1305

[75] Basson BR, Kinon BJ, Taylor CC, Szymanski KA, Gilmore JA, et al (2001) Factors influencing acute weight change in patients with schizophrenia treated with olanzapine, haloperidol, or risperidone. J Clin Psychiatry 62: 231–238. 11379836 10.4088/jcp.v62n0404

[76] Budman CL, Gayer A, Lesser M, Shi Q, Bruun RD (2001) An open-label study of the treatment efficacy of olanzapine for Tourette's disorder. J Clin Psychiatry 62: 290–294. 11379844 10.4088/jcp.v62n0412

[77] Butterfield MI, Becker ME, Connor KM, Sutherland S, Churchill LE, et al (2001) Olanzapine in the treatment of post-traumatic stress disorder: a pilot study. Int Clin Psychopharmacol 16: 197–203. 11459333 10.1097/00004850-200107000-00003

[78] Conley RR, Mahmoud R (2001) A randomized double-blind study of risperidone and olanzapine in the treatment of schizophrenia or schizoaffective disorder. Am J Psychiatry 158: 765–774. 11329400 10.1176/appi.ajp.158.5.765

[79] Dossenbach MR, Kratky P, Schneidman M, Grundy SL, Metcalfe S, et al (2001) Evidence for the effectiveness of olanzapine among patients nonresponsive and/or intolerant to risperidone. J Clin Psychiatry 62 Suppl 2: 28–34. 11232749

[80] Herran A, Garcia-Unzueta MT, Amado JA, de La Maza MT, Alvarez C, et al (2001) Effects of long-term treatment with antipsychotics on serum leptin levels. Br J Psychiatry 179: 59–62. 11435270 10.1192/bjp.179.1.59

[81] Kingsbury SJ, Fayek M, Trufasiu D, Zada J, Simpson GM (2001) The apparent effects of ziprasidone on plasma lipids and glucose. J Clin Psychiatry 62: 347–349. 11411816 10.4088/jcp.v62n0507

[82] Kinon BJ, Basson BR, Gilmore JA, Tollefson GD (2001) Long-term olanzapine treatment: weight change and weight-related health factors in schizophrenia. J Clin Psychiatry 62: 92–100. 11247108

[83] Lindenmayer JP, Volavka J, Lieberman J, Sheitman B, Citrome L, et al (2001) Olanzapine for schizophrenia refractory to typical and atypical antipsychotics: an open-label, prospective trial. J Clin Psychopharmacol 21: 448–453. 11476131 10.1097/00004714-200108000-00014

[84] Sajatovic M, Brescan DW, Perez DE, DiGiovanni SK, Hattab H, et al (2001) Quetiapine alone and added to a mood stabilizer for serious mood disorders. J Clin Psychiatry 62: 728–732. 11681770 10.4088/jcp.v62n0911

[85] Sanger TM, Grundy SL, Gibson PJ, Namjoshi MA, Greaney MG, et al (2001) Long-term olanzapine therapy in the treatment of bipolar I disorder: an open-label continuation phase study. J Clin Psychiatry 62: 273–281. 11379842 10.4088/jcp.v62n0410

[86] Simpson MM, Goetz RR, Devlin MJ, Goetz SA, Walsh BT (2001) Weight gain and antipsychotic medication: differences between antipsychotic-free and treatment periods. J Clin Psychiatry 62: 694–700. 11681765 10.4088/jcp.v62n0906

[87] Street JS, Clark WS, Kadam DL, Mitan SJ, Juliar BE, et al (2001) Long-term efficacy of olanzapine in the control of psychotic and behavioral symptoms in nursing home patients with Alzheimer's dementia. Int J Geriatr Psychiatry 16 Suppl 1: S62–70. 11748789 10.1002/1099-1166(200112)16:1+<::aid-gps569>3.0.co;2-j

[88] Tollefson GD, Birkett MA, Kiesler GM, Wood AJ (2001) Double-blind comparison of olanzapine versus clozapine in schizophrenic patients clinically eligible for treatment with clozapine. Biol Psychiatry 49: 52–63. 11163780 10.1016/s0006-3223(00)01026-x

[89] Yap HL, Mahendran R, Lim D, Liow PH, Lee A, et al (2001) Risperidone in the treatment of first episode psychosis. Singapore Med J 42: 170–173. 11465317

[90] Zanarini MC, Frankenburg FR (2001) Olanzapine treatment of female borderline personality disorder patients: a double-blind, placebo-controlled pilot study. J Clin Psychiatry 62: 849–854. 11775043 10.4088/jcp.v62n1103

[91] Barak Y (2002) No weight gain among elderly schizophrenia patients after 1 year of risperidone treatment. J Clin Psychiatry 63: 117–119. 11874211 10.4088/jcp.v63n0205

[92] Barak Y, Shamir E, Zemishlani H, Mirecki I, Toren P, et al (2002) Olanzapine vs. haloperidol in the treatment of elderly chronic schizophrenia patients. Prog Neuropsychopharmacol Biol Psychiatry 26: 1199–1202. 12452546 10.1016/s0278-5846(01)00322-0

[93] Baymiller SP, Ball P, McMahon RP, Buchanan RW (2002) Weight and blood pressure change during clozapine treatment. Clin Neuropharmacol 25: 202–206. 12151907 10.1097/00002826-200207000-00003

[94] Czobor P, Volavka J, Sheitman B, Lindenmayer JP, Citrome L, et al (2002) Antipsychotic-induced weight gain and therapeutic response: a differential association. J Clin Psychopharmacol 22: 244–251. 12006893 10.1097/00004714-200206000-00003

[95] Gothelf D, Falk B, Singer P, Kairi M, Phillip M, et al (2002) Weight gain associated with increased food intake and low habitual activity levels in male adolescent schizophrenic inpatients treated with olanzapine. Am J Psychiatry 159: 1055–1057. 12042200 10.1176/appi.ajp.159.6.1055

[96] Hirsch SR, Kissling W, Bauml J, Power A, O'Connor R (2002) A 28-week comparison of ziprasidone and haloperidol in outpatients with stable schizophrenia. J Clin Psychiatry 63: 516–523. 12088164 10.4088/jcp.v63n0609

[97] Kane JM, Carson WH, Saha AR, McQuade RD, Ingenito GG, et al (2002) Efficacy and safety of aripiprazole and haloperidol versus placebo in patients with schizophrenia and schizoaffective disorder. J Clin Psychiatry 63: 763–771. 12363115 10.4088/jcp.v63n0903

[98] Lee CT, Conde BJ, Mazlan M, Visanuyothin T, Wang A, et al (2002) Switching to olanzapine from previous antipsychotics: a regional collaborative multicenter trial assessing 2 switching techniques in Asia Pacific. J Clin Psychiatry 63: 569–576. 12143912 10.4088/jcp.v63n0706

[99] Lindenmayer JP, Czobor P, Volavka J, Lieberman JA, Citrome L, et al (2002) Olanzapine in refractory schizophrenia after failure of typical or atypical antipsychotic treatment: an open-label switch study. J Clin Psychiatry 63: 931–935. 12416603 10.4088/jcp.v63n1011

[100] Llorca PM, Lancon C, Disdier B, Farisse J, Sapin C, et al (2002) Effectiveness of clozapine in neuroleptic-resistant schizophrenia: clinical response and plasma concentrations. J Psychiatry Neurosci 27: 30–37. 11836974 PMC149793

[101] Margolese HC, Chouinard G, Beauclair L, Belanger MC (2002) Therapeutic tolerance and rebound psychosis during quetiapine maintenance monotherapy in patients with schizophrenia and schizoaffective disorder. J Clin Psychopharmacol 22: 347–352. 12172332 10.1097/00004714-200208000-00003

[102] Martin S, Ljo H, Peuskens J, Thirumalai S, Giudicelli A, et al (2002) A double-blind, randomised comparative trial of amisulpride versus olanzapine in the treatment of schizophrenia: short-term results at two months. Curr Med Res Opin 18: 355–362. 12442883 10.1185/030079902125001128

[103] Rodriguez-Perez V, Lopez A, Blanco C, Pena C, Abel A, et al (2002) Olanzapine for the treatment of chronic refractory schizophrenia: a 12-month follow-up naturalistic study. Prog Neuropsychopharmacol Biol Psychiatry 26: 1055–1062. 12452526 10.1016/s0278-5846(02)00222-1

[104] Sechter D, Peuskens J, Fleurot O, Rein W, Lecrubier Y (2002) Amisulpride vs. risperidone in chronic schizophrenia: results of a 6-month double-blind study. Neuropsychopharmacology 27: 1071–1081. 12464464 10.1016/S0893-133X(02)00375-5

[105] Sowell MO, Mukhopadhyay N, Cavazzoni P, Shankar S, Steinberg HO, et al (2002) Hyperglycemic clamp assessment of insulin secretory responses in normal subjects treated with olanzapine, risperidone, or placebo. J Clin Endocrinol Metab 87: 2918–2923. 12050274 10.1210/jcem.87.6.8599

[106] Tauscher-Wisniewski S, Kapur S, Tauscher J, Jones C, Daskalakis ZJ, et al (2002) Quetiapine: an effective antipsychotic in first-episode schizophrenia despite only transiently high dopamine-2 receptor blockade. J Clin Psychiatry 63: 992–997. 12444812 10.4088/jcp.v63n1106

[107] Apiquian R, Ulloa E, Fresan A, Loyzaga C, Nicolini H, et al (2003) Amoxapine shows atypical antipsychotic effects in patients with schizophrenia: results from a prospective open-label study. Schizophr Res 59: 35–39. 12413640 10.1016/s0920-9964(01)00342-5

[108] Baldessarini RJ, Hennen J, Wilson M, Calabrese J, Chengappa R, et al (2003) Olanzapine versus placebo in acute mania: treatment responses in subgroups. J Clin Psychopharmacol 23: 370–376. 12920413 10.1097/01.jcp.0000085410.08426.9a

[109] Beasley CM Jr, Sutton VK, Hamilton SH, Walker DJ, Dossenbach M, et al (2003) A double-blind, randomized, placebo-controlled trial of olanzapine in the prevention of psychotic relapse. J Clin Psychopharmacol 23: 582–594. 14624189 10.1097/01.jcp.0000095348.32154.ec

[110] Bobes J, Rejas J, Garcia-Garcia M, Rico-Villademoros F, Garcia-Portilla MP, et al (2003) Weight gain in patients with schizophrenia treated with risperidone, olanzapine, quetiapine or haloperidol: results of the EIRE study. Schizophr Res 62: 77–88. 12765747 10.1016/s0920-9964(02)00431-0

[111] Casey DE (2005) Metabolic issues and cardiovascular disease in patients with psychiatric disorders. Am J Med 118 Suppl 2: 15S–22S. 15903291 10.1016/j.amjmed.2005.01.046

[112] Chengappa KN, Parepally H, Brar JS, Mullen J, Shilling A, et al (2003) A random-assignment, double-blind, clinical trial of once- vs twice-daily administration of quetiapine fumarate in patients with schizophrenia or schizoaffective disorder: a pilot study. Can J Psychiatry 48: 187–194. 12728743 10.1177/070674370304800307

[113] Chiu NY, Yang YK, Chen PS, Chang CC, Lee IH, et al (2003) Olanzapine in Chinese treatment-resistant patients with schizophrenia: an open-label, prospective trial. Psychiatry Clin Neurosci 57: 478–484. 12950701 10.1046/j.1440-1819.2003.01151.x

[114] Godleski LS, Goldsmith LJ, Vieweg WV, Zettwoch NC, Stikovac DM, et al (2003) Switching from depot antipsychotic drugs to olanzapine in patients with chronic schizophrenia. J Clin Psychiatry 64: 119–122. 12633119 10.4088/jcp.v64n0203

[115] Hwang JP, Yang CH, Lee TW, Tsai SJ (2003) The efficacy and safety of olanzapine for the treatment of geriatric psychosis. J Clin Psychopharmacol 23: 113–118. 12640211 10.1097/00004714-200304000-00002

[116] Hwang TJ, Lee SM, Sun HJ, Lin HN, Tsai SJ, et al (2003) Amisulpride versus risperidone in the treatment of schizophrenic patients: a double-blind pilot study in Taiwan. J Formos Med Assoc 102: 30–36. 12684609

[117] Jeste DV, Barak Y, Madhusoodanan S, Grossman F, Gharabawi G (2003) International multisite double-blind trial of the atypical antipsychotics risperidone and olanzapine in 175 elderly patients with chronic schizophrenia. Am J Geriatr Psychiatry 11: 638–647. 14609804 10.1176/appi.ajgp.11.6.638

[118] Kasper S, Lerman MN, McQuade RD, Saha A, Carson WH, et al (2003) Efficacy and safety of aripiprazole vs. haloperidol for long-term maintenance treatment following acute relapse of schizophrenia. Int J Neuropsychopharmacol 6: 325–337. 14609439 10.1017/S1461145703003651

[119] Keck PE Jr, Marcus R, Tourkodimitris S, Ali M, Liebeskind A, et al (2003) A placebo-controlled, double-blind study of the efficacy and safety of aripiprazole in patients with acute bipolar mania. Am J Psychiatry 160: 1651–1658. 12944341 10.1176/appi.ajp.160.9.1651

[120] Kelly DL, Conley RR, Richardson CM, Tamminga CA, Carpenter WT Jr (2003) Adverse effects and laboratory parameters of high-dose olanzapine vs. clozapine in treatment-resistant schizophrenia. Ann Clin Psychiatry 15: 181–186. 14971863 10.1023/b:acli.0000008171.90644.f8

[121] Kinon BJ, Hill AL, Liu H, Kollack-Walker S (2003) Olanzapine orally disintegrating tablets in the treatment of acutely ill non-compliant patients with schizophrenia. Int J Neuropsychopharmacol 6: 97–102. 12890301 10.1017/S1461145703003389

[122] Lane HY, Chang YC, Cheng YC, Liu GC, Lin XR, et al (2003) Effects of patient demographics, risperidone dosage, and clinical outcome on body weight in acutely exacerbated schizophrenia. J Clin Psychiatry 64: 316–320. 12716274 10.4088/jcp.v64n0314

[123] Marder SR, McQuade RD, Stock E, Kaplita S, Marcus R, et al (2003) Aripiprazole in the treatment of schizophrenia: safety and tolerability in short-term, placebo-controlled trials. Schizophr Res 61: 123–136. 12729864 10.1016/s0920-9964(03)00050-1

[124] McIntyre RS, Mancini DA, Basile VS, Srinivasan J, Kennedy SH (2003) Antipsychotic-induced weight gain: bipolar disorder and leptin. J Clin Psychopharmacol 23: 323–327. 12920406 10.1097/01.jcp.0000085403.08426.f4

[125] McIntyre RS, Trakas K, Lin D, Balshaw R, Hwang P, et al (2003) Risk of weight gain associated with antipsychotic treatment: results from the Canadian National Outcomes Measurement Study in Schizophrenia. Can J Psychiatry 48: 689–694. 14674052 10.1177/070674370304801008

[126] Pigott TA, Carson WH, Saha AR, Torbeyns AF, Stock EG, et al (2003) Aripiprazole for the prevention of relapse in stabilized patients with chronic schizophrenia: a placebo-controlled 26-week study. J Clin Psychiatry 64: 1048–1056. 14628980 10.4088/jcp.v64n0910

[127] Potkin SG, Saha AR, Kujawa MJ, Carson WH, Ali M, et al (2003) Aripiprazole, an antipsychotic with a novel mechanism of action, and risperidone vs placebo in patients with schizophrenia and schizoaffective disorder. Arch Gen Psychiatry 60: 681–690. 12860772 10.1001/archpsyc.60.7.681

[128] Ritchie CW, Chiu E, Harrigan S, Hall K, Hassett A, et al (2003) The impact upon extra-pyramidal side effects, clinical symptoms and quality of life of a switch from conventional to atypical antipsychotics (risperidone or olanzapine) in elderly patients with schizophrenia. Int J Geriatr Psychiatry 18: 432–440. 12766921 10.1002/gps.862

[129] Sanger TM, Tohen M, Vieta E, Dunner DL, Bowden CL, et al (2003) Olanzapine in the acute treatment of bipolar I disorder with a history of rapid cycling. J Affect Disord 73: 155–161. 12507748 10.1016/s0165-0327(02)00334-8

[130] Tohen M, Goldberg JF, Gonzalez-Pinto Arrillaga AM, Azorin JM, Vieta E, et al (2003) A 12-week, double-blind comparison of olanzapine vs haloperidol in the treatment of acute mania. Arch Gen Psychiatry 60: 1218–1226. 14662554 10.1001/archpsyc.60.12.1218

[131] van Bruggen J, Tijssen J, Dingemans P, Gersons B, Linszen D (2003) Symptom response and side-effects of olanzapine and risperidone in young adults with recent onset schizophrenia. Int Clin Psychopharmacol 18: 341–346. 14571154 10.1097/00004850-200311000-00005

[132] Weiden PJ, Simpson GM, Potkin SG, O'Sullivan RL (2003) Effectiveness of switching to ziprasidone for stable but symptomatic outpatients with schizophrenia. J Clin Psychiatry 64: 580–588. 12755663 10.4088/jcp.v64n0514

[133] Addington DE, Pantelis C, Dineen M, Benattia I, Romano SJ (2004) Efficacy and tolerability of ziprasidone versus risperidone in patients with acute exacerbation of schizophrenia or schizoaffective disorder: an 8-week, double-blind, multicenter trial. J Clin Psychiatry 65: 1624–1633. 15641867 10.4088/jcp.v65n1207

[134] Appelberg B, Tuisku K, Joffe G (2004) Is it worth while changing clinically stable schizophrenic out-patients with mild to moderate residual symptoms and/or side effects from conventional to atypical antipsychotics? A prospective, randomised study with olanzapine. Eur Psychiatry 19: 516–518. 15589715 10.1016/j.eurpsy.2004.06.035

[135] Barak Y, Shamir E, Mirecki I, Weizman R, Aizenberg D (2004) Switching elderly chronic psychotic patients to olanzapine. Int J Neuropsychopharmacol 7: 165–169. 14741062 10.1017/S1461145703004048

[136] Bitter I, Dossenbach MR, Brook S, Feldman PD, Metcalfe S, et al (2004) Olanzapine versus clozapine in treatment-resistant or treatment-intolerant schizophrenia. Prog Neuropsychopharmacol Biol Psychiatry 28: 173–180. 14687871 10.1016/j.pnpbp.2003.09.033

[137] Bogenschutz MP, George Nurnberg H (2004) Olanzapine versus placebo in the treatment of borderline personality disorder. J Clin Psychiatry 65: 104–109. 14744178 10.4088/jcp.v65n0118

[138] Buckley PF, Goldstein JM, Emsley RA (2004) Efficacy and tolerability of quetiapine in poorly responsive, chronic schizophrenia. Schizophr Res 66: 143–150. 15061246 10.1016/j.schres.2003.06.001

[139] Cohen SA, Fitzgerald BJ, Khan SR, Khan A (2004) The effect of a switch to ziprasidone in an adult population with autistic disorder: chart review of naturalistic, open-label treatment. J Clin Psychiatry 65: 110–113. 14744179 10.4088/jcp.v65n0119

[140] Covell NH, Weissman EM, Essock SM (2004) Weight gain with clozapine compared to first generation antipsychotic medications. Schizophr Bull 30: 229–240. 15279042 10.1093/oxfordjournals.schbul.a007074

[141] de Haan L, van Amelsvoort T, Rosien K, Linszen D (2004) Weight loss after switching from conventional olanzapine tablets to orally disintegrating olanzapine tablets. Psychopharmacology (Berl) 175: 389–390. 15322727 10.1007/s00213-004-1951-2

[142] De Deyn PP, Carrasco MM, Deberdt W, Jeandel C, Hay DP, et al (2004) Olanzapine versus placebo in the treatment of psychosis with or without associated behavioral disturbances in patients with Alzheimer's disease. Int J Geriatr Psychiatry 19: 115–126. 14758577 10.1002/gps.1032

[143] Emsley R, Turner HJ, Schronen J, Botha K, Smit R, et al (2004) A single-blind, randomized trial comparing quetiapine and haloperidol in the treatment of tardive dyskinesia. J Clin Psychiatry 65: 696–701. 15163258 10.4088/jcp.v65n0516

[144] Gupta S, Masand PS, Virk S, Schwartz T, Hameed A, et al (2004) Weight decline in patients switching from olanzapine to quetiapine. Schizophr Res 70: 57–62. 15246464 10.1016/j.schres.2003.09.016

[145] Hennen J, Perlis RH, Sachs G, Tohen M, Baldessarini RJ (2004) Weight gain during treatment of bipolar I patients with olanzapine. J Clin Psychiatry 65: 1679–1687. 15641874 10.4088/jcp.v65n1214

[146] Kinon BJ, Jeste DV, Kollack-Walker S, Stauffer V, Liu-Seifert H (2004) Olanzapine treatment for tardive dyskinesia in schizophrenia patients: a prospective clinical trial with patients randomized to blinded dose reduction periods. Prog Neuropsychopharmacol Biol Psychiatry 28: 985–996. 15380859 10.1016/j.pnpbp.2004.05.016

[147] Lane HY, Chang YC, Chiu CC, Lee SH, Lin CY, et al (2004) Fine-tuning risperidone dosage for acutely exacerbated schizophrenia: clinical determinants. Psychopharmacology (Berl) 172: 393–399. 14663551 10.1007/s00213-003-1685-6

[148] Lasser R, Bossie CA, Gharabawi G, Eerdekens M, Nasrallah HA (2004) Efficacy and safety of long-acting risperidone in stable patients with schizoaffective disorder. J Affect Disord 83: 263–275. 15555724 10.1016/j.jad.2004.05.008

[149] Lasser RA, Bossie CA, Gharabawi GM, Turner M (2004) Patients with schizophrenia previously stabilized on conventional depot antipsychotics experience significant clinical improvements following treatment with long-acting risperidone. Eur Psychiatry 19: 219–225. 15196604 10.1016/j.eurpsy.2003.11.007

[150] Lindenmayer JP, Eerdekens E, Berry SA, Eerdekens M (2004) Safety and efficacy of long-acting risperidone in schizophrenia: a 12-week, multicenter, open-label study in stable patients switched from typical and atypical oral antipsychotics. J Clin Psychiatry 65: 1084–1089. 15323593

[151] Moretti R, Torre P, Antonello RM, Cattaruzza T, Cazzato G, et al (2004) Olanzapine as a possible treatment for anxiety due to vascular dementia: an open study. Am J Alzheimers Dis Other Demen 19: 81–88. 15106388 10.1177/153331750401900215 PMC10833965

[152] McIntyre RS, Mancini DA, Srinivasan J, McCann S, Konarski JZ, et al (2004) The antidepressant effects of risperidone and olanzapine in bipolar disorder. Can J Clin Pharmacol 11: e218–226. 15520475

[153] McQuade RD, Stock E, Marcus R, Jody D, Gharbia NA, et al (2004) A comparison of weight change during treatment with olanzapine or aripiprazole: results from a randomized, double-blind study. J Clin Psychiatry 65 Suppl 18: 47–56. 15600384

[154] Reich DB, Winternitz S, Hennen J, Watts T, Stanculescu C (2004) A preliminary study of risperidone in the treatment of posttraumatic stress disorder related to childhood abuse in women. J Clin Psychiatry 65: 1601–1606. 15641864 10.4088/jcp.v65n1204

[155] Sacchetti E, Panariello A, Regini C, Valsecchi P (2004) Quetiapine in hospitalized patients with schizophrenia refractory to treatment with first-generation antipsychotics: a 4-week, flexible-dose, single-blind, exploratory, pilot trial. Schizophr Res 69: 325–331. 15469204 10.1016/s0920-9964(03)00225-1

[156] Simpson GM, Glick ID, Weiden PJ, Romano SJ, Siu CO (2004) Randomized, controlled, double-blind multicenter comparison of the efficacy and tolerability of ziprasidone and olanzapine in acutely ill inpatients with schizophrenia or schizoaffective disorder. Am J Psychiatry 161: 1837–1847. 15465981 10.1176/ajp.161.10.1837

[157] Vieta E, Brugue E, Goikolea JM, Sanchez-Moreno J, Reinares M, et al (2004) Acute and continuation risperidone monotherapy in mania. Hum Psychopharmacol 19: 41–45. 14716711 10.1002/hup.556

[158] Ascher-Svanum H, Stensland MD, Kinon BJ, Tollefson GD (2005) Weight gain as a prognostic indicator of therapeutic improvement during acute treatment of schizophrenia with placebo or active antipsychotic. J Psychopharmacol 19: 110–117. 16280344 10.1177/0269881105058978

[159] Ascher-Svanum H, Stensland M, Zhao Z, Kinon BJ (2005) Acute weight gain, gender, and therapeutic response to antipsychotics in the treatment of patients with schizophrenia. BMC Psychiatry 5: 3. 15649317 10.1186/1471-244X-5-3 PMC547901

[160] Buchanan RW, Ball MP, Weiner E, Kirkpatrick B, Gold JM, et al (2005) Olanzapine treatment of residual positive and negative symptoms. Am J Psychiatry 162: 124–129. 15625210 10.1176/appi.ajp.162.1.124

[161] Calabrese JR, Keck PE Jr, Macfadden W, Minkwitz M, Ketter TA, et al (2005) A randomized, double-blind, placebo-controlled trial of quetiapine in the treatment of bipolar I or II depression. Am J Psychiatry 162: 1351–1360. 15994719 10.1176/appi.ajp.162.7.1351

[162] Chue P, Eerdekens M, Augustyns I, Lachaux B, Molcan P, et al (2005) Comparative efficacy and safety of long-acting risperidone and risperidone oral tablets. Eur Neuropsychopharmacol 15: 111–117. 15572280 10.1016/j.euroneuro.2004.07.003

[163] Chue P (2005) Study of long-term quetiapine treatment. The journal of applied research 5: 246–252.

[164] De Deyn P, Jeste DV, Swanink R, Kostic D, Breder C, et al (2005) Aripiprazole for the treatment of psychosis in patients with Alzheimer's disease: a randomized, placebo-controlled study. J Clin Psychopharmacol 25: 463–467. 16160622 10.1097/01.jcp.0000178415.22309.8f

[165] Deberdt WG, Dysken MW, Rappaport SA, Feldman PD, Young CA, et al (2005) Comparison of olanzapine and risperidone in the treatment of psychosis and associated behavioral disturbances in patients with dementia. Am J Geriatr Psychiatry 13: 722–730. 16085789 10.1176/appi.ajgp.13.8.722

[166] Gastpar M, Masiak M, Latif MA, Frazzingaro S, Medori R, et al (2005) Sustained improvement of clinical outcome with risperidone long-acting injectable in psychotic patients previously treated with olanzapine. J Psychopharmacol 19: 32–38. 16144784 10.1177/0269881105056598

[167] Gasquet I, Haro JM, Novick D, Edgell ET, Kennedy L, et al (2005) Pharmacological treatment and other predictors of treatment outcomes in previously untreated patients with schizophrenia: results from the European Schizophrenia Outpatient Health Outcomes (SOHO) study. Int Clin Psychopharmacol 20: 199–205. 15933480 10.1097/00004850-200507000-00002

[168] Gomez-Esteban JC, Zarranz JJ, Velasco F, Lezcano E, Lachen MC, et al (2005) Use of ziprasidone in parkinsonian patients with psychosis. Clin Neuropharmacol 28: 111–114. 15965308 10.1097/01.wnf.0000164297.91643.ff

[169] Herrera-Estrella M, Apiquian R, Fresan A, Sanchez-Torres I (2005) The effects of amisulpride on five dimensions of psychopathology in patients with schizophrenia: a prospective open-label study. BMC Psychiatry 5: 22. 15869707 10.1186/1471-244X-5-22 PMC1090597

[170] Hollifield M, Thompson PM, Ruiz JE, Uhlenhuth EH (2005) Potential effectiveness and safety of olanzapine in refractory panic disorder. Depress Anxiety 21: 33–40. 15786486 10.1002/da.20050

[171] Kinon BJ, Kaiser CJ, Ahmed S, Rotelli MD, Kollack-Walker S (2005) Association between early and rapid weight gain and change in weight over one year of olanzapine therapy in patients with schizophrenia and related disorders. J Clin Psychopharmacol 25: 255–258. 15876905 10.1097/01.jcp.0000161501.65890.22

[172] Lambert M, Haro JM, Novick D, Edgell ET, Kennedy L, et al (2005) Olanzapine vs. other antipsychotics in actual out-patient settings: six months tolerability results from the European Schizophrenia Out-patient Health Outcomes study. Acta Psychiatr Scand 111: 232–243. 15701108 10.1111/j.1600-0447.2004.00451.x

[173] Lieberman JA, Stroup TS, McEvoy JP, Swartz MS, Rosenheck RA, et al (2005) Effectiveness of antipsychotic drugs in patients with chronic schizophrenia. N Engl J Med 353: 1209–1223. 16172203 10.1056/NEJMoa051688

[174] Mauri MC, Steinhilber CP, Marino R, Invernizzi E, Fiorentini A, et al (2005) Clinical outcome and olanzapine plasma levels in acute schizophrenia. Eur Psychiatry 20: 55–60. 15642445 10.1016/j.eurpsy.2004.09.009

[175] Hasnain M, Vieweg WV (2013) Weight considerations in psychotropic drug prescribing and switching. Postgrad Med 125: 117–129. 10.3810/pgm.2013.09.2706 24113670

[176] Mohl A, Westlye K, Opjordsmoen S, Lex A, Schreiner A, et al (2005) Long-acting risperidone in stable patients with schizoaffective disorder. J Psychopharmacol 19: 22–31. 16144783 10.1177/0269881105056515

[177] Moller HJ, Llorca PM, Sacchetti E, Martin SD, Medori R, et al (2005) Efficacy and safety of direct transition to risperidone long-acting injectable in patients treated with various antipsychotic therapies. Int Clin Psychopharmacol 20: 121–130. 15812261 10.1097/00004850-200505000-00001

[178] Montejo Gonzalez A, Rico-Villademoros F, Tafalla M, Majadas S (2005) A 6-month prospective observational study on the effects quatiapine on sexual functioning. Journal of Clinical Psychopharmacology 25: 533–538. 16282833 10.1097/01.jcp.0000186872.04984.56

[179] Murashita M, Kusumi I, Inoue T, Takahashi Y, Hosoda H, et al (2005) Olanzapine increases plasma ghrelin level in patients with schizophrenia. Psychoneuroendocrinology 30: 106–110. 15358448 10.1016/j.psyneuen.2004.05.008

[180] Naber D, Riedel M, Klimke A, Vorbach EU, Lambert M, et al (2005) Randomized double blind comparison of olanzapine vs. clozapine on subjective well-being and clinical outcome in patients with schizophrenia. Acta Psychiatr Scand 111: 106–115. 15667429 10.1111/j.1600-0447.2004.00486.x

[181] Nick B, Vauth R, Braendle D, Riecher-Rossler A (2005) Symptom control, functioning and satisfaction among Swiss patients treated with risperidone long-acting injectable. International Journal of Psychiatry and Clinical Practice 10: 174–181. 10.1080/13651500600633477 24941055

[182] Perry PJ, Argo TR, Carnahan RM, Lund BC, Holman TL, et al (2005) The association of weight gain and olanzapine plasma concentrations. J Clin Psychopharmacol 25: 250–254. 15876904 10.1097/01.jcp.0000162800.64378.82

[183] Theisen FM, Gebhardt S, Haberhausen M, Heinzel-Gutenbrunner M, Wehmeier PM, et al (2005) Clozapine-induced weight gain: a study in monozygotic twins and same-sex sib pairs. Psychiatr Genet 15: 285–289. 16314759 10.1097/00041444-200512000-00011

[184] Schooler N, Rabinowitz J, Davidson M, Emsley R, Harvey PD, et al (2005) Risperidone and haloperidol in first-episode psychosis: a long-term randomized trial. Am J Psychiatry 162: 947–953. 15863797 10.1176/appi.ajp.162.5.947

[185] Silva de Lima M, de Jesus Mari J, Breier A, Maria Costa A, Ponde de Sena E, et al (2005) Quality of life in schizophrenia: a multicenter, randomized, naturalistic, controlled trial comparing olanzapine to first-generation antipsychotics. J Clin Psychiatry 66: 831–838. 16013897

[186] Simpson GM, Weiden P, Pigott T, Murray S, Siu CO, et al (2005) Six-month, blinded, multicenter continuation study of ziprasidone versus olanzapine in schizophrenia. Am J Psychiatry 162: 1535–1538. 16055779 10.1176/appi.ajp.162.8.1535

[187] Smulevich AB, Khanna S, Eerdekens M, Karcher K, Kramer M, et al (2005) Acute and continuation risperidone monotherapy in bipolar mania: a 3-week placebo-controlled trial followed by a 9-week double-blind trial of risperidone and haloperidol. Eur Neuropsychopharmacol 15: 75–84. 15572276 10.1016/j.euroneuro.2004.06.003

[188] Soler J, Pascual JC, Campins J, Barrachina J, Puigdemont D, et al (2005) Double-blind, placebo-controlled study of dialectical behavior therapy plus olanzapine for borderline personality disorder. Am J Psychiatry 162: 1221–1224. 15930077 10.1176/appi.ajp.162.6.1221

[189] Villeneuve E, Lemelin S (2005) Open-label study of atypical neuroleptic quetiapine for treatment of borderline personality disorder: impulsivity as main target. J Clin Psychiatry 66: 1298–1303. 16259544 10.4088/jcp.v66n1013

[190] Zipursky RB, Gu H, Green AI, Perkins DO, Tohen MF, et al (2005) Course and predictors of weight gain in people with first-episode psychosis treated with olanzapine or haloperidol. Br J Psychiatry 187: 537–543. 16319406 10.1192/bjp.187.6.537

[191] Leelahanaj T, Kongsakon R, Netrakom P (2005) A 4-week, double-blind comparison of olanzapine with haloperidol in the treatment of amphetamine psychosis. J Med Assoc Thai 88 Suppl 3: S43–52. 16858942

[192] Alvarez E, Ciudad A, Olivares JM, Bousono M, Gomez JC (2006) A randomized, 1-year follow-up study of olanzapine and risperidone in the treatment of negative symptoms in outpatients with schizophrenia. J Clin Psychopharmacol 26: 238–249. 16702888 10.1097/01.jcp.0000222513.63767.de

[193] Azorin JM, Strub N, Loft H (2006) A double-blind, controlled study of sertindole versus risperidone in the treatment of moderate-to-severe schizophrenia. Int Clin Psychopharmacol 21: 49–56. 16317317 10.1097/01.yic.0000177020.26311.a7

[194] Chiu CC, Chen KP, Liu HC, Lu ML (2006) The early effect of olanzapine and risperidone on insulin secretion in atypical-naive schizophrenic patients. J Clin Psychopharmacol 26: 504–507. 16974193 10.1097/01.jcp.0000237947.80764.d9

[195] Christensen AF, Poulsen J, Nielsen CT, Bork B, Christensen A, et al (2006) Patients with schizophrenia treated with aripiprazole, a multicentre naturalistic study. Acta Psychiatr Scand 113: 148–153. 16423167 10.1111/j.1600-0447.2005.00736.x

[196] Chrzanowski WK, Marcus RN, Torbeyns A, Nyilas M, McQuade RD (2006) Effectiveness of long-term aripiprazole therapy in patients with acutely relapsing or chronic, stable schizophrenia: a 52-week, open-label comparison with olanzapine. Psychopharmacology (Berl) 189: 259–266. 17058105 10.1007/s00213-006-0564-3

[197] Ciudad A, Olivares JM, Bousono M, Gomez JC, Alvarez E (2006) Improvement in social functioning in outpatients with schizophrenia with prominent negative symptoms treated with olanzapine or risperidone in a 1 year randomized, open-label trial. Prog Neuropsychopharmacol Biol Psychiatry 30: 1515–1522. 16820255 10.1016/j.pnpbp.2006.05.010

[198] Hosojima H, Togo T, Odawara T, Hasegawa K, Miura S, et al (2006) Early effects of olanzapine on serum levels of ghrelin, adiponectin and leptin in patients with schizophrenia. J Psychopharmacol 20: 75–79. 16204328 10.1177/0269881105056647

[199] Kane JM, Khanna S, Rajadhyaksha S, Giller E (2006) Efficacy and tolerability of ziprasidone in patients with treatment-resistant schizophrenia. Int Clin Psychopharmacol 21: 21–28. 16317313 10.1097/01.yic.0000182114.65134.81

[200] Keefe RS, Seidman LJ, Christensen BK, Hamer RM, Sharma T, et al (2006) Long-term neurocognitive effects of olanzapine or low-dose haloperidol in first-episode psychosis. Biol Psychiatry 59: 97–105. 16140282 10.1016/j.biopsych.2005.06.022

[201] Kinon BJ, Lipkovich I, Edwards SB, Adams DH, Ascher-Svanum H, et al (2006) A 24-week randomized study of olanzapine versus ziprasidone in the treatment of schizophrenia or schizoaffective disorder in patients with prominent depressive symptoms. J Clin Psychopharmacol 26: 157–162. 16633144 10.1097/01.jcp.0000204137.82298.06

[202] Lecrubier Y, Quintin P, Bouhassira M, Perrin E, Lancrenon S (2006) The treatment of negative symptoms and deficit states of chronic schizophrenia: olanzapine compared to amisulpride and placebo in a 6-month double-blind controlled clinical trial. Acta Psychiatr Scand 114: 319–327. 17022791 10.1111/j.1600-0447.2006.00887.x

[203] Lee C, Wu KH, Habil H, Dyachkova Y, Lee P (2006) Treatment with olanzapine, risperidone or typical antipsychotic drugs in Asian patients with schizophrenia. Aust N Z J Psychiatry 40: 437–445. 16683970 10.1080/j.1440-1614.2006.01820.x

[204] Lipkovich I, Citrome L, Perlis R, Deberdt W, Houston JP, et al (2006) Early predictors of substantial weight gain in bipolar patients treated with olanzapine. J Clin Psychopharmacol 26: 316–320. 16702898 10.1097/01.jcp.0000219916.88810.1c

[205] McEvoy JP, Lieberman JA, Stroup TS, Davis SM, Meltzer HY, et al (2006) Effectiveness of clozapine versus olanzapine, quetiapine, and risperidone in patients with chronic schizophrenia who did not respond to prior atypical antipsychotic treatment. Am J Psychiatry 163: 600–610. 16585434 10.1176/ajp.2006.163.4.600

[206] McGlashan TH, Zipursky RB, Perkins D, Addington J, Miller T, et al (2006) Randomized, double-blind trial of olanzapine versus placebo in patients prodromally symptomatic for psychosis. Am J Psychiatry 163: 790–799. 16648318 10.1176/ajp.2006.163.5.790

[207] Olie JP, Spina E, Murray S, Yang R (2006) Ziprasidone and amisulpride effectively treat negative symptoms of schizophrenia: results of a 12-week, double-blind study. Int Clin Psychopharmacol 21: 143–151. 16528136 10.1097/01.yic.0000182121.59296.70

[208] Perlis RH, Baker RW, Zarate CA Jr, Brown EB, Schuh LM, et al (2006) Olanzapine versus risperidone in the treatment of manic or mixed States in bipolar I disorder: a randomized, double-blind trial. J Clin Psychiatry 67: 1747–1753. 17196055 10.4088/jcp.v67n1112

[209] Potkin SG, Gharabawi GM, Greenspan AJ, Mahmoud R, Kosik-Gonzalez C, et al (2006) A double-blind comparison of risperidone, quetiapine and placebo in patients with schizophrenia experiencing an acute exacerbation requiring hospitalization. Schizophr Res 85: 254–265. 16797162 10.1016/j.schres.2006.03.027

[210] Sachs G, Sanchez R, Marcus R, Stock E, McQuade R, et al (2006) Aripiprazole in the treatment of acute manic or mixed episodes in patients with bipolar I disorder: a 3-week placebo-controlled study. J Psychopharmacol 20: 536–546. 16401666 10.1177/0269881106059693

[211] Schneider LS, Tariot PN, Dagerman KS, Davis SM, Hsiao JK, et al (2006) Effectiveness of atypical antipsychotic drugs in patients with Alzheimer's disease. N Engl J Med 355: 1525–1538. 17035647 10.1056/NEJMoa061240

[212] Stroup TS, Lieberman JA, McEvoy JP, Swartz MS, Davis SM, et al (2006) Effectiveness of olanzapine, quetiapine, risperidone, and ziprasidone in patients with chronic schizophrenia following discontinuation of a previous atypical antipsychotic. Am J Psychiatry 163: 611–622. 16585435 10.1176/ajp.2006.163.4.611

[213] Strous RD, Kupchik M, Roitman S, Schwartz S, Gonen N, et al (2006) Comparison between risperidone, olanzapine, and clozapine in the management of chronic schizophrenia: a naturalistic prospective 12-week observational study. Hum Psychopharmacol 21: 235–243. 16783815 10.1002/hup.764

[214] Thase ME, Macfadden W, Weisler RH, Chang W, Paulsson B, et al (2006) Efficacy of quetiapine monotherapy in bipolar I and II depression: a double-blind, placebo-controlled study (the BOLDER II study). J Clin Psychopharmacol 26: 600–609. 17110817 10.1097/01.jcp.0000248603.76231.b7

[215] Tohen M, Calabrese JR, Sachs GS, Banov MD, Detke HC, et al (2006) Randomized, placebo-controlled trial of olanzapine as maintenance therapy in patients with bipolar I disorder responding to acute treatment with olanzapine. Am J Psychiatry 163: 247–256. 16449478 10.1176/appi.ajp.163.2.247

[216] Vanelle JM, Douki S (2006) A double-blind randomised comparative trial of amisulpride versus olanzapine for 2 months in the treatment of subjects with schizophrenia and comorbid depression. Eur Psychiatry 21: 523–530. 17113759 10.1016/j.eurpsy.2006.09.003

[217] Wang X, Savage R, Borisov A, Rosenberg J, Woolwine B, et al (2006) Efficacy of risperidone versus olanzapine in patients with schizophrenia previously on chronic conventional antipsychotic therapy: a switch study. J Psychiatr Res 40: 669–676. 16762371 10.1016/j.jpsychires.2006.03.008

[218] Zhong KX, Sweitzer DE, Hamer RM, Lieberman JA (2006) Comparison of quetiapine and risperidone in the treatment of schizophrenia: A randomized, double-blind, flexible-dose, 8-week study. J Clin Psychiatry 67: 1093–1103. 16889453 10.4088/jcp.v67n0712

[219] Arranz B, San L, Duenas RM, Centeno M, Ramirez N, et al (2007) Lower weight gain with the orally disintegrating olanzapine than with standard tablets in first-episode never treated psychotic patients. Hum Psychopharmacol 22: 11–15. 17191265 10.1002/hup.819

[220] Baptista T, Martinez M, Lacruz A, Arellano A, Mendoza S, et al (2007) Insulin resistance index and counter-regulatory factors during olanzapine or risperidone administration in subjects with schizophrenia. Schizophr Res 89: 350–352. 17029751 10.1016/j.schres.2006.08.020

[221] Baptista T, Davila A, El Fakih Y, Uzcategui E, Rangel NN, et al (2007) Similar frequency of abnormal correlation between serum leptin levels and BMI before and after olanzapine treatment in schizophrenia. Int Clin Psychopharmacol 22: 205–211. 17519643 10.1097/YIC.0b013e328080ca44

[222] Chan HY, Lin WW, Lin SK, Hwang TJ, Su TP, et al (2007) Efficacy and safety of aripiprazole in the acute treatment of schizophrenia in Chinese patients with risperidone as an active control: a randomized trial. J Clin Psychiatry 68: 29–36. 10.4088/jcp.v68n0104 17284127

[223] Davidson M, Emsley R, Kramer M, Ford L, Pan G, et al (2007) Efficacy, safety and early response of paliperidone extended-release tablets (paliperidone ER): results of a 6-week, randomized, placebo-controlled study. Schizophr Res 93: 117–130. 17466492 10.1016/j.schres.2007.03.003

[224] Dossenbach M, Treuer T, Kryzhanovskaya L, Saylan M, Dominguez S, et al (2007) Olanzapine versus chlorpromazine in the treatment of schizophrenia: a pooled analysis of four 6-week, randomized, open-label studies in the Middle East and North Africa. Journal of Clinical Psychopharmacology 27: 329–337. 17632215 10.1097/JCP.0b013e3180ca83b1

[225] Forsthoff A, Grunze H, Seemuller F, Stampfer R, Dittmann S, et al (2007) Risperidone monotherapy in manic inpatients: an open label, multicentre trial. World J Biol Psychiatry 8: 256–261. 17853251 10.1080/15622970601169766

[226] Gharabawi GM, Gearhart NC, Lasser RA, Mahmoud RA, Zhu Y, et al (2007) Maintenance therapy with once-monthly administration of long-acting injectable risperidone in patients with schizophrenia or schizoaffective disorder: a pilot study of an extended dosing interval. Ann Gen Psychiatry 6: 3. 17261186 10.1186/1744-859X-6-3 PMC1803785

[227] Han C, Lee MS, Pae CU, Ko YH, Patkar AA, et al (2007) Usefulness of long-acting injectable risperidone during 12-month maintenance therapy of bipolar disorder. Prog Neuropsychopharmacol Biol Psychiatry 31: 1219–1223. 17532106 10.1016/j.pnpbp.2007.04.017

[228] Haro JM, Suarez D, Novick D, Brown J, Usall J, et al (2007) Three-year antipsychotic effectiveness in the outpatient care of schizophrenia: observational versus randomized studies results. Eur Neuropsychopharmacol 17: 235–244. 17137759 10.1016/j.euroneuro.2006.09.005

[229] Kane J, Canas F, Kramer M, Ford L, Gassmann-Mayer C, et al (2007) Treatment of schizophrenia with paliperidone extended-release tablets: a 6-week placebo-controlled trial. Schizophr Res 90: 147–161. 17092691 10.1016/j.schres.2006.09.012

[230] Keck PE Jr, Calabrese JR, McIntyre RS, McQuade RD, Carson WH, et al (2007) Aripiprazole monotherapy for maintenance therapy in bipolar I disorder: a 100-week, double-blind study versus placebo. J Clin Psychiatry 68: 1480–1491. 17960961 10.4088/jcp.v68n1003

[231] Keks NA, Ingham M, Khan A, Karcher K (2007) Long-acting injectable risperidone v. olanzapine tablets for schizophrenia or schizoaffective disorder. Randomised, controlled, open-label study. Br J Psychiatry 191: 131–139. 17666497 10.1192/bjp.bp.105.017020

[232] Kerwin R, Millet B, Herman E, Banki CM, Lublin H, et al (2007) A multicentre, randomized, naturalistic, open-label study between aripiprazole and standard of care in the management of community-treated schizophrenic patients Schizophrenia Trial of Aripiprazole: (STAR) study. Eur Psychiatry 22: 433–443. 17555947 10.1016/j.eurpsy.2007.03.002

[233] Kim SW, Shin IS, Kim JM, Lee SH, Lee JH, et al (2007) Amisulpride versus risperidone in the treatment of depression in patients with schizophrenia: a randomized, open-label, controlled trial. Prog Neuropsychopharmacol Biol Psychiatry 31: 1504–1509. 17692448 10.1016/j.pnpbp.2007.07.005

[234] Kramer M, Simpson G, Maciulis V, Kushner S, Vijapurkar U, et al (2007) Paliperidone extended-release tablets for prevention of symptom recurrence in patients with schizophrenia: a randomized, double-blind, placebo-controlled study. J Clin Psychopharmacol 27: 6–14. 17224706 10.1097/JCP.0b013e31802dda4a

[235] Lindenmayer JP, Khan A, Eerdekens M, Van Hove I, Kushner S (2007) Long-term safety and tolerability of long-acting injectable risperidone in patients with schizophrenia or schizoaffective disorder. Eur Neuropsychopharmacol 17: 138–144. 17049818 10.1016/j.euroneuro.2006.08.004

[236] Lindenmayer JP, Khan A, Iskander A, Abad MT, Parker B (2007) A randomized controlled trial of olanzapine versus haloperidol in the treatment of primary negative symptoms and neurocognitive deficits in schizophrenia. J Clin Psychiatry 68: 368–379. 17388705 10.4088/jcp.v68n0303

[237] Loebel AD, Khanna S, Rajadhyaksha S, Siu CO, Giller E, et al (2007) Ziprasidone in treatment-resistant schizophrenia: a 52-week, open-label continuation study. J Clin Psychiatry 68: 1333–1338. 17915970 10.4088/jcp.v68n0902

[238] Marder SR, Kramer M, Ford L, Eerdekens E, Lim P, et al (2007) Efficacy and safety of paliperidone extended-release tablets: results of a 6-week, randomized, placebo-controlled study. Biol Psychiatry 62: 1363–1370. 17601495 10.1016/j.biopsych.2007.01.017

[239] Mauri MC, Colasanti A, Rossattini M, Volonteri LS, Dragogna F, et al (2007) Ziprasidone outcome and tolerability: a practical clinical trial with plasma drug levels. Pharmacopsychiatry 40: 89–92. 17541882 10.1055/s-2007-973835

[240] McEvoy JP, Daniel DG, Carson WH Jr, McQuade RD, Marcus RN (2007) A randomized, double-blind, placebo-controlled, study of the efficacy and safety of aripiprazole 10, 15 or 20 mg/day for the treatment of patients with acute exacerbations of schizophrenia. J Psychiatr Res 41: 895–905. 17631314 10.1016/j.jpsychires.2007.05.002

[241] McEvoy JP, Lieberman JA, Perkins DO, Hamer RM, Gu H, et al (2007) Efficacy and tolerability of olanzapine, quetiapine, and risperidone in the treatment of early psychosis: a randomized, double-blind 52-week comparison. Am J Psychiatry 164: 1050–1060. 17606657 10.1176/ajp.2007.164.7.1050

[242] Neovius M, Eberhard J, Lindstrom E, Levander S (2007) Weight development in patients treated with risperidone: a 5-year naturalistic study. Acta Psychiatr Scand 115: 277–285. 17355518 10.1111/j.1600-0447.2006.00899.x

[243] Perez-Iglesias R, Crespo-Facorro B, Amado JA, Garcia-Unzueta MT, Ramirez-Bonilla ML, et al (2007) A 12-week randomized clinical trial to evaluate metabolic changes in drug-naive, first-episode psychosis patients treated with haloperidol, olanzapine, or risperidone. J Clin Psychiatry 68: 1733–1740. 18052567 10.4088/jcp.v68n1113

[244] Peuskens J, De Hert M, Mortimer A (2007) Metabolic control in patients with schizophrenia treated with amisulpride or olanzapine. Int Clin Psychopharmacol 22: 145–152. 17414740 10.1097/YIC.0b013e3280148c29

[245] Popovic V, Doknic M, Maric N, Pekic S, Damjanovic A, et al (2007) Changes in neuroendocrine and metabolic hormones induced by atypical antipsychotics in normal-weight patients with schizophrenia. Neuroendocrinology 85: 249–256. 17570902 10.1159/000103868

[246] Potkin SG, Cohen M, Panagides J (2007) Efficacy and tolerability of asenapine in acute schizophrenia: a placebo- and risperidone-controlled trial. J Clin Psychiatry 68: 1492–1500. 17960962 10.4088/jcp.v68n1004

[247] Riedel M, Muller N, Spellmann I, Engel RR, Musil R, et al (2007) Efficacy of olanzapine versus quetiapine on cognitive dysfunctions in patients with an acute episode of schizophrenia. Eur Arch Psychiatry Clin Neurosci 257: 402–412. 17629725 10.1007/s00406-007-0748-9

[248] Ruhrmann S, Kissling W, Lesch OM, Schmauss M, Seemann U, et al (2007) Efficacy of flupentixol and risperidone in chronic schizophrenia with predominantly negative symptoms. Prog Neuropsychopharmacol Biol Psychiatry 31: 1012–1022. 17412473 10.1016/j.pnpbp.2007.02.014

[249] Strassnig M, Miewald J, Keshavan M, Ganguli R (2007) Weight gain in newly diagnosed first-episode psychosis patients and healthy comparisons: one-year analysis. Schizophr Res 93: 90–98. 17478082 10.1016/j.schres.2007.02.024 PMC2100420

[250] Stroup TS, Lieberman JA, McEvoy JP, Swartz MS, Davis SM, et al (2007) Effectiveness of olanzapine, quetiapine, and risperidone in patients with chronic schizophrenia after discontinuing perphenazine: a CATIE study. Am J Psychiatry 164: 415–427. 17329466 10.1176/ajp.2007.164.3.415

[251] Tamayo JM, Mazzotti G, Tohen M, Gattaz WF, Zapata R, et al (2007) Outcomes for Latin American versus White patients suffering from acute mania in a randomized, double-blind trial comparing olanzapine and haloperidol. J Clin Psychopharmacol 27: 126–134. 17414234 10.1097/JCP.0b013e318033bd4a

[252] Vieta E, Calabrese JR, Goikolea JM, Raines S, Macfadden W (2007) Quetiapine monotherapy in the treatment of patients with bipolar I or II depression and a rapid-cycling disease course: a randomized, double-blind, placebo-controlled study. Bipolar Disord 9: 413–425. 17547587 10.1111/j.1399-5618.2007.00479.x

[253] Zimbroff D, Warrington L, Loebel A, Yang R, Siu C (2007) Comparison of ziprasidone and aripiprazole in acutely ill patients with schizophrenia or schizoaffective disorder: a randomized, double-blind, 4-week study. Int Clin Psychopharmacol 22: 363–370. 17917555 10.1097/YIC.0b013e32816f7779

[254] Villarreal G, Calais LA, Canive JM, Lundy SL, Pickard J, et al (2007) Prospective study to evaluate the efficacy of aripiprazole as a monotherapy in patients with severe chronic posttraumatic stress disorder: an open trial. Psychopharmacol Bull 40: 6–18. 17514183

[255] Ader M, Garvey WT, Phillips LS, Nemeroff CB, Gharabawi G, et al (2008) Ethnic heterogeneity in glucoregulatory function during treatment with atypical antipsychotics in patients with schizophrenia. J Psychiatr Res 42: 1076–1085. 18295798 10.1016/j.jpsychires.2008.01.004 PMC3769976

[256] Blonde L, Kan HJ, Gutterman EM, L'Italien GJ, Kim MS, et al (2008) Predicted risk of diabetes and coronary heart disease in patients with schizophrenia: aripiprazole versus standard of care. J Clin Psychiatry 69: 741–748. 18435564 10.4088/jcp.v69n0507

[257] Canuso CM, Youssef EA, Bossie CA, Turkoz I, Schreiner A, et al (2008) Paliperidone extended-release tablets in schizophrenia patients previously treated with risperidone. Int Clin Psychopharmacol 23: 209–215. 18545059 10.1097/YIC.0b013e3282fce651

[258] Chawla B, Luxton-Andrew H (2008) Long-term weight loss observed with olanzapine orally disintegrating tablets in overweight patients with chronic schizophrenia. A 1 year open-label, prospective trial. Hum Psychopharmacol 23: 211–216. 18219624 10.1002/hup.921

[259] De Hert M, Schreurs V, Sweers K, Van Eyck D, Hanssens L, et al (2008) Typical and atypical antipsychotics differentially affect long-term incidence rates of the metabolic syndrome in first-episode patients with schizophrenia: a retrospective chart review. Schizophr Res 101: 295–303. 18299188 10.1016/j.schres.2008.01.028

[260] Emsley R, Berwaerts J, Eerdekens M, Kramer M, Lane R, et al (2008) Efficacy and safety of oral paliperidone extended-release tablets in the treatment of acute schizophrenia: pooled data from three 52-week open-label studies. Int Clin Psychopharmacol 23: 343–356. 18854723 10.1097/YIC.0b013e328314e1f3

[261] Emsley R, Medori R, Koen L, Oosthuizen PP, Niehaus DJ, et al (2008) Long-acting injectable risperidone in the treatment of subjects with recent-onset psychosis: a preliminary study. J Clin Psychopharmacol 28: 210–213. 18344732 10.1097/JCP.0b013e318167269d

[262] Faries DE, Ascher-Svanum H, Nyhuis AW, Kinon BJ (2008) Switching from risperidone to olanzapine in a one-year, randomized, open-label effectiveness study of schizophrenia. Curr Med Res Opin 24: 1399–1405. 18397549 10.1185/030079908x297385

[263] Graham KA, Cho H, Brownley KA, Harp JB (2008) Early treatment-related changes in diabetes and cardiovascular disease risk markers in first episode psychosis subjects. Schizophr Res 101: 287–294. 18255275 10.1016/j.schres.2007.12.476 PMC2443741

[264] Kelly DL, Conley RR, Love RC, Morrison JA, McMahon RP (2008) Metabolic risk with second-generation antipsychotic treatment: a double-blind randomized 8-week trial of risperidone and olanzapine. Ann Clin Psychiatry 20: 71–78. 18568578 10.1080/10401230802017050

[265] Kinon BJ, Stauffer VL, Kollack-Walker S, Chen L, Sniadecki J (2008) Olanzapine versus aripiprazole for the treatment of agitation in acutely ill patients with schizophrenia. J Clin Psychopharmacol 28: 601–607. 19011427 10.1097/JCP.0b013e31818aaf6c

[266] Kinon BJ, Volavka J, Stauffer V, Edwards SE, Liu-Seifert H, et al (2008) Standard and higher dose of olanzapine in patients with schizophrenia or schizoaffective disorder: a randomized, double-blind, fixed-dose study. J Clin Psychopharmacol 28: 392–400. 18626265 10.1097/JCP.0b013e31817e63a5

[267] Lauriello J, Lambert T, Andersen S, Lin D, Taylor CC, et al (2008) An 8-week, double-blind, randomized, placebo-controlled study of olanzapine long-acting injection in acutely ill patients with schizophrenia. J Clin Psychiatry 69: 790–799. 18452346 10.4088/jcp.v69n0512

[268] Loebl T, Angarita GA, Pachas GN, Huang KL, Lee SH, et al (2008) A randomized, double-blind, placebo-controlled trial of long-acting risperidone in cocaine-dependent men. J Clin Psychiatry 69: 480–486. 18294021 10.4088/jcp.v69n0321

[269] Malempati RN, Bond DJ, Yatham LN (2008) Depot risperidone in the outpatient management of bipolar disorder: a 2-year study of 10 patients. Int Clin Psychopharmacol 23: 88–94. 18301123 10.1097/YIC.0b013e3282f2b4c5

[270] McElroy SL, Nelson EB, Welge JA, Kaehler L, Keck PE Jr (2008) Olanzapine in the treatment of pathological gambling: a negative randomized placebo-controlled trial. J Clin Psychiatry 69: 433–440. 18251624 10.4088/jcp.v69n0314

[271] Meltzer HY, Bobo WV, Roy A, Jayathilake K, Chen Y, et al (2008) A randomized, double-blind comparison of clozapine and high-dose olanzapine in treatment-resistant patients with schizophrenia. J Clin Psychiatry 69: 274–285. 18232726 10.4088/jcp.v69n0214

[272] Meltzer HY, Bobo WV, Nuamah IF, Lane R, Hough D, et al (2008) Efficacy and tolerability of oral paliperidone extended-release tablets in the treatment of acute schizophrenia: pooled data from three 6-week, placebo-controlled studies. J Clin Psychiatry 69: 817–829. 18466043 10.4088/jcp.v69n0515

[273] Moeller H-J, Johnson S, Mateva T, Brecher M, Svensson O, et al (2008) Evaluation of the feasibility of switching from immidiate release quetiapine to extended release quetiapine fumarate in stable outpatients with schizophrenia. Journal of Clinical Psychopharmacology 23: 95–105. 10.1097/YIC.0b013e3282f2d42c 18301124

[274] Muzina DJ, Momah C, Eudicone JM, Pikalov A, McQuade RD, et al (2008) Aripiprazole monotherapy in patients with rapid-cycling bipolar I disorder: an analysis from a long-term, double-blind, placebo-controlled study. Int J Clin Pract 62: 679–687. 18373615 10.1111/j.1742-1241.2008.01735.x PMC2324208

[275] Newcomer JW, Campos JA, Marcus RN, Breder C, Berman RM, et al (2008) A multicenter, randomized, double-blind study of the effects of aripiprazole in overweight subjects with schizophrenia or schizoaffective disorder switched from olanzapine. J Clin Psychiatry 69: 1046–1056. 18605811 10.4088/jcp.v69n0702

[276] Roerig JL, Steffen KJ, Mitchell JE, Crosby RD, Gosnell BA (2008) A comparison of the effects of olanzapine and risperidone versus placebo on ghrelin plasma levels. J Clin Psychopharmacol 28: 21–26. 18204336 10.1097/jcp.0b013e3181613325

[277] Sacchetti E, Valsecchi P, Parrinello G (2008) A randomized, flexible-dose, quasi-naturalistic comparison of quetiapine, risperidone, and olanzapine in the short-term treatment of schizophrenia: the QUERISOLA trial. Schizophr Res 98: 55–65. 17933497 10.1016/j.schres.2007.09.011

[278] Saddichha S, Manjunatha N, Ameen S, Akhtar S (2008) Diabetes and schizophrenia - effect of disease or drug? Results from a randomized, double-blind, controlled prospective study in first-episode schizophrenia. Acta Psychiatr Scand 117: 342–347. 18307585 10.1111/j.1600-0447.2008.01158.x

[279] Sajatovic M, Calabrese JR, Mullen J (2008) Quetiapine for the treatment of bipolar mania in older adults. Bipolar Disord 10: 662–671. 18837860 10.1111/j.1399-5618.2008.00614.x

[280] Sajatovic M, Coconcea N, Ignacio RV, Blow FC, Hays RW, et al (2008) Aripiprazole therapy in 20 older adults with bipolar disorder: a 12-week, open-label trial. J Clin Psychiatry 69: 41–46. 18312036 10.4088/jcp.v69n0106

[281] Schulz SC, Zanarini MC, Bateman A, Bohus M, Detke HC, et al (2008) Olanzapine for the treatment of borderline personality disorder: variable dose 12-week randomised double-blind placebo-controlled study. Br J Psychiatry 193: 485–492. 19043153 10.1192/bjp.bp.107.037903

[282] Smith E, Rothschild AJ, Heo M, Peasley-Miklus C, Caswell M, et al (2008) Weight gain during olanzapine treatment for psychotic depression: effects of dose and age. Int Clin Psychopharmacol 23: 130–137. 18408527 10.1097/YIC.0b013e3282f424d6

[283] Streim JE, Porsteinsson AP, Breder CD, Swanink R, Marcus R, et al (2008) A randomized, double-blind, placebo-controlled study of aripiprazole for the treatment of psychosis in nursing home patients with Alzheimer disease. Am J Geriatr Psychiatry 16: 537–550. 18591574 10.1097/JGP.0b013e318165db77

[284] Thase ME, Jonas A, Khan A, Bowden CL, Wu X, et al (2008) Aripiprazole monotherapy in nonpsychotic bipolar I depression: results of 2 randomized, placebo-controlled studies. J Clin Psychopharmacol 28: 13–20. 18204335 10.1097/jcp.0b013e3181618eb4

[285] van Winkel R, De Hert M, Van Eyck D, Hanssens L, Wampers M, et al (2008) Prevalence of diabetes and the metabolic syndrome in a sample of patients with bipolar disorder. Bipolar Disord 10: 342–348. 18271914 10.1111/j.1399-5618.2007.00520.x

[286] Weiden PJ, Cutler AJ, Polymeropoulos MH, Wolfgang CD (2008) Safety profile of iloperidone: a pooled analysis of 6-week acute-phase pivotal trials. J Clin Psychopharmacol 28: S12–19. 18334908 10.1097/JCP.0b013e3181694f5a

[287] Addington DE, Labelle A, Kulkarni J, Johnson G, Loebel A, et al (2009) A comparison of ziprasidone and risperidone in the long-term treatment of schizophrenia: a 44-week, double-blind, continuation study. Can J Psychiatry 54: 46–54. 19175979 10.1177/070674370905400108

[288] Alptekin K, Hafez J, Brook S, Akkaya C, Tzebelikos E, et al (2009) Efficacy and tolerability of switching to ziprasidone from olanzapine, risperidone or haloperidol: an international, multicenter study. Int Clin Psychopharmacol 24: 229–238. 19531959 10.1097/YIC.0b013e32832c2624

[289] Fleischhacker WW, McQuade RD, Marcus RN, Archibald D, Swanink R, et al (2009) A double-blind, randomized comparative study of aripiprazole and olanzapine in patients with schizophrenia. Biol Psychiatry 65: 510–517. 18986646 10.1016/j.biopsych.2008.07.033

[290] Haro JM, Novick D, Suarez D, Roca M (2009) Antipsychotic treatment discontinuation in previously untreated patients with schizophrenia: 36-month results from the SOHO study. J Psychiatr Res 43: 265–273. 18644606 10.1016/j.jpsychires.2008.06.001

[291] Hatta K, Sato K, Hamakawa H, Takebayashi H, Kimura N, et al (2009) Effectiveness of second-generation antipsychotics with acute-phase schizophrenia. Schizophr Res 113: 49–55. 19553086 10.1016/j.schres.2009.05.030

[292] Hori H, Ueda N, Yoshimura R, Yamamoto H, Wani K, et al (2009) Olanzapine orally disintegrating tablets (Zyprexa Zydis) rapidly improve excitement components in the acute phase of first-episode schizophrenic patients: an open-label prospective study. World J Biol Psychiatry 10: 741–745. 19707954 10.1080/15622970903166312

[293] Ingole S, Belorkar NR, Waradkar P, Shrivastava M (2009) Comparison of effects of olanzapine and risperidone on body mass index and blood sugar level in schizophrenic patients. Indian J Physiol Pharmacol 53: 47–54. 19810576

[294] Kane JM, Osuntokun O, Kryzhanovskaya LA, Xu W, Stauffer VL, et al (2009) A 28-week, randomized, double-blind study of olanzapine versus aripiprazole in the treatment of schizophrenia. J Clin Psychiatry 70: 572–581. 19323965 10.4088/jcp.08m04421

[295] Karagianis J, Grossman L, Landry J, Reed VA, de Haan L, et al (2009) A randomized controlled trial of the effect of sublingual orally disintegrating olanzapine versus oral olanzapine on body mass index: the PLATYPUS Study. Schizophr Res 113: 41–48. 19535229 10.1016/j.schres.2009.05.024

[296] Karagianis J, Williams R, Davis L, Procyshyn R, Monga N, et al (2009) Antipsychotic switching: results from a one-year prospective, observational study of patients with schizophrenia. Curr Med Res Opin 25: 2121–2132. 19601707 10.1185/03007990903102966

[297] Keck PE, Orsulak PJ, Cutler AJ, Sanchez R, Torbeyns A, et al (2009) Aripiprazole monotherapy in the treatment of acute bipolar I mania: a randomized, double-blind, placebo- and lithium-controlled study. J Affect Disord 112: 36–49. 18835043 10.1016/j.jad.2008.05.014

[298] Kim SW, Shin IS, Kim JM, Lee SH, Lee YH, et al (2009) Effects of switching to long-acting injectable risperidone from oral atypical antipsychotics on cognitive function in patients with schizophrenia. Hum Psychopharmacol 24: 565–573. 19790174 10.1002/hup.1057

[299] Kluge M, Schuld A, Schacht A, Himmerich H, Dalal MA, et al (2009) Effects of clozapine and olanzapine on cytokine systems are closely linked to weight gain and drug-induced fever. Psychoneuroendocrinology 34: 118–128. 18835660 10.1016/j.psyneuen.2008.08.016

[300] Krakowski M, Czobor P, Citrome L (2009) Weight gain, metabolic parameters, and the impact of race in aggressive inpatients randomized to double-blind clozapine, olanzapine or haloperidol. Schizophr Res 110: 95–102. 19269139 10.1016/j.schres.2009.02.006

[301] Lee KU, Jeon YW, Lee HK, Jun TY (2009) Efficacy and safety of quetiapine for depressive symptoms in patients with schizophrenia. Hum Psychopharmacol 24: 447–452. 19606454 10.1002/hup.1047

[302] McIntyre RS, Cohen M, Zhao J, Alphs L, Macek TA, et al (2009) A 3-week, randomized, placebo-controlled trial of asenapine in the treatment of acute mania in bipolar mania and mixed states. Bipolar Disord 11: 673–686. 19839993 10.1111/j.1399-5618.2009.00748.x

[303] Meyer JM, Rosenblatt LC, Kim E, Baker RA, Whitehead R (2009) The moderating impact of ethnicity on metabolic outcomes during treatment with olanzapine and aripiprazole in patients with schizophrenia. J Clin Psychiatry 70: 318–325. 19192469 10.4088/jcp.08m04267

[304] Nakamura M, Ogasa M, Guarino J, Phillips D, Severs J, et al (2009) Lurasidone in the treatment of acute schizophrenia: a double-blind, placebo-controlled trial. J Clin Psychiatry 70: 829–836. 19497249 10.4088/JCP.08m04905

[305] Newcomer JW, Ratner RE, Eriksson JW, Emsley R, Meulien D, et al (2009) A 24-week, multicenter, open-label, randomized study to compare changes in glucose metabolism in patients with schizophrenia receiving treatment with olanzapine, quetiapine, or risperidone. J Clin Psychiatry 70: 487–499. 19358783 10.4088/jcp.08m04132 PMC3703648

[306] Pae CU, Serretti A, Chiesa A, Mandelli L, Lee C, et al (2009) Immediate versus gradual suspension of previous treatments during switch to aripiprazole: results of a randomized, open label study. Eur Neuropsychopharmacol 19: 562–570. 19442491 10.1016/j.euroneuro.2009.04.002

[307] Peuskens J, Gillain B, De Graeve D, Van Vleymen B, Albert A (2009) Belgian Schizophrenia Outcome Survey - results of a 2-year naturalistic study in patients stabilised on monotherapy with olanzapine, risperidone or haloperidol. Eur Psychiatry 24: 154–163. 19118983 10.1016/j.eurpsy.2008.11.002

[308] Ryckmans V, Kahn JP, Modell S, Werner C, McQuade RD, et al (2009) Switching to aripiprazole in outpatients with schizophrenia experiencing insufficient efficacy and/or safety/tolerability issues with risperidone: a randomized, multicentre, open-label study. Pharmacopsychiatry 42: 114–121. 19452380 10.1055/s-0028-1112134

[309] Rossi A, Bagala A, Del Curatolo V, Scapati F, Bernareggi MM, et al (2009) Remission in schizophrenia: one-year Italian prospective study of risperidone long-acting injectable (RLAI) in patients with schizophrenia or schizoaffective disorder. Hum Psychopharmacol 24: 574–583. 19790173 10.1002/hup.1067

[310] Sacchetti E, Galluzzo A, Valsecchi P, Romeo F, Gorini B, et al (2009) Ziprasidone vs clozapine in schizophrenia patients refractory to multiple antipsychotic treatments: the MOZART study. Schizophr Res 113: 112–121. 19606529 10.1016/j.schres.2009.05.002

[311] Sheehan DV, McElroy SL, Harnett-Sheehan K, Keck PE Jr, Janavs J, et al (2009) Randomized, placebo-controlled trial of risperidone for acute treatment of bipolar anxiety. J Affect Disord 115: 376–385. 19042026 10.1016/j.jad.2008.10.005

[312] Smith RC, Lindenmayer JP, Davis JM, Kelly E, Viviano TF, et al (2009) Effects of olanzapine and risperidone on glucose metabolism and insulin sensitivity in chronic schizophrenic patients with long-term antipsychotic treatment: a randomized 5-month study. J Clin Psychiatry 70: 1501–1513. 19814947 10.4088/JCP.08m04446yel

[313] Stroup TS, Lieberman JA, McEvoy JP, Davis SM, Swartz MS, et al (2009) Results of phase 3 of the CATIE schizophrenia trial. Schizophr Res 107: 1–12. 19027269 10.1016/j.schres.2008.10.011 PMC2675163

[314] Takahashi H, Oshimo T, Ishigooka J (2009) Efficacy and tolerability of aripiprazole in first-episode drug-naive patients with schizophrenia: an open-label trial. Clin Neuropharmacol 32: 149–150. 19483480 10.1097/WNF.0b013e31817c6b06

[315] Treuer T, Hoffmann VP, Chen AK, Irimia V, Ocampo M, et al (2009) Factors associated with weight gain during olanzapine treatment in patients with schizophrenia or bipolar disorder: results from a six-month prospective, multinational, observational study. World J Biol Psychiatry 10: 729–740. 19606406 10.1080/15622970903079507

[316] Weisler R, Joyce M, McGill L, Lazarus A, Szamosi J, et al (2009) Extended release quetiapine fumarate monotherapy for major depressive disorder: results of a double-blind, randomized, placebo-controlled study. CNS Spectr 14: 299–313. 19668121 10.1017/s1092852900020307

[317] Young AH, Oren DA, Lowy A, McQuade RD, Marcus RN, et al (2009) Aripiprazole monotherapy in acute mania: 12-week randomised placebo- and haloperidol-controlled study. Br J Psychiatry 194: 40–48. 19118324 10.1192/bjp.bp.108.049965

[318] Bhowmick S, Hazra A, Ghosh M (2010) Amisulpride versus olanzapine in the treatment of schizophrenia in Indian patients: randomized controlled trial. Aust N Z J Psychiatry 44: 237–242. 20050717 10.3109/00048670903487134

[319] Bitter I, Treuer T, Dilbaz N, Oyffe I, Ciorabai EM, et al (2010) Patients' preference for olanzapine orodispersible tablet compared with conventional oral tablet in a multinational, randomized, crossover study. World J Biol Psychiatry 11: 894–903. 20653494 10.3109/15622975.2010.505663 PMC2981076

[320] Bobo WV, Epstein RA, Shelton RC (2010) Olanzapine monotherapy for acute depression in patients with bipolar I or II disorder: results of an 8-week open label trial. Hum Psychopharmacol 25: 30–36. 20029794 10.1002/hup.1082

[321] Bortnick B, El-Khalili N, Banov M, Adson D, Datto C, et al (2011) Efficacy and tolerability of extended release quetiapine fumarate (quetiapine XR) monotherapy in major depressive disorder: a placebo-controlled, randomized study. J Affect Disord 128: 83–94. 20691481 10.1016/j.jad.2010.06.031

[322] Bushe C, Sniadecki J, Bradley AJ, Poole Hoffmann V (2010) Comparison of metabolic and prolactin variables from a six-month randomised trial of olanzapine and quetiapine in schizophrenia. J Psychopharmacol 24: 1001–1009. 19240085 10.1177/0269881108101783

[323] Canuso CM, Grinspan A, Kalali A, Damaraju CV, Merriman U, et al (2010) Medication satisfaction in schizophrenia: a blinded-initiation study of paliperidone extended release in patients suboptimally responsive to risperidone. Int Clin Psychopharmacol 25: 155–164. 20216424 10.1097/YIC.0b013e3283372977

[324] Chiu CC, Chen CH, Chen BY, Yu SH, Lu ML (2010) The time-dependent change of insulin secretion in schizophrenic patients treated with olanzapine. Prog Neuropsychopharmacol Biol Psychiatry 34: 866–870. 20394794 10.1016/j.pnpbp.2010.04.003

[325] van Dooren FE, Nefs G, Schram MT, Verhey FR, Denollet J, et al (2013) Depression and risk of mortality in people with diabetes mellitus: a systematic review and meta-analysis. PLoS One 8: e57058. 23472075 10.1371/journal.pone.0057058 PMC3589463

[326] Hsieh MH, Lin WW, Chen ST, Chen KC, Chen KP, et al (2010) A 64-week, multicenter, open-label study of aripiprazole effectiveness in the management of patients with schizophrenia or schizoaffective disorder in a general psychiatric outpatient setting. Ann Gen Psychiatry 9: 35. 20849577 10.1186/1744-859X-9-35 PMC2949853

[327] Kane JM, Detke HC, Naber D, Sethuraman G, Lin DY, et al (2010) Olanzapine long-acting injection: a 24-week, randomized, double-blind trial of maintenance treatment in patients with schizophrenia. Am J Psychiatry 167: 181–189. 20008947 10.1176/appi.ajp.2009.07081221

[328] Kemp DE, Calabrese JR, Tran QV, Pikalov A, Eudicone JM, et al (2010) Metabolic syndrome in patients enrolled in a clinical trial of aripiprazole in the maintenance treatment of bipolar I disorder: a post hoc analysis of a randomized, double-blind, placebo-controlled trial. J Clin Psychiatry 71: 1138–1144. 20492838 10.4088/JCP.09m05159gre PMC3590811

[329] Kim SW, Shin IS, Kim JM, Bae KY, Yang SJ, et al (2010) Effectiveness of switching from aripiprazole to ziprasidone in patients with schizophrenia. Clin Neuropharmacol 33: 121–125. 20502130 10.1097/WNF.0b013e3181d52b85

[330] Daly EJ, Kent JM, Janssens L, Newcomer JW, Husken G, et al (2013) Metabolic and body mass parameters after treatment with JNJ-37822681, a novel fast-dissociating D2 receptor antagonist, vs olanzapine in patients with schizophrenia. Ann Clin Psychiatry 25: 173–183. 23926573

[331] McIntyre RS, Cohen M, Zhao J, Alphs L, Macek TA, et al (2010) Asenapine for long-term treatment of bipolar disorder: a double-blind 40-week extension study. J Affect Disord 126: 358–365. 20537396 10.1016/j.jad.2010.04.005

[332] McIntyre RS, Cohen M, Zhao J, Alphs L, Macek TA, et al (2010) Asenapine in the treatment of acute mania in bipolar I disorder: a randomized, double-blind, placebo-controlled trial. J Affect Disord 122: 27–38. 20096936 10.1016/j.jad.2009.12.028

[333] Meltzer HY, Bobo WV, Lee MA, Cola P, Jayathilake K (2010) A randomized trial comparing clozapine and typical neuroleptic drugs in non-treatment-resistant schizophrenia. Psychiatry Res 177: 286–293. 20378185 10.1016/j.psychres.2010.02.018

[334] Parellada E, Kouniakis F, Siurkute A, Schreiner A, Don L (2010) Safety and efficacy of long-acting injectable risperidone in daily practice: an open-label, noninterventional, prospective study in schizophrenia and related disorders. Int Clin Psychopharmacol 25: 149–154. 20305567 10.1097/YIC.0b013e328336c93f

[335] Quiroz JA, Yatham LN, Palumbo JM, Karcher K, Kushner S, et al (2010) Risperidone long-acting injectable monotherapy in the maintenance treatment of bipolar I disorder. Biol Psychiatry 68: 156–162. 20227682 10.1016/j.biopsych.2010.01.015

[336] Schoemaker J, Naber D, Vrijland P, Panagides J, Emsley R (2010) Long-term assessment of Asenapine vs. Olanzapine in patients with schizophrenia or schizoaffective disorder. Pharmacopsychiatry 43: 138–146. 20205074 10.1055/s-0030-1248313

[337] Suppes T, Datto C, Minkwitz M, Nordenhem A, Walker C, et al (2010) Effectiveness of the extended release formulation of quetiapine as monotherapy for the treatment of acute bipolar depression. J Affect Disord 121: 106–115. 19903574 10.1016/j.jad.2009.10.007

[338] Van Ameringen M, Mancini C, Patterson B, Bennett M, Oakman J (2010) A randomized, double-blind, placebo-controlled trial of olanzapine in the treatment of trichotillomania. J Clin Psychiatry 71: 1336–1343. 20441724 10.4088/JCP.09m05114gre

[339] Bobo WV, Bonaccorso S, Jayathilake K, Meltzer HY (2011) Prediction of long-term metabolic effects of olanzapine and risperidone treatment from baseline body mass index in schizophrenia and bipolar disorder. Psychiatry Res 189: 200–207. 21802150 10.1016/j.psychres.2011.07.008

[340] Bobo WV, Epstein RA Jr, Shelton RC (2011) Effects of orally disintegrating vs regular olanzapine tablets on body weight, eating behavior, glycemic and lipid indices, and gastrointestinal hormones: a randomized, open comparison in outpatients with bipolar depression. Ann Clin Psychiatry 23: 193–201. 21808751

[341] Chen CH, Lin TY, Chen TT, Chen VC, Lin NC, et al (2011) A prospective study of glucose homeostasis in quetiapine-treated schizophrenic patients by using the intravenous glucose tolerance test. Prog Neuropsychopharmacol Biol Psychiatry 35: 965–969. 21291941 10.1016/j.pnpbp.2011.01.015

[342] Gaebel W, Schreiner A, Bergmans P, de Arce R, Rouillon F, et al (2011) Relapse prevention in schizophrenia and schizoaffective disorder with ripseridone long-acting injectable vs quetiapine: Results of a long-term open label randomized clinical trial. Neuropsychopharmacology 25: 2367–2377. 10.1038/npp.2010.111 PMC3055334 20686456

[343] Gopal S, Vijapurkar U, Lim P, Morozova M, Eerdekens M, et al (2011) A 52-week open-label study of the safety and tolerability of paliperidone palmitate in patients with schizophrenia. J Psychopharmacol 25: 685–697. 20615933 10.1177/0269881110372817

[344] Hardy TA, Henry RR, Forrester TD, Kryzhanovskaya LA, Campbell GM, et al (2011) Impact of olanzapine or risperidone treatment on insulin sensitivity in schizophrenia or schizoaffective disorder. Diabetes Obes Metab 13: 726–735. 21435142 10.1111/j.1463-1326.2011.01398.x

[345] Hill AL, Sun B, Karagianis JL, Watson SB, McDonnell DP (2011) Dose-associated changes in safety and efficacy parameters observed in a 24-week maintenance trial of olanzapine long-acting injection in patients with schizophrenia. BMC Psychiatry 11: 28. 21324135 10.1186/1471-244X-11-28 PMC3048520

[346] Kane JM, Mackle M, Snow-Adami L, Zhao J, Szegedi A, et al (2011) A randomized placebo-controlled trial of asenapine for the prevention of relapse of schizophrenia after long-term treatment. J Clin Psychiatry 72: 349–355. 21367356 10.4088/JCP.10m06306

[347] Kane JM, Potkin SG, Daniel DG, Buckley PF (2011) A double-blind, randomized study comparing the efficacy and safety of sertindole and risperidone in patients with treatment-resistant schizophrenia. J Clin Psychiatry 72: 194–204. 20673553 10.4088/JCP.07m03733yel

[348] Karayal ON, Glue P, Bachinsky M, Stewart M, Chappell P, et al (2011) Switching from quetiapine to ziprasidone: a sixteen-week, open-label, multicenter study evaluating the effectiveness and safety of ziprasidone in outpatient subjects with schizophrenia or schizoaffective disorder. J Psychiatr Pract 17: 100–109. 21430488 10.1097/01.pra.0000396061.05269.c8

[349] Lee JS, Ahn JH, Lee JI, Kim JH, Jung I, et al (2011) Dose pattern and effectiveness of paliperidone extended-release tablets in patients with schizophrenia. Clin Neuropharmacol 34: 186–190. 21725234 10.1097/WNF.0b013e3182281c05

[350] Lindenmayer JP, Citrome L, Khan A, Kaushik S (2011) A randomized, double-blind, parallel-group, fixed-dose, clinical trial of quetiapine at 600 versus 1200 mg/d for patients with treatment-resistant schizophrenia or schizoaffective disorder. J Clin Psychopharmacol 31: 160–168. 21346616 10.1097/JCP.0b013e31820f4fe0

[351] McDonnell DP, Kryzhanovskaya LA, Zhao F, Detke HC, Feldman PD (2011) Comparison of metabolic changes in patients with schizophrenia during randomized treatment with intramuscular olanzapine long-acting injection versus oral olanzapine. Hum Psychopharmacol 10.1002/hup.1225 21823172

[352] Ozguven HD, Baskak B, Oner O, Atbasoglu C (2011) Metabolic effects of olanzapine and quetiapine: a six week randomized, single blind, controlled study. 2011 4: 10–17.

[353] Pandina G, Lane R, Gopal S, Gassmann-Mayer C, Hough D, et al (2011) A double-blind study of paliperidone palmitate and risperidone long-acting injectable in adults with schizophrenia. Prog Neuropsychopharmacol Biol Psychiatry 35: 218–226. 21092748 10.1016/j.pnpbp.2010.11.008

[354] Rodrigues Louza M, Elkis H, Ruschel S, Reis de Oliveira I, Affonseca Bressan R, et al (2011) Long-acting injectable risperidone in partially adherent and non-adherent patients with schizophrenia. Neuropsychiatric Disease and Treatment 7: 391–398. 21822391 10.2147/NDT.S20589 PMC3148931

[355] Sevy S, Robinson DG, Sunday S, Napolitano B, Miller R, et al (2011) Olanzapine vs. risperidone in patients with first-episode schizophrenia and a lifetime history of cannabis use disorders: 16-week clinical and substance use outcomes. Psychiatry Res 188: 310–314. 21636134 10.1016/j.psychres.2011.05.001 PMC3146636

[356] Stroup TS, McEvoy JP, Ring KD, Hamer RH, LaVange LM, et al (2011) A randomized trial examining the effectiveness of switching from olanzapine, quetiapine, or risperidone to aripiprazole to reduce metabolic risk: comparison of antipsychotics for metabolic problems (CAMP). Am J Psychiatry 168: 947–956. 21768610 10.1176/appi.ajp.2011.10111609 PMC3773729

[357] White MP, Koran LM (2011) Open-label trial of aripiprazole in the treatment of trichotillomania. J Clin Psychopharmacol 31: 503–506. 21694623 10.1097/JCP.0b013e318221b1ba

[358] Xiang YT, Wang CY, Ungvari GS, Kreyenbuhl JA, Chiu HF, et al (2011) Weight changes and their associations with demographic and clinical characteristics in risperidone maintenance treatment for schizophrenia. Pharmacopsychiatry 44: 135–141. 21710403 10.1055/s-0031-1277178

[359] Zanarini MC, Schulz SC, Detke HC, Tanaka Y, Zhao F, et al (2011) A dose comparison of olanzapine for the treatment of borderline personality disorder: a 12-week randomized, double-blind, placebo-controlled study. J Clin Psychiatry 72: 1353–1362. 21535995 10.4088/JCP.08m04138yel

