# Supplementary figures and images for: Almost All Antipsychotics Result in Weight Gain: A Meta-Analysis
Source: PLoS One. 2014 Apr 24;9(4):e94112. doi: 10.1371/journal.pone.0094112 (PMC3998960; doi:10.1371/journal.pone.0094112)

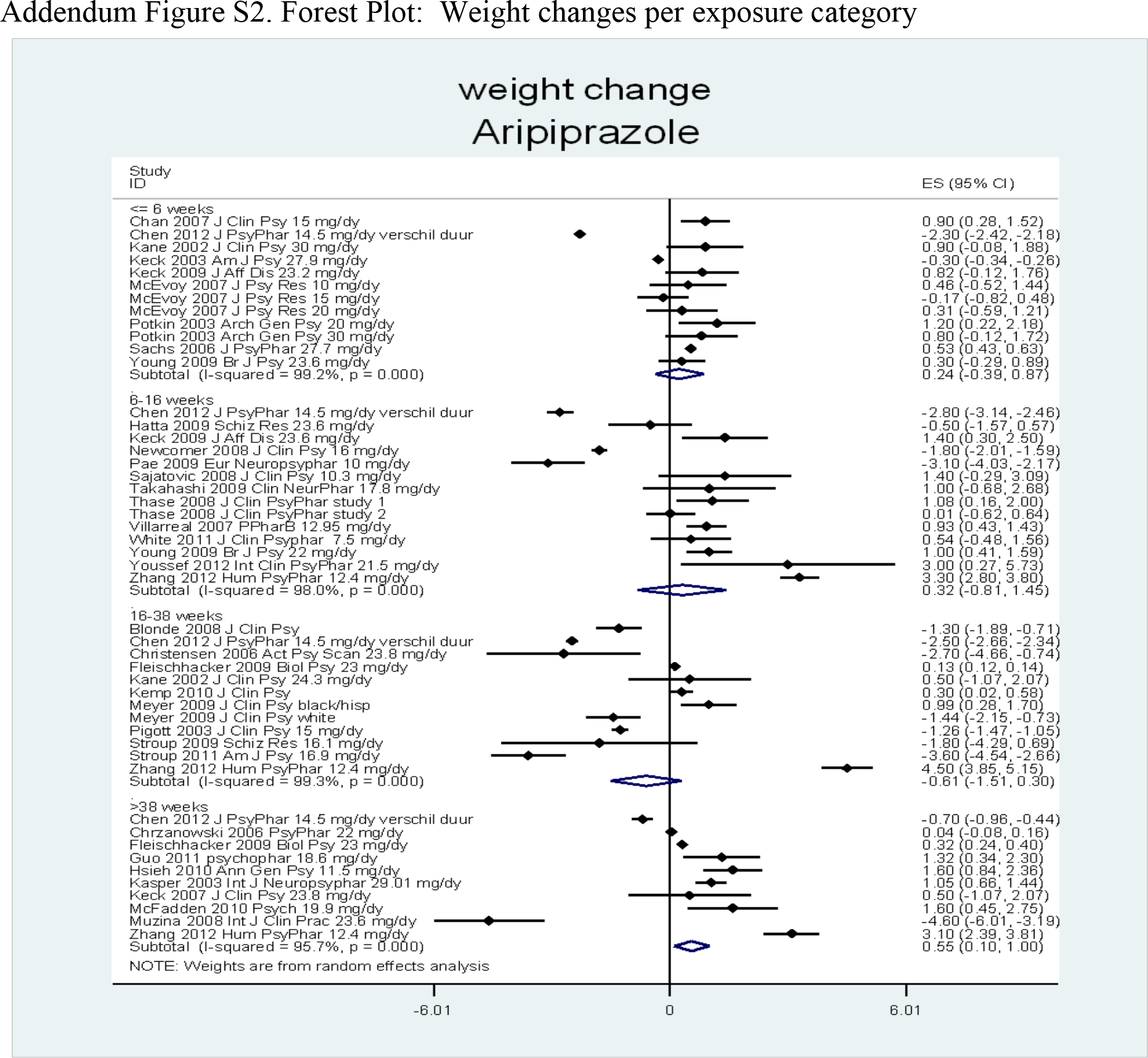

Supplement: File S1 — Forest Plots S1–S8 Weight changes per exposure category. (ZIP) [file pone.0094112.s002.zip › Aripiprazole Figure S2 Forest Plot.tif]

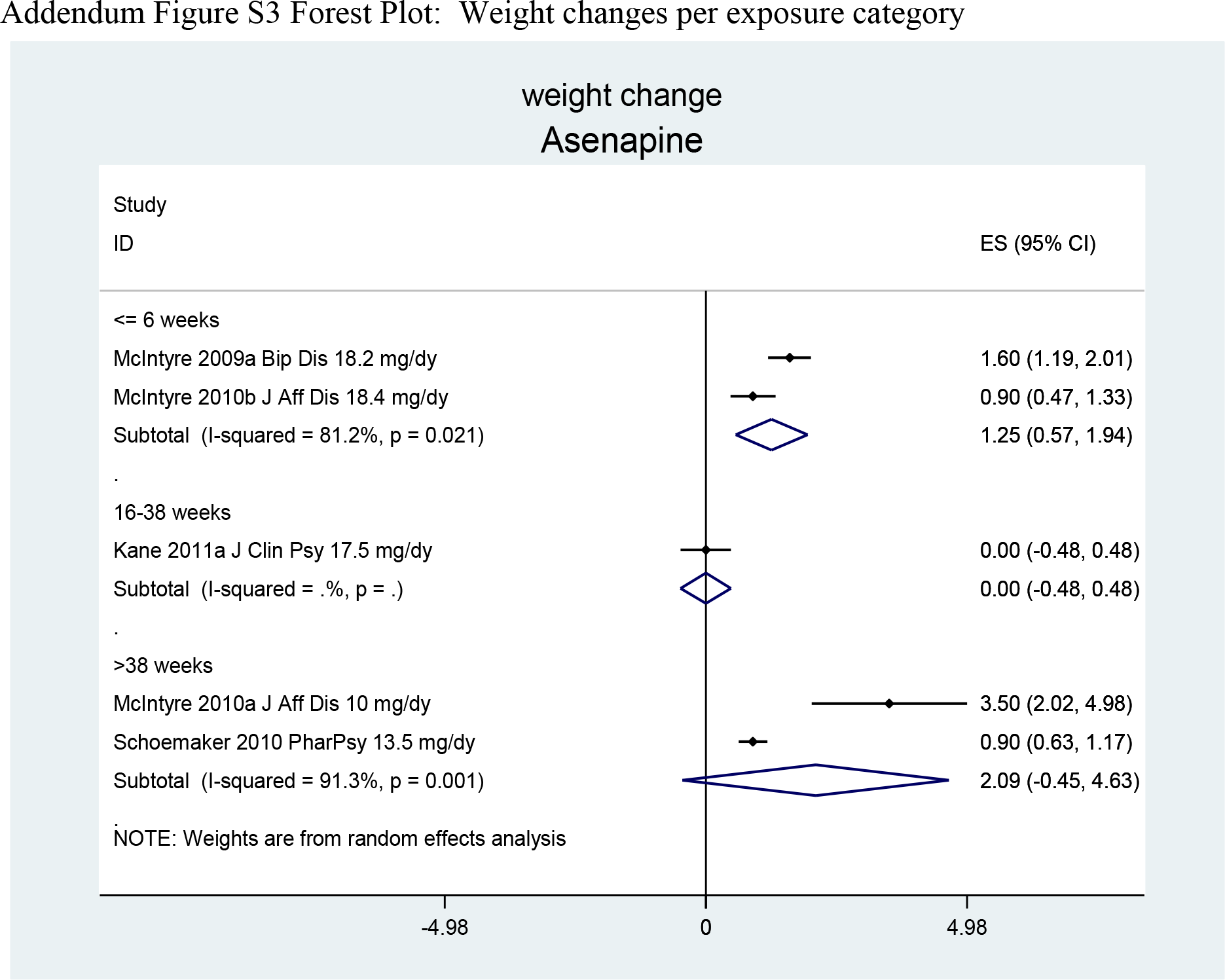

Supplement: File S1 — Forest Plots S1–S8 Weight changes per exposure category. (ZIP) [file pone.0094112.s002.zip › Asenapine Figure S3 Forest Plot.tif]

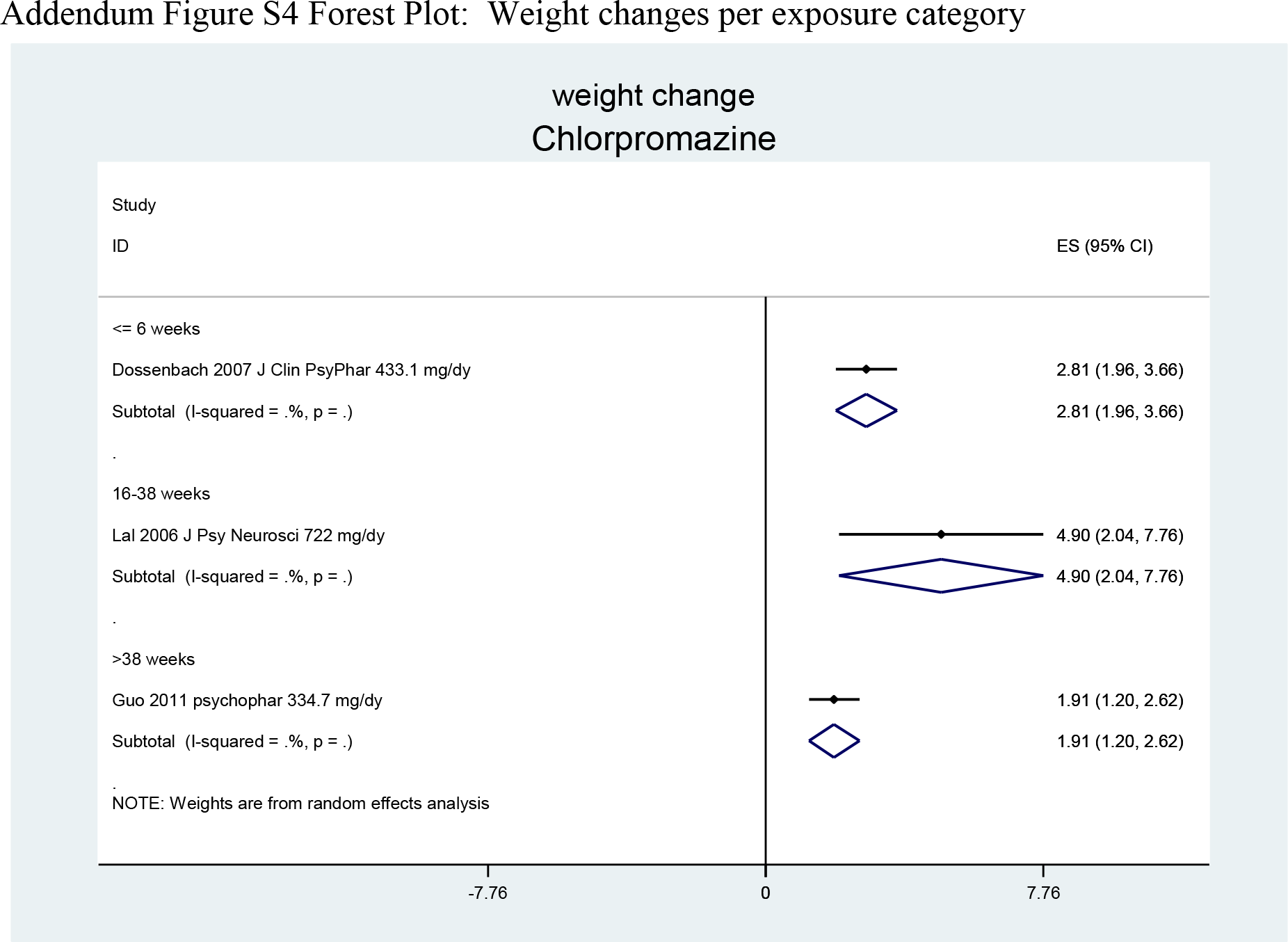

Supplement: File S1 — Forest Plots S1–S8 Weight changes per exposure category. (ZIP) [file pone.0094112.s002.zip › Chlorpromazine Figure S4 Forest Plot.tif]

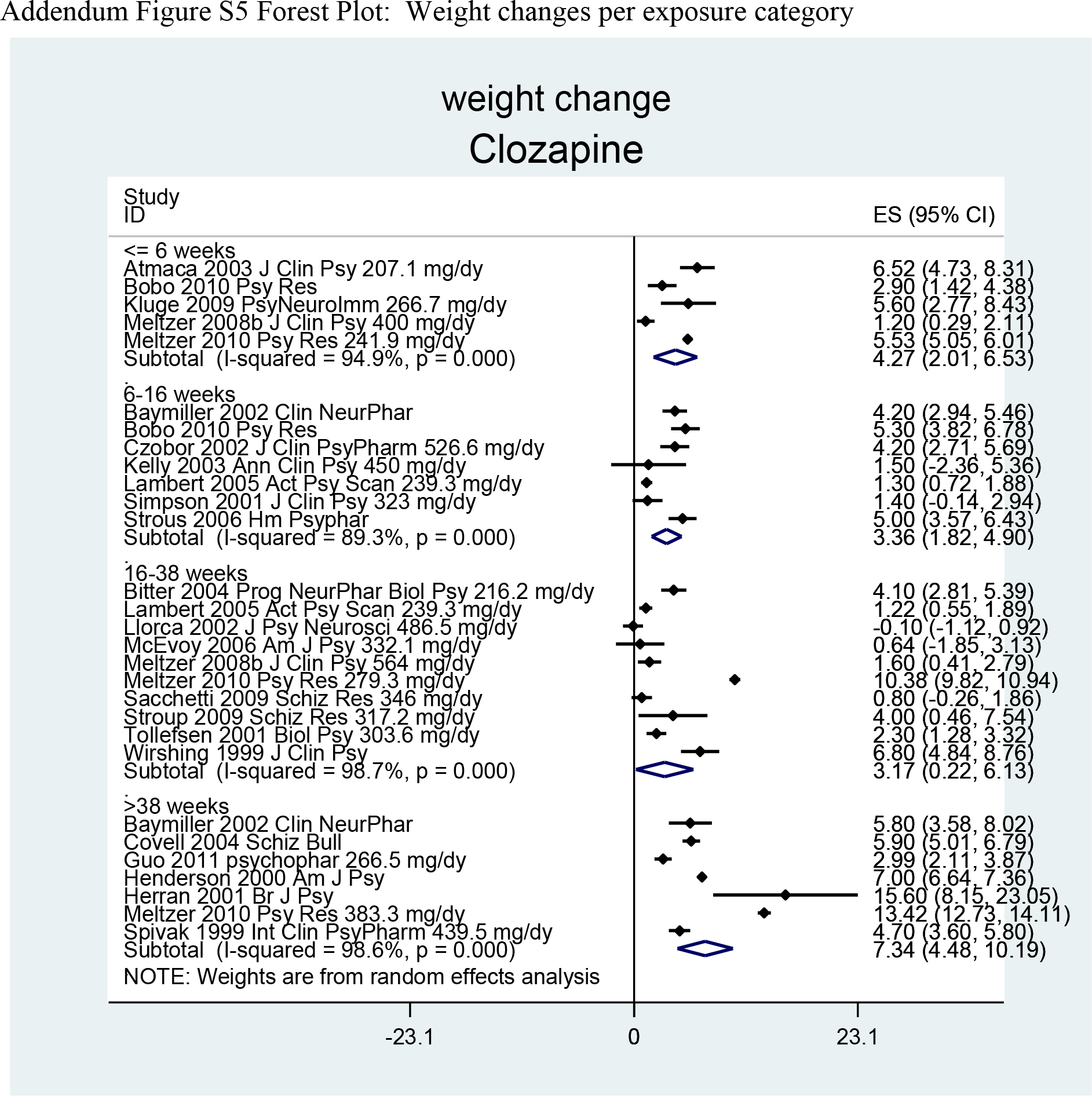

Supplement: File S1 — Forest Plots S1–S8 Weight changes per exposure category. (ZIP) [file pone.0094112.s002.zip › Clozapine Figure S5 Forest Plot.tif]

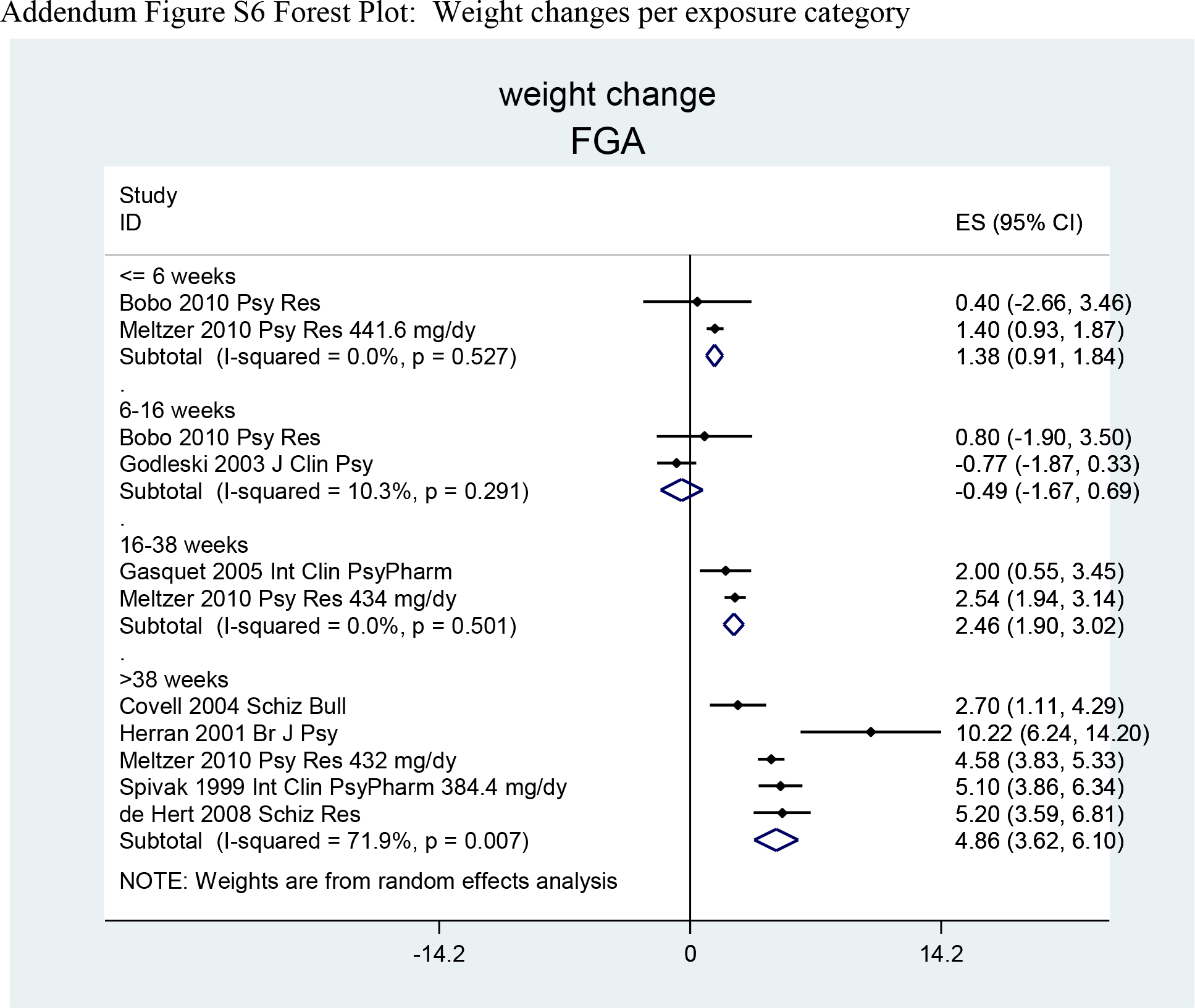

Supplement: File S1 — Forest Plots S1–S8 Weight changes per exposure category. (ZIP) [file pone.0094112.s002.zip › FGA Figure S6 Forest Plot.tif]

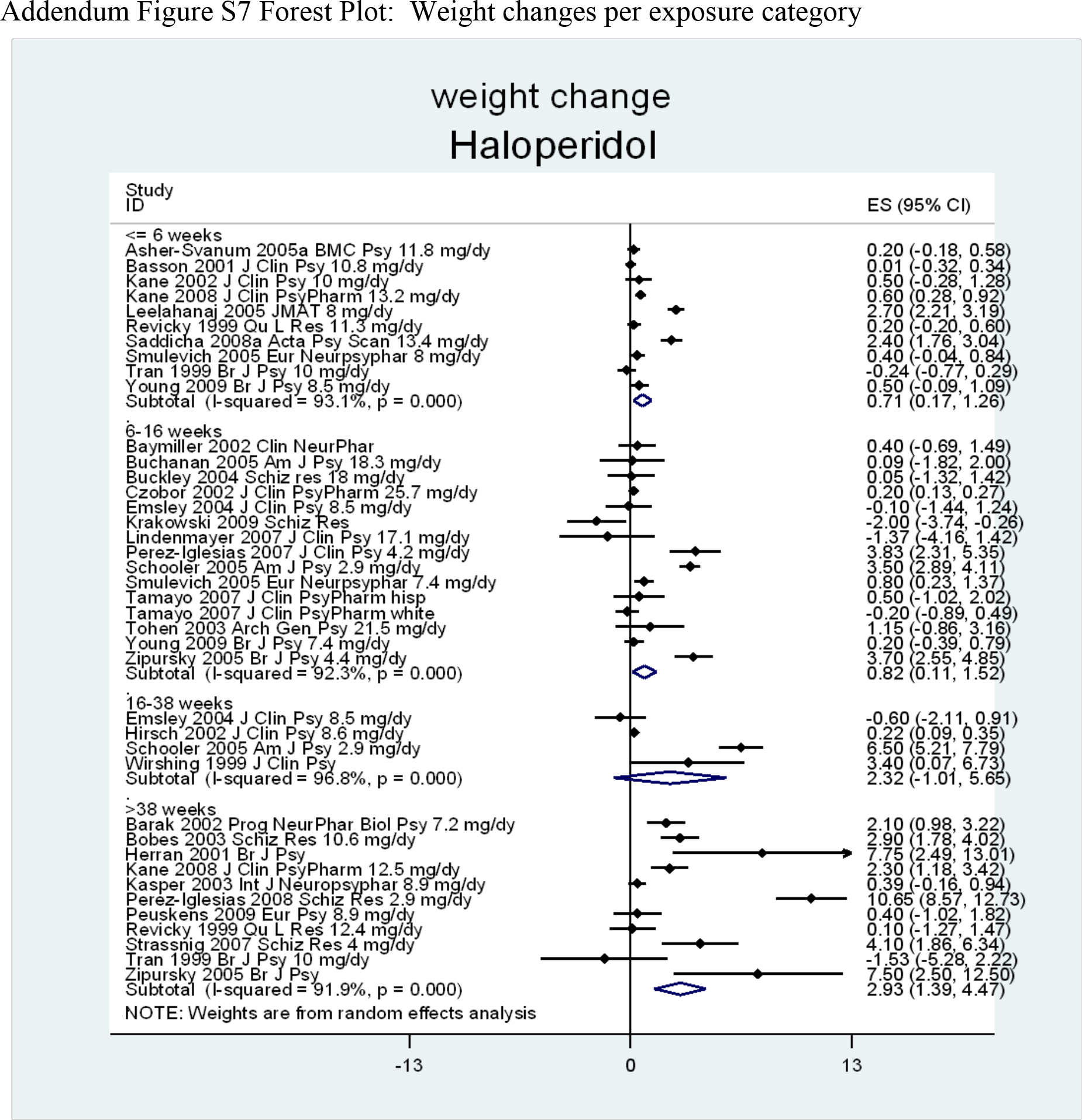

Supplement: File S1 — Forest Plots S1–S8 Weight changes per exposure category. (ZIP) [file pone.0094112.s002.zip › Haloperidol Figure S7 Forest Plot.tif]

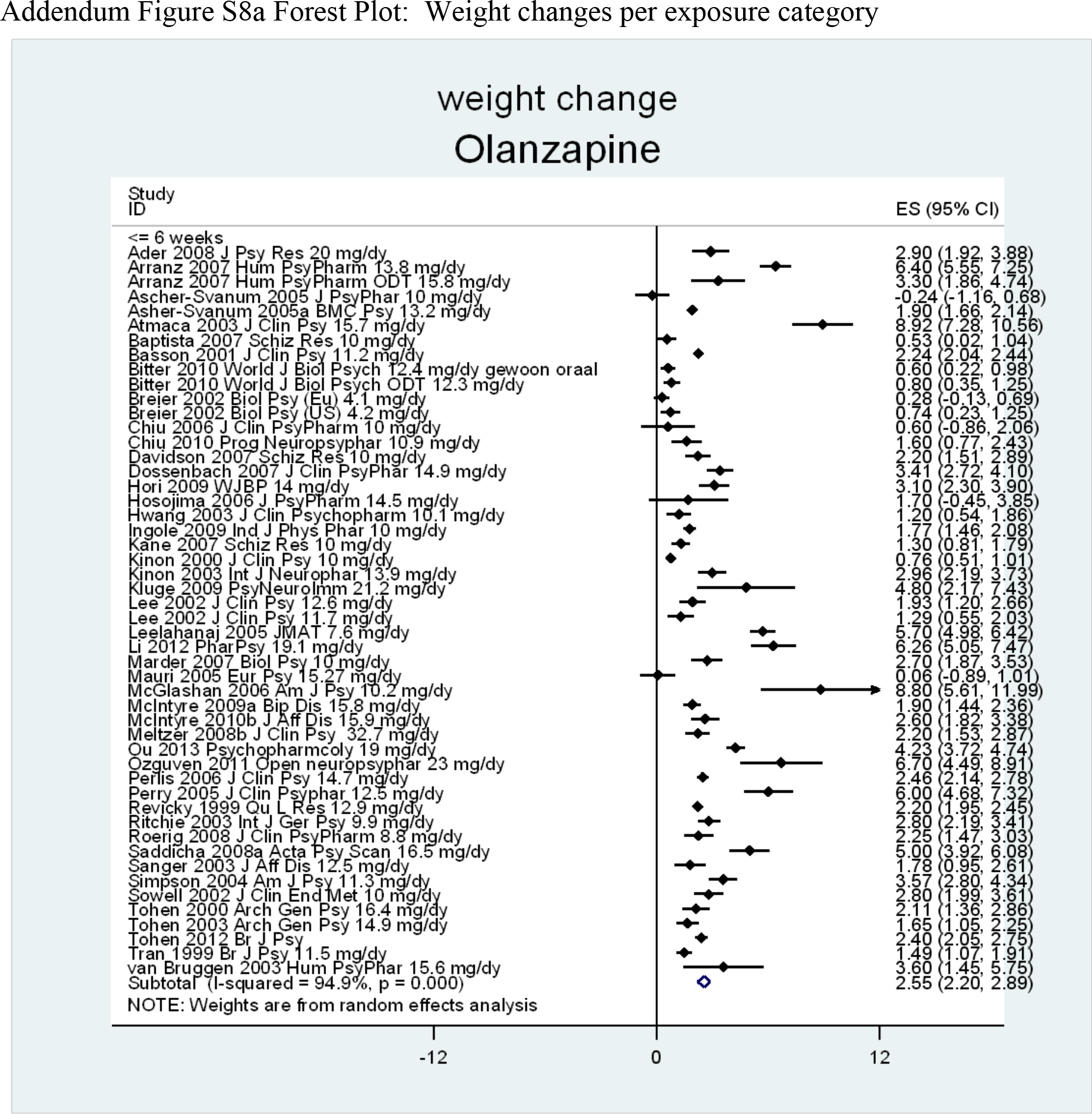

Supplement: File S1 — Forest Plots S1–S8 Weight changes per exposure category. (ZIP) [file pone.0094112.s002.zip › Olanzapine Figure S8a Forest Plot.tif]

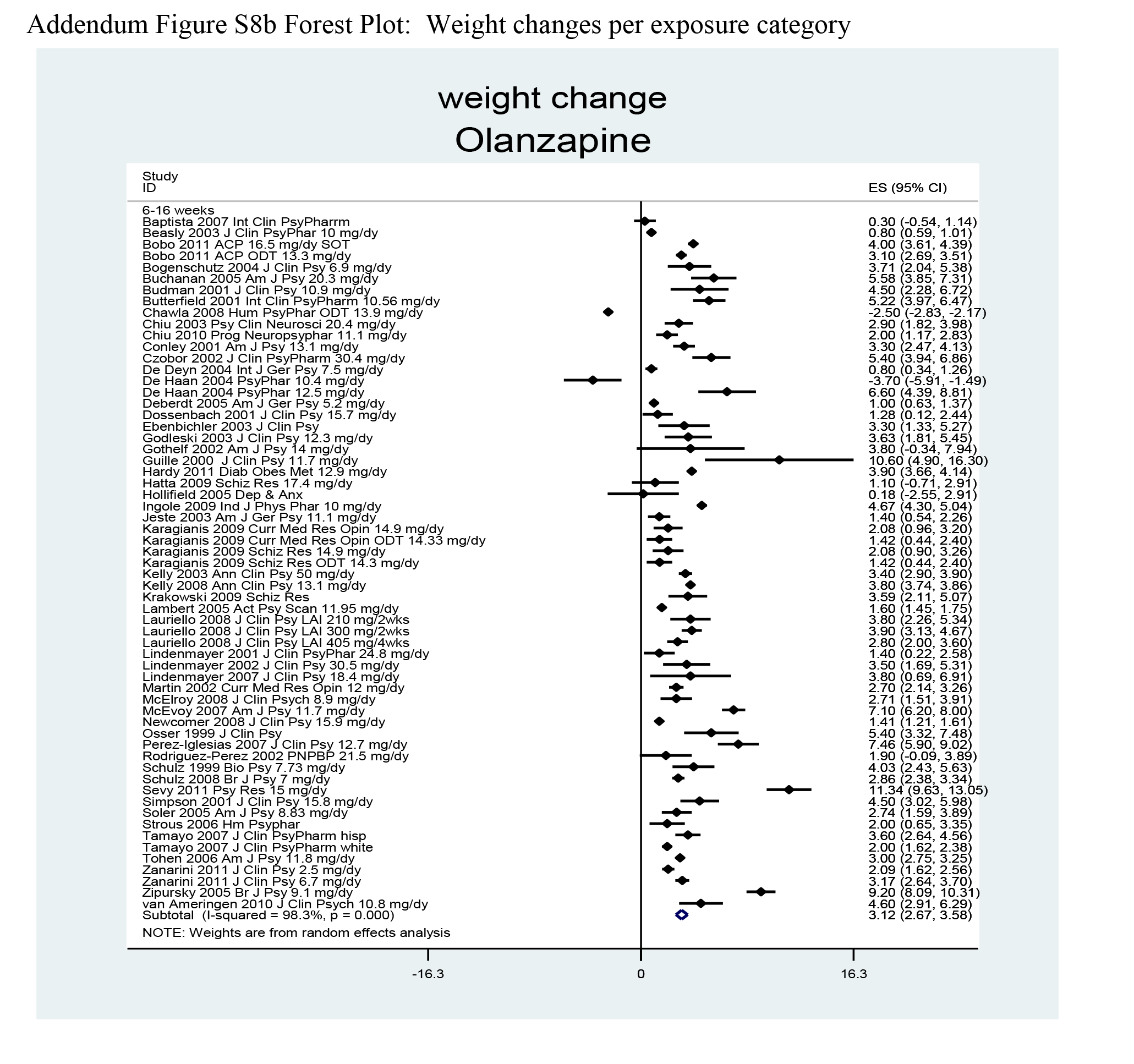

Supplement: File S1 — Forest Plots S1–S8 Weight changes per exposure category. (ZIP) [file pone.0094112.s002.zip › Olanzapine Figure S8b Forest Plot.tif]

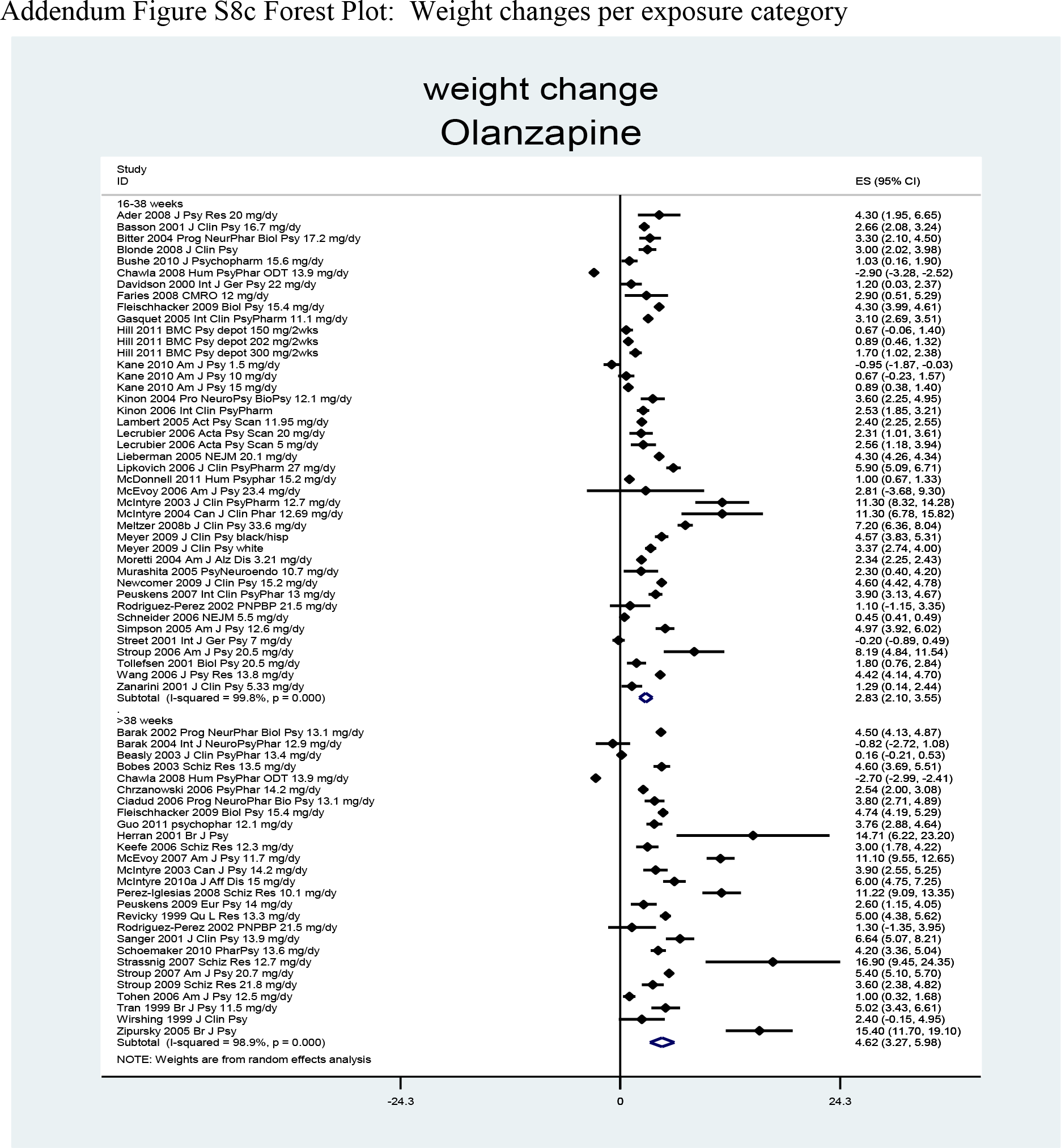

Supplement: File S1 — Forest Plots S1–S8 Weight changes per exposure category. (ZIP) [file pone.0094112.s002.zip › Olanzapine Figure S8c Forest Plot.tif]

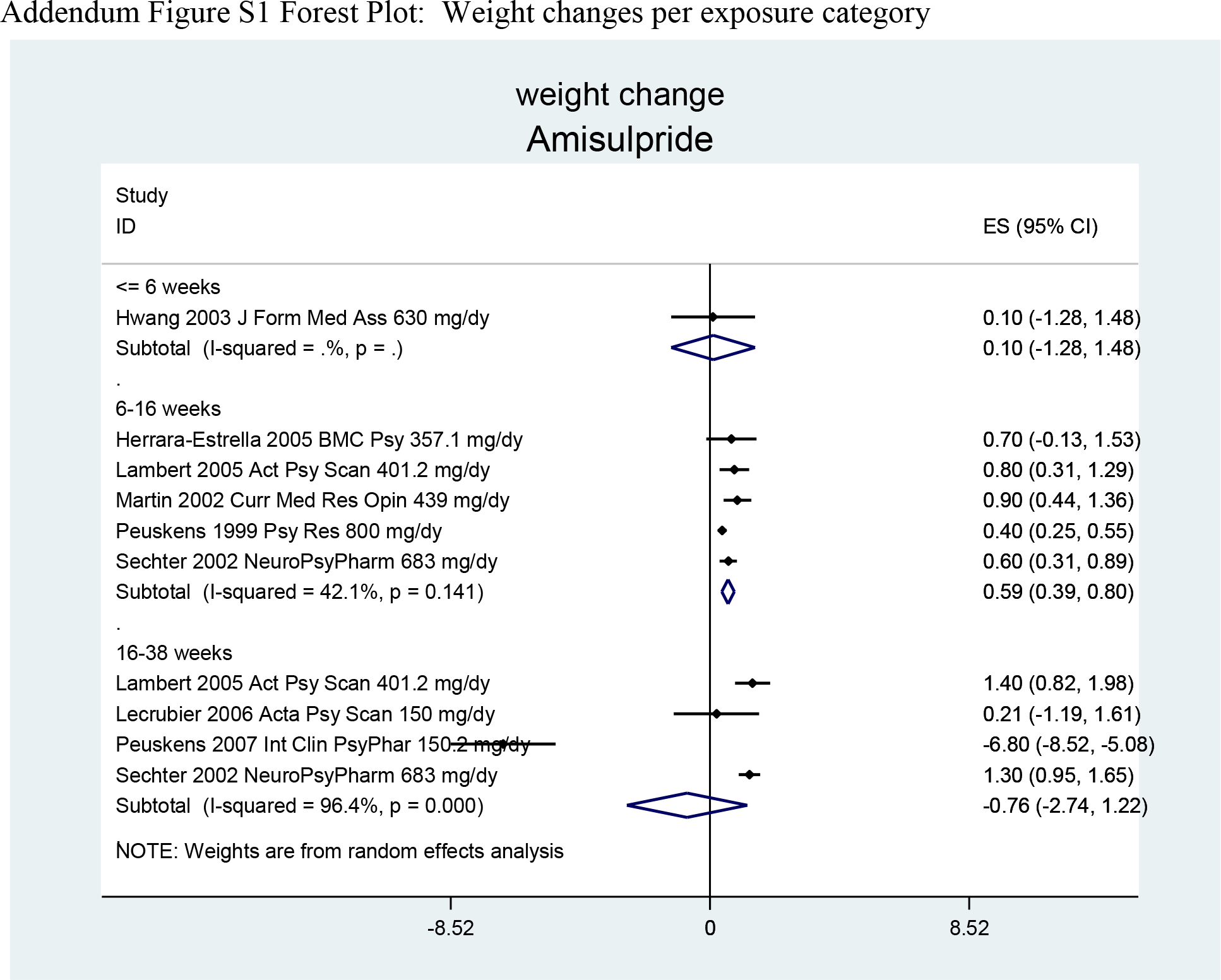

Supplement: File S1 — Forest Plots S1–S8 Weight changes per exposure category. (ZIP) [file pone.0094112.s002.zip › Amisulpride Figure S1 Forest Plot.tif]

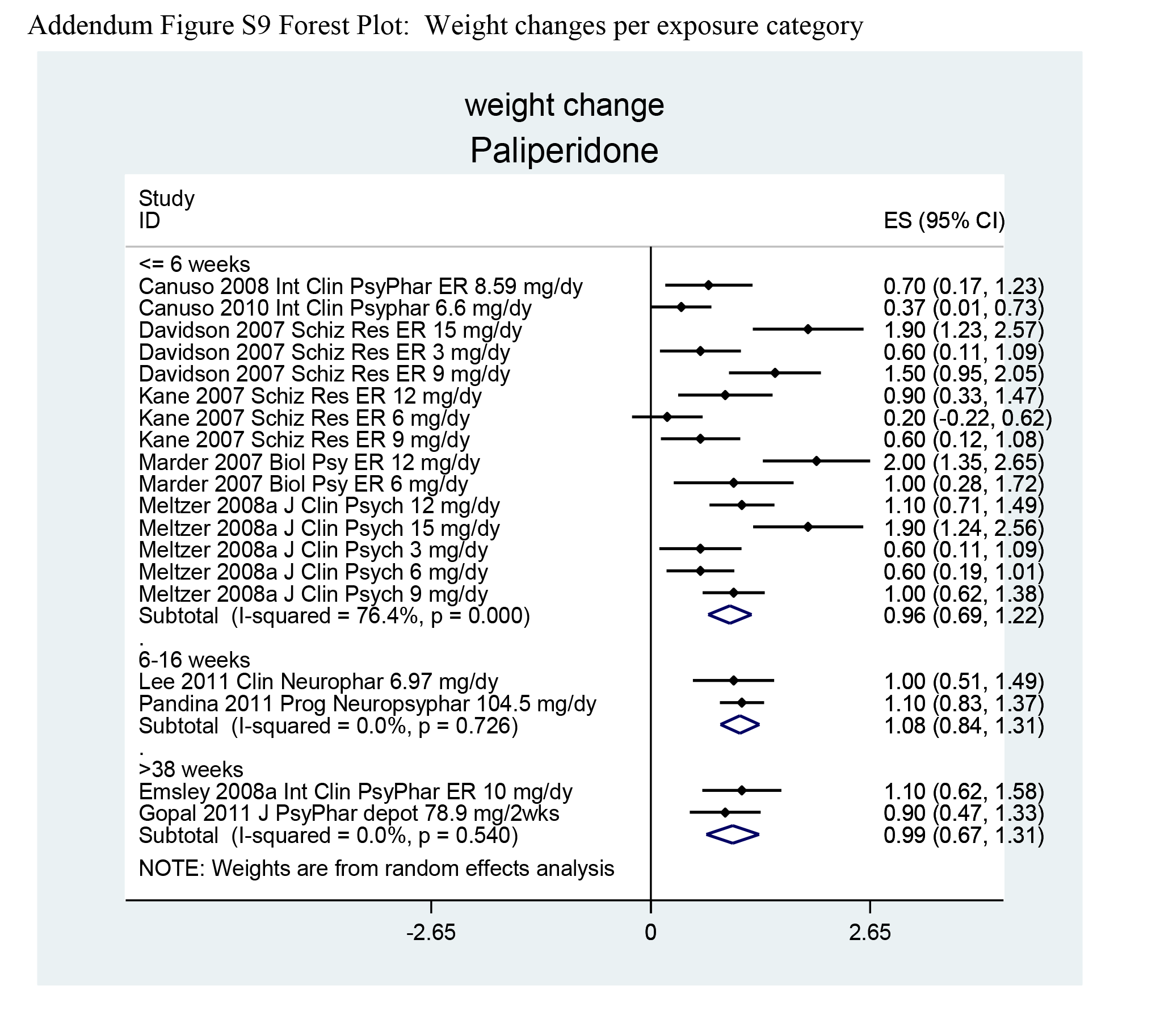

Supplement: File S2 — Forets Plots S9–S16 Weight changes per exposure category. (ZIP) [file pone.0094112.s003.zip › Pailperidone Figure S9 Forest Plot.tif]

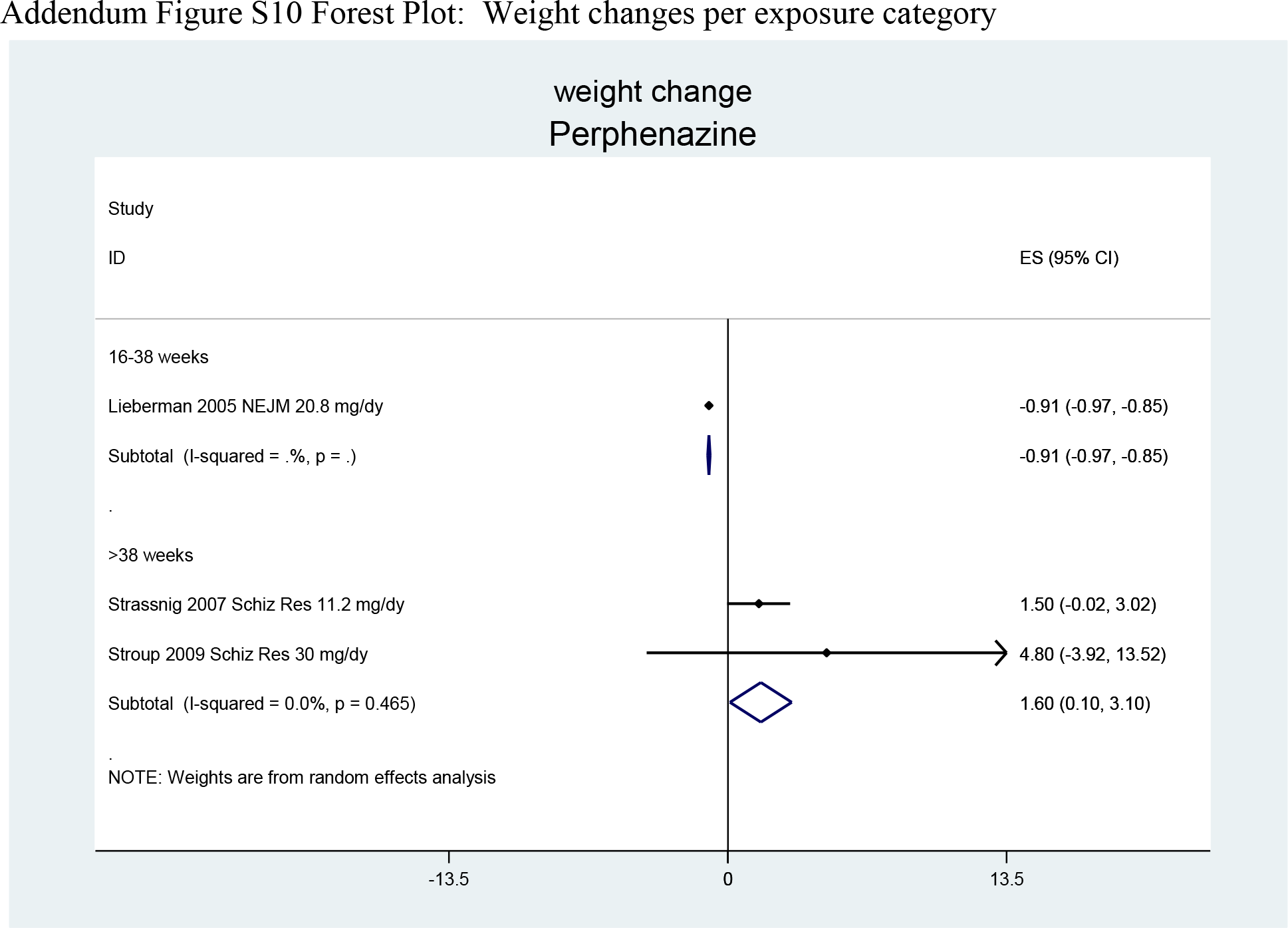

Supplement: File S2 — Forets Plots S9–S16 Weight changes per exposure category. (ZIP) [file pone.0094112.s003.zip › Perphenazine Figure S10 Forest Plot.tif]

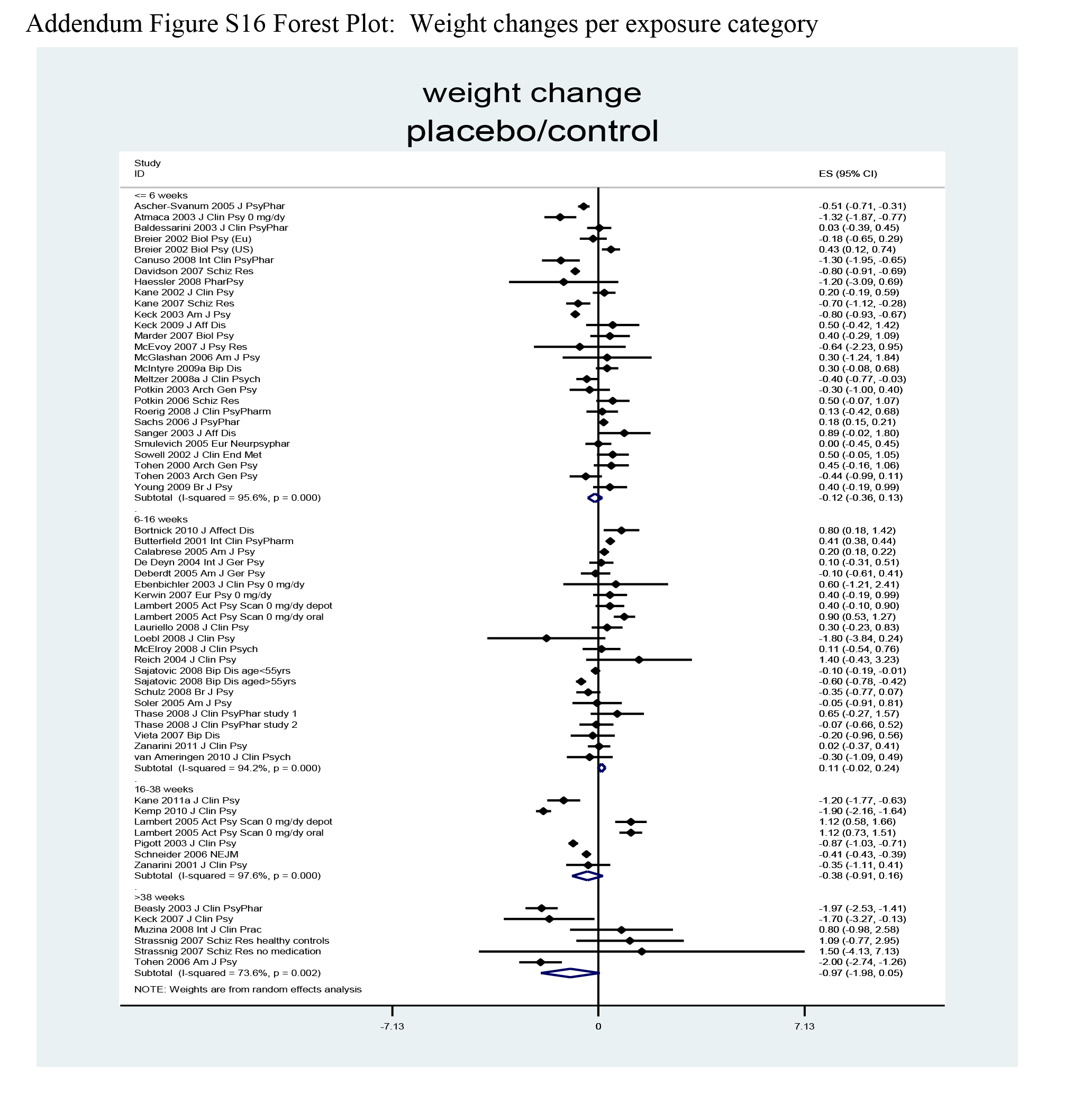

Supplement: File S2 — Forets Plots S9–S16 Weight changes per exposure category. (ZIP) [file pone.0094112.s003.zip › Placebo Figure S16 Forest Plot.tif]

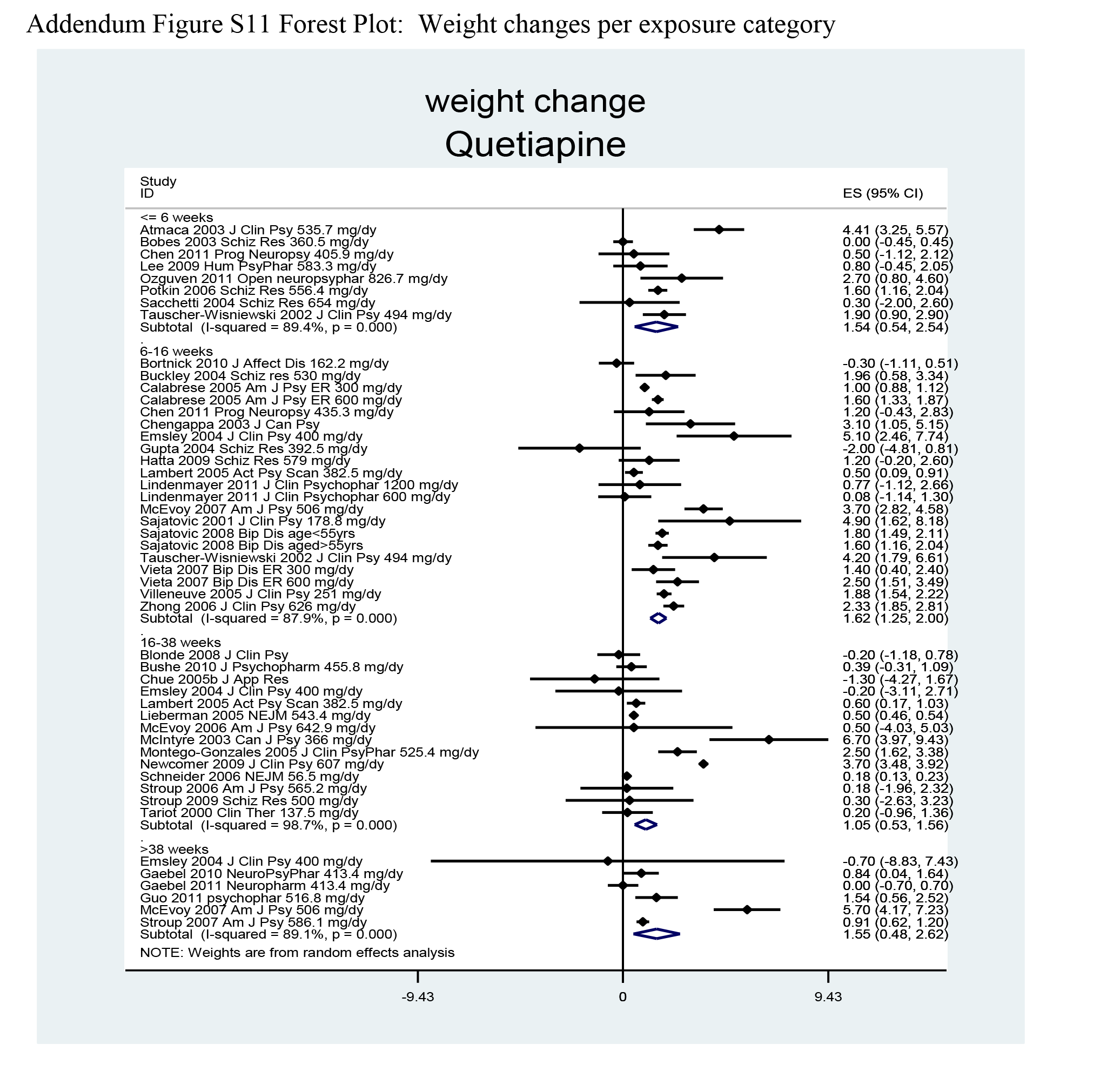

Supplement: File S2 — Forets Plots S9–S16 Weight changes per exposure category. (ZIP) [file pone.0094112.s003.zip › Quetiapine Figure S11 Forest Plot.tif]

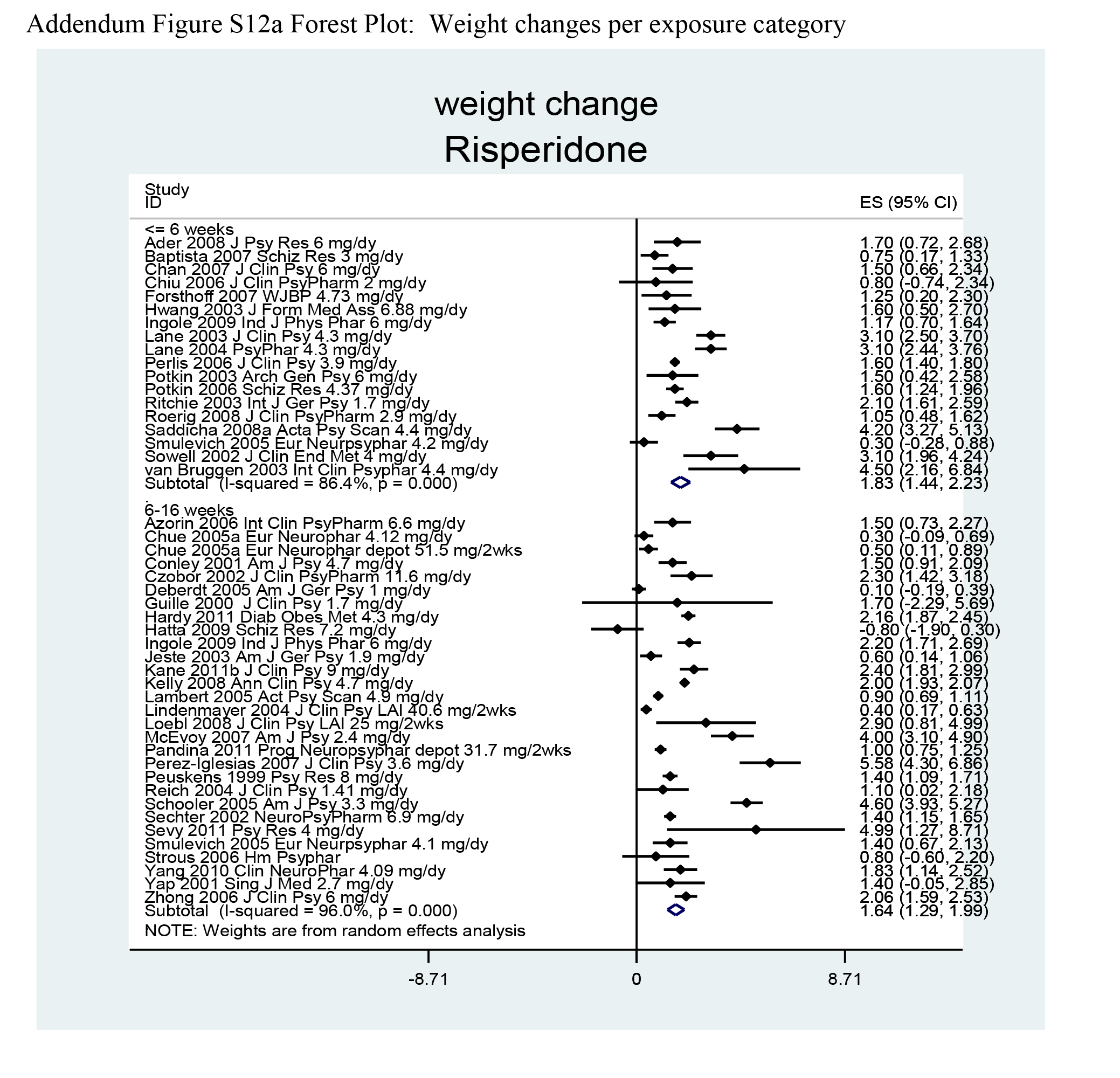

Supplement: File S2 — Forets Plots S9–S16 Weight changes per exposure category. (ZIP) [file pone.0094112.s003.zip › Risperidone Figure S12a Forest Plot.tif]

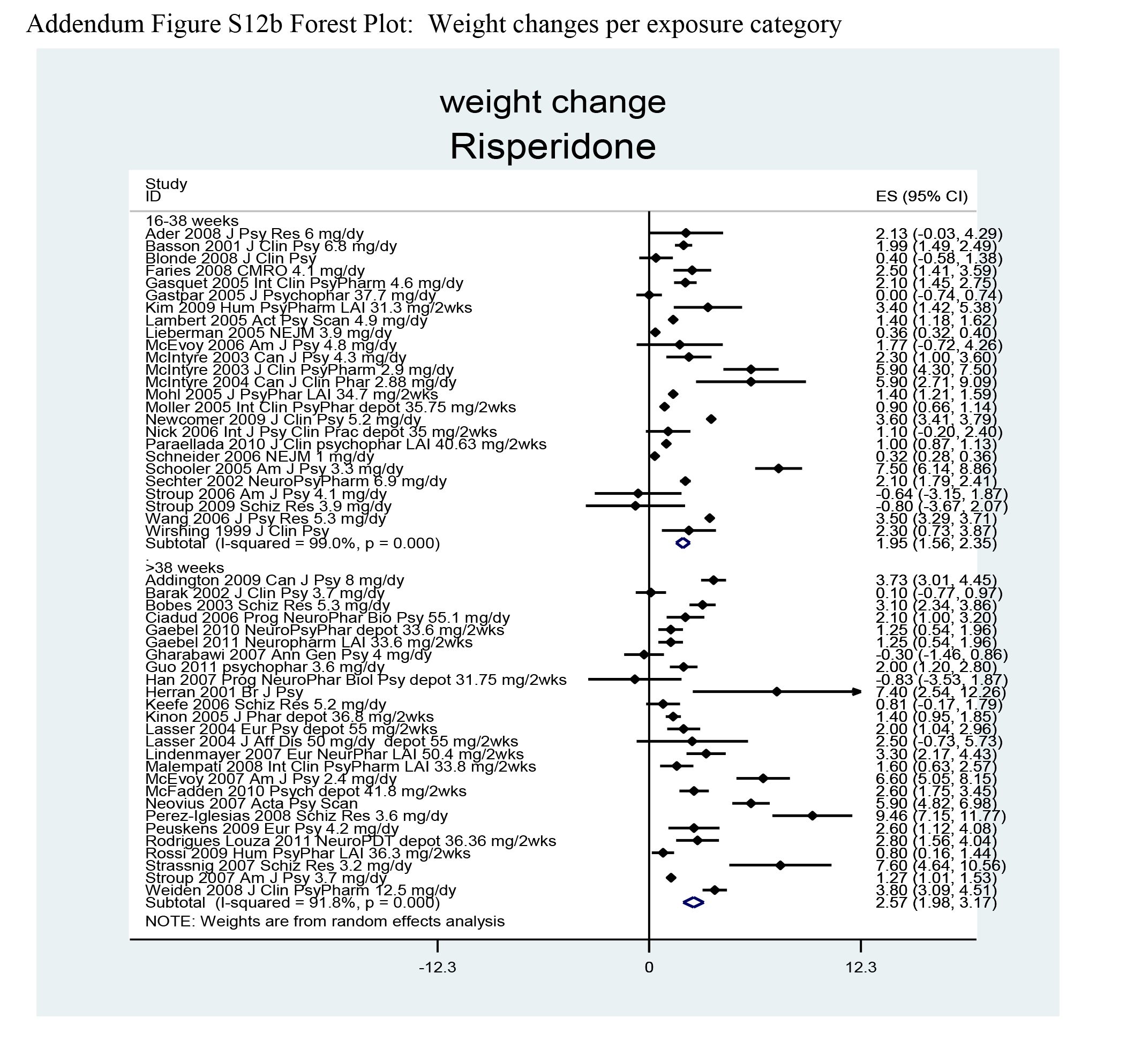

Supplement: File S2 — Forets Plots S9–S16 Weight changes per exposure category. (ZIP) [file pone.0094112.s003.zip › Rispridone Figure S12b Forest Plot.tif]

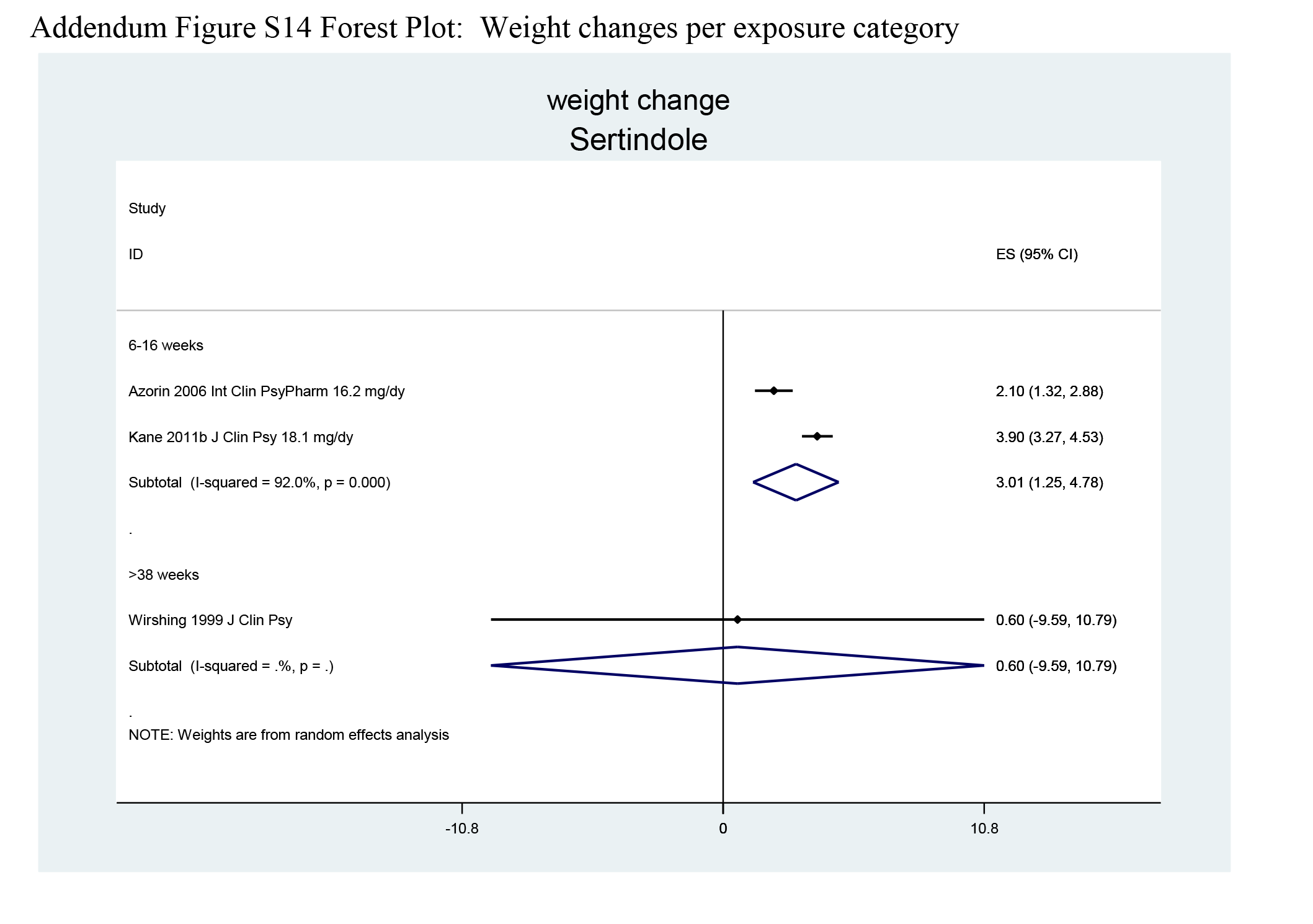

Supplement: File S2 — Forets Plots S9–S16 Weight changes per exposure category. (ZIP) [file pone.0094112.s003.zip › Sertindole Figure S14 Forest Plot.tif]

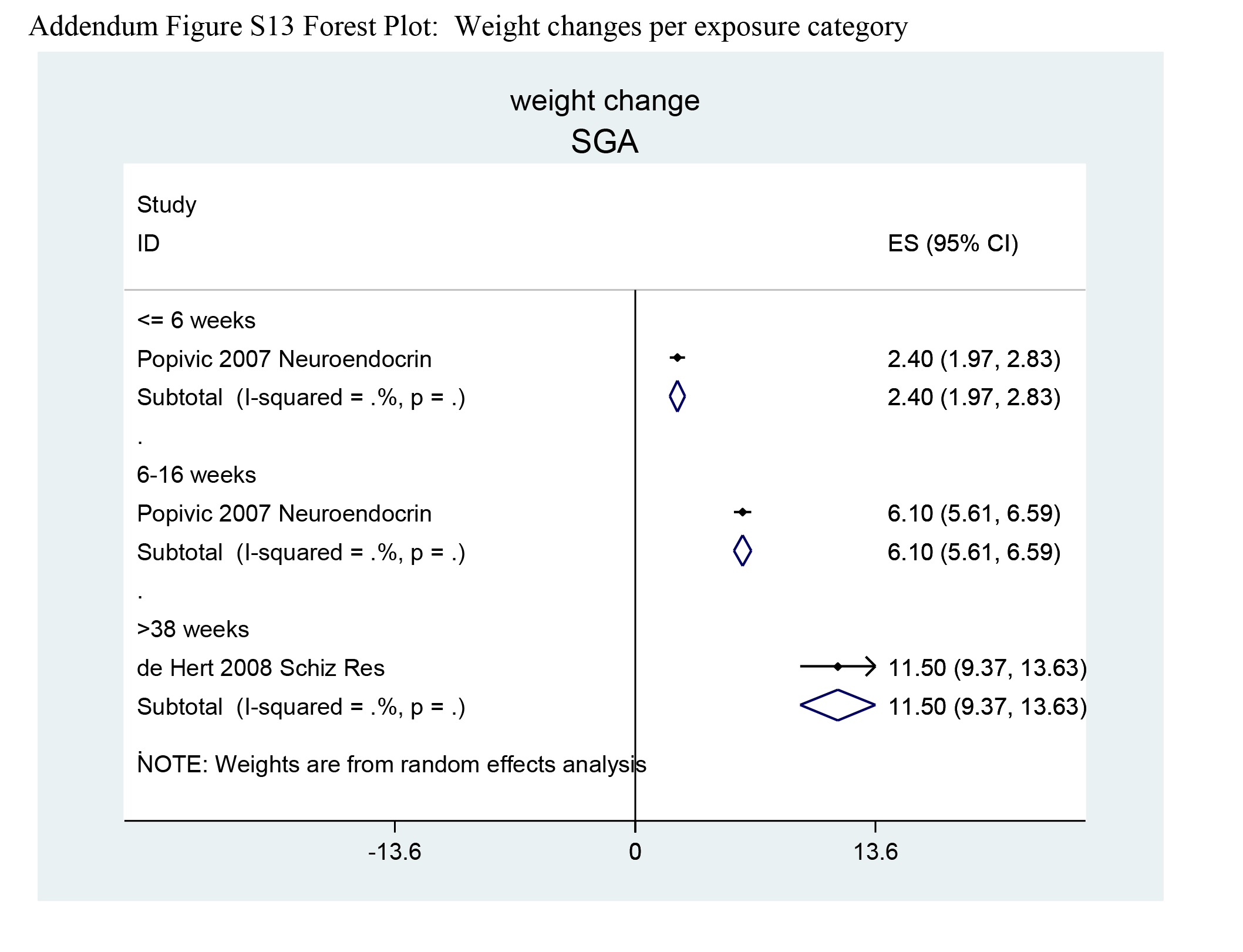

Supplement: File S2 — Forets Plots S9–S16 Weight changes per exposure category. (ZIP) [file pone.0094112.s003.zip › SGA Figure S13 Forest Plot.tif]

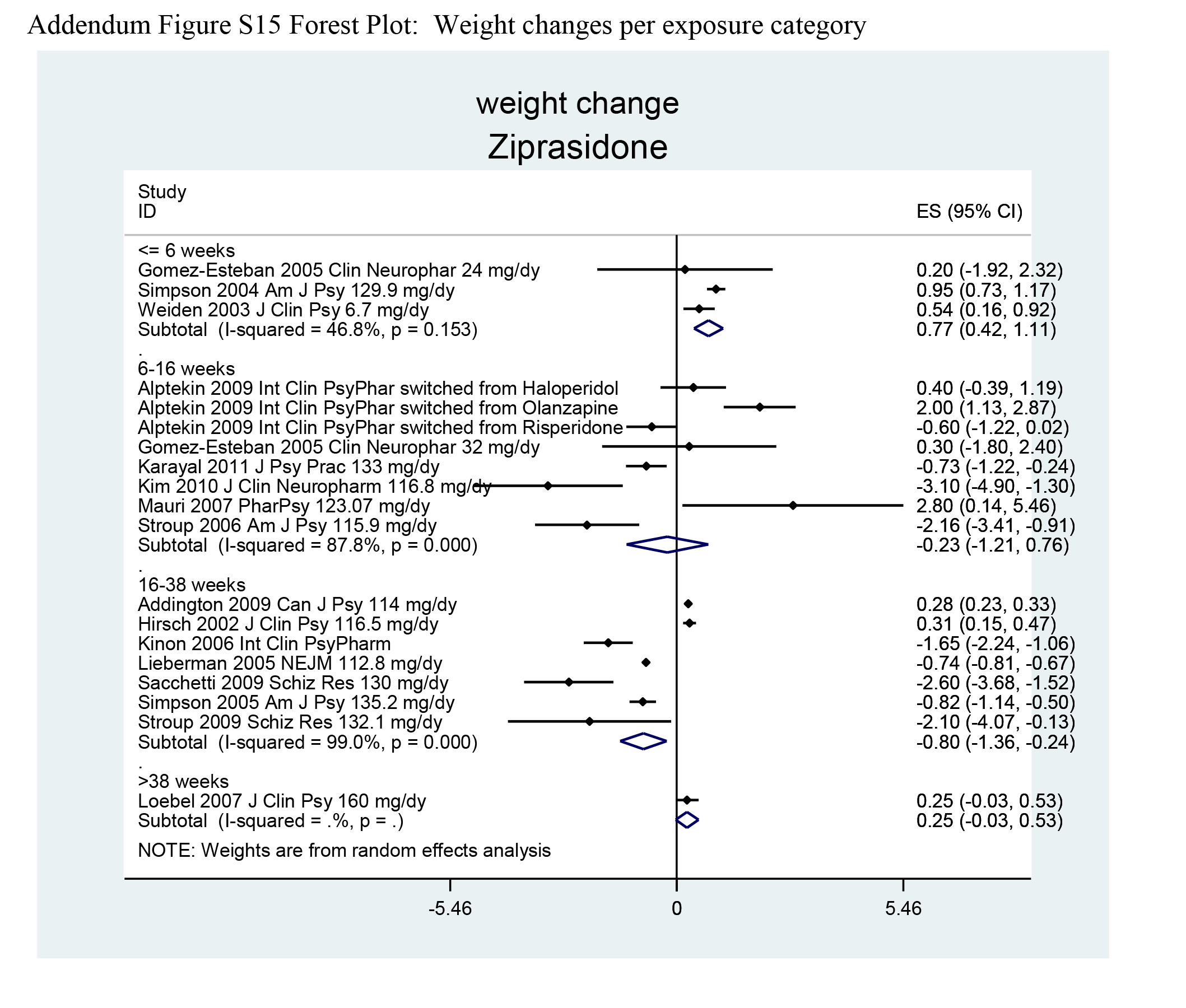

Supplement: File S2 — Forets Plots S9–S16 Weight changes per exposure category. (ZIP) [file pone.0094112.s003.zip › Ziprasidone Figure S15 Forest Plot.tif]

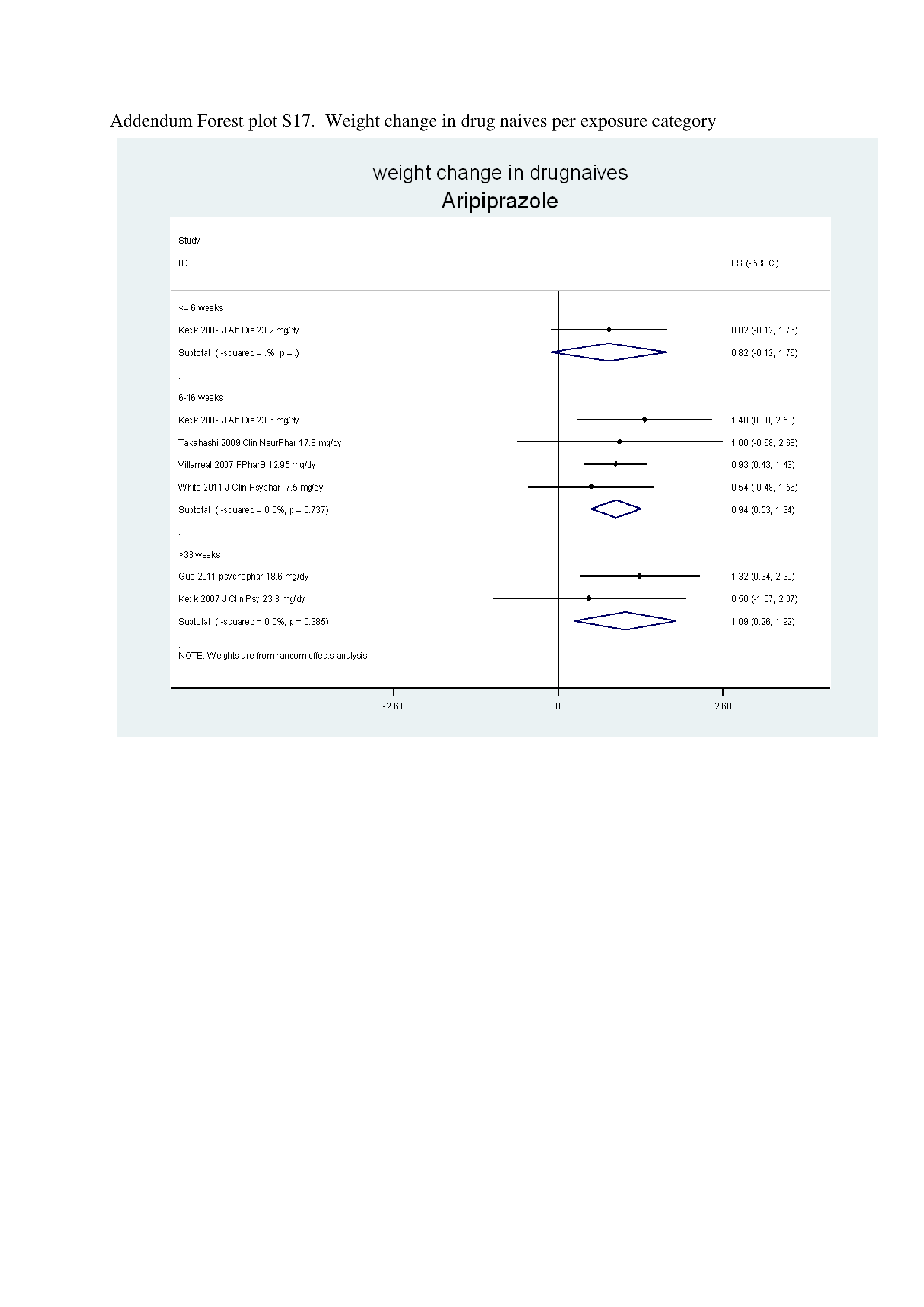

Supplement: File S3 — Forest plots S17–S24. Weight changes in AP naives per exposure category. (ZIP) [file pone.0094112.s004.zip › aripiprazole Figure S17 Forest Plot.tiff]

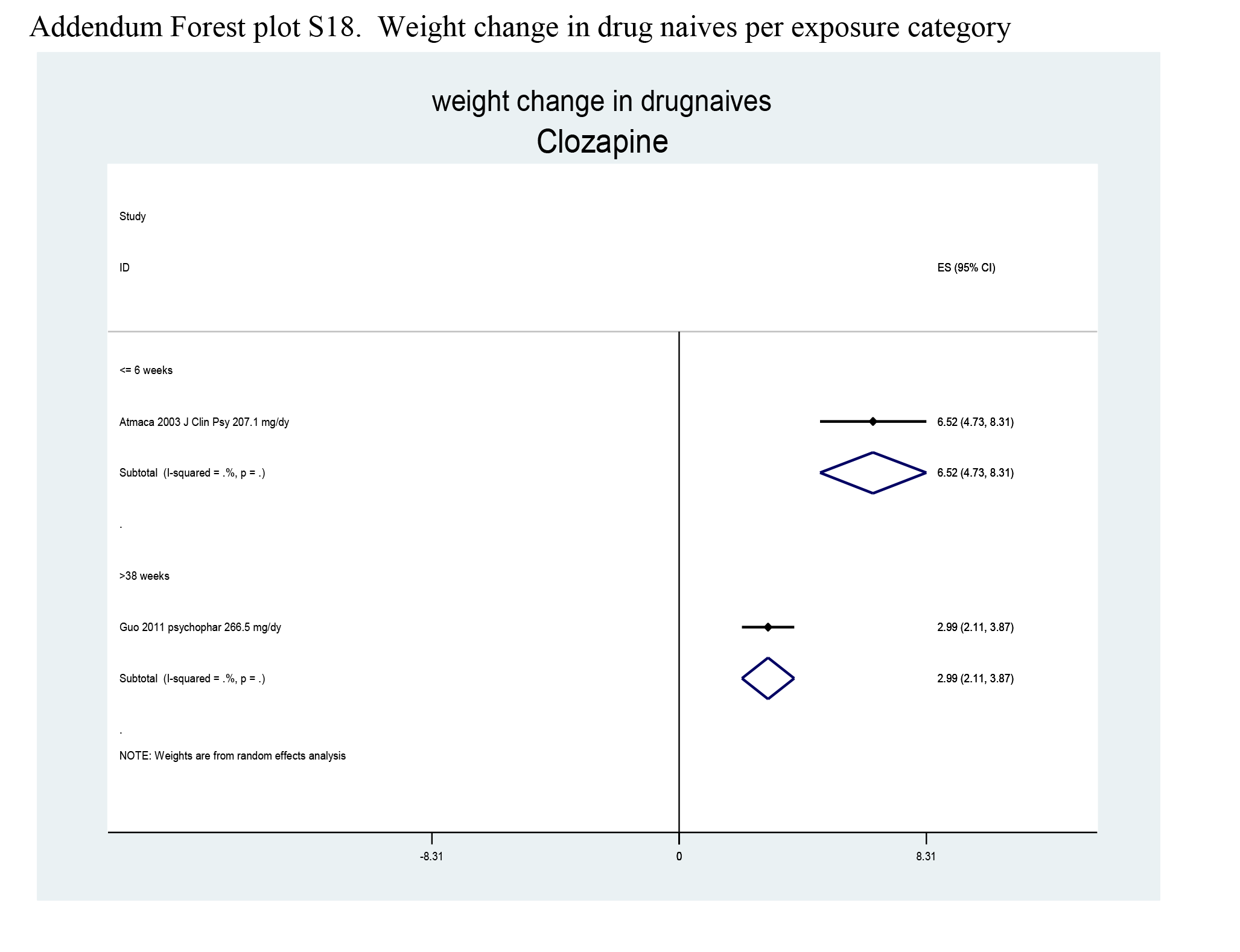

Supplement: File S3 — Forest plots S17–S24. Weight changes in AP naives per exposure category. (ZIP) [file pone.0094112.s004.zip › Clozapine Figure S18 Forest Plot.tif]

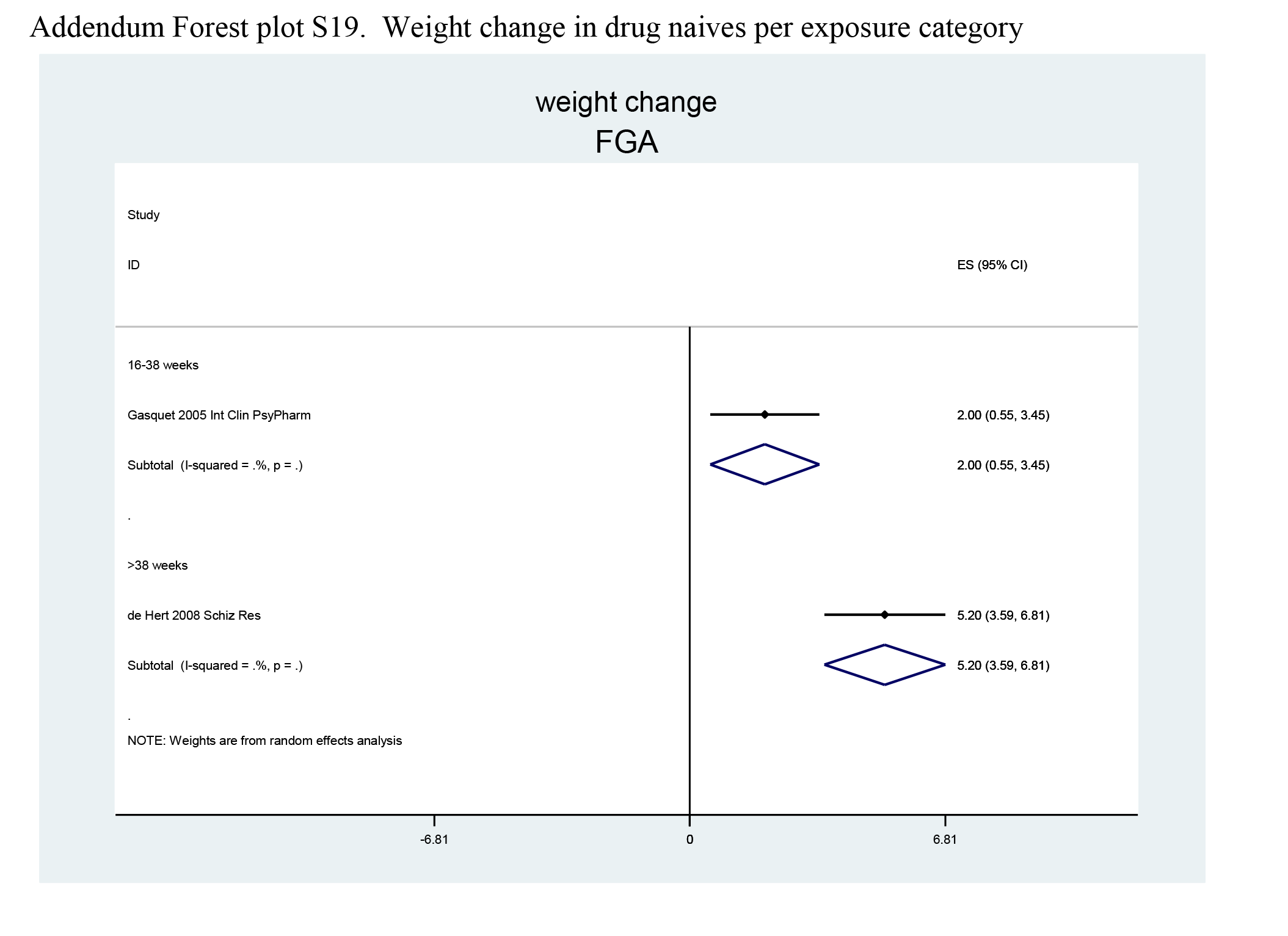

Supplement: File S3 — Forest plots S17–S24. Weight changes in AP naives per exposure category. (ZIP) [file pone.0094112.s004.zip › FGA Figure S19 Forest Plot.tif]

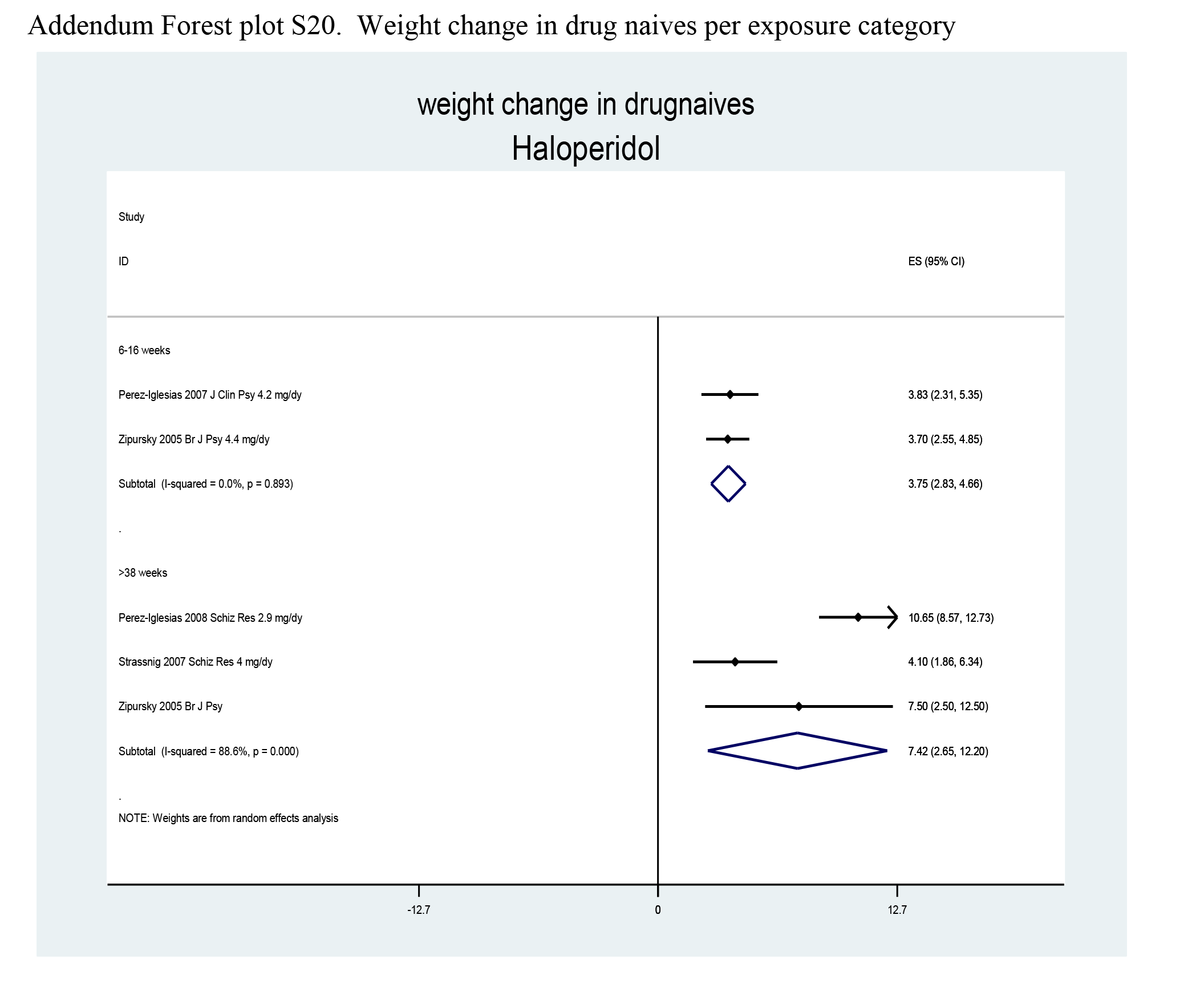

Supplement: File S3 — Forest plots S17–S24. Weight changes in AP naives per exposure category. (ZIP) [file pone.0094112.s004.zip › Haloperidol Figure S20 Forest Plot.tif]

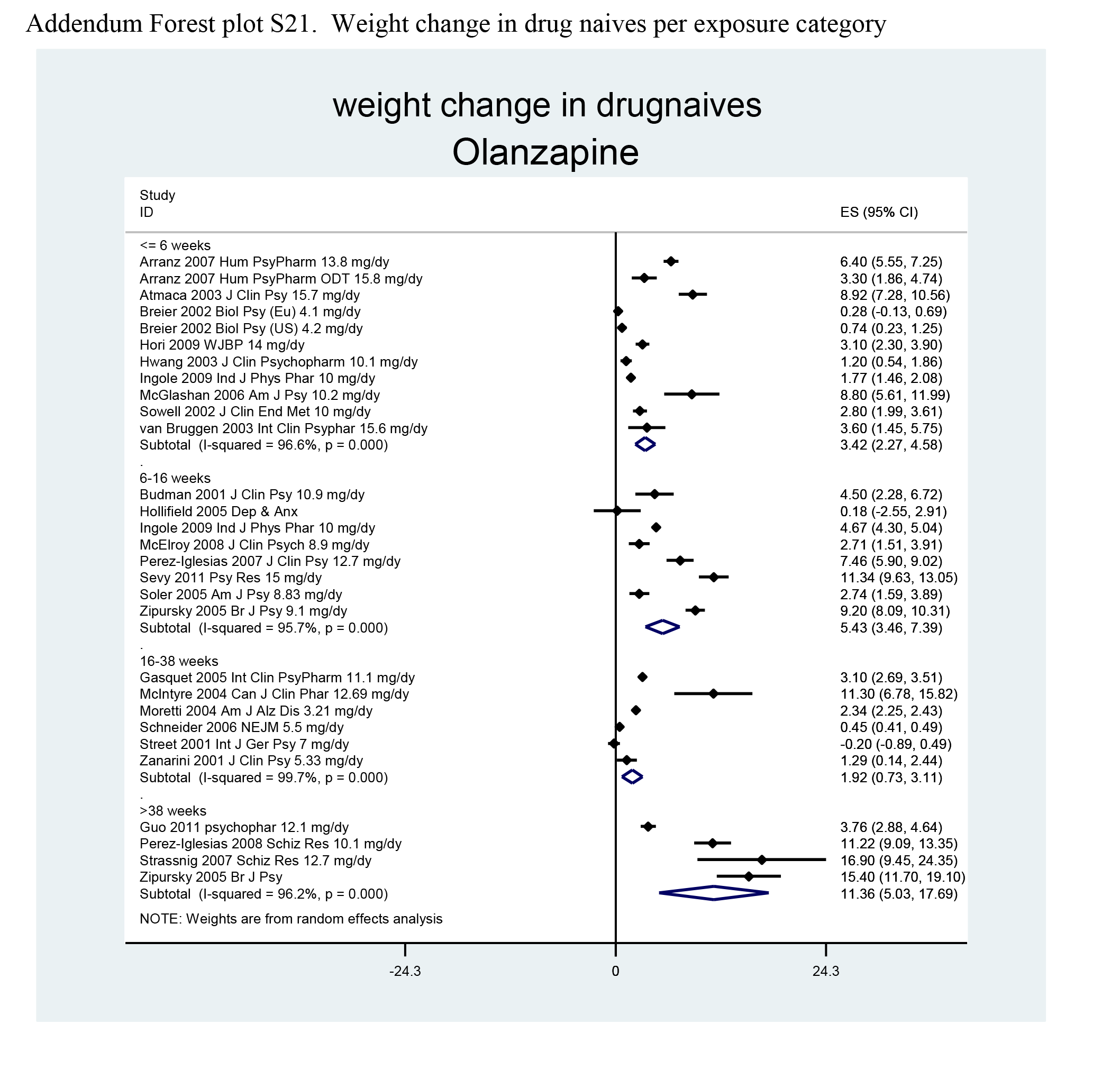

Supplement: File S3 — Forest plots S17–S24. Weight changes in AP naives per exposure category. (ZIP) [file pone.0094112.s004.zip › Olanzapine Figure S21 Forest Plot.tif]

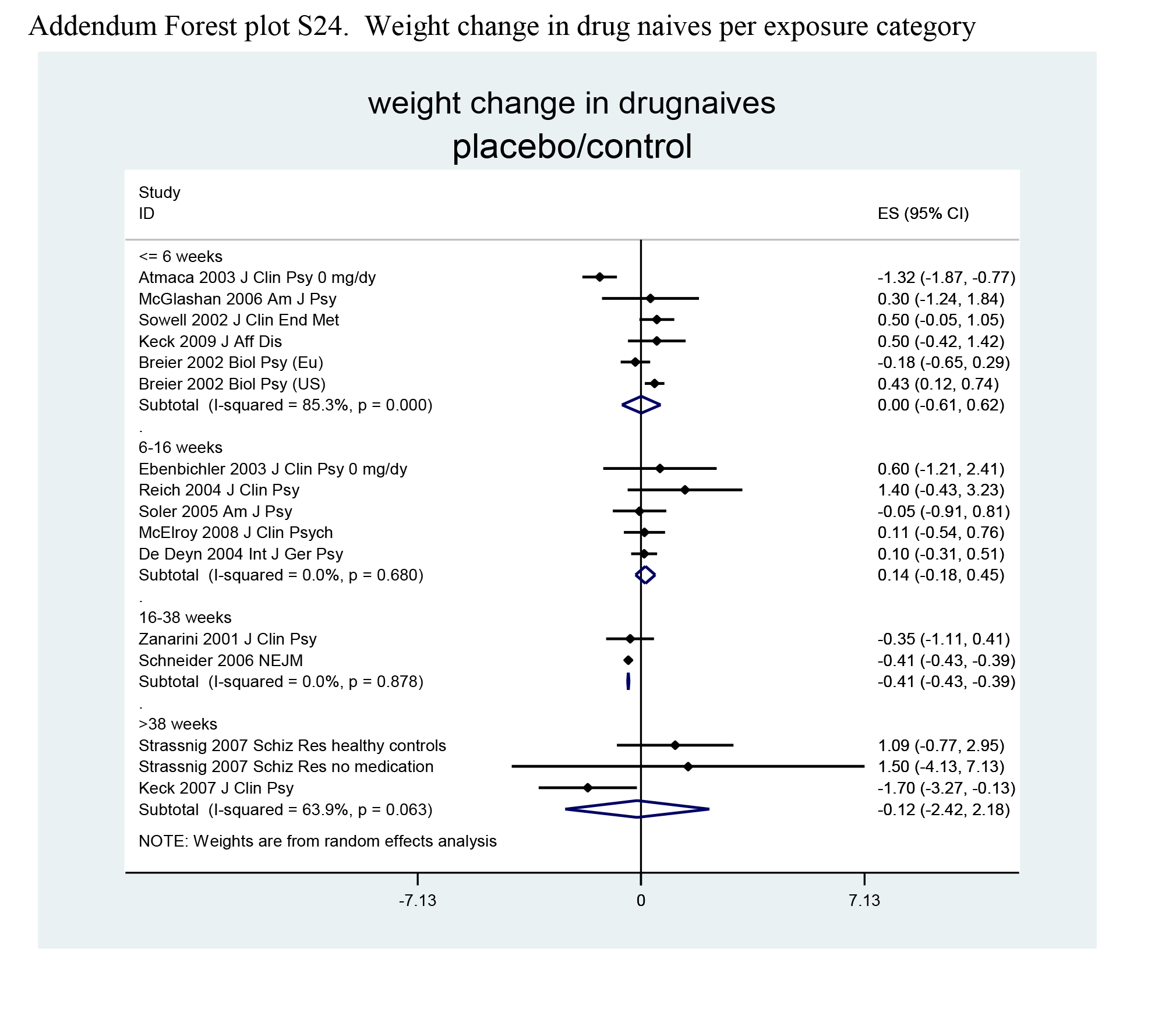

Supplement: File S3 — Forest plots S17–S24. Weight changes in AP naives per exposure category. (ZIP) [file pone.0094112.s004.zip › Placebo Figure S24 Forest Plot.tif]

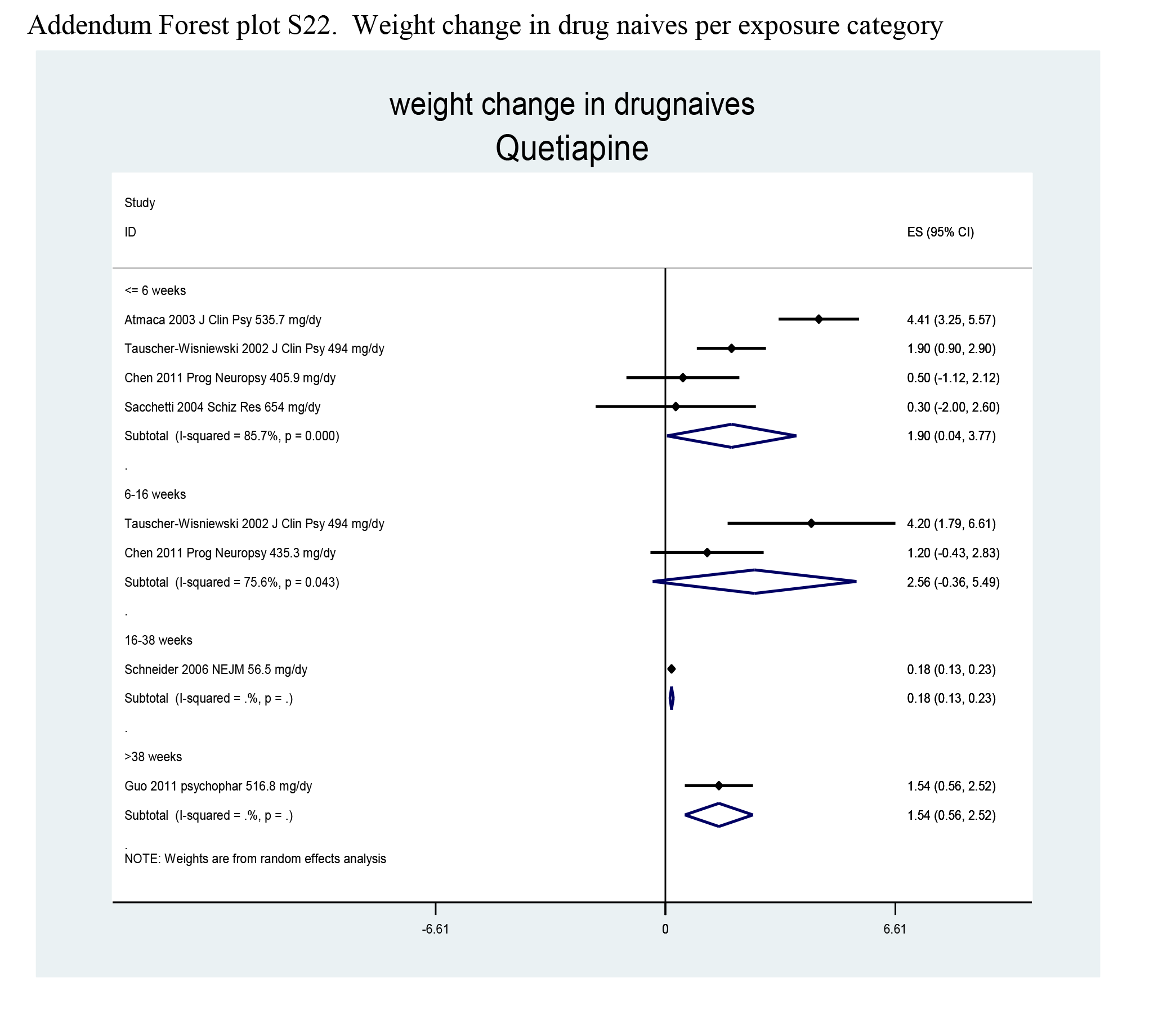

Supplement: File S3 — Forest plots S17–S24. Weight changes in AP naives per exposure category. (ZIP) [file pone.0094112.s004.zip › Quetiapine Figure S22 Forest Plot.tif]

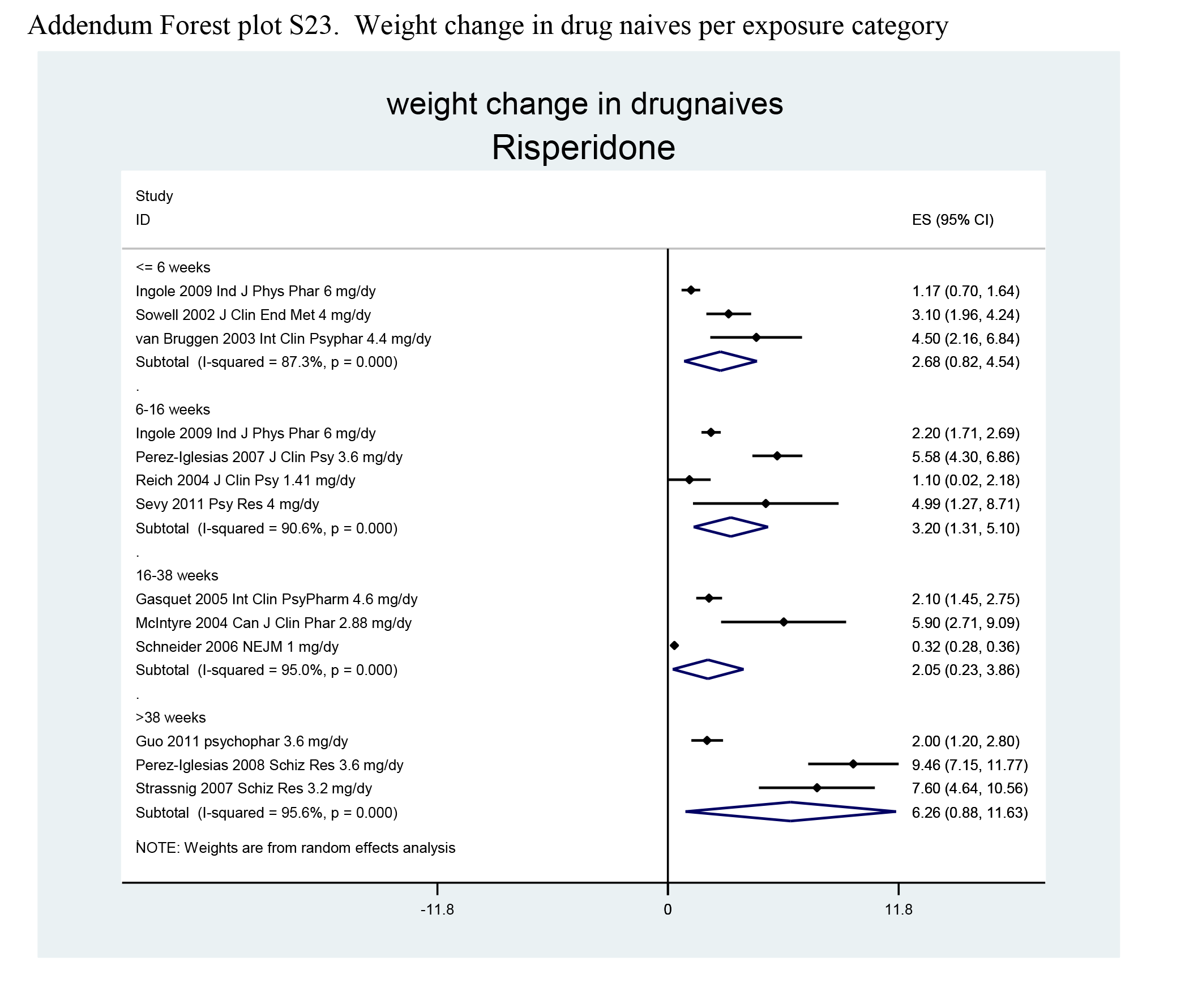

Supplement: File S3 — Forest plots S17–S24. Weight changes in AP naives per exposure category. (ZIP) [file pone.0094112.s004.zip › Risperidone Figure S23 Forest Plot.tif]

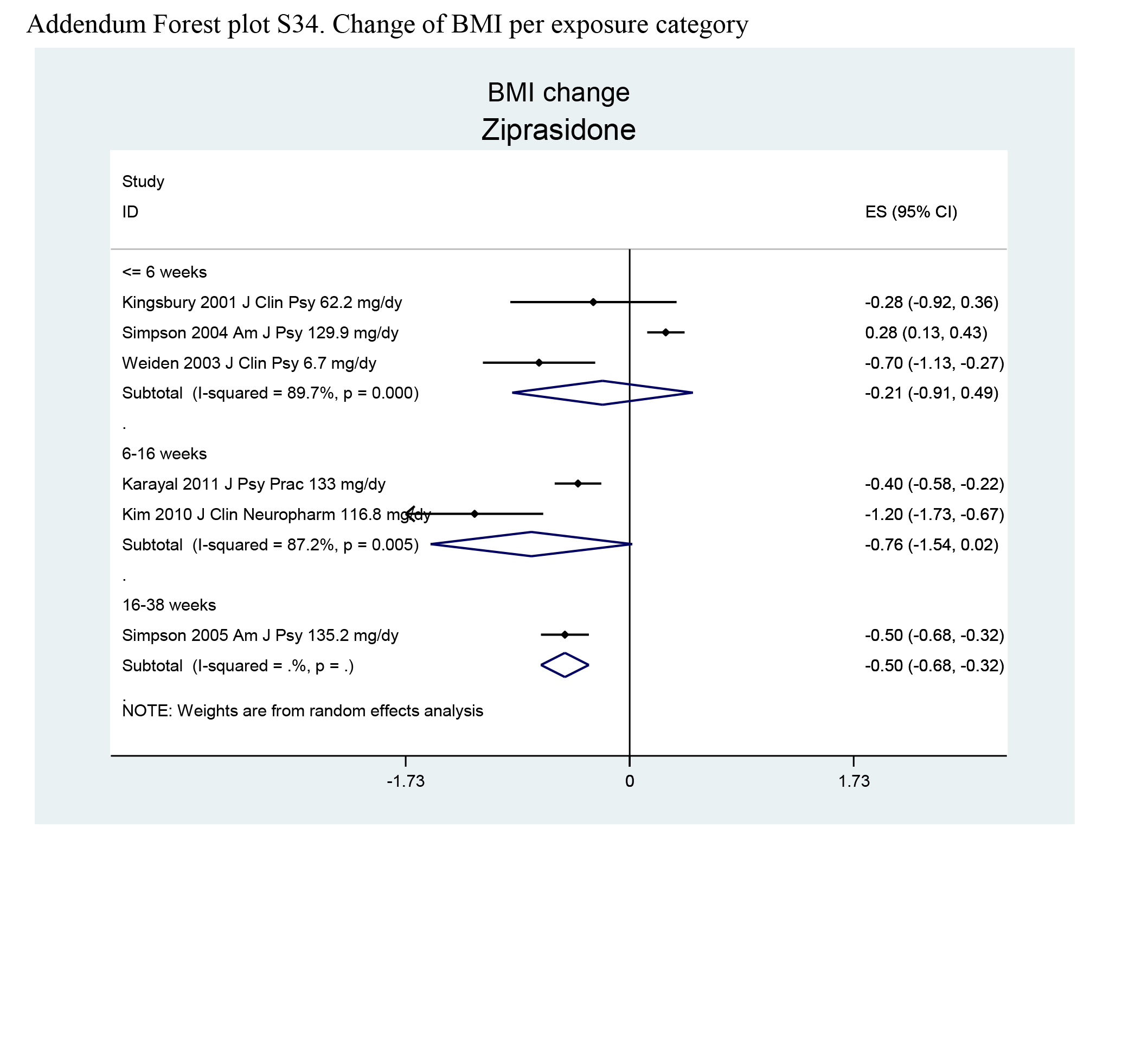

Supplement: File S4 — Forest Plots S25–S35. Change of BMI per exposure category. (ZIP) [file pone.0094112.s005.zip › Ziprasidone Figure Forest Plot.tif]

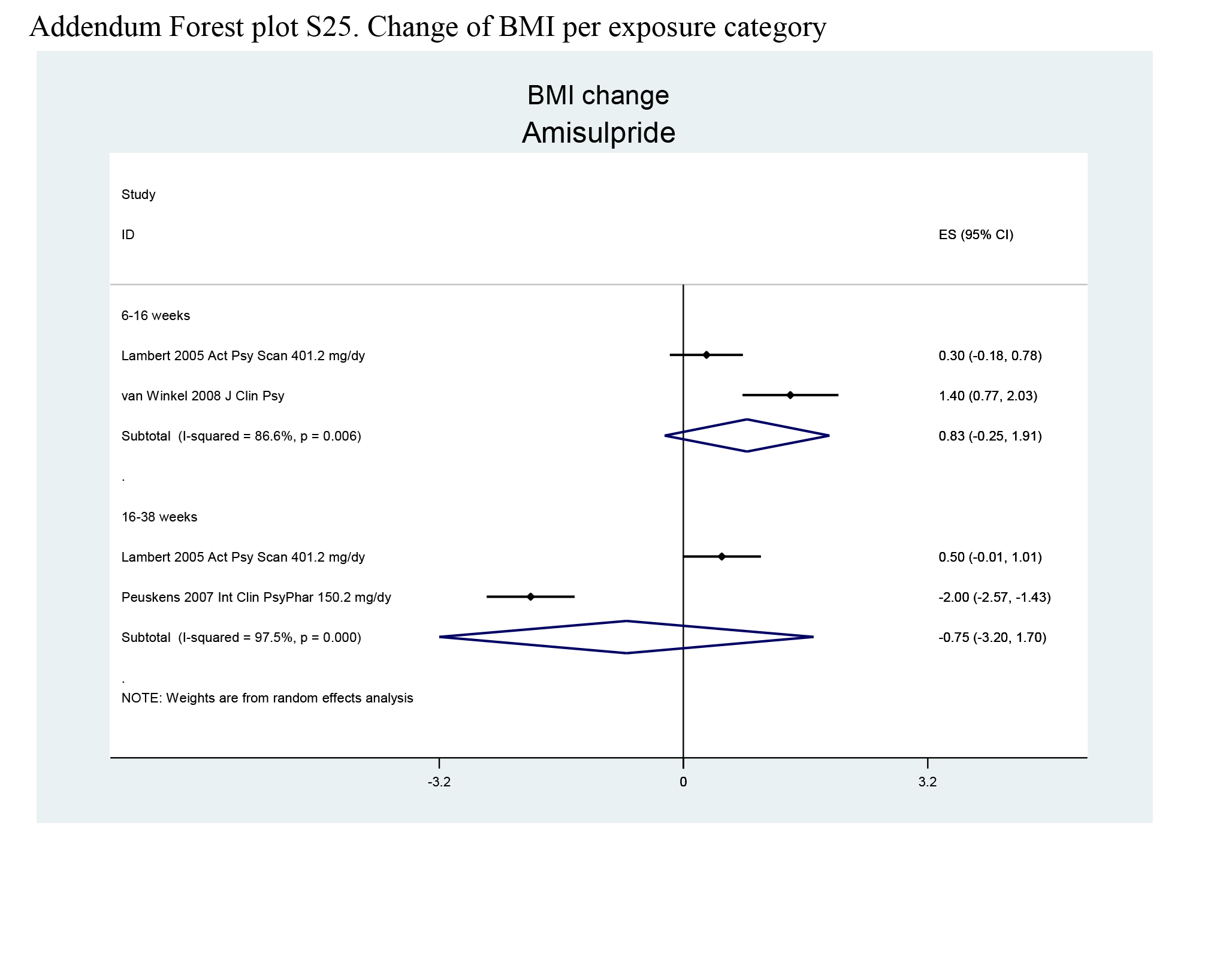

Supplement: File S4 — Forest Plots S25–S35. Change of BMI per exposure category. (ZIP) [file pone.0094112.s005.zip › Amisulpride Figure S25 Forest Plot.tif]

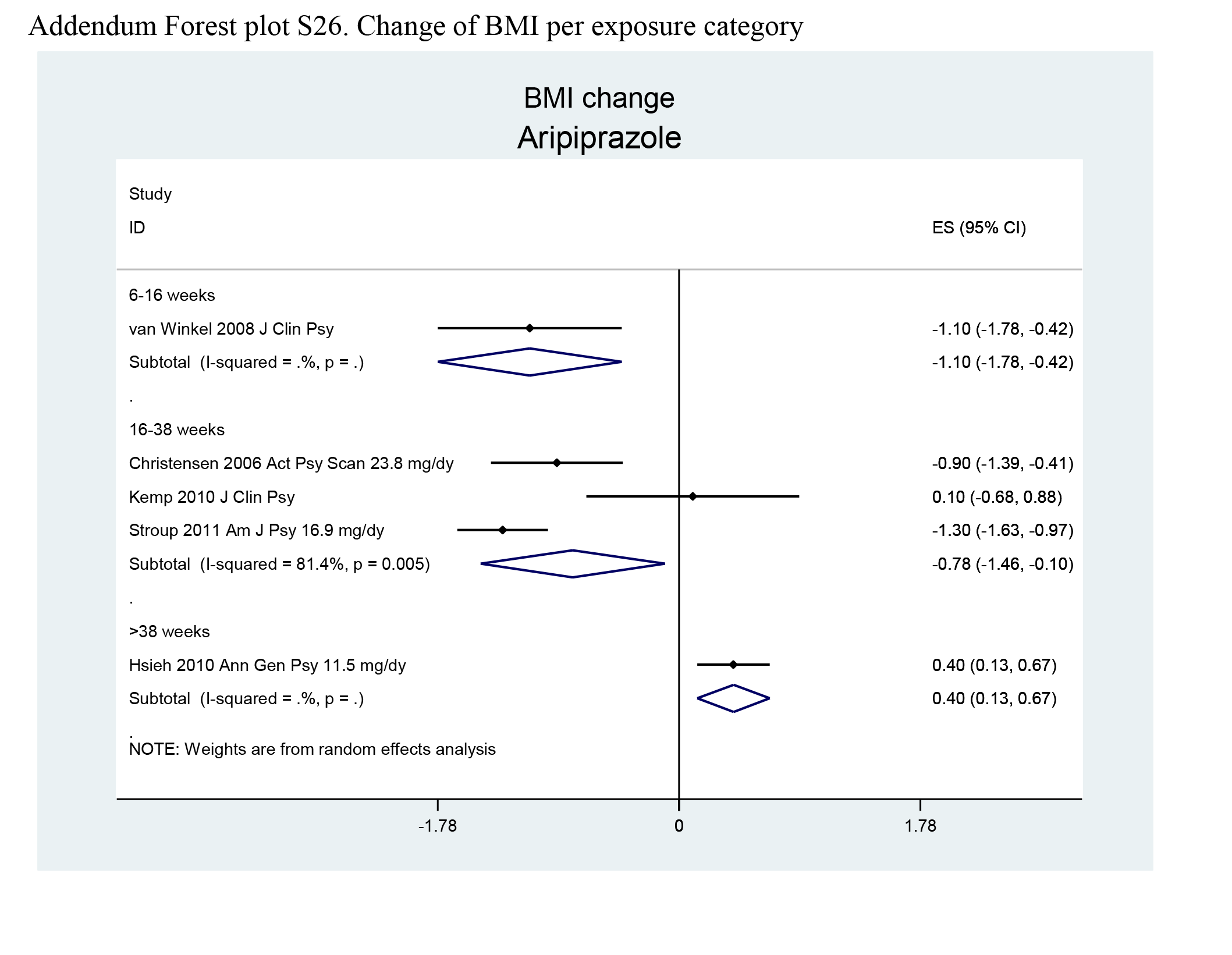

Supplement: File S4 — Forest Plots S25–S35. Change of BMI per exposure category. (ZIP) [file pone.0094112.s005.zip › Aripiprazole Figure S26 Forest Plot.tif]

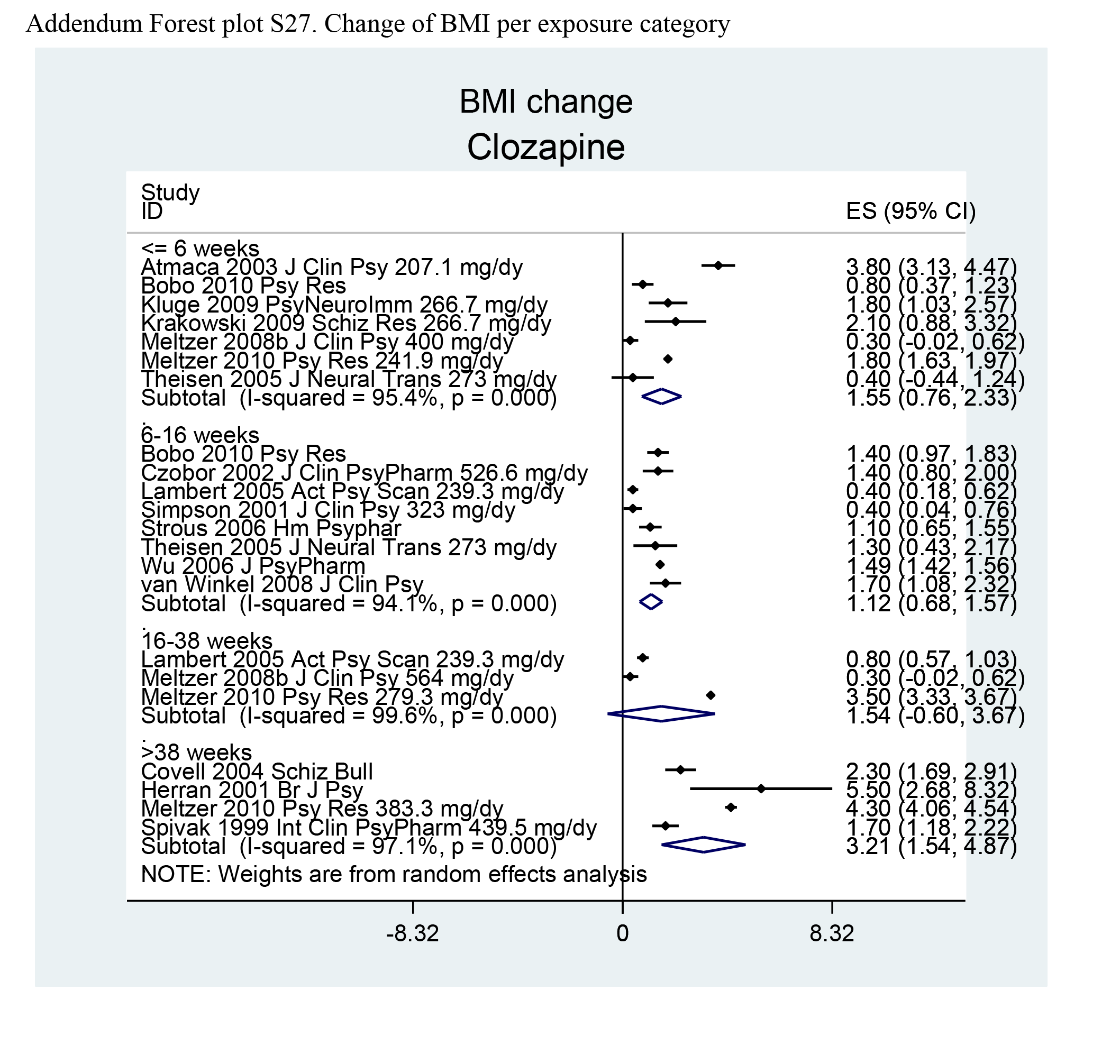

Supplement: File S4 — Forest Plots S25–S35. Change of BMI per exposure category. (ZIP) [file pone.0094112.s005.zip › Clozapine Figure Forest Plot.tif]

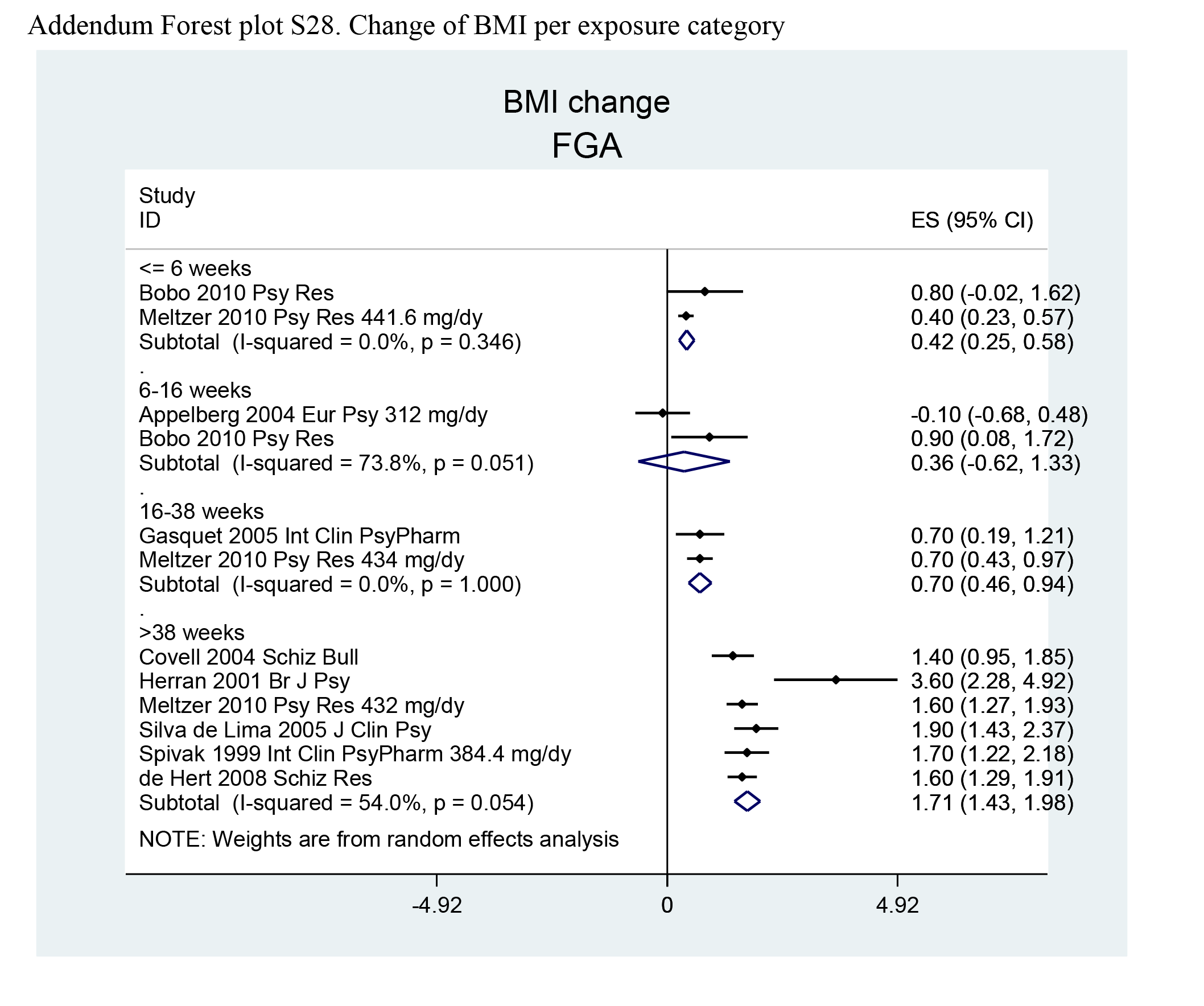

Supplement: File S4 — Forest Plots S25–S35. Change of BMI per exposure category. (ZIP) [file pone.0094112.s005.zip › FGA Figure S28 Forest Plot.tif]

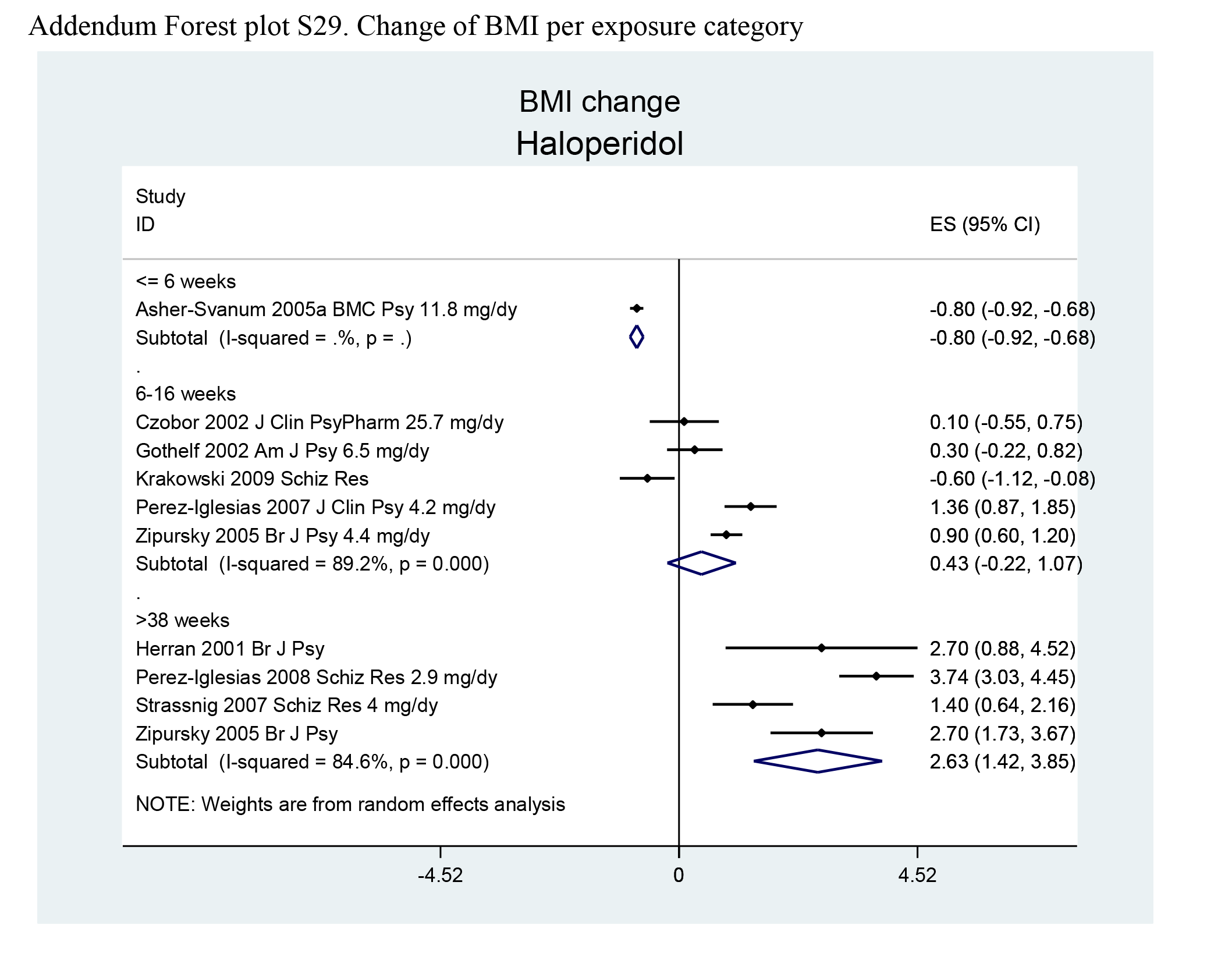

Supplement: File S4 — Forest Plots S25–S35. Change of BMI per exposure category. (ZIP) [file pone.0094112.s005.zip › Haloperidol Figure S29 Forest Plot.tif]

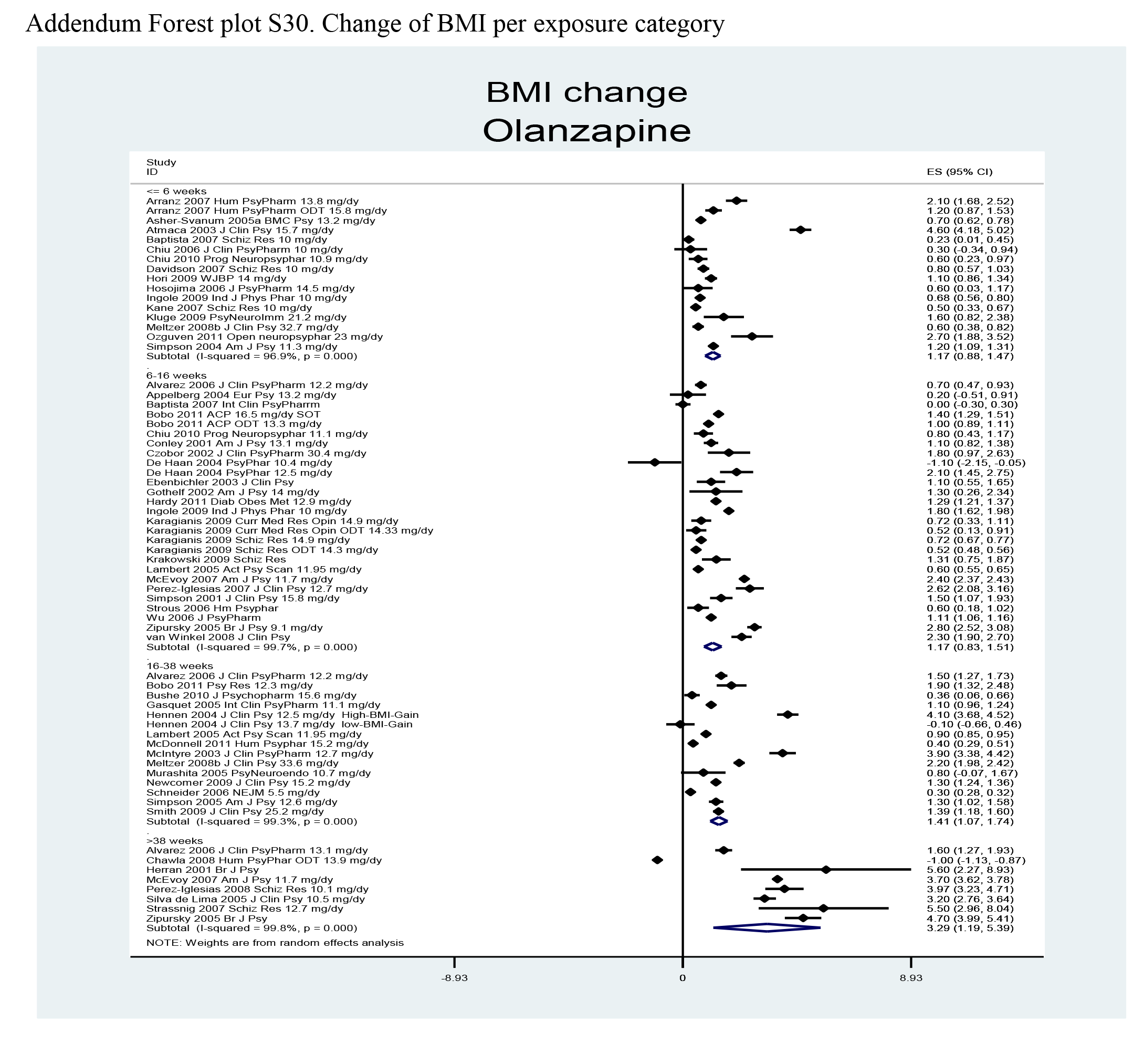

Supplement: File S4 — Forest Plots S25–S35. Change of BMI per exposure category. (ZIP) [file pone.0094112.s005.zip › Olanzapine Figure S30 Forest Plot.tif]

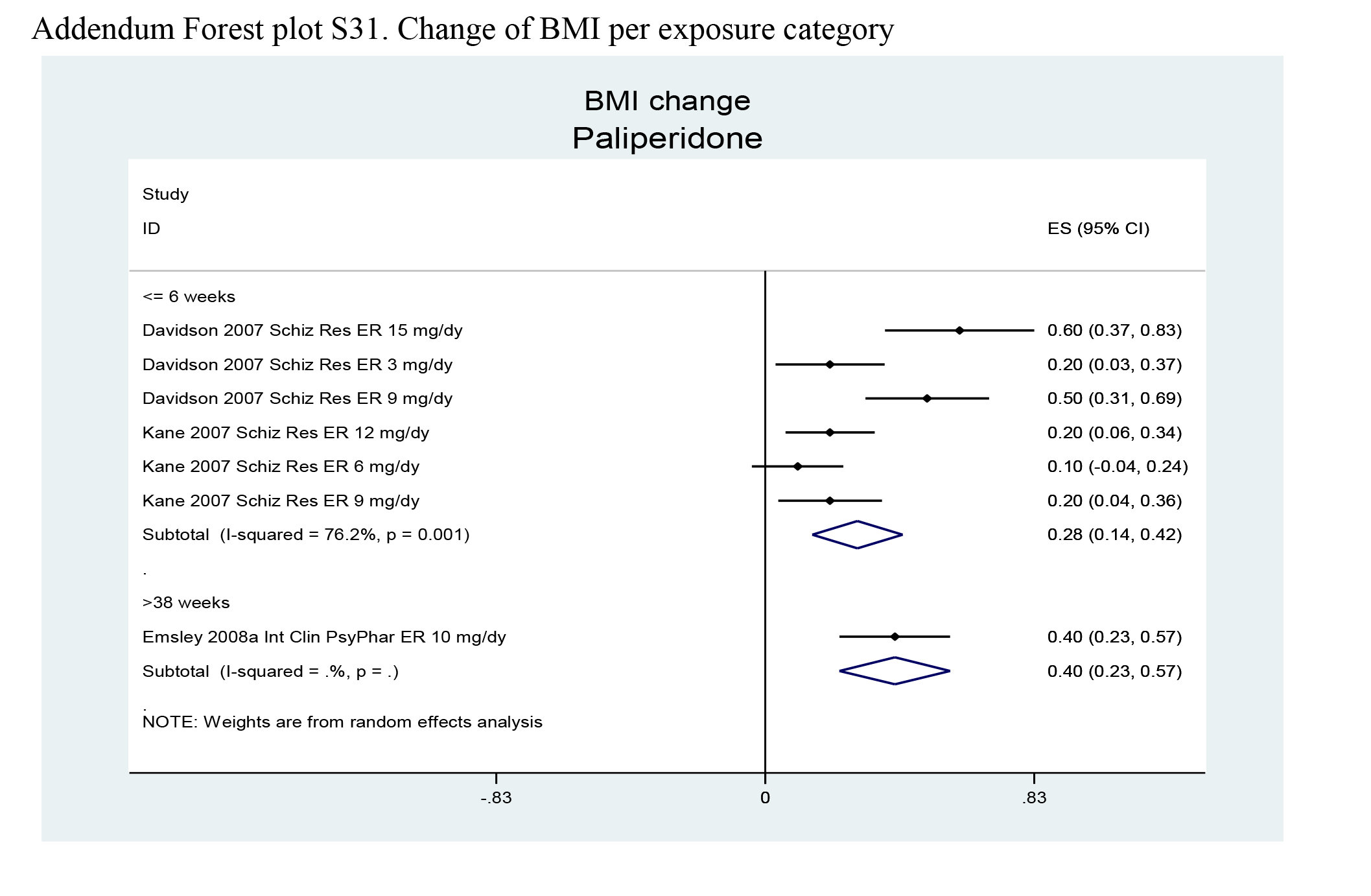

Supplement: File S4 — Forest Plots S25–S35. Change of BMI per exposure category. (ZIP) [file pone.0094112.s005.zip › Paliperidone Figure S31 Forest Plot.tif]

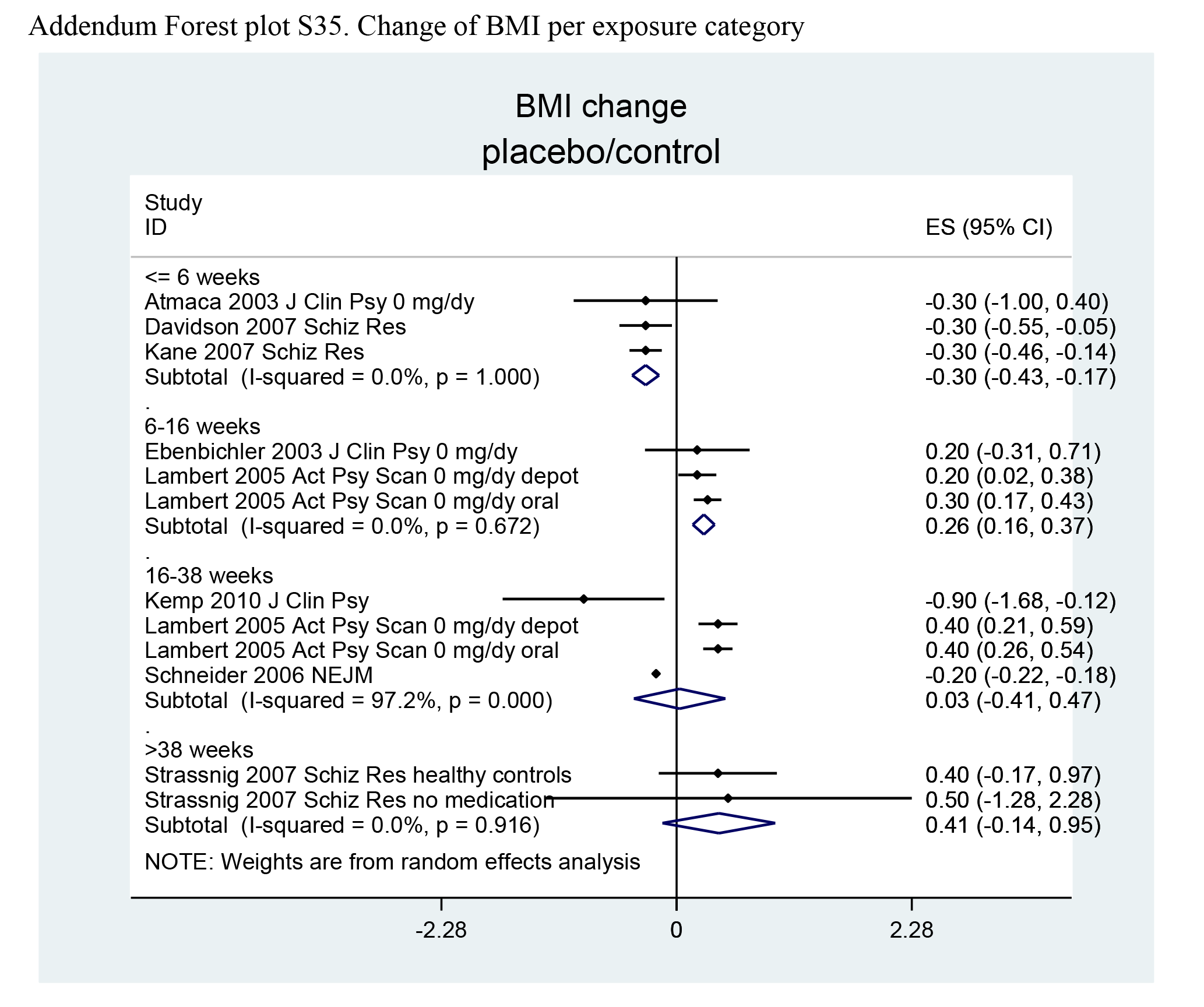

Supplement: File S4 — Forest Plots S25–S35. Change of BMI per exposure category. (ZIP) [file pone.0094112.s005.zip › Placebo Figure S35 Forest Plot.tif]

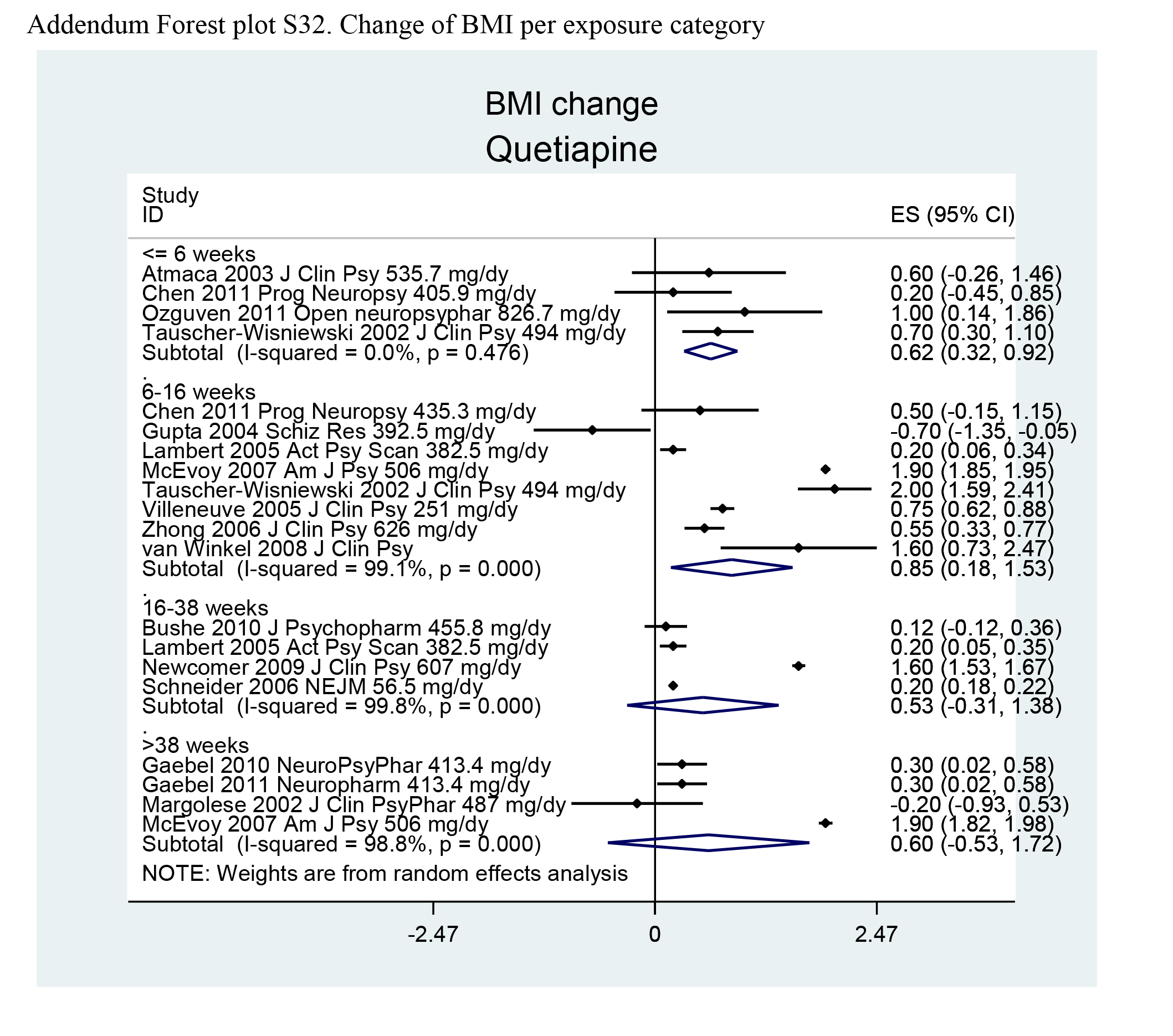

Supplement: File S4 — Forest Plots S25–S35. Change of BMI per exposure category. (ZIP) [file pone.0094112.s005.zip › Quetiapine Figure S32 Forest Plot.tif]

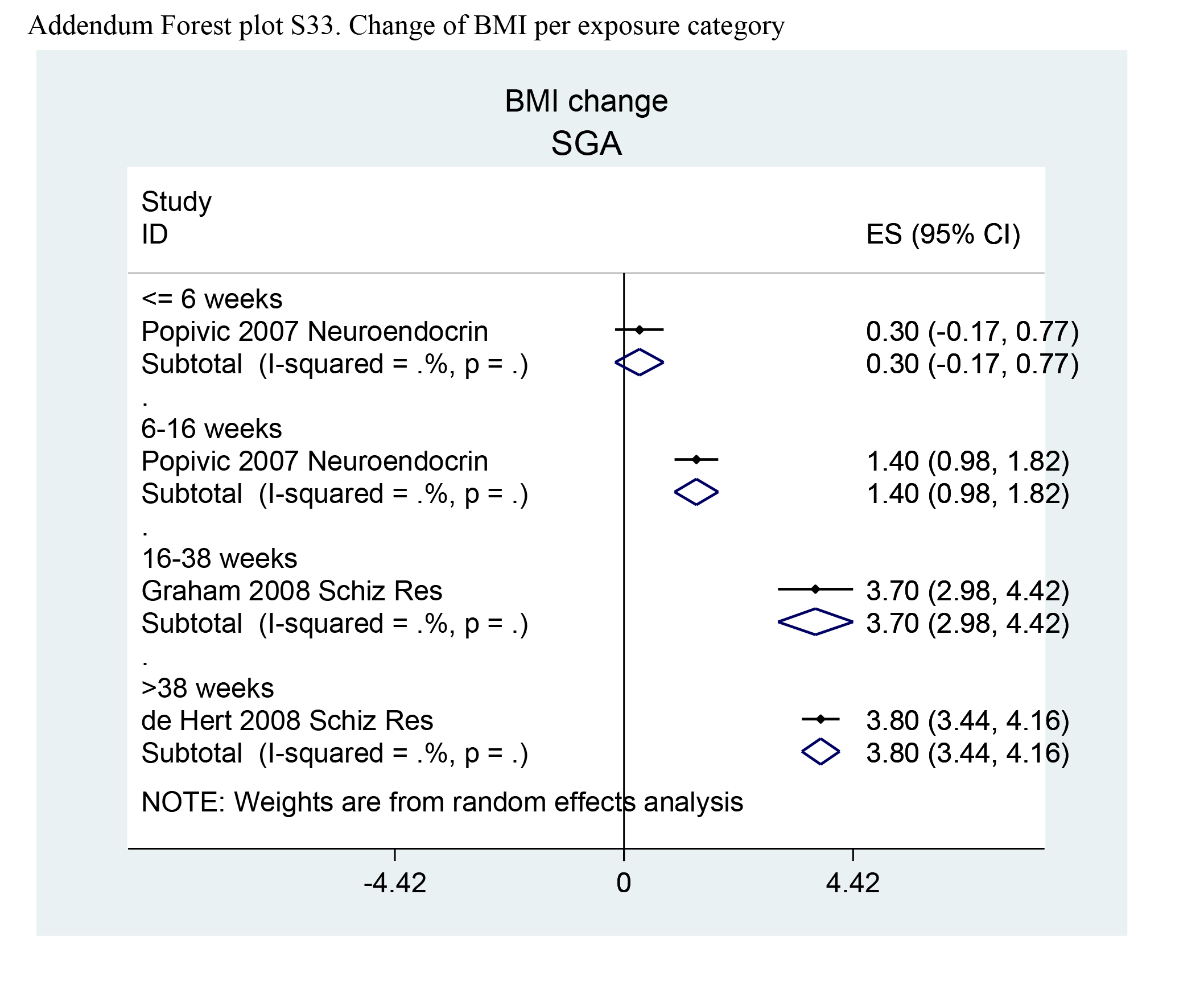

Supplement: File S4 — Forest Plots S25–S35. Change of BMI per exposure category. (ZIP) [file pone.0094112.s005.zip › SGA Figure S33 Forest Plot.tif]

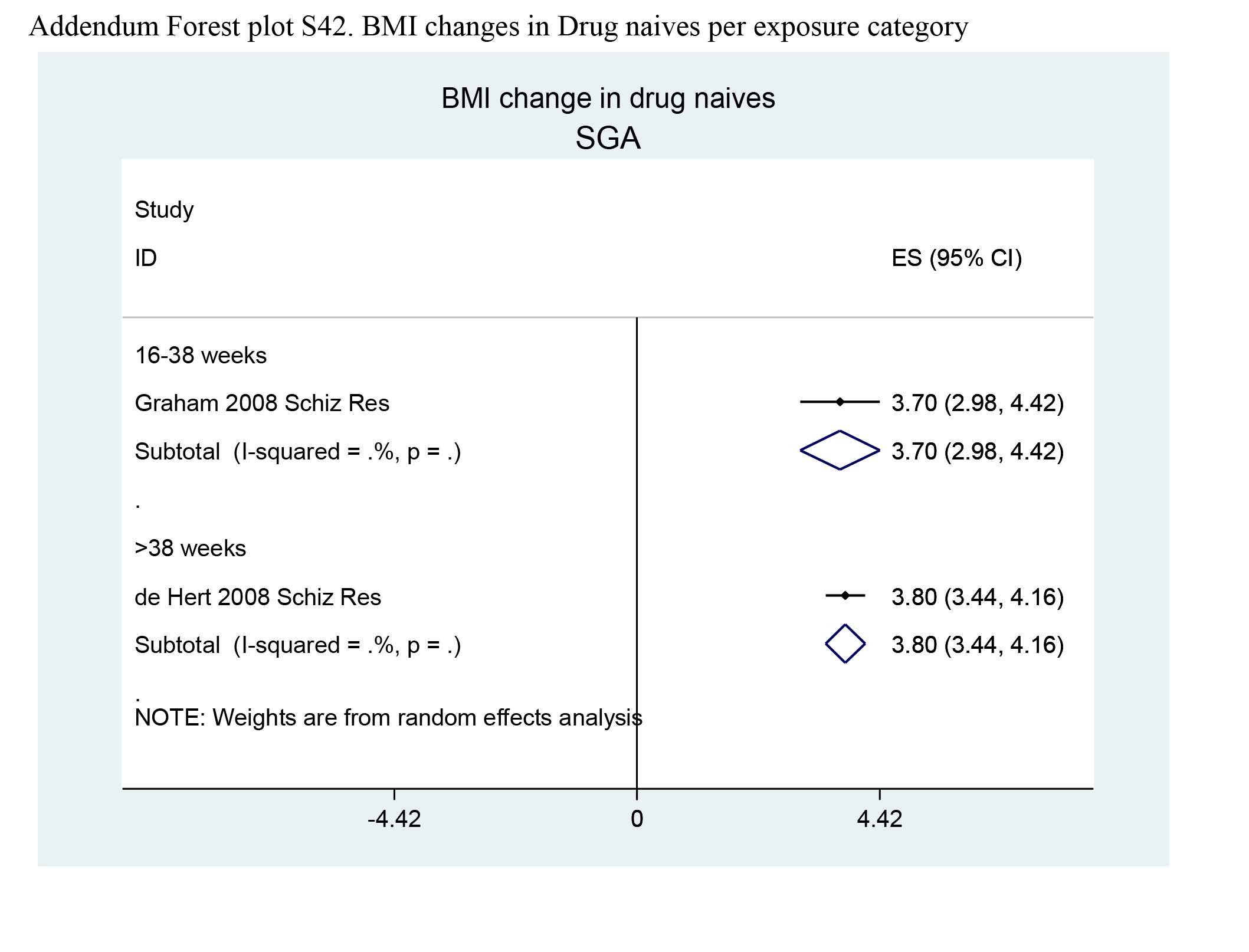

Supplement: File S5 — Forest Plots S36–S43. Changes of BMI in AP naives per exposure category. (ZIP) [file pone.0094112.s006.zip › SGA Figure S42 Forest Plot.tif]

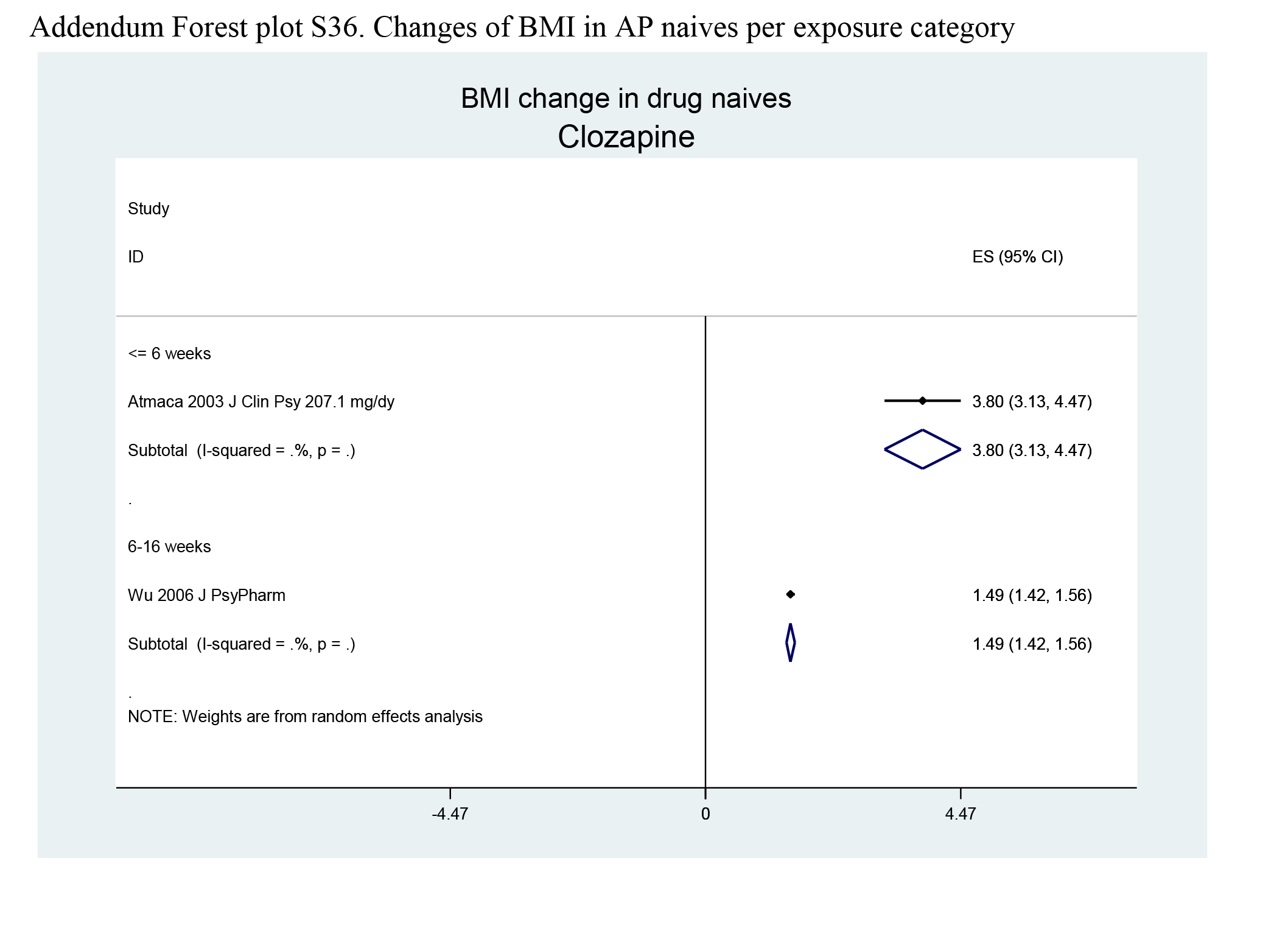

Supplement: File S5 — Forest Plots S36–S43. Changes of BMI in AP naives per exposure category. (ZIP) [file pone.0094112.s006.zip › Clozapine Figure S36 Forest Plot.tif]

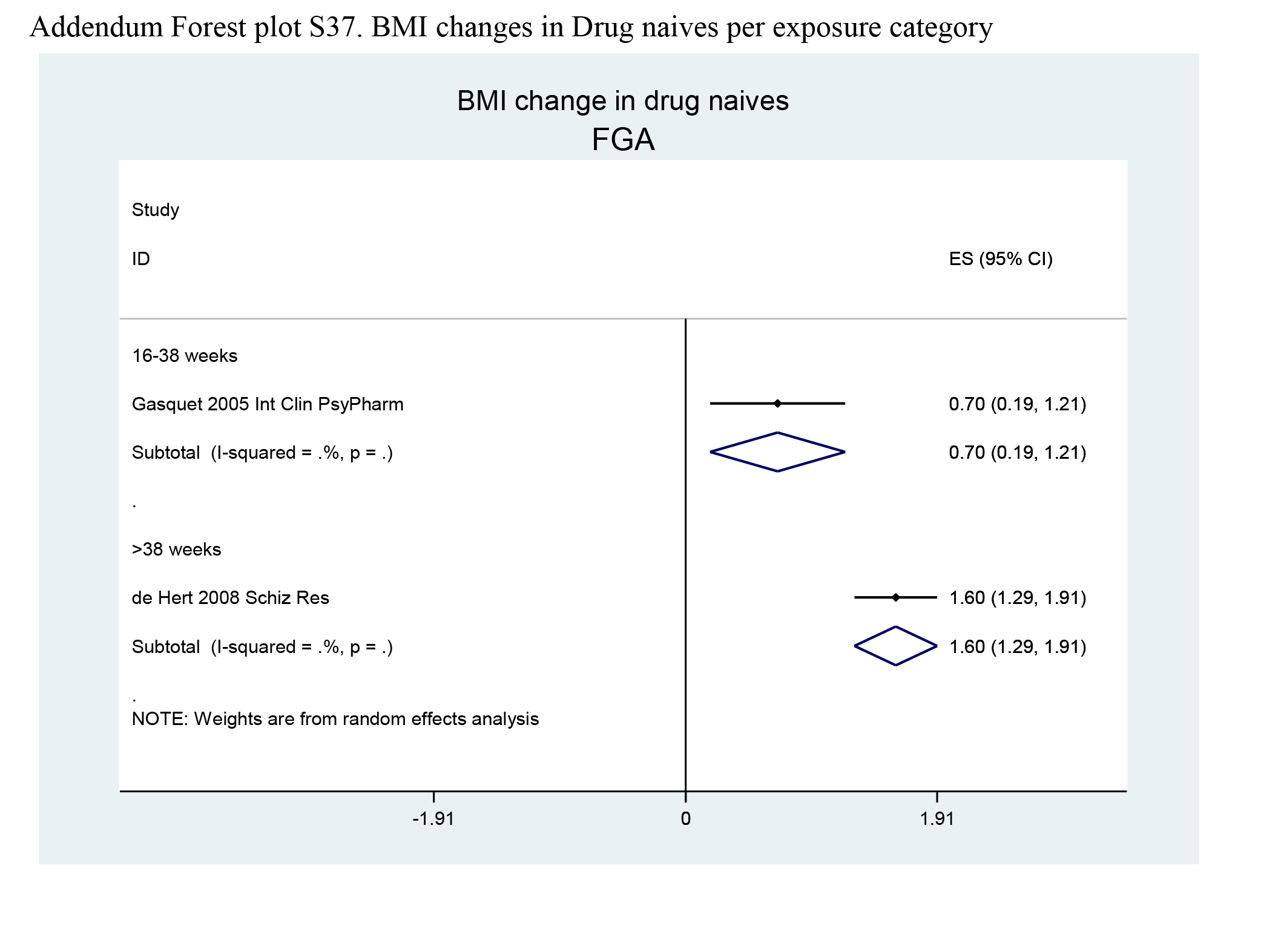

Supplement: File S5 — Forest Plots S36–S43. Changes of BMI in AP naives per exposure category. (ZIP) [file pone.0094112.s006.zip › FGA Figure S37 Forest Plot.tif]

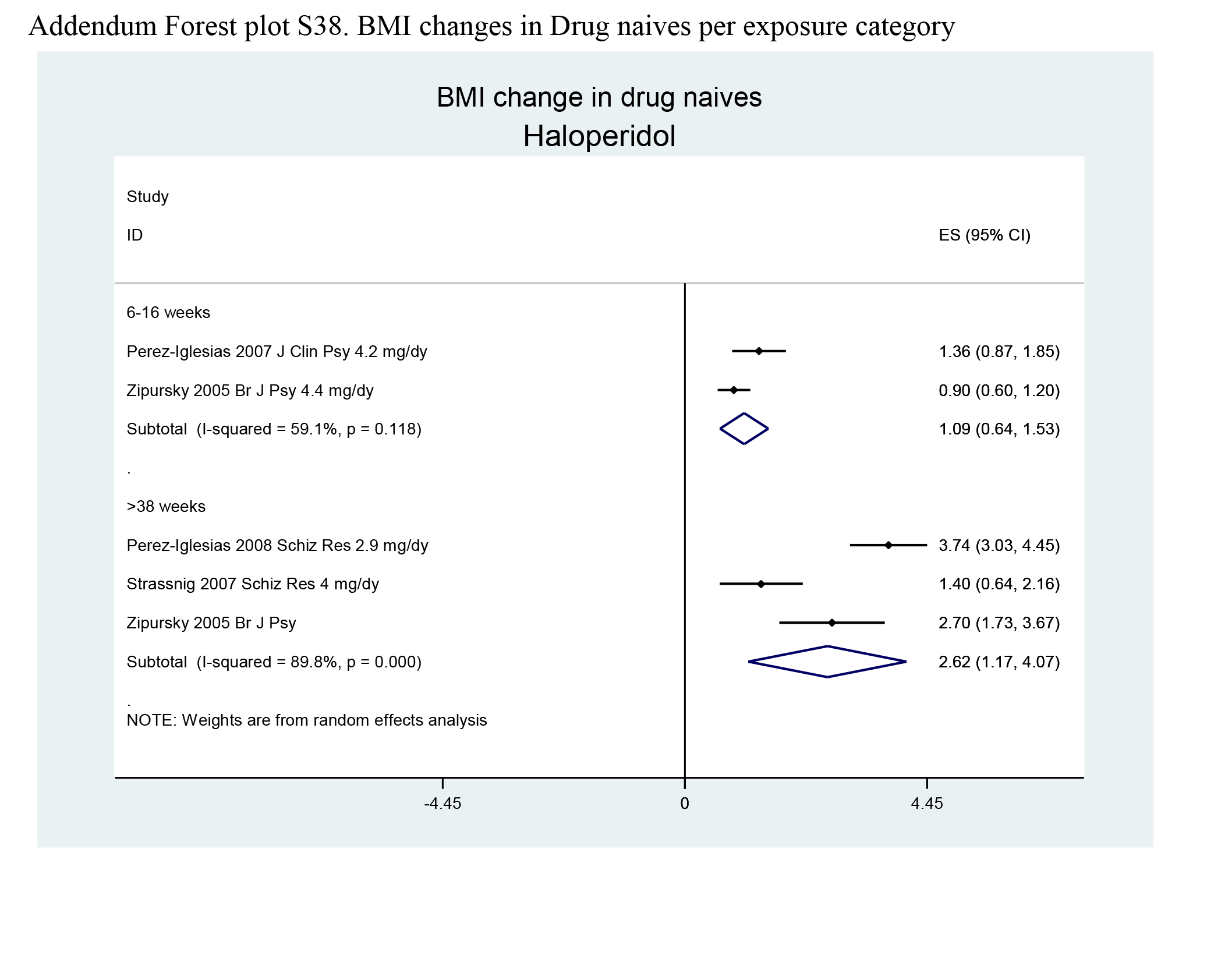

Supplement: File S5 — Forest Plots S36–S43. Changes of BMI in AP naives per exposure category. (ZIP) [file pone.0094112.s006.zip › Haloperidol Figure S38 Forest Plot.tif]

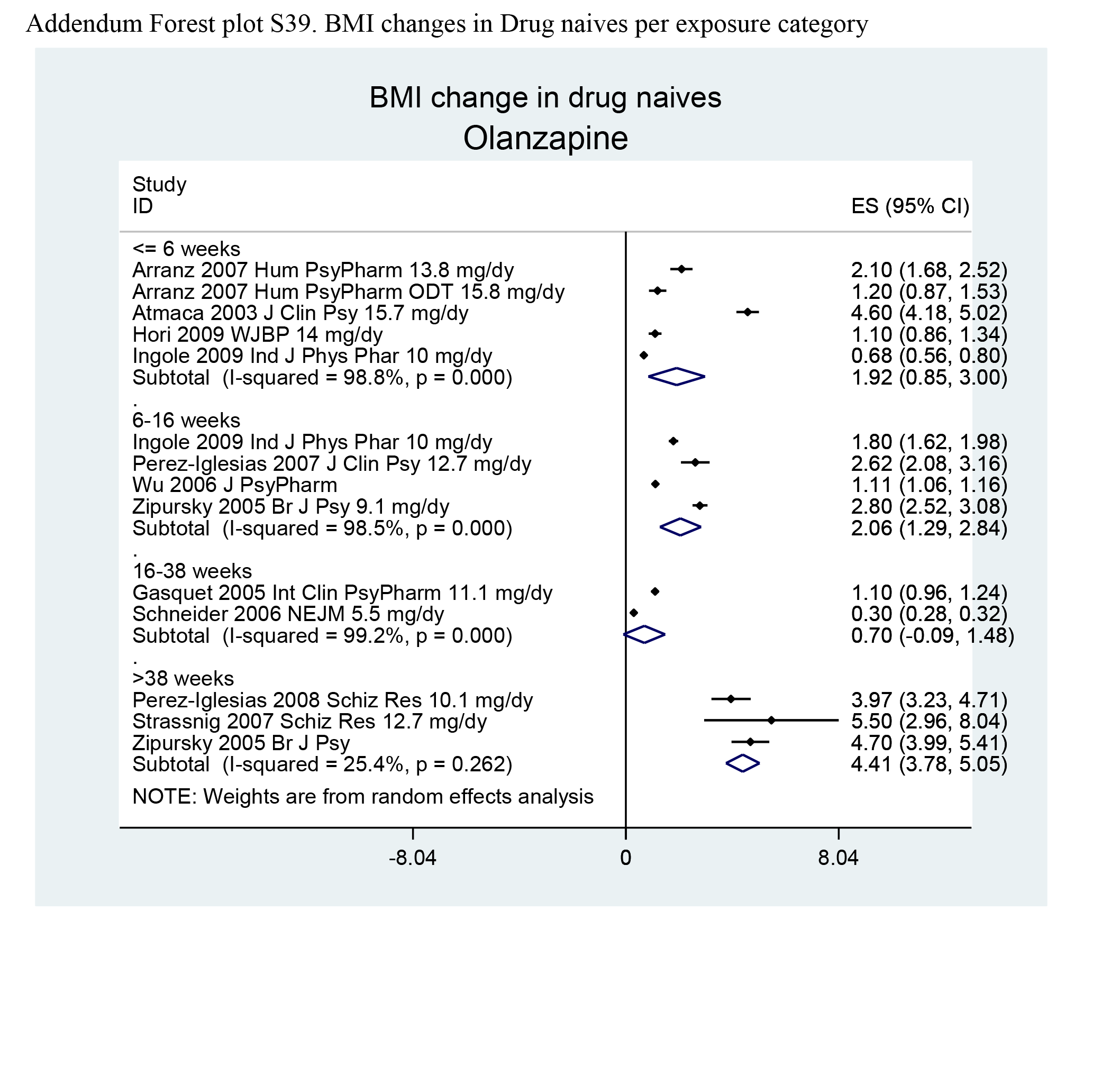

Supplement: File S5 — Forest Plots S36–S43. Changes of BMI in AP naives per exposure category. (ZIP) [file pone.0094112.s006.zip › Olanzapine Figure S39 Forest Plot.tif]

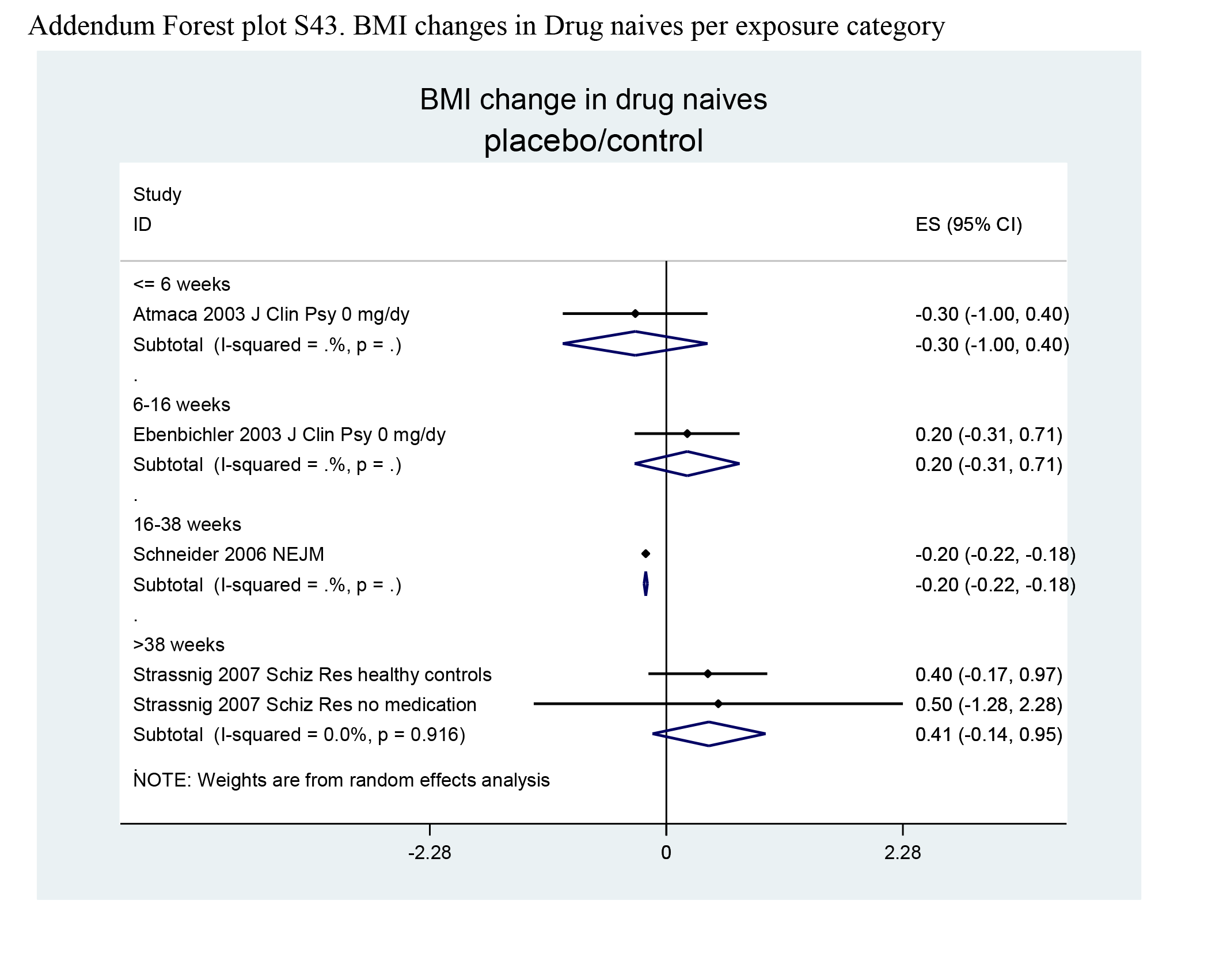

Supplement: File S5 — Forest Plots S36–S43. Changes of BMI in AP naives per exposure category. (ZIP) [file pone.0094112.s006.zip › Placebo Figure S43 Forest Plot.tif]

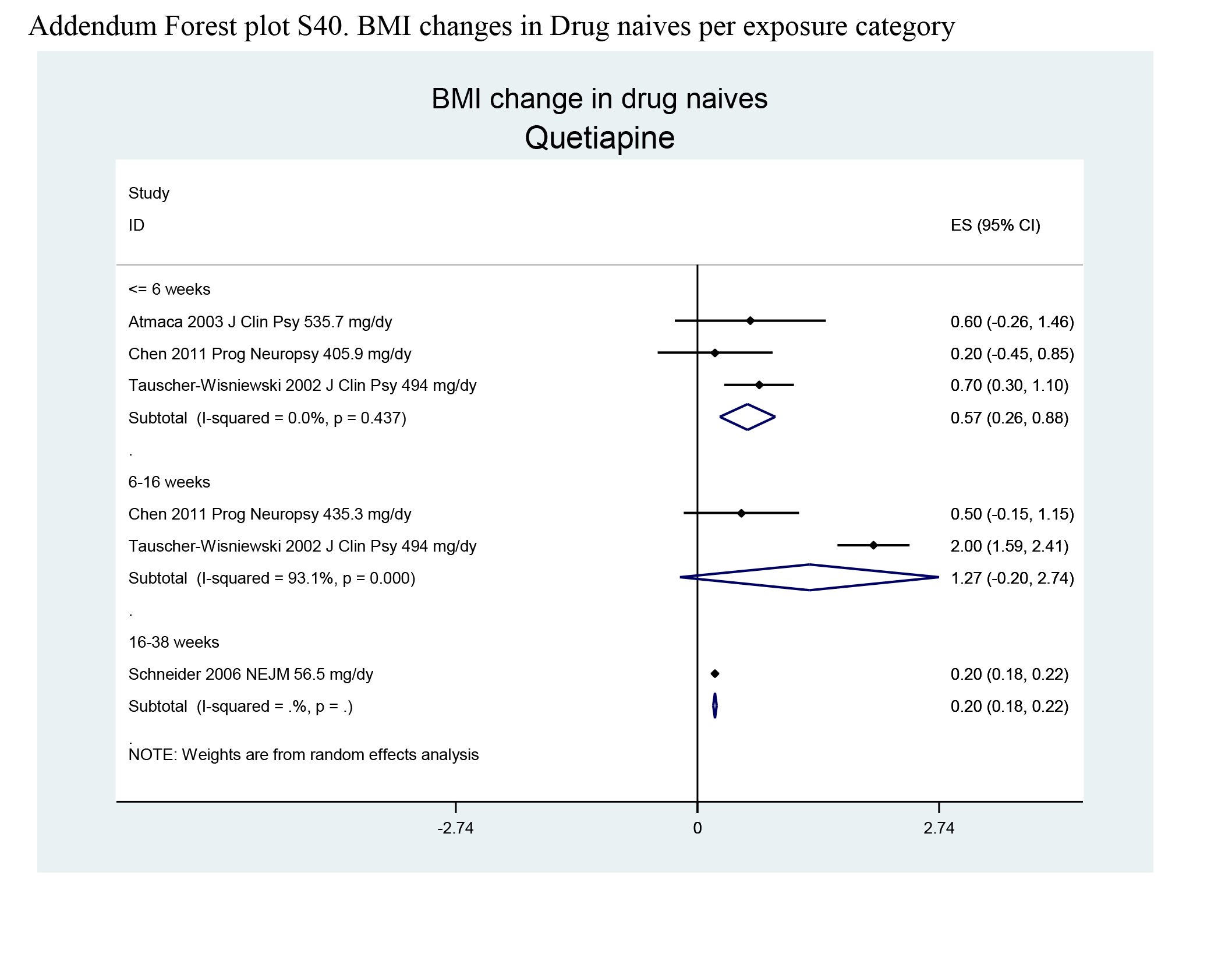

Supplement: File S5 — Forest Plots S36–S43. Changes of BMI in AP naives per exposure category. (ZIP) [file pone.0094112.s006.zip › Quetiapine Figure Forest Plot.tif]

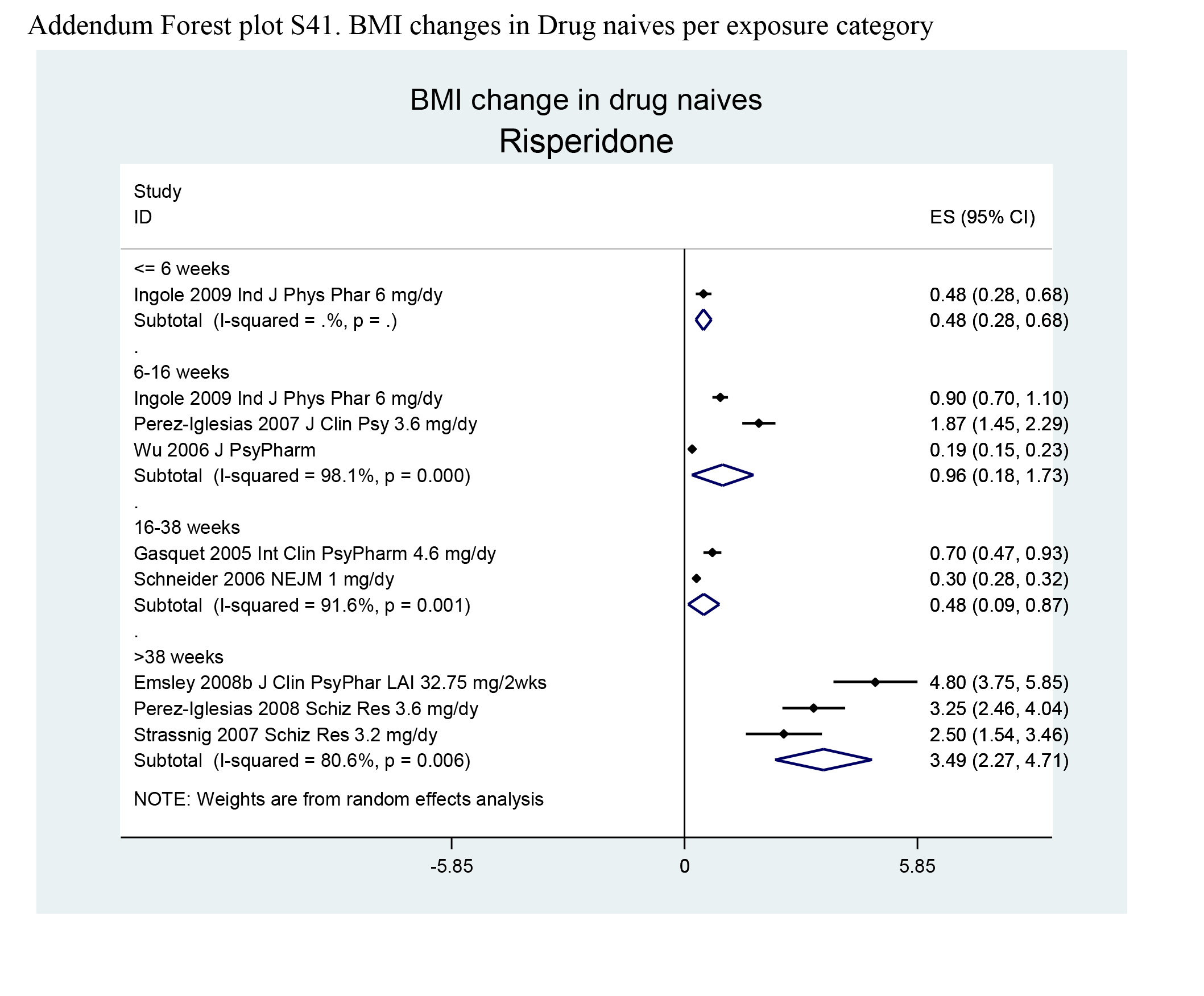

Supplement: File S5 — Forest Plots S36–S43. Changes of BMI in AP naives per exposure category. (ZIP) [file pone.0094112.s006.zip › Risperidone Figure S41 Forest Plot.tif]

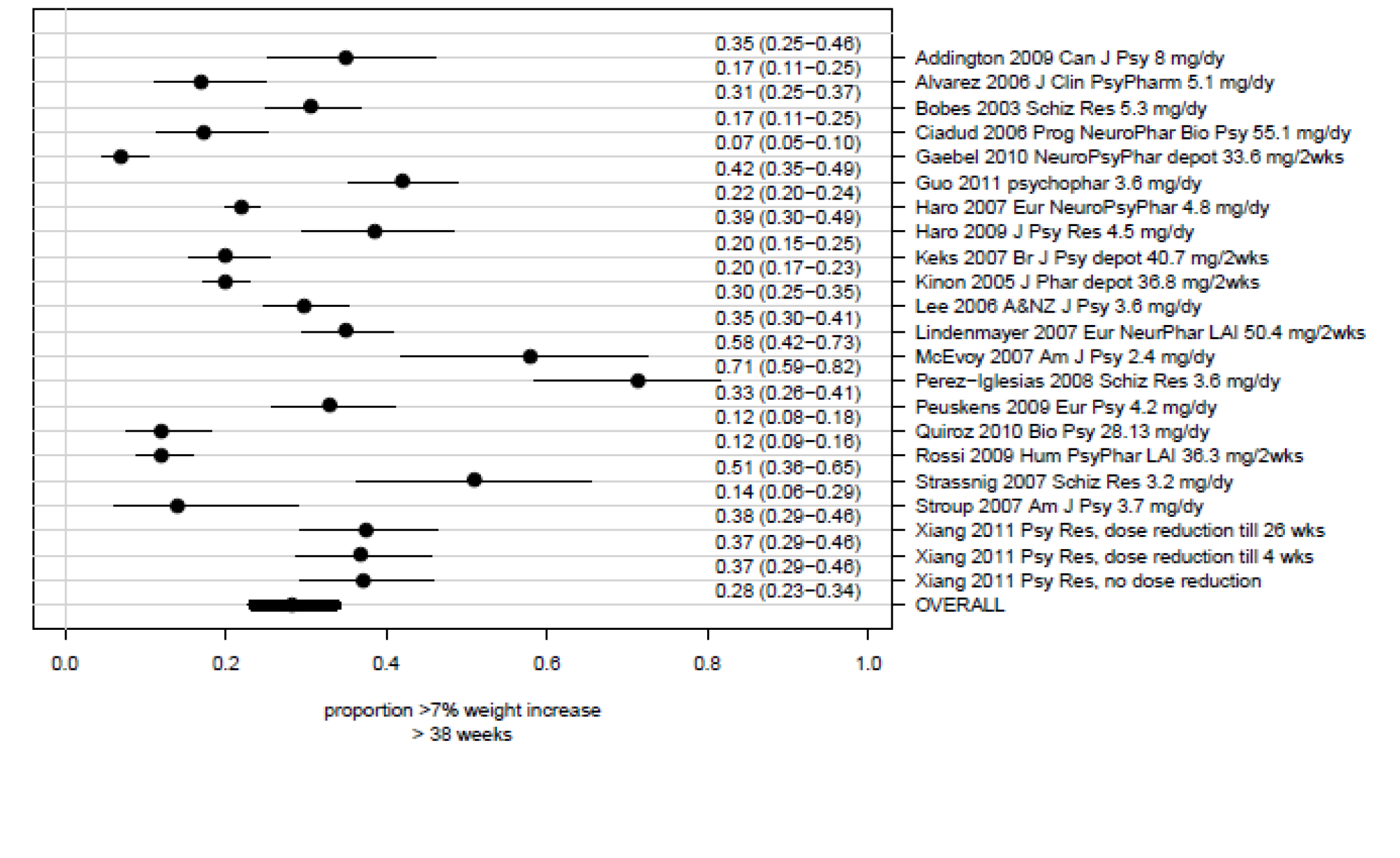

Supplement: File S6 — Forest Plots S44–S51d - Proportion (7%) of weight gain per exposure category. (ZIP) [file pone.0094112.s007.zip › Olanzapine Figure S51d Forest Plot.tif]

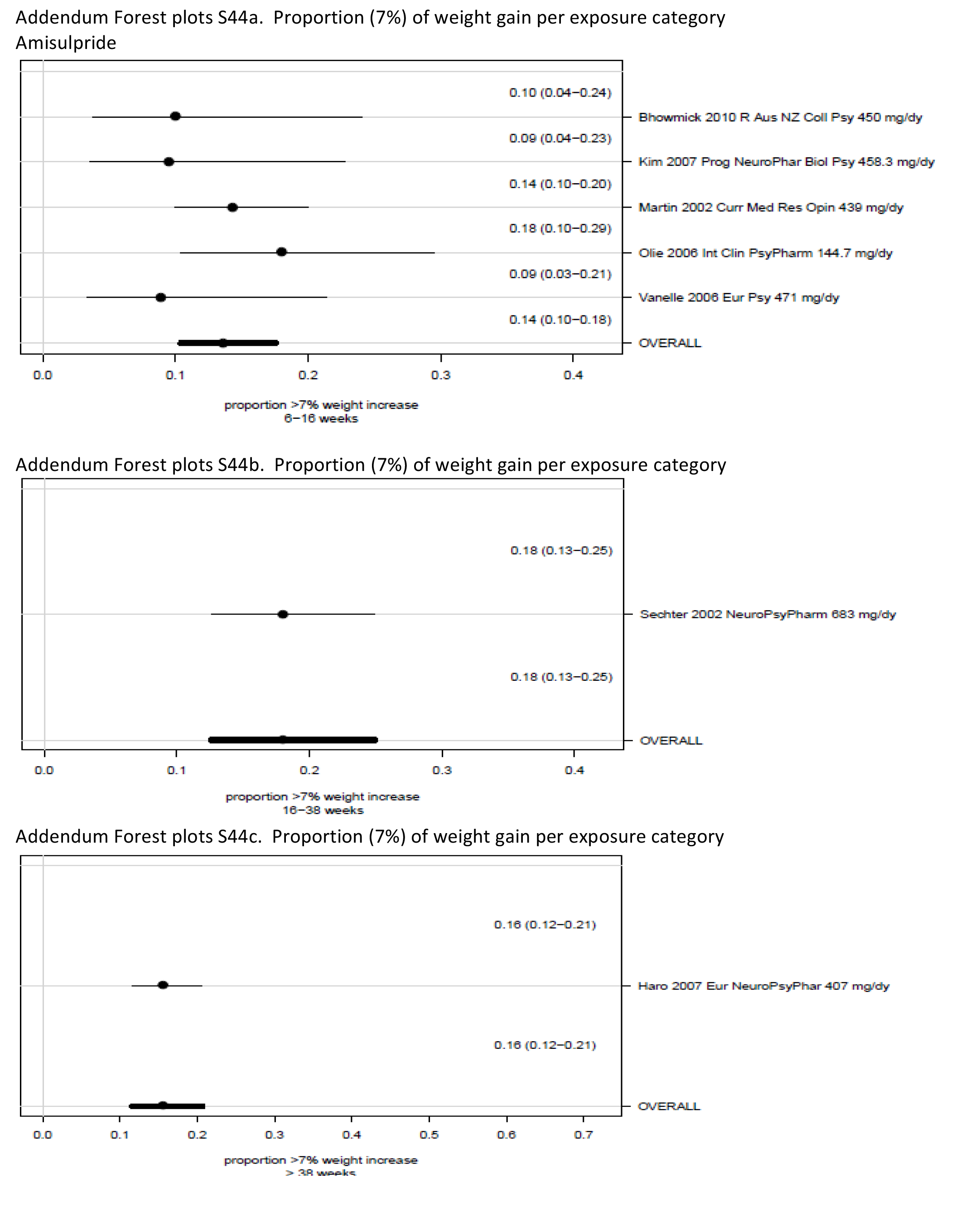

Supplement: File S6 — Forest Plots S44–S51d - Proportion (7%) of weight gain per exposure category. (ZIP) [file pone.0094112.s007.zip › Amisulpride Figure S44 Forest Plot.tif]

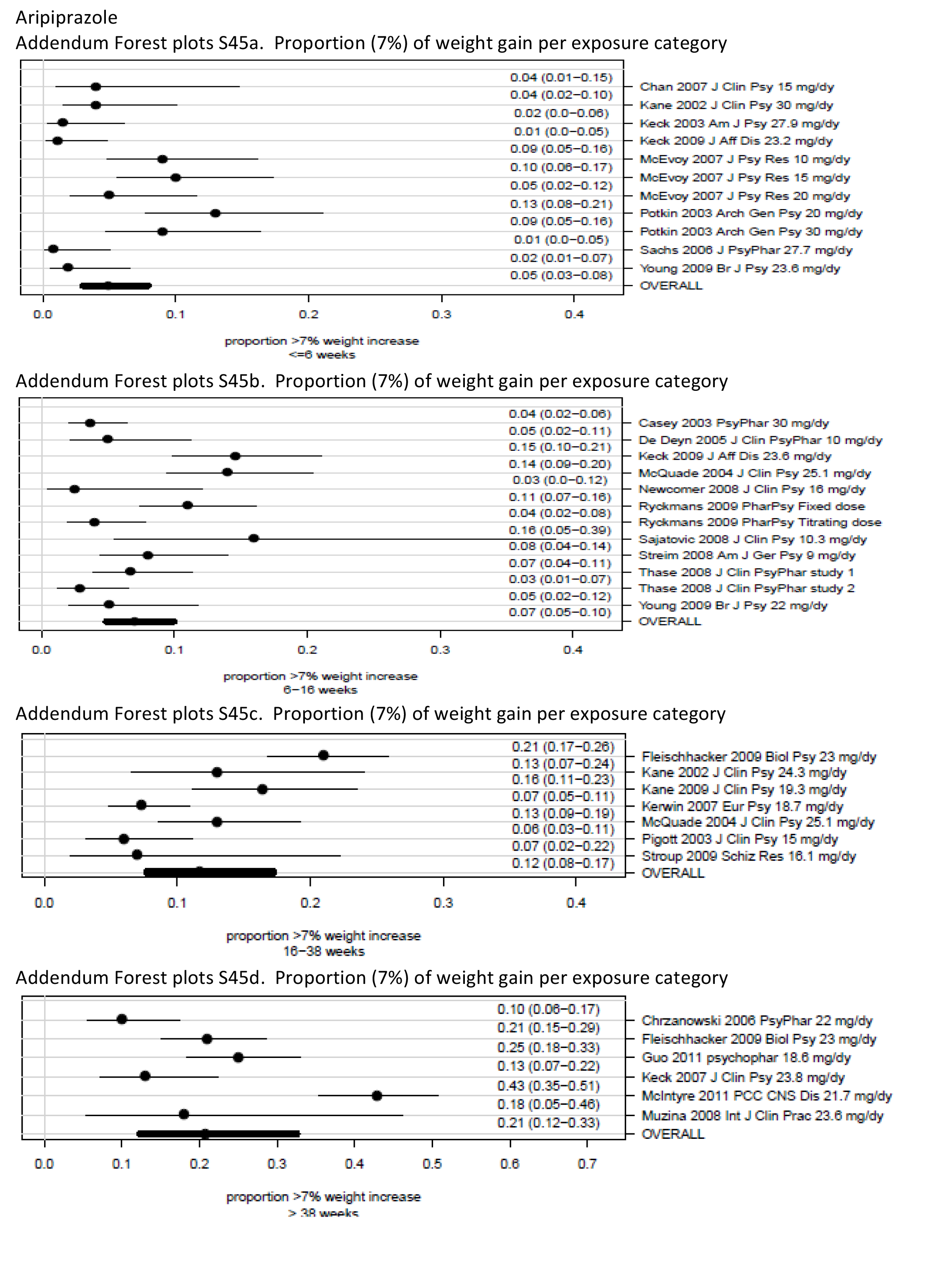

Supplement: File S6 — Forest Plots S44–S51d - Proportion (7%) of weight gain per exposure category. (ZIP) [file pone.0094112.s007.zip › Aripirazole Figure S45 Forest Plot.tif]

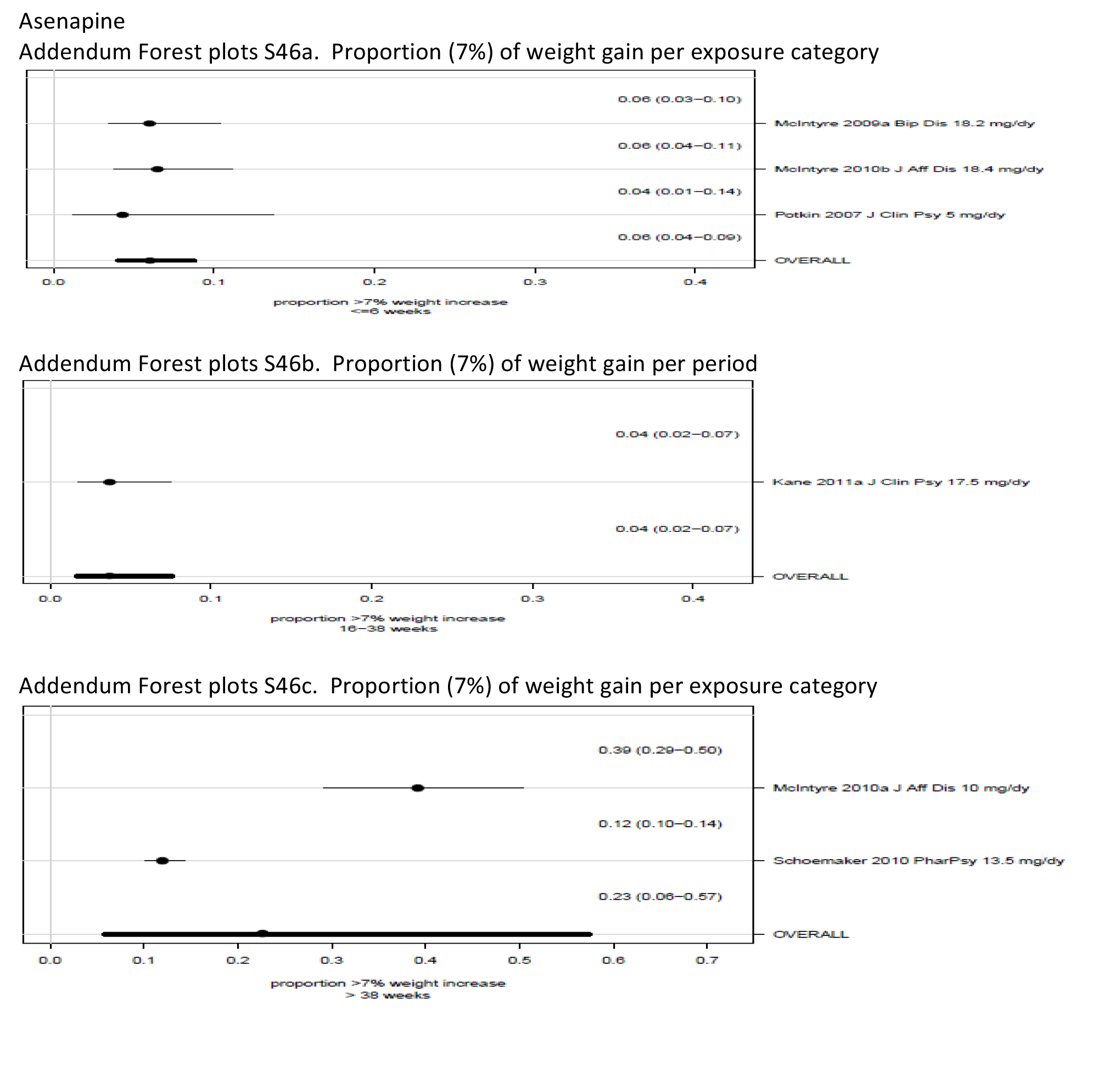

Supplement: File S6 — Forest Plots S44–S51d - Proportion (7%) of weight gain per exposure category. (ZIP) [file pone.0094112.s007.zip › Asenapine Figure S46 Forest Plot.tif]

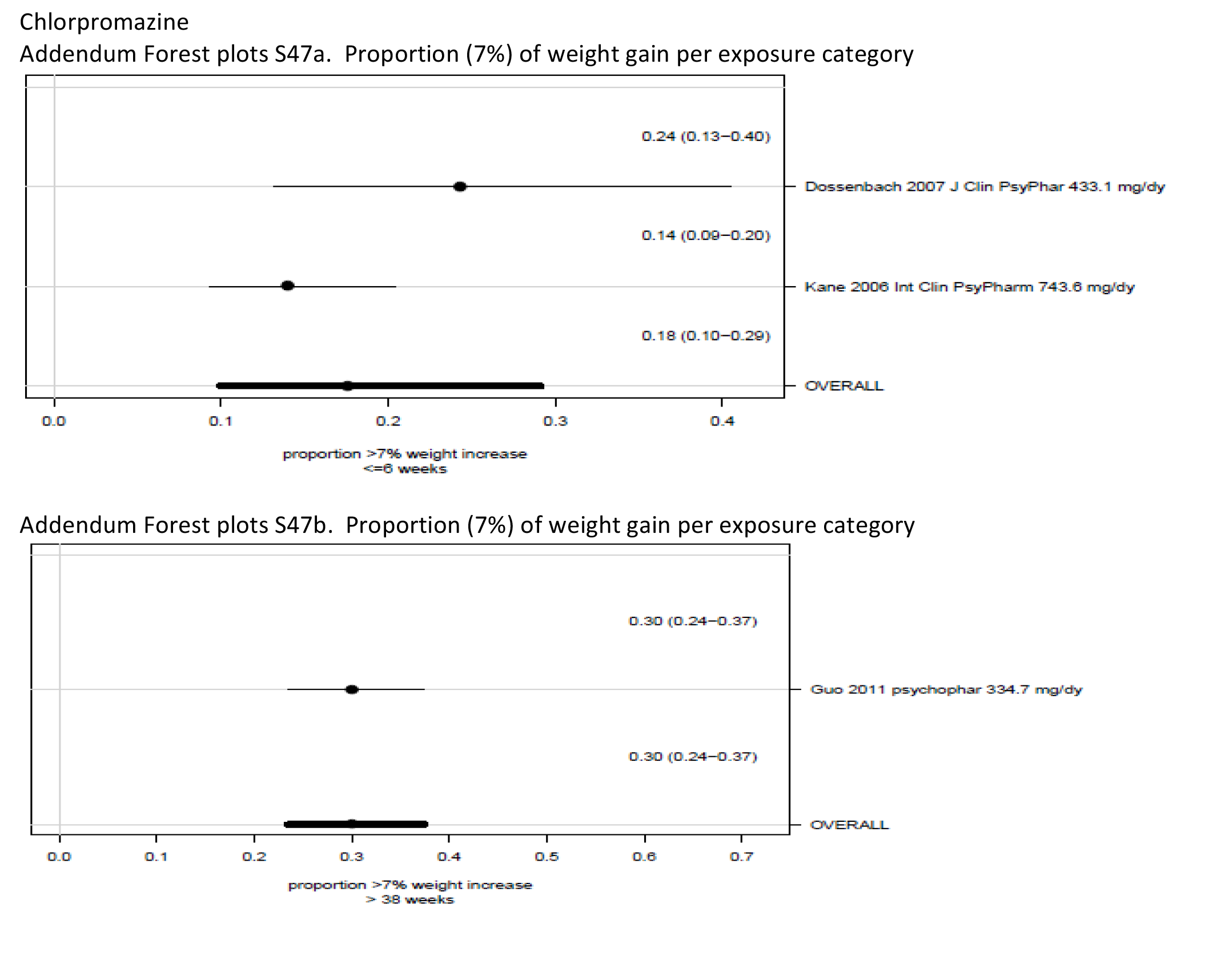

Supplement: File S6 — Forest Plots S44–S51d - Proportion (7%) of weight gain per exposure category. (ZIP) [file pone.0094112.s007.zip › Chlorpromazine Figure S47 Forest Plot.tif]

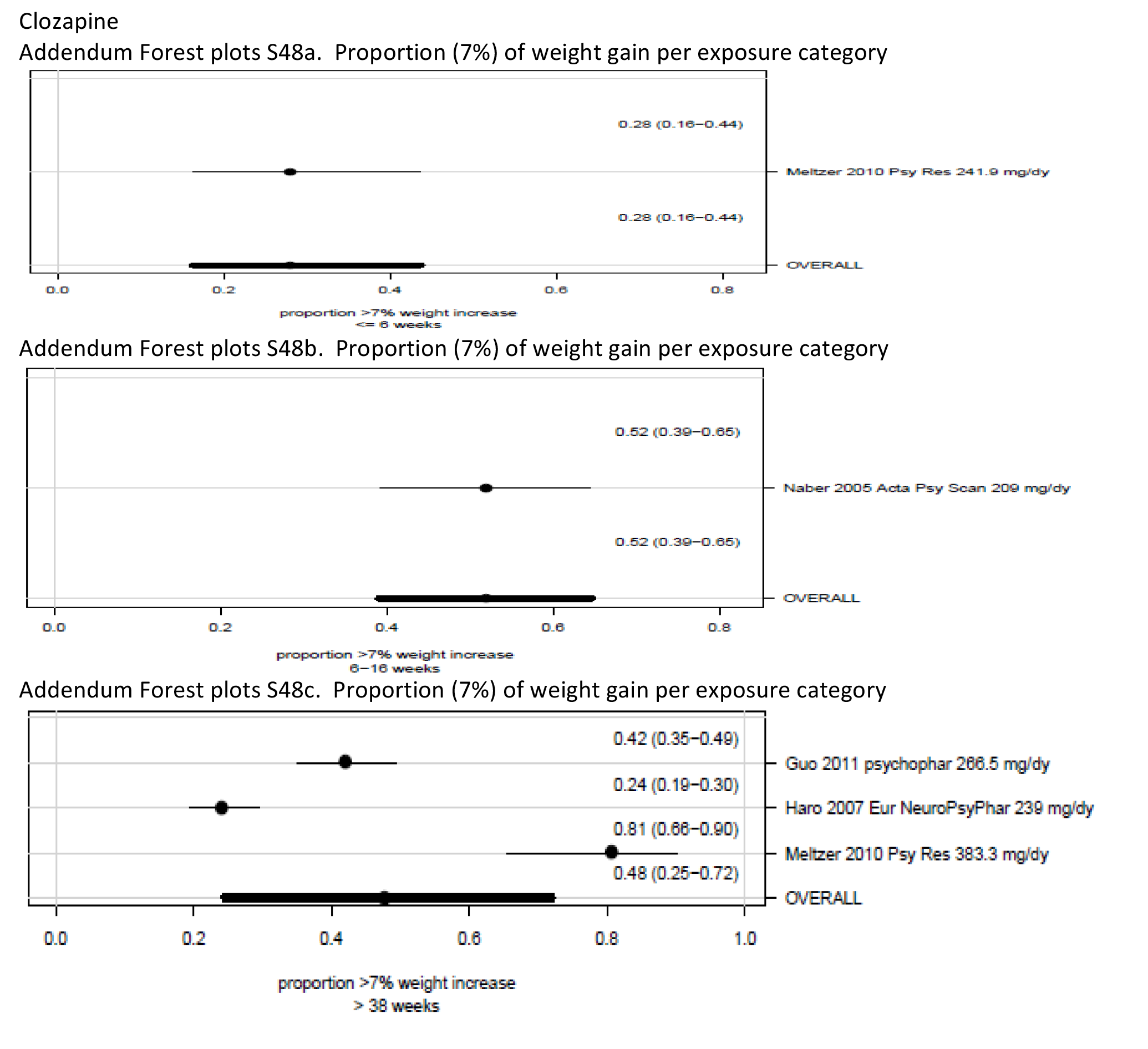

Supplement: File S6 — Forest Plots S44–S51d - Proportion (7%) of weight gain per exposure category. (ZIP) [file pone.0094112.s007.zip › Clozapine Figure S48 Forest Plot.tif]

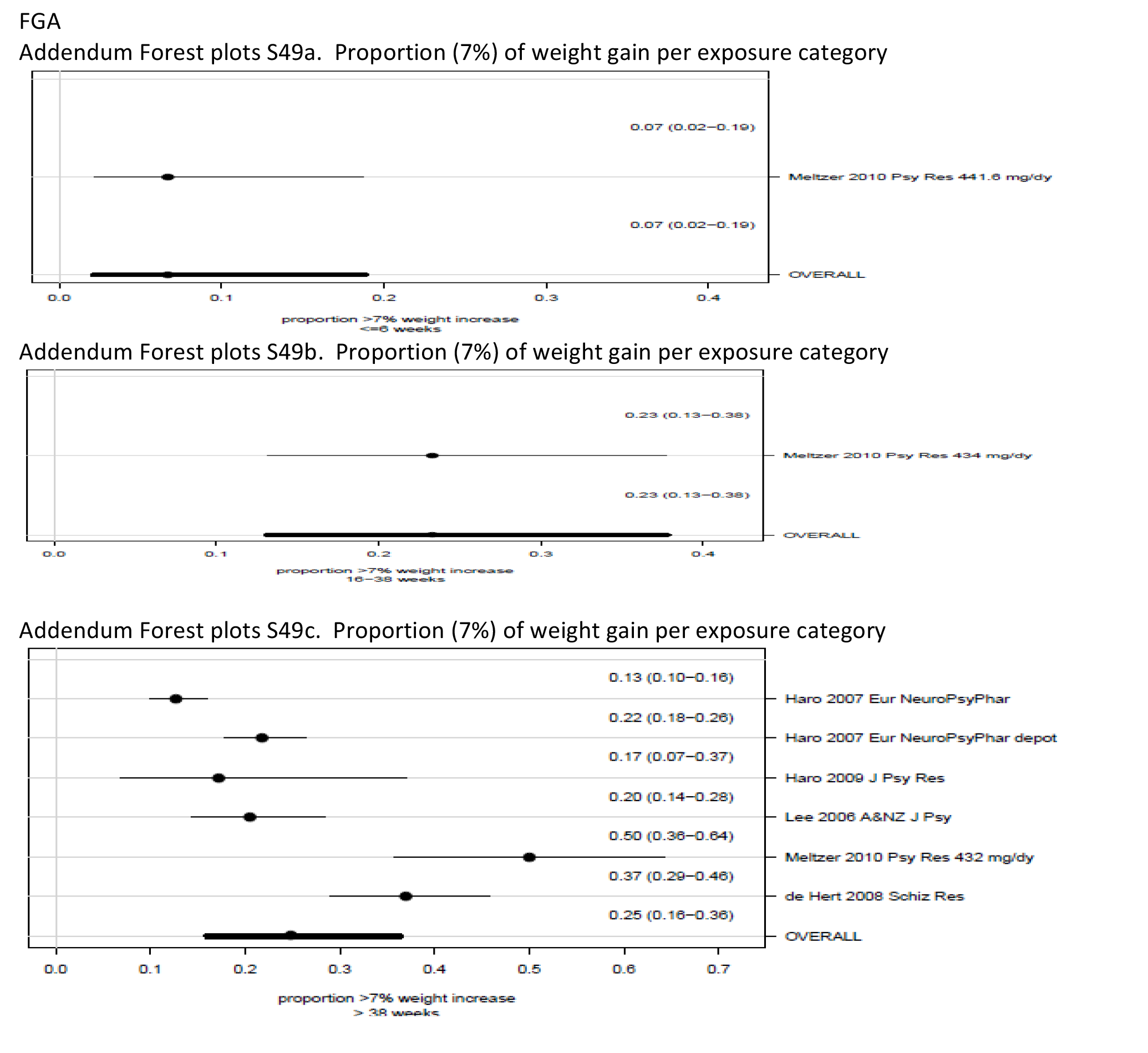

Supplement: File S6 — Forest Plots S44–S51d - Proportion (7%) of weight gain per exposure category. (ZIP) [file pone.0094112.s007.zip › FGA Figure S49 Forest Plot.tif]

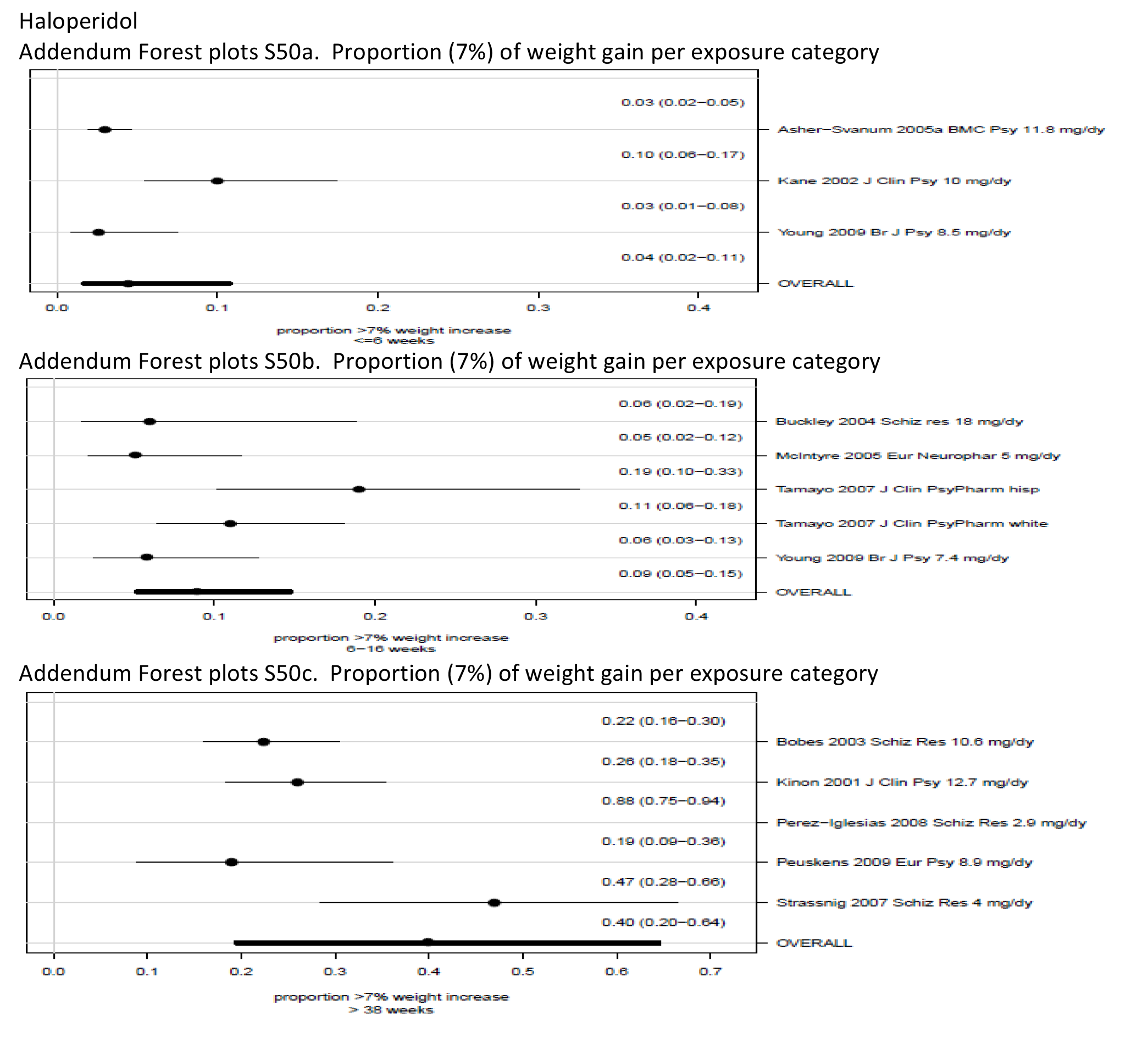

Supplement: File S6 — Forest Plots S44–S51d - Proportion (7%) of weight gain per exposure category. (ZIP) [file pone.0094112.s007.zip › Haloperidol Figure S50 Forest Plot.tif]

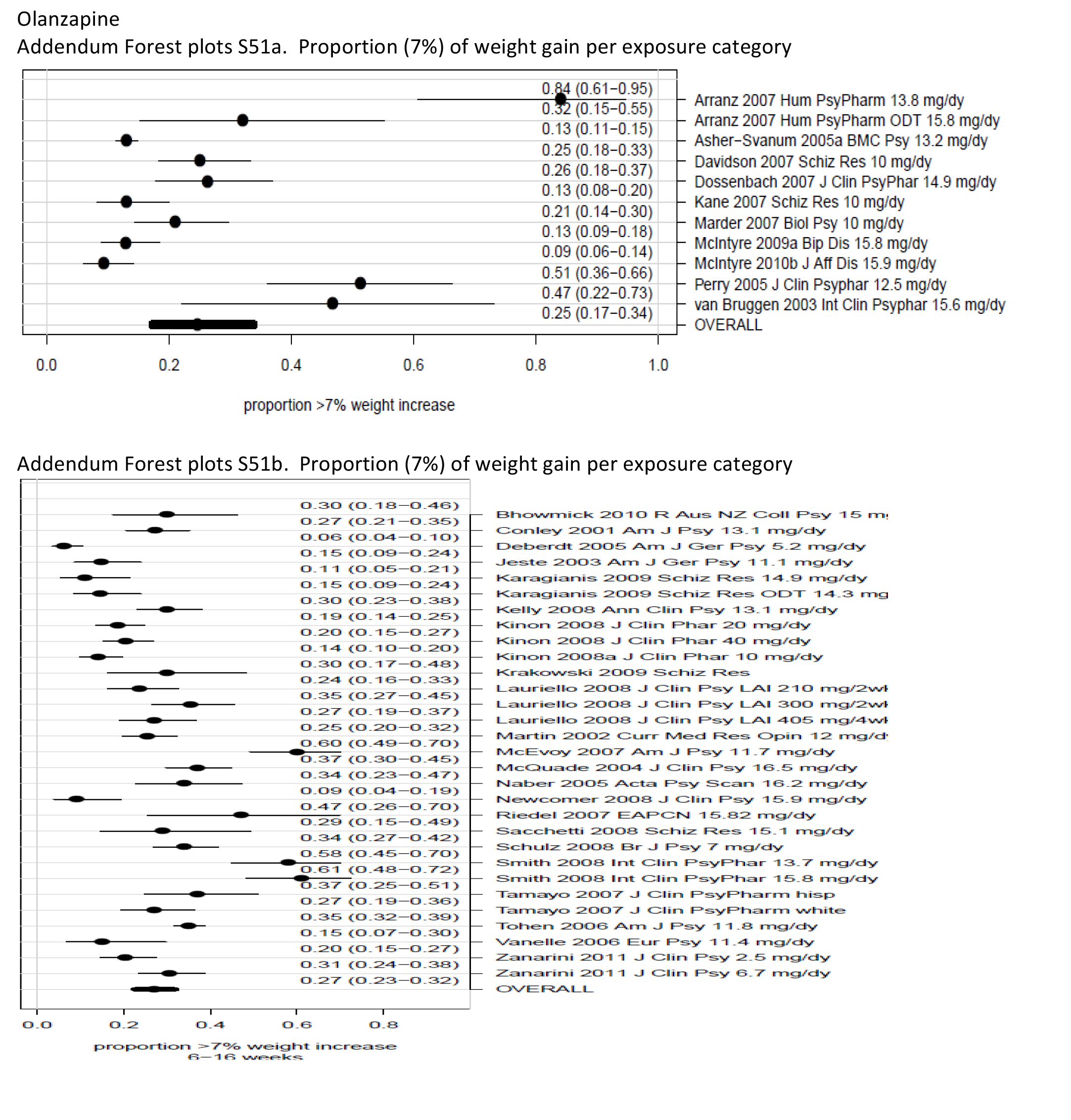

Supplement: File S6 — Forest Plots S44–S51d - Proportion (7%) of weight gain per exposure category. (ZIP) [file pone.0094112.s007.zip › Olanzapine Figure S51a-b Forest Plot.tif]

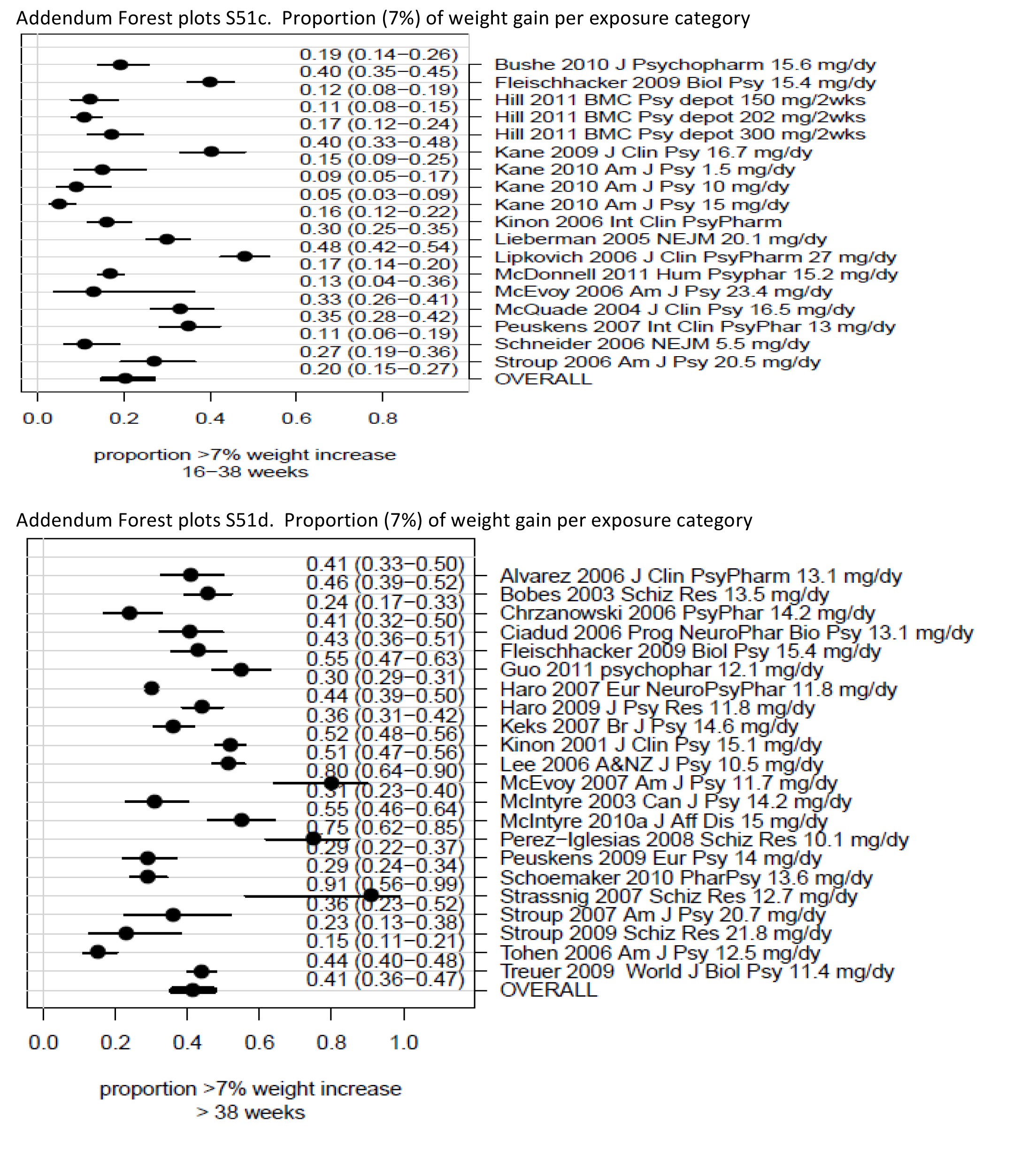

Supplement: File S6 — Forest Plots S44–S51d - Proportion (7%) of weight gain per exposure category. (ZIP) [file pone.0094112.s007.zip › Olanzapine Figure S51c Forest Plot.tif]

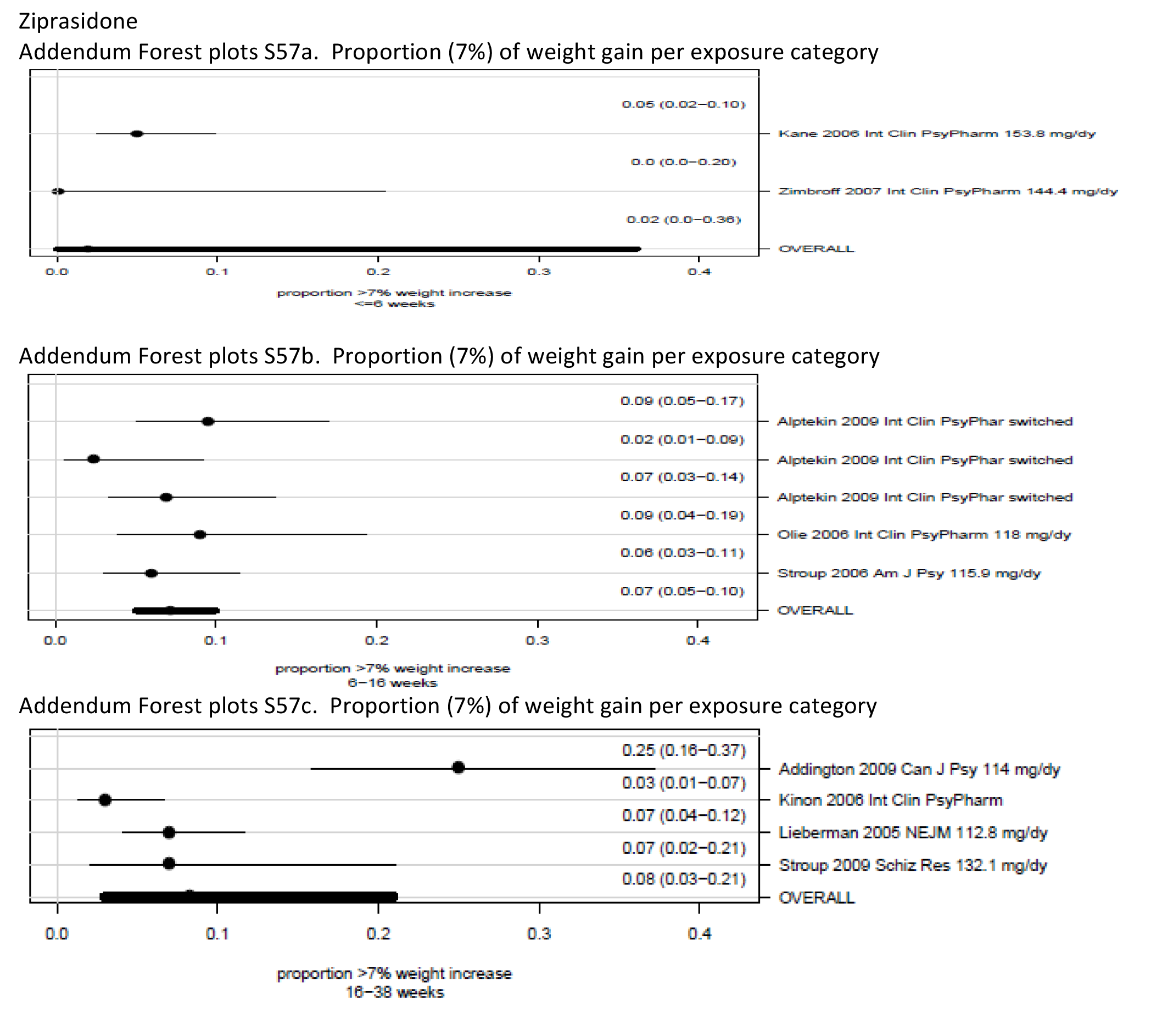

Supplement: File S7 — Forest Plots S52–S58 - Proportion (7%) of weight gain per exposure category. (ZIP) [file pone.0094112.s008.zip › Ziprasidone Figure S57 Forest Plot.tif]

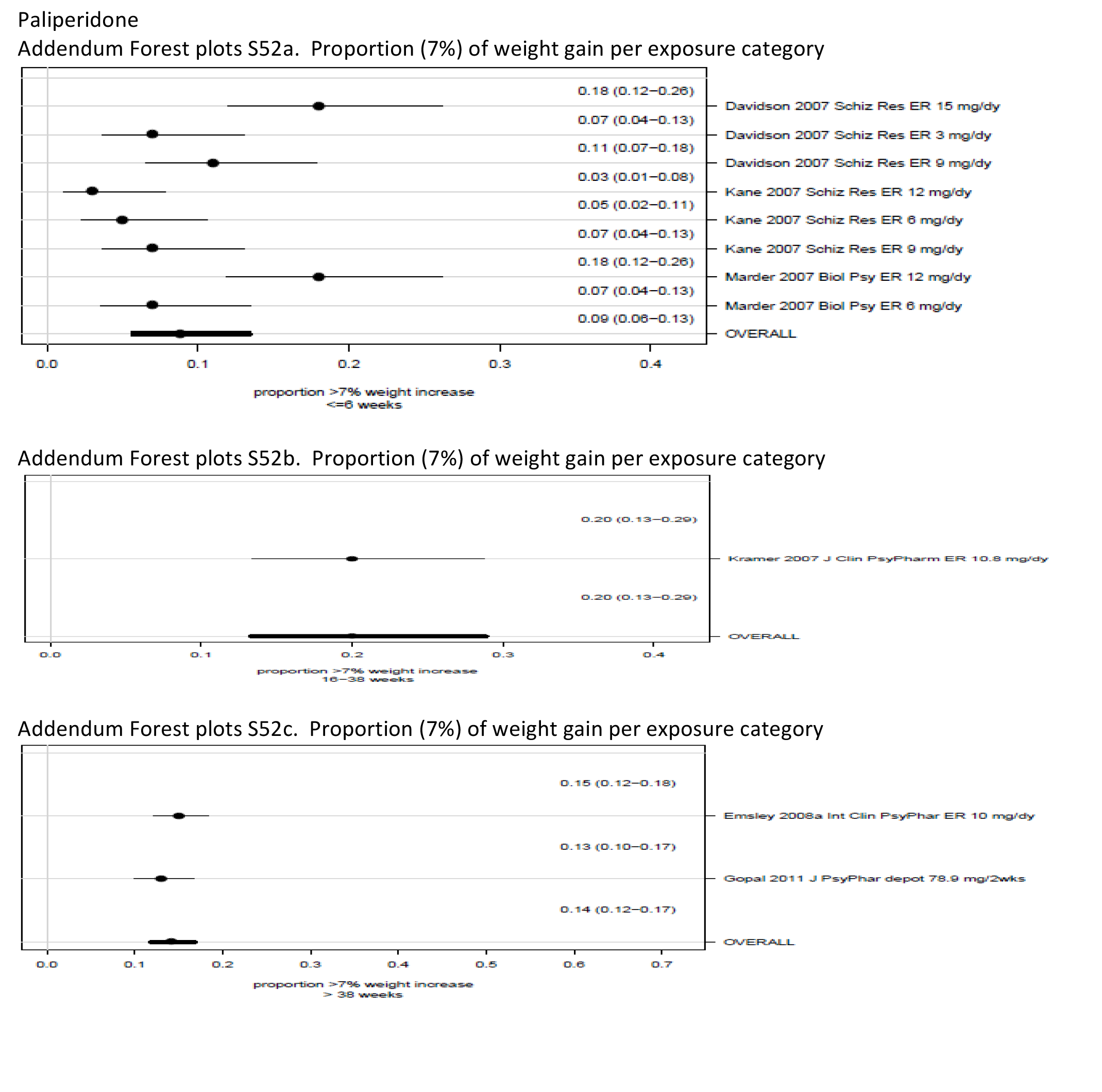

Supplement: File S7 — Forest Plots S52–S58 - Proportion (7%) of weight gain per exposure category. (ZIP) [file pone.0094112.s008.zip › Paliperidone Figure S52 Forest Plot.tif]

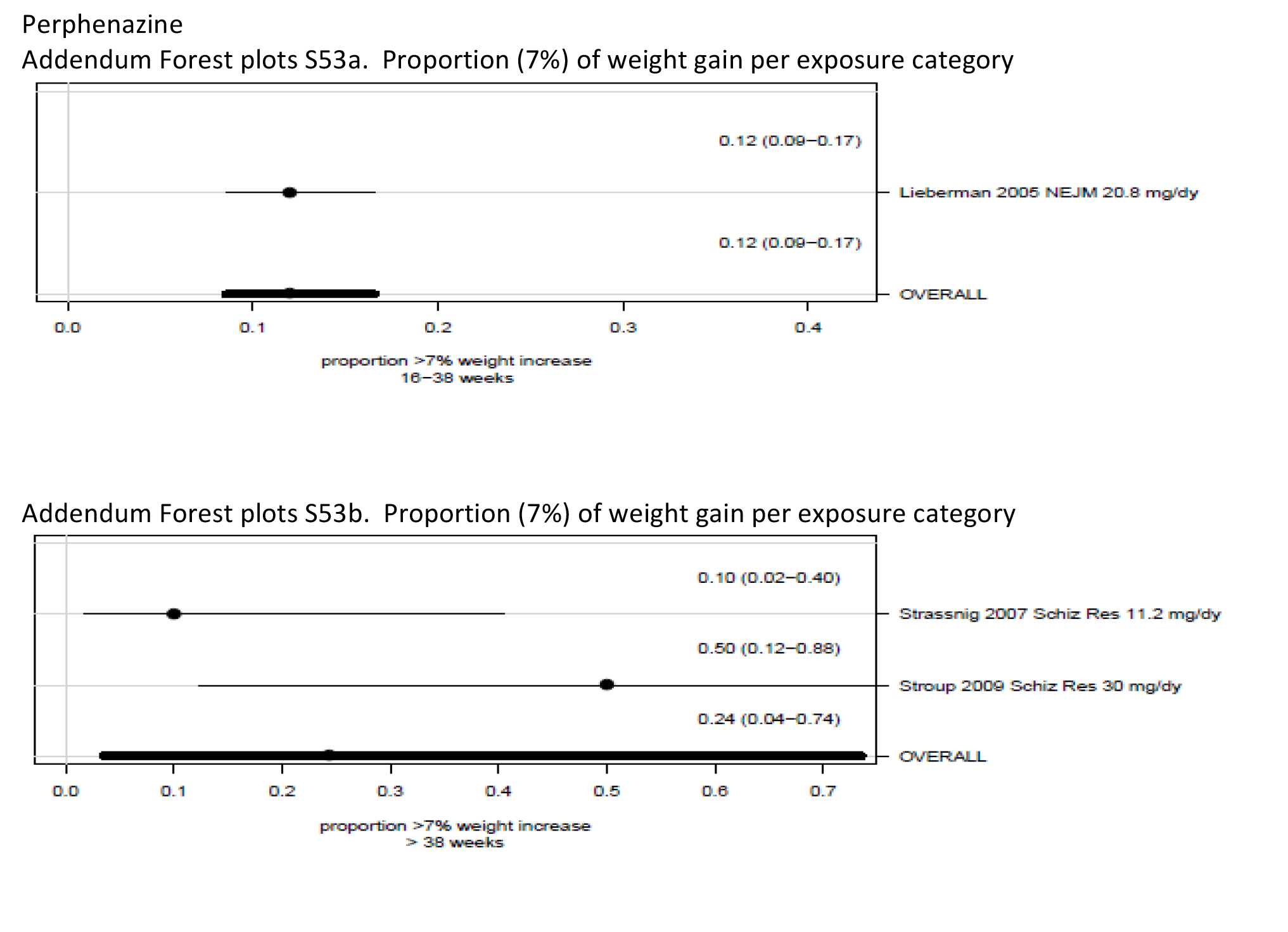

Supplement: File S7 — Forest Plots S52–S58 - Proportion (7%) of weight gain per exposure category. (ZIP) [file pone.0094112.s008.zip › Perphenazine Figure S53 Forest Plot.tif]

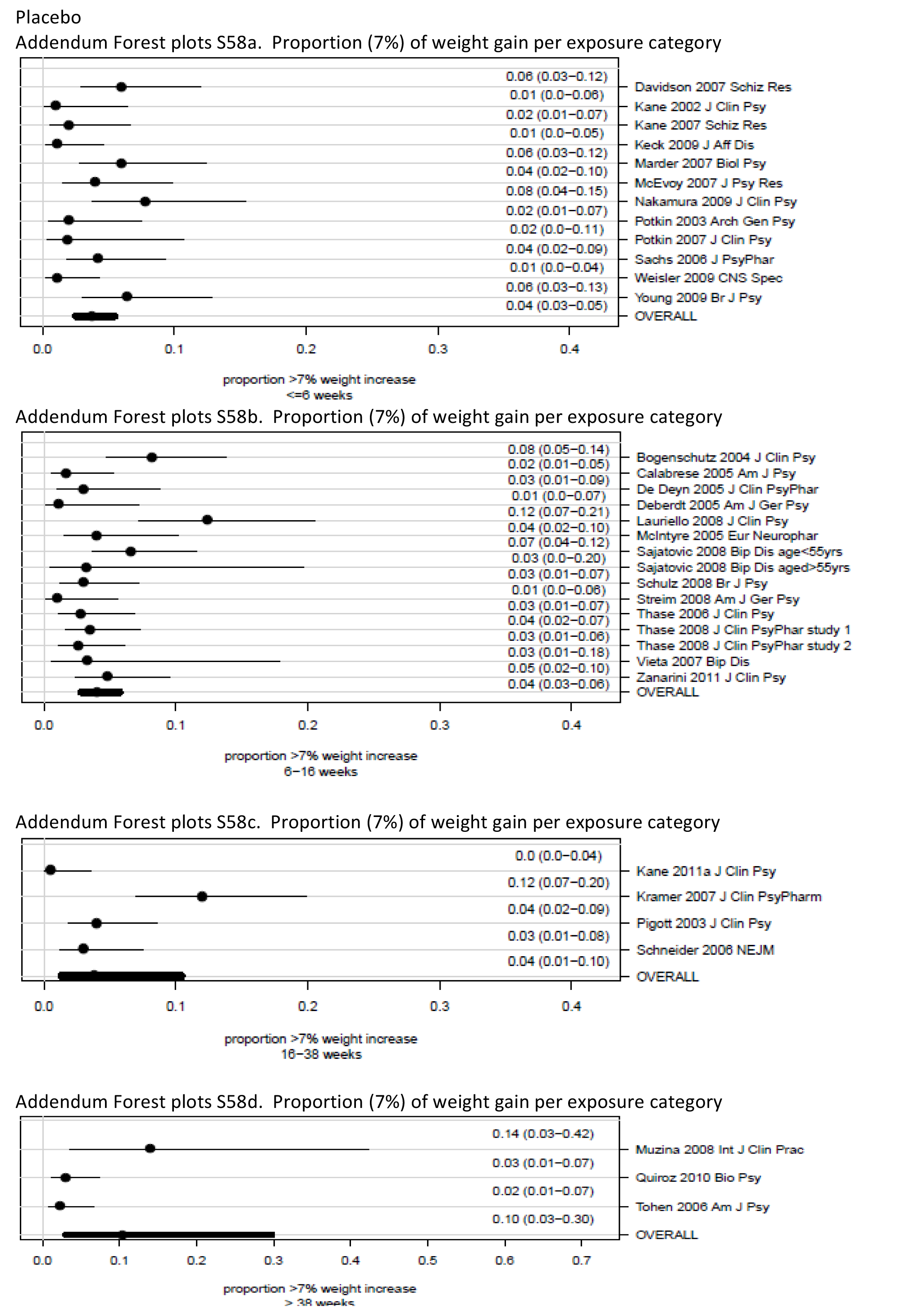

Supplement: File S7 — Forest Plots S52–S58 - Proportion (7%) of weight gain per exposure category. (ZIP) [file pone.0094112.s008.zip › Placebo Figure S58 Forest Plot.tif]

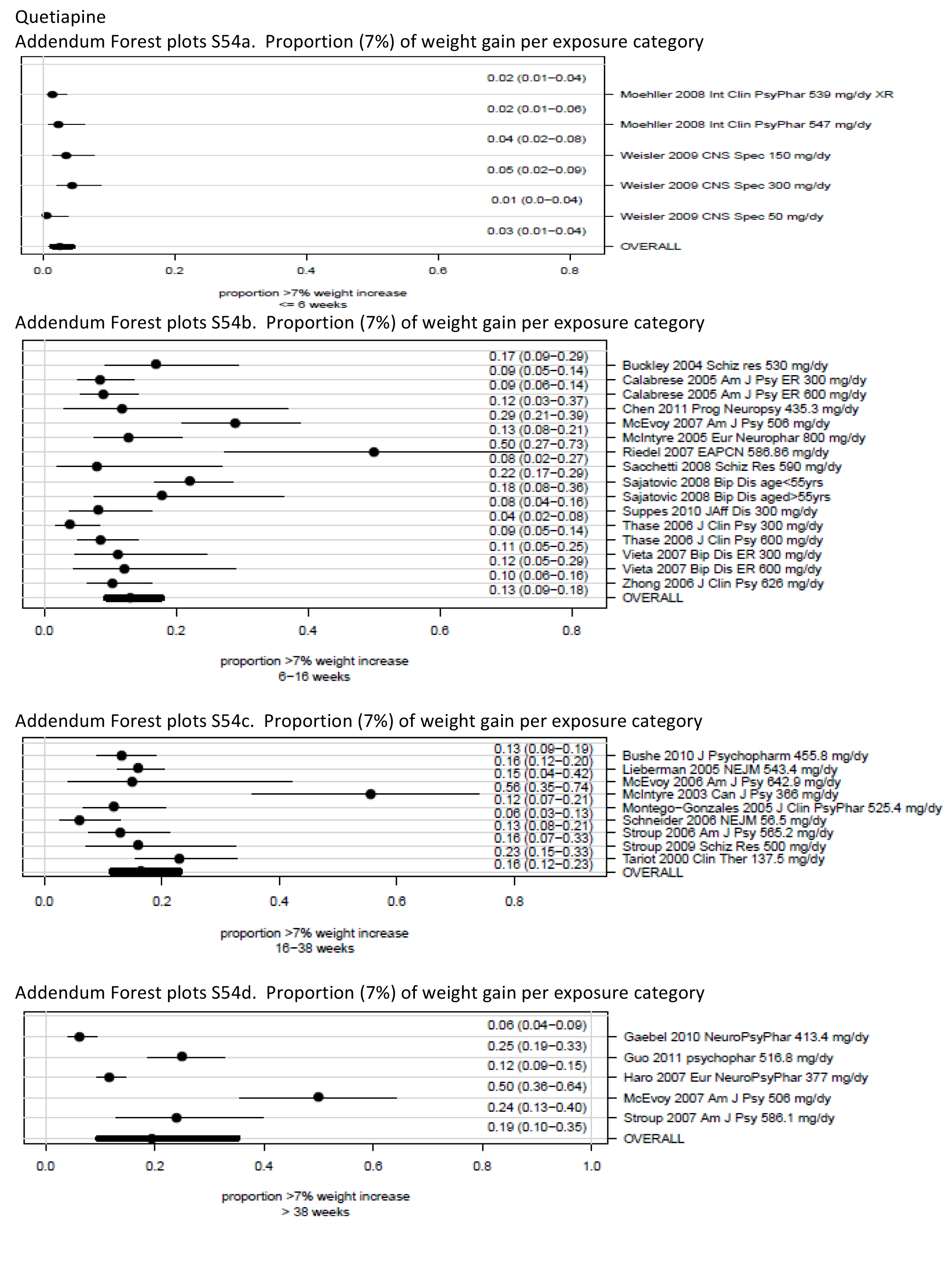

Supplement: File S7 — Forest Plots S52–S58 - Proportion (7%) of weight gain per exposure category. (ZIP) [file pone.0094112.s008.zip › Quetiapine Figure S54 Forest Plot.tif]

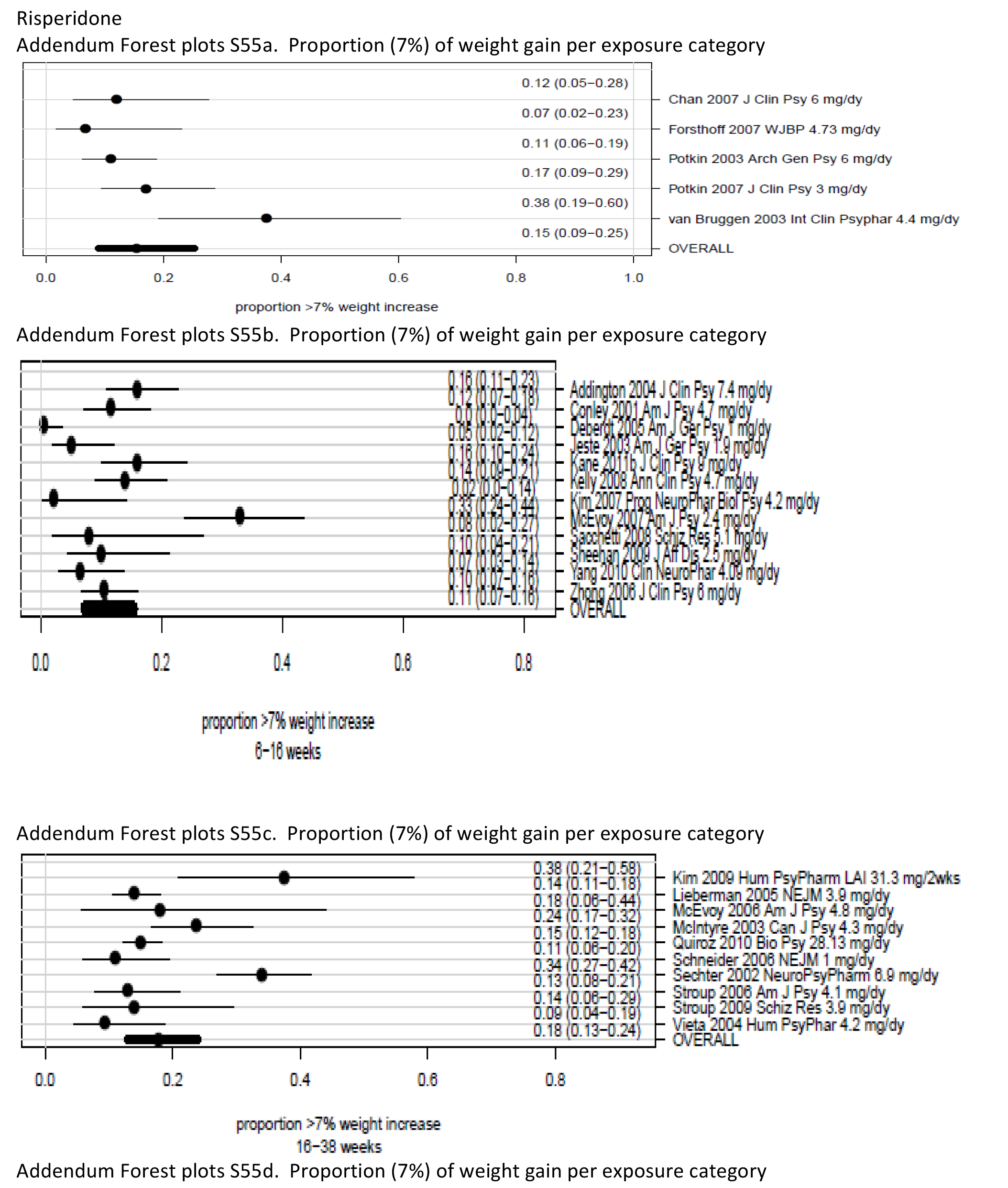

Supplement: File S7 — Forest Plots S52–S58 - Proportion (7%) of weight gain per exposure category. (ZIP) [file pone.0094112.s008.zip › Risperidone Figure S55 Forest Plot.tif]

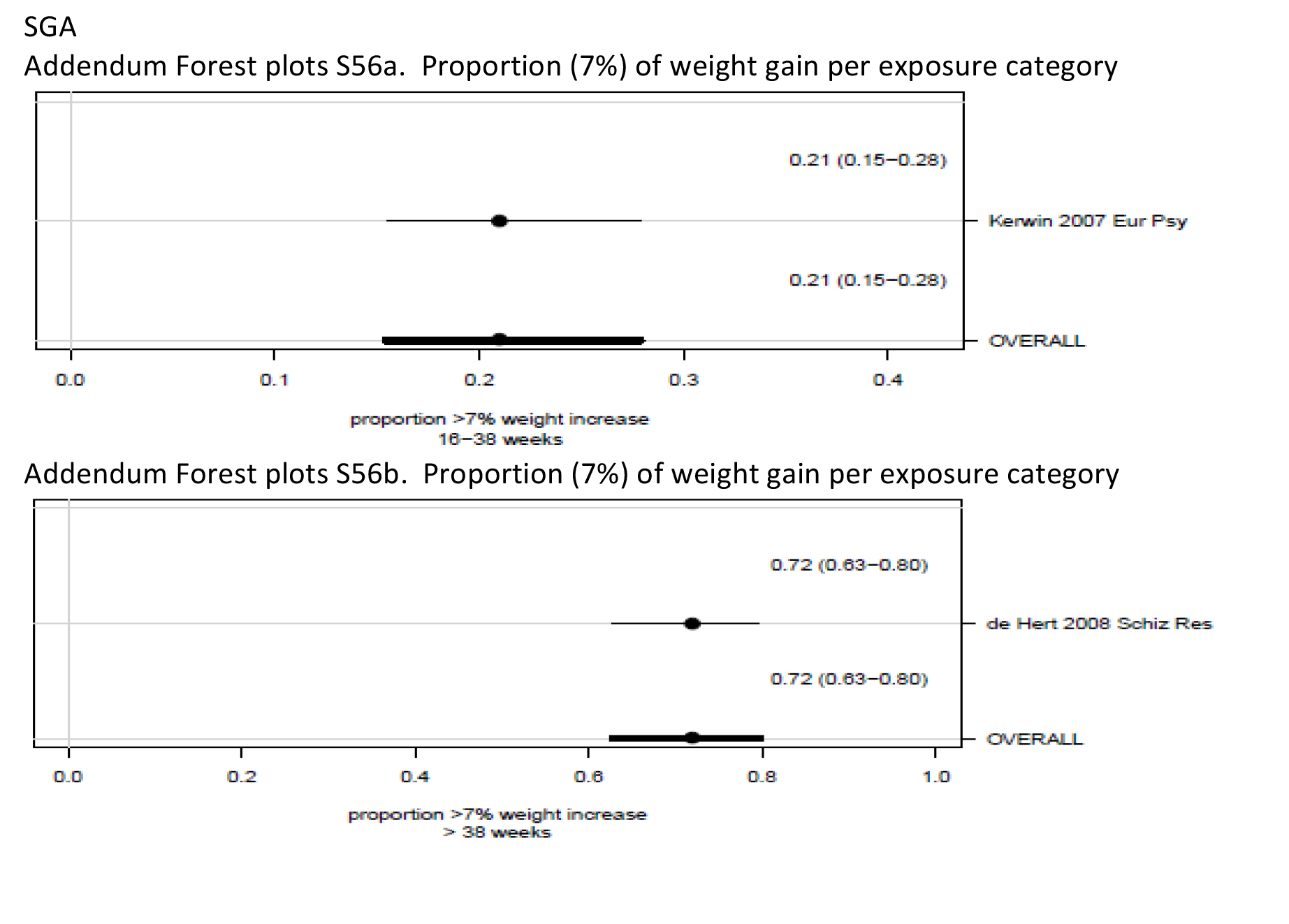

Supplement: File S7 — Forest Plots S52–S58 - Proportion (7%) of weight gain per exposure category. (ZIP) [file pone.0094112.s008.zip › SGA Figure S56 Forest Plot.tif]

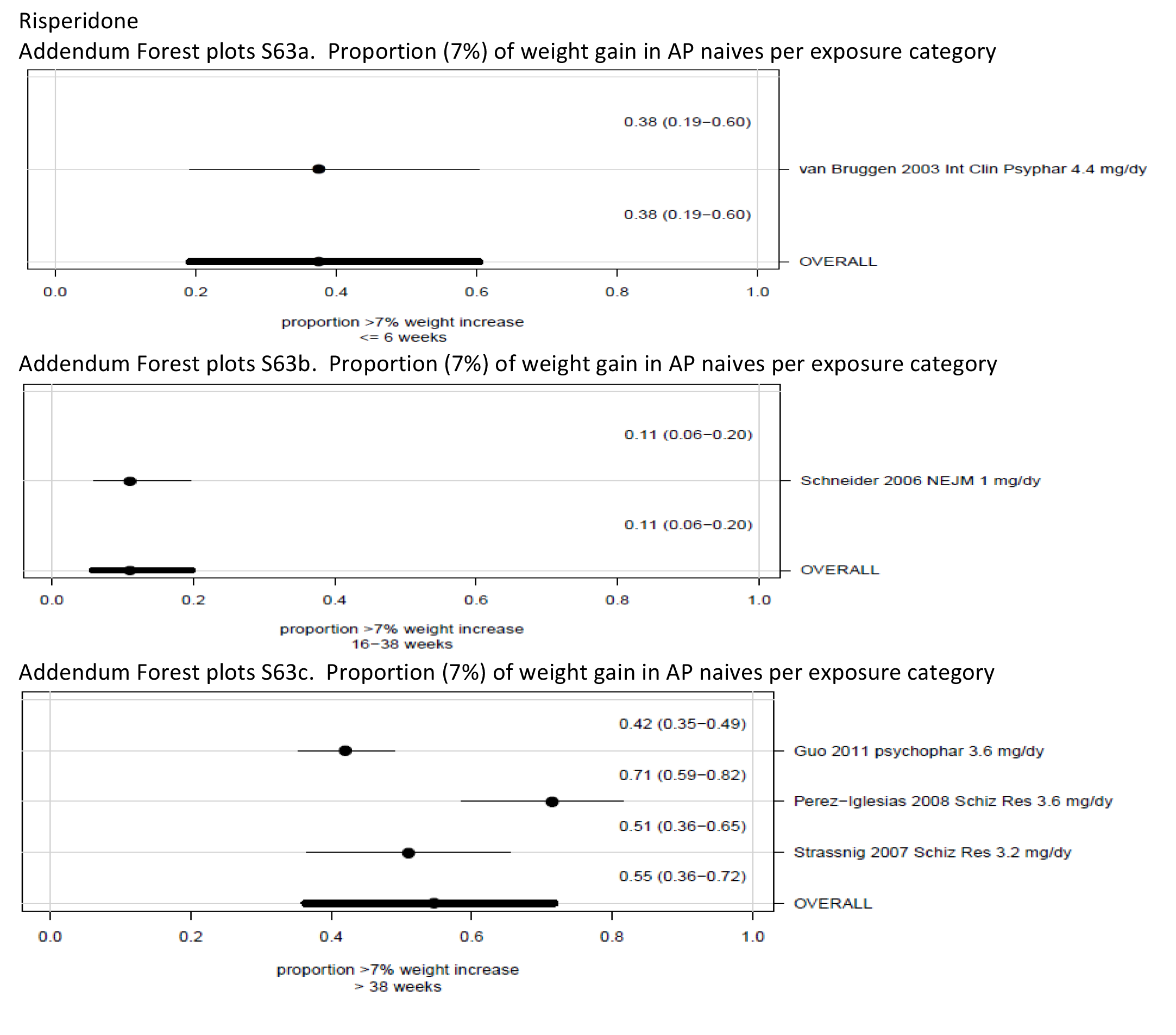

Supplement: File S8 — Forest plots S59a–S64c. Proportion (7%) of weight increase in AP naives per exposure category. (ZIP) [file pone.0094112.s009.zip › Risperidone Figure S63 Forest Plot.tif]

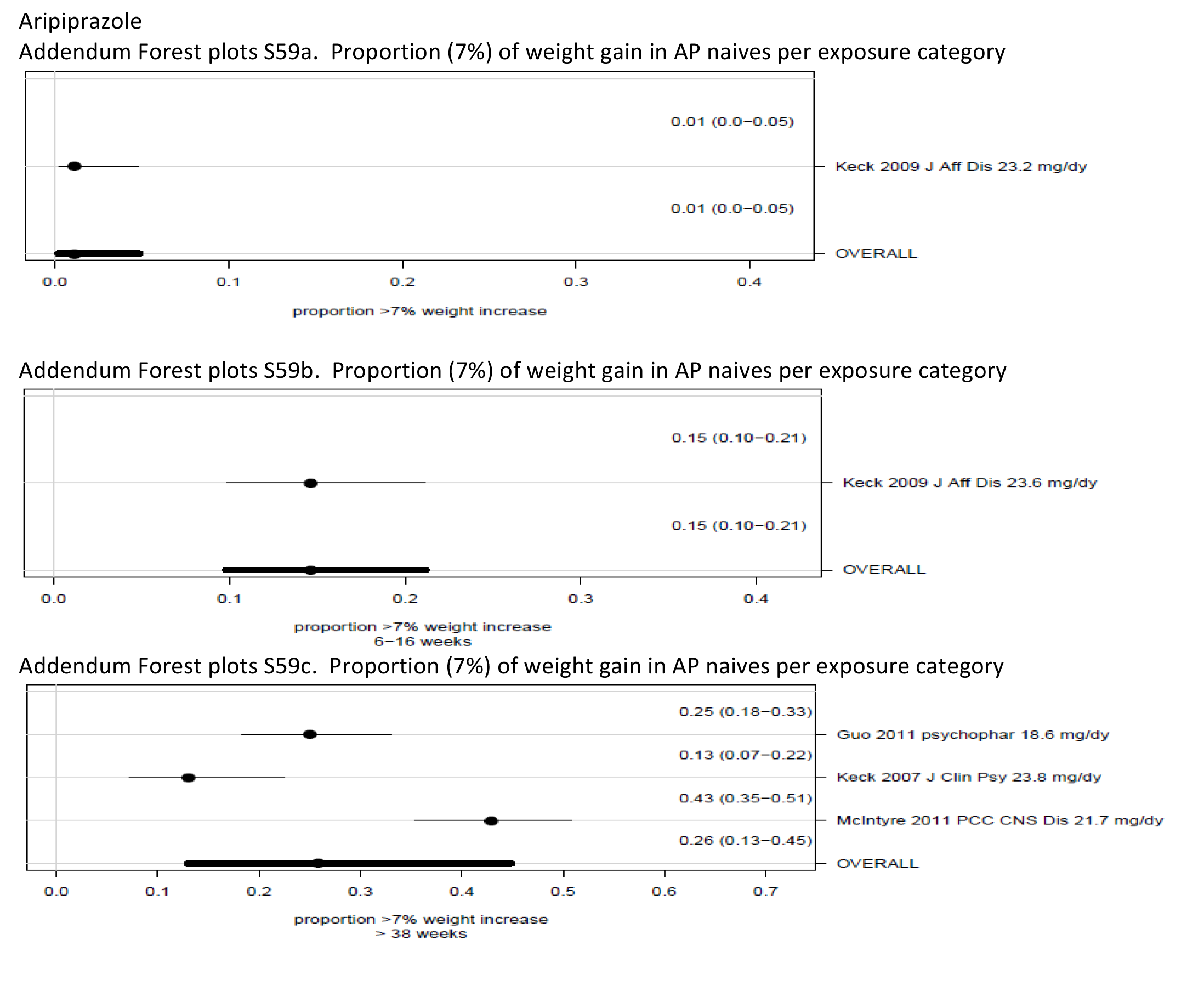

Supplement: File S8 — Forest plots S59a–S64c. Proportion (7%) of weight increase in AP naives per exposure category. (ZIP) [file pone.0094112.s009.zip › Aripiprazole Figure S59 Forest Plot.tif]

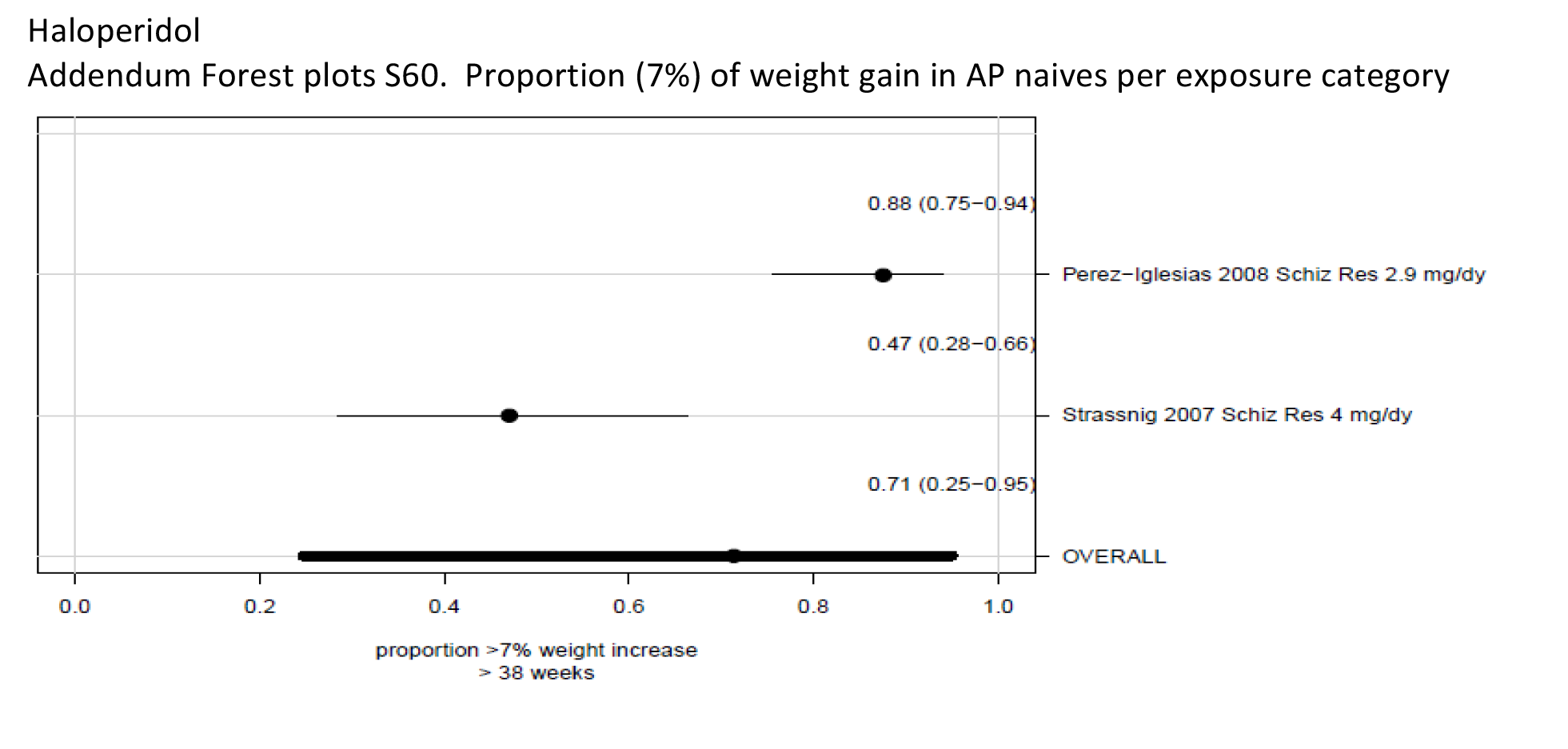

Supplement: File S8 — Forest plots S59a–S64c. Proportion (7%) of weight increase in AP naives per exposure category. (ZIP) [file pone.0094112.s009.zip › Haloperidol Figure S60 Forest Plot.tif]

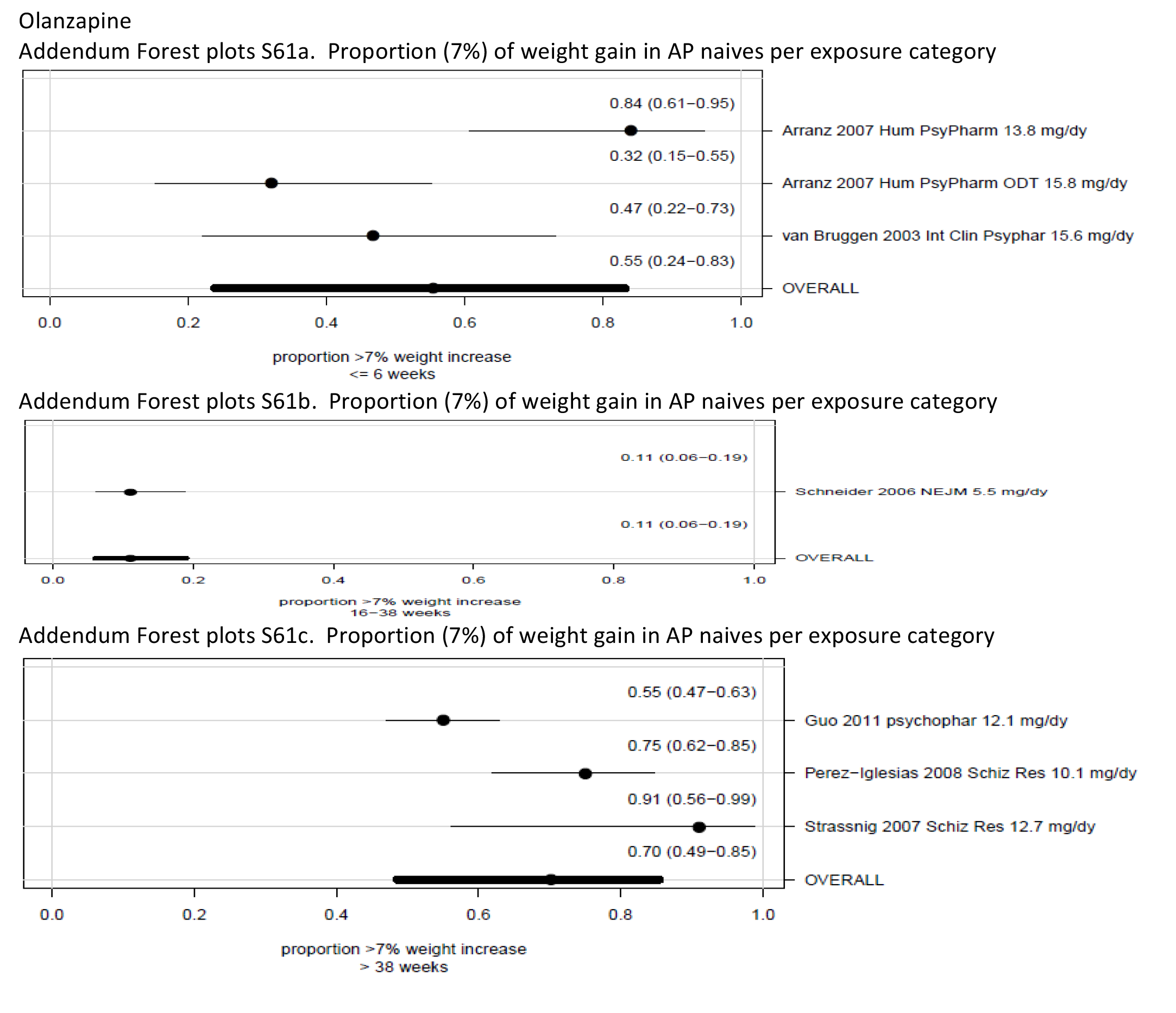

Supplement: File S8 — Forest plots S59a–S64c. Proportion (7%) of weight increase in AP naives per exposure category. (ZIP) [file pone.0094112.s009.zip › Olanzapine Figure S61 Forest Plot.tif]

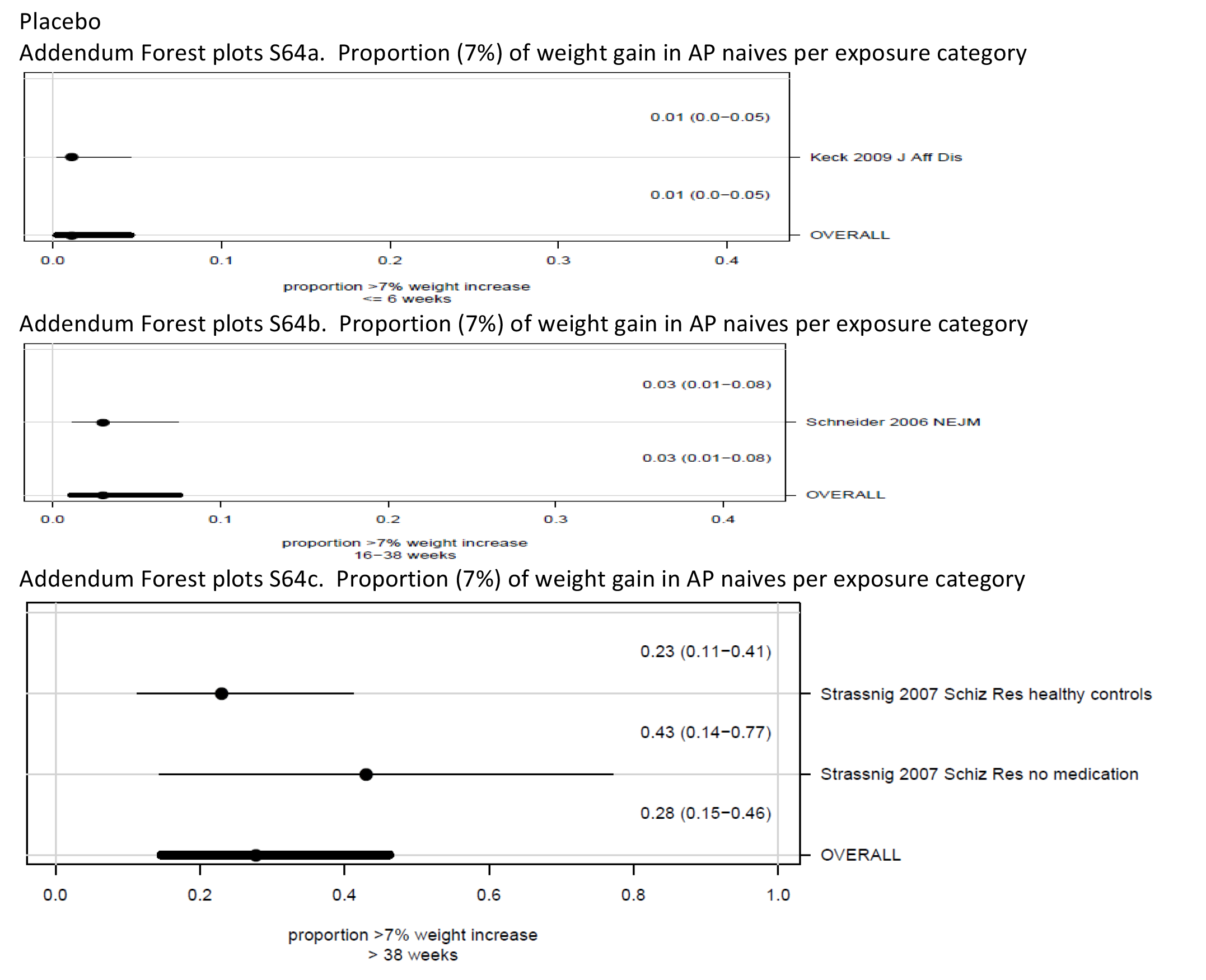

Supplement: File S8 — Forest plots S59a–S64c. Proportion (7%) of weight increase in AP naives per exposure category. (ZIP) [file pone.0094112.s009.zip › Placebo Figure S64 Forest Plot.tif]

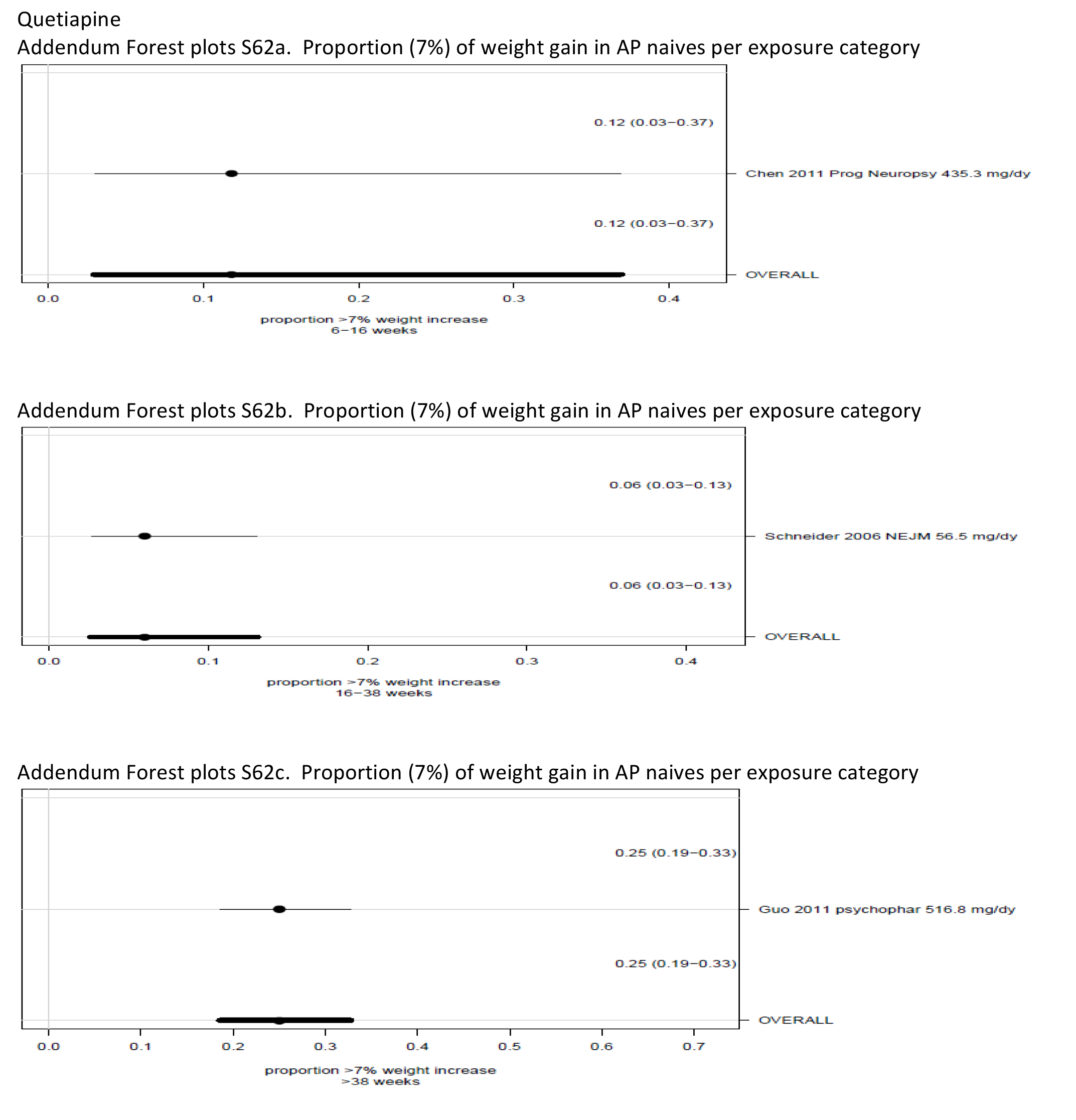

Supplement: File S8 — Forest plots S59a–S64c. Proportion (7%) of weight increase in AP naives per exposure category. (ZIP) [file pone.0094112.s009.zip › Quetiapine Figure S62 Forest Plot.tif]

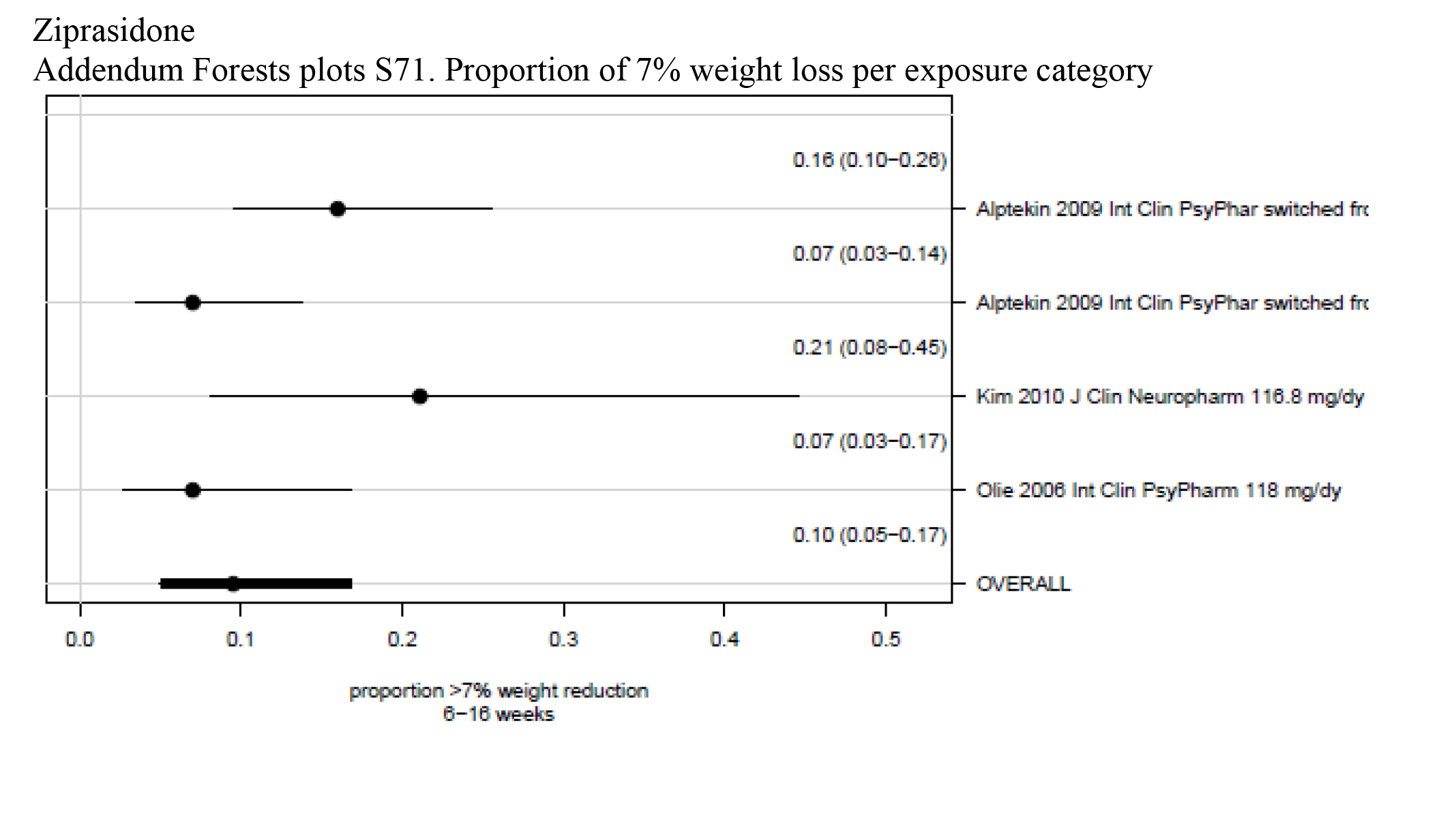

Supplement: File S9 — Forest Plots S65–S72d. Proportion of 7% weight loss per exposure category. (ZIP) [file pone.0094112.s010.zip › Ziprasidone Figure S71 Forest Plot.tif]

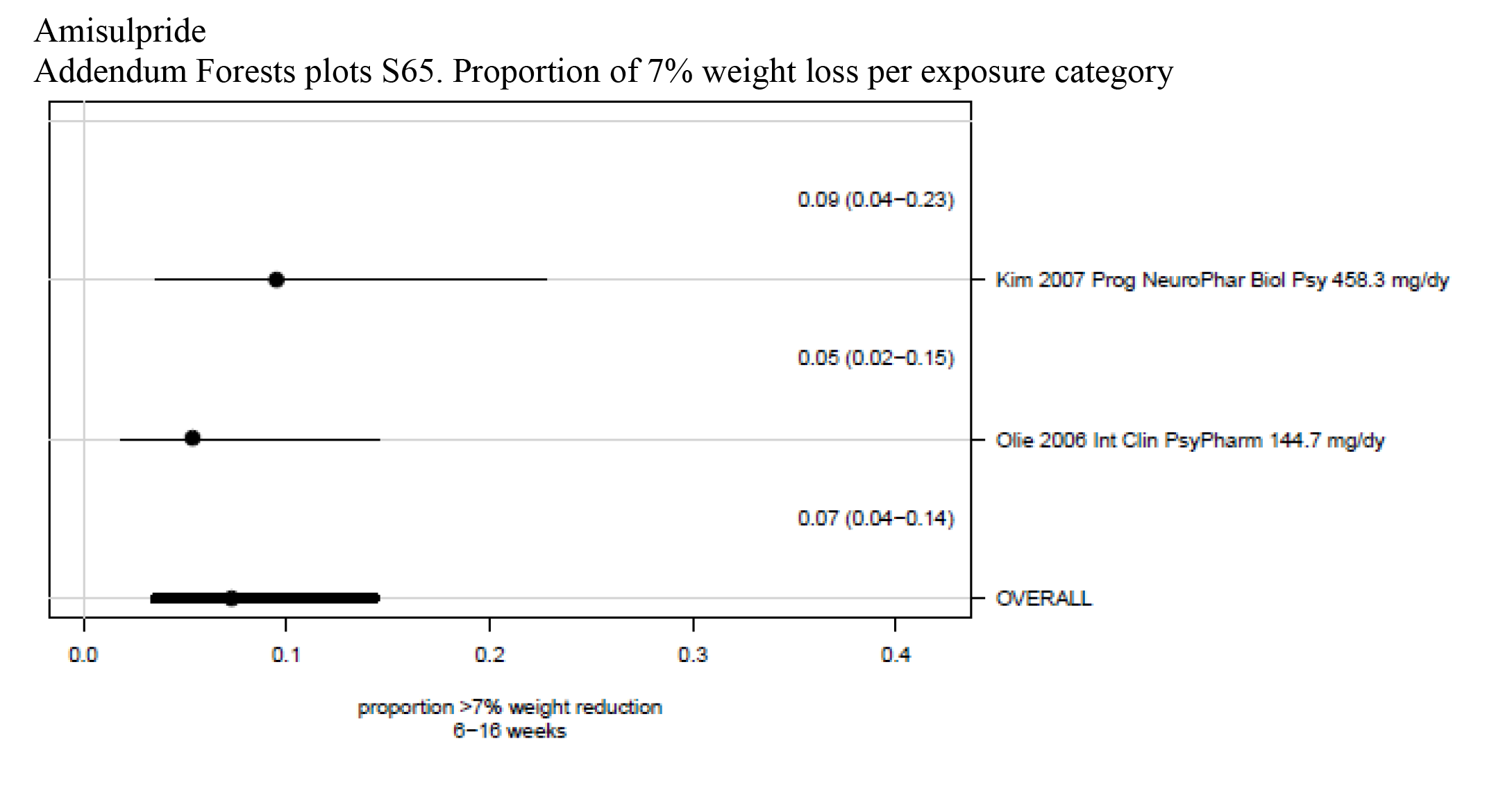

Supplement: File S9 — Forest Plots S65–S72d. Proportion of 7% weight loss per exposure category. (ZIP) [file pone.0094112.s010.zip › Amisulpride Figure S65 Forest Plot.tiff]

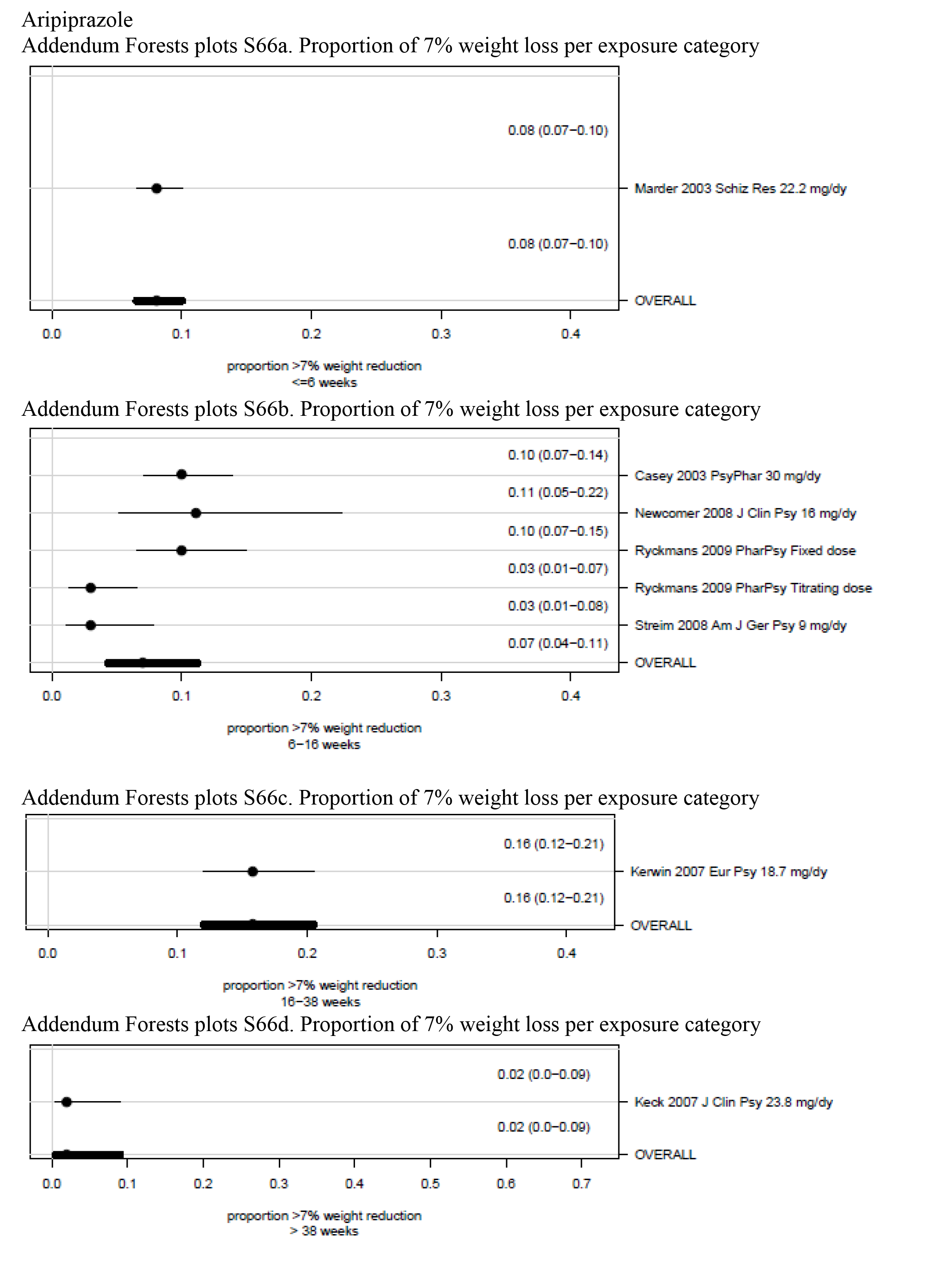

Supplement: File S9 — Forest Plots S65–S72d. Proportion of 7% weight loss per exposure category. (ZIP) [file pone.0094112.s010.zip › Aripiprazole Figure S66 Forest Plot.tif]

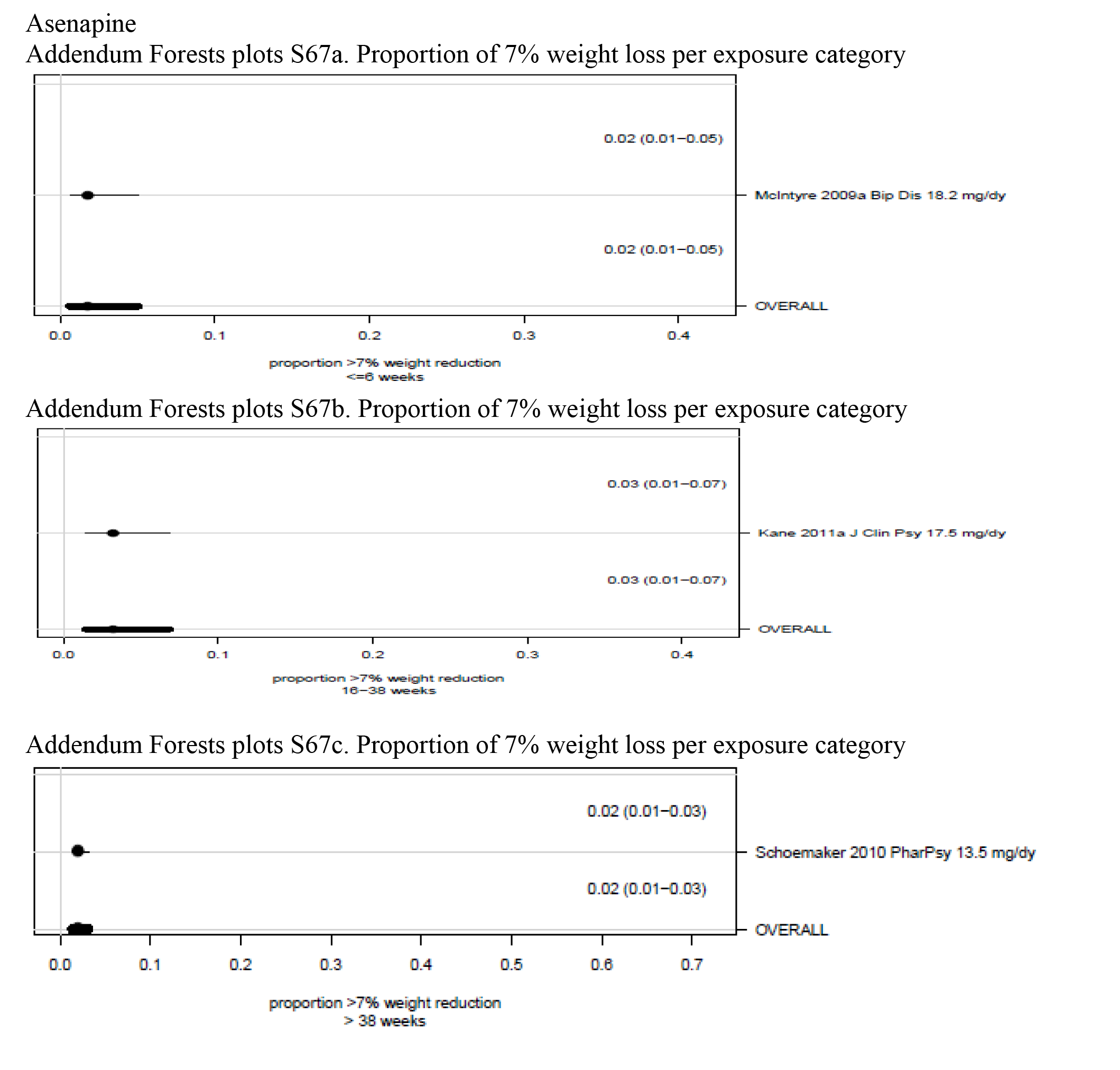

Supplement: File S9 — Forest Plots S65–S72d. Proportion of 7% weight loss per exposure category. (ZIP) [file pone.0094112.s010.zip › Asenapine Figure S67 Forest Plot.tif]

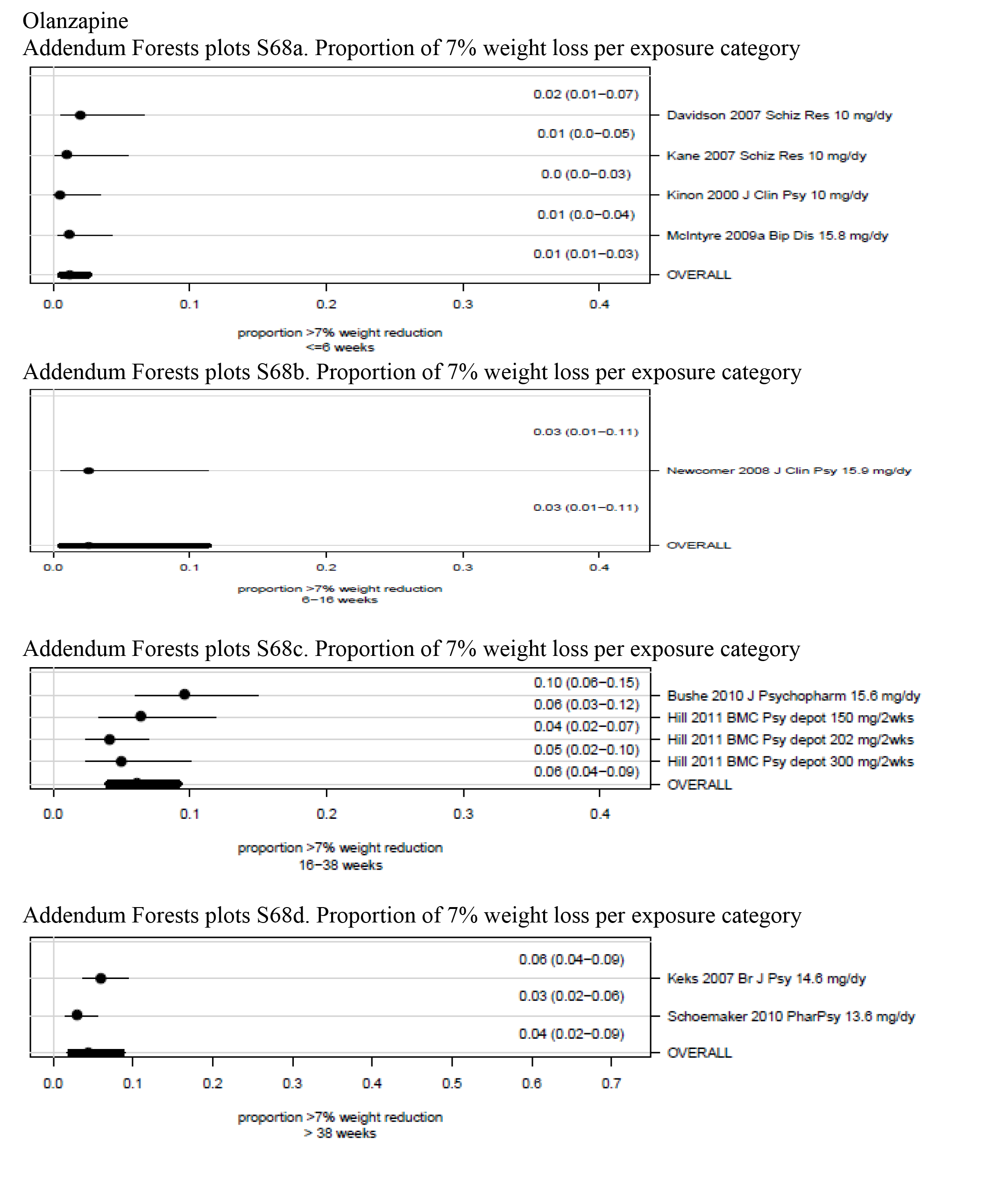

Supplement: File S9 — Forest Plots S65–S72d. Proportion of 7% weight loss per exposure category. (ZIP) [file pone.0094112.s010.zip › Olanzapine Figure S68 Forest Plot.tif]

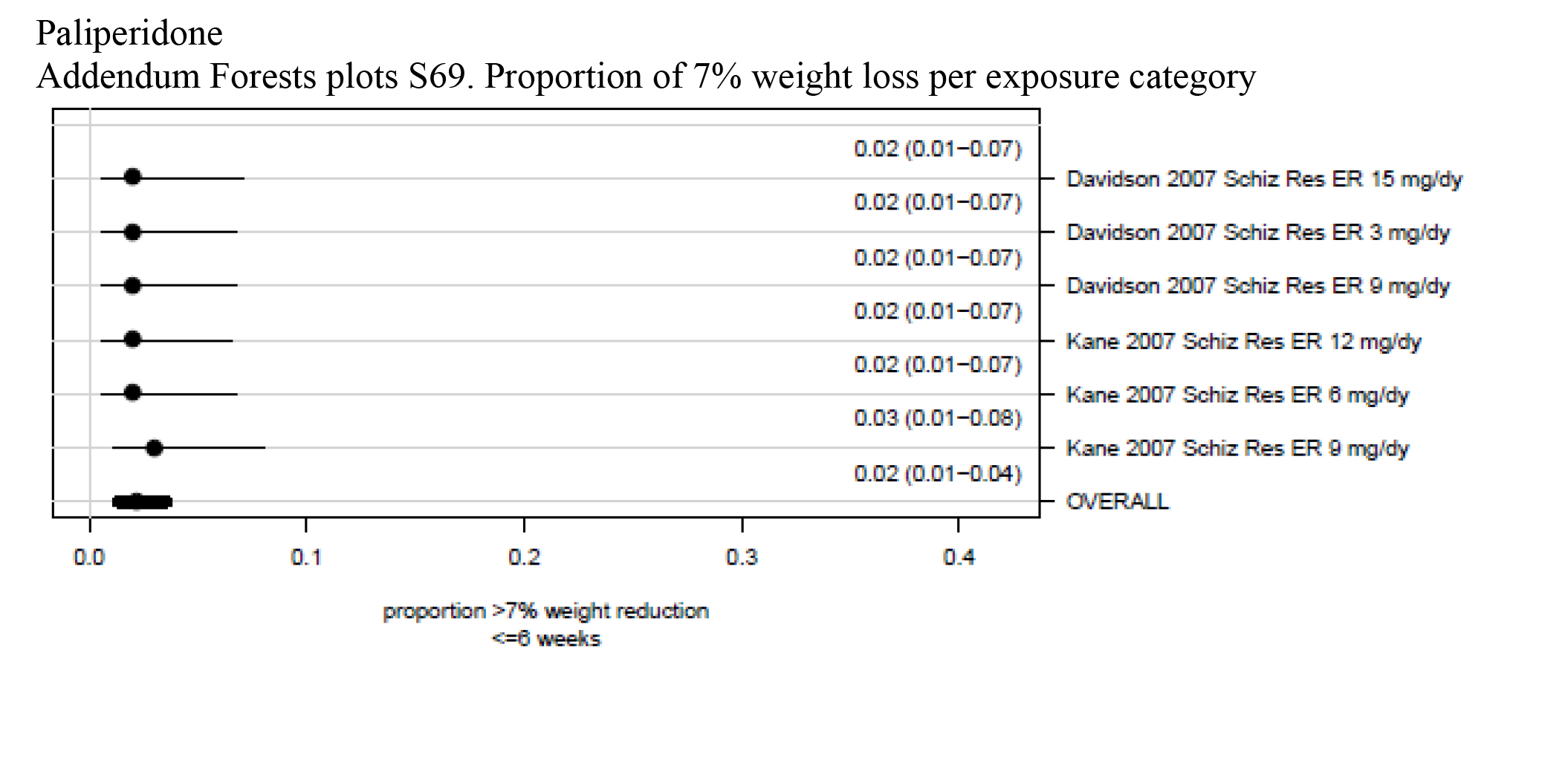

Supplement: File S9 — Forest Plots S65–S72d. Proportion of 7% weight loss per exposure category. (ZIP) [file pone.0094112.s010.zip › Paliperidone Figure S69 Forest Plot.tif]

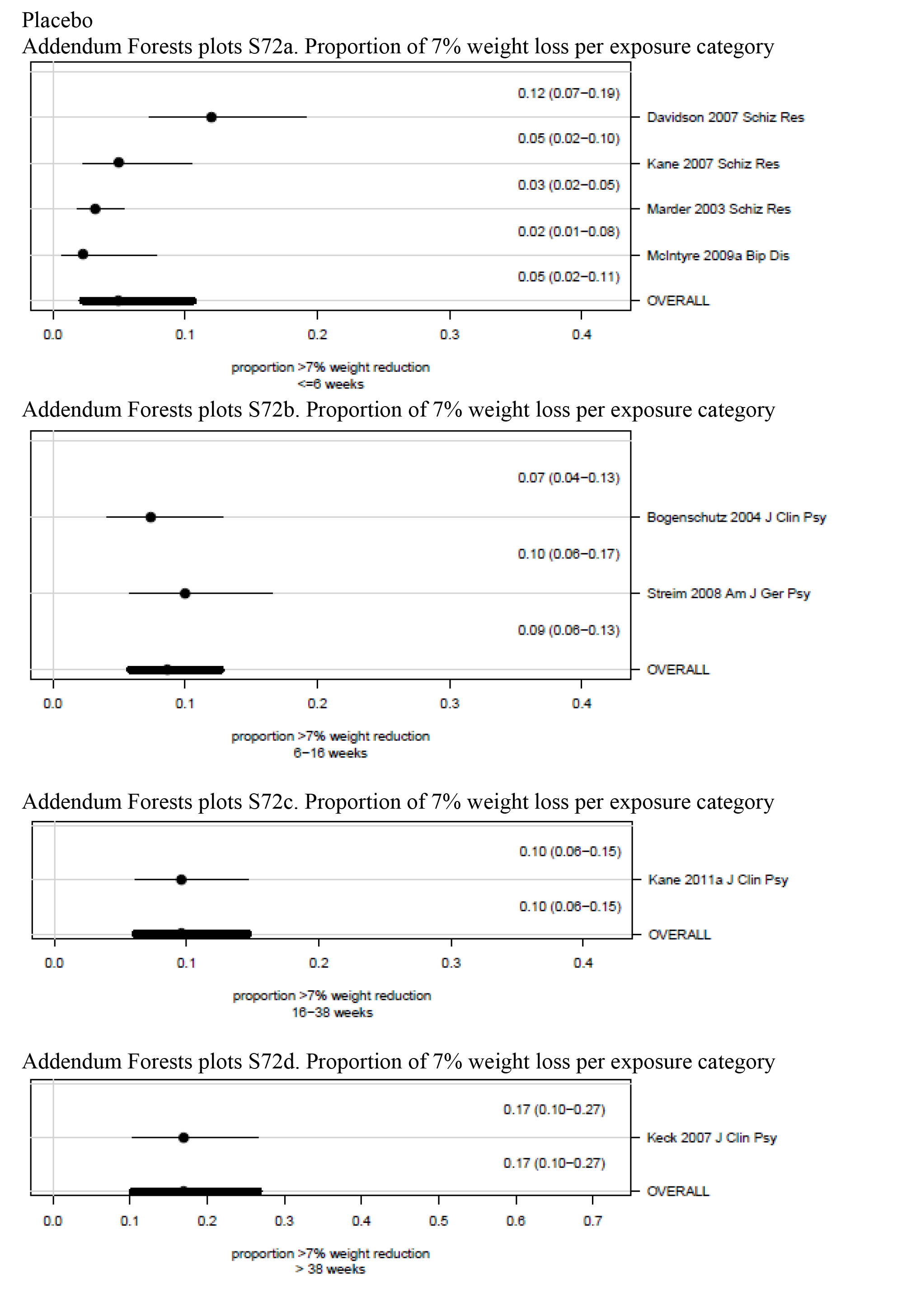

Supplement: File S9 — Forest Plots S65–S72d. Proportion of 7% weight loss per exposure category. (ZIP) [file pone.0094112.s010.zip › Placebo Figure S72 Forest Plot.tif]

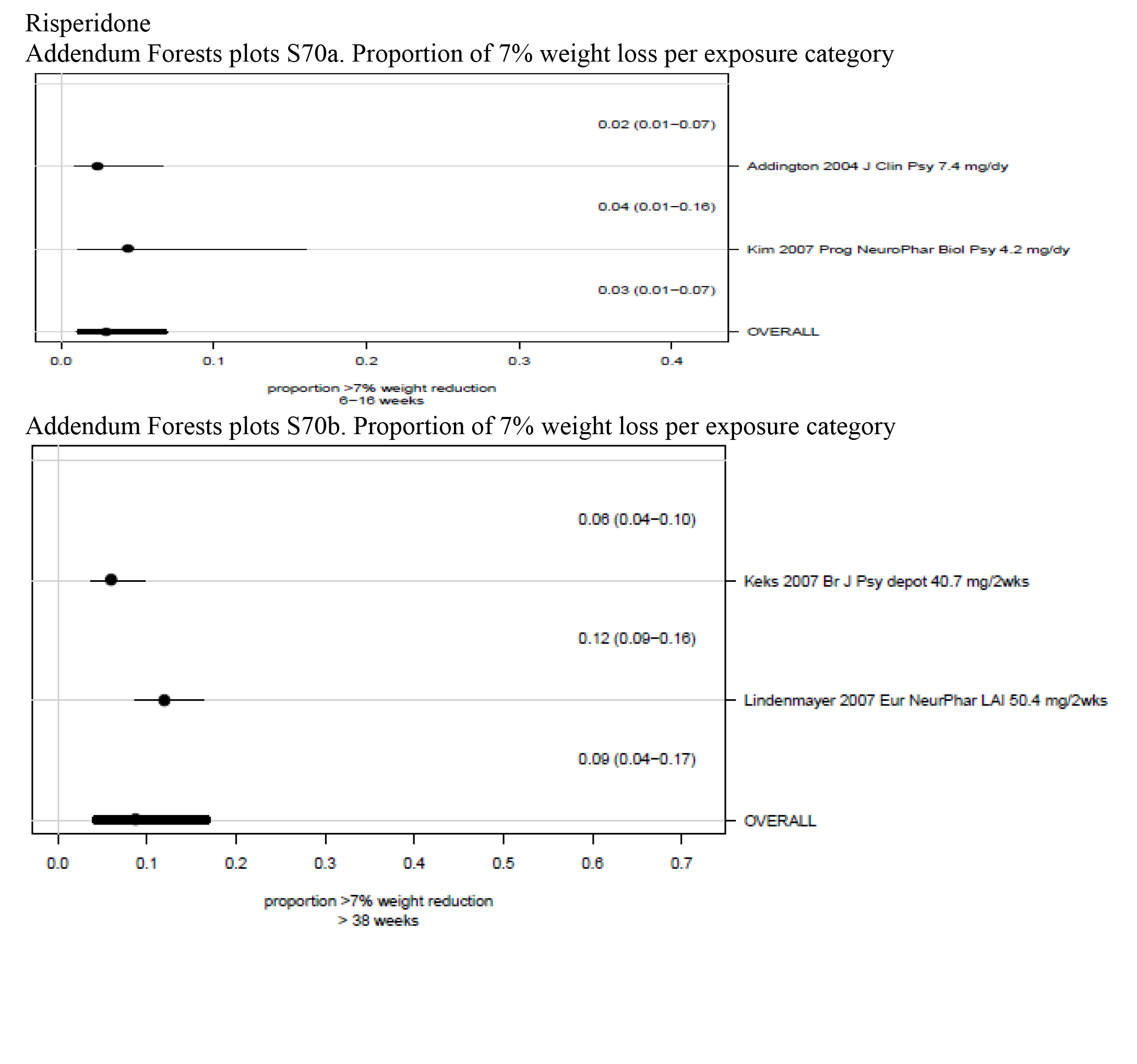

Supplement: File S9 — Forest Plots S65–S72d. Proportion of 7% weight loss per exposure category. (ZIP) [file pone.0094112.s010.zip › Risperidone Figure S70 Forest Plot.tif]
