# Supplementary material for: Almost All Antipsychotics Result in Weight Gain: A Meta-Analysis
Source: PLoS One. 2014 Apr 24;9(4):e94112. doi: 10.1371/journal.pone.0094112 (PMC3998960; doi:10.1371/journal.pone.0094112)
Supplement: Table S1 — Number of studies reporting on each of the antipsychotics (switch studies and drug naive separately). (DOCX) [file pone.0094112.s011.docx]

Table S1. Number of studies reporting on each of the antipsychotics (switch studies and drug naive separately)

|  | Weight change (switch / drug naive) | BMI change | percentage > 7% weight gain | percentage > 7% weight loss |
| --- | --- | --- | --- | --- |
| amisulpride | 8/0 | 3/0 | 7/0 | 2/0 |
| amoxapine | 1/0 | 0/0 | 0/0 | 0/0 |
| aripiprazole | 26/6 | 5/0 | 22/4 | 6/1 |
| asenapine | 5/0 | 0/0 | 6/0 | 3/0 |
| blonanserin | 1/0 | 0/0 | 1/0 | 0/0 |
| chlorpromazine | 2/1 | 0/0 | 2/1 | 0/0 |
| clozapine | 21/2 | 14/2 | 5/1 | 1/0 |
| FGA | 7/2 | 8/2 | 4/1 | 1/0 |
| fluphenazine | 1/0 | 1/0 | 1/0 | 0/0 |
| haloperidol | 28/4 | 5/4 | 9/2 | 1/0 |
| iloperidone | 1/0 | 0/0 | 0/0 | 0/0 |
| levopromethazine | 1/0 | 0/0 | 0/0 | 0/0 |
| lurasidone | 1/0 | 0/0 | 1/0 | 0/0 |
| melperone | 1/0 | 1/0 | 0/0 | 0/0 |
| olanzapine | 118/26 | 41/11 | 59/0 | 9/0 |
| paliperidone | 10/0 | 3/0 | 6/0 | 2/0 |
| perphenazine | 2/1 | 0/1 | 2/1 | 0/0 |
| pimozide | 0/0 | 0/0 | 0/0 | 0/0 |
| quetiapine | 44/6 | 12/4 | 24/3 | 1/0 |
| risperidone | 78/12 | 31/8 | 40/5 | 4/0 |
| SGA | 1/1 | 1/2 | 1/1 | 1/0 |
| sertindole | 3/0 | 0/0 | 1/0 | 0/0 |
| sulpiride | 0/1 | 0/1 | 0/1 | 0/0 |
| ziprasidone | 16/1 | 5/1 | 9/0 | 3/0 |
| zuclopentixol | 1/0 | 0/0 | 0/0 | 0/0 |
| placebo / healthy control / no medication | 40/15 | 3/4 | 30/3 | 7/1 |
