# Supplementary material for: Almost All Antipsychotics Result in Weight Gain: A Meta-Analysis
Source: PLoS One. 2014 Apr 24;9(4):e94112. doi: 10.1371/journal.pone.0094112 (PMC3998960; doi:10.1371/journal.pone.0094112)
Supplement: Table S2 — Weight changes per exposure category. (DOCX) [file pone.0094112.s012.docx]

Table S2. Weight changes per exposure category

| **weight change per period** | **Effect Size** | **95%CI** | ***Hetero-geneity*** | ***df*** | ***p*** | ***I^2^*** | ***Tau^2^*** | ***Significance test Z*** | ***p*** |
| --- | --- | --- | --- | --- | --- | --- | --- | --- | --- |
| *amisulpiride* |  |  |  |  |  |  |  |  |  |
| ≤6 wk (st=1; n=22) | 1 | -1.28 - 1.48 | 0 | 0 |  |  | 0.000 | 0.14 | 0.0887 |
| 6-16 wk (st=5; n=783) | 0.59 | 0.39 - 0.80 | 6.9 | 4 | 0.041 | 42.1% | 0.022 | 5.66 | <0.001 |
| 16-38 wk (st=4; n=520) | -0.76 | -2.74 - 1.22 | 84.38 | 3 | <0.001 | 96.4% | 3.751 | 0.75 | 0.452 |
| *aripiprazole* |  |  |  |  |  |  |  |  |  |
| ≤6 wk (st=11; n=1199) | 0.47 | 0.6 - 0.87 | 271.85 | 10 | <0.001 | 96.3% | 0.337 | 2.25 | 0.024 |
| 6-16 wk (st=11; n=872) | 0.32 | -0.81 - 1.45 | 188.35 | 9 | <0.001 | 95.2 | 2.593 | 0.1 | 0.92 |
| 16-38 wk (st=10; n=1079) | -0.87 | -1.51 – -0.23 | 291.71 | 9 | <0.001 | 96.9% | 0.828 | 2.67 | 0.008 |
| >38 wk (st=8; n= 1724) | 0.46 | 0.05 - 0.87 | 98.66 | 7 | <0.001 | 92.9% | 0.208 | 2.19 | 0.028 |
| *asenapine* |  |  |  |  |  |  |  |  |  |
| ≤6 wk (st=2; n=379) | 1.25 | 0.57 - 1.94 | 5.33 | 1 | 0.021 | 81.2% | 0.199 | 3.58 | <0.001 |
| 16-38 wk (st=1; n=194) | 0 | -0.48- 0.48 | 0 | 0 |  |  | 0.000 | 0 | 1 |
| >38 wk (st=2; n=987) | 2.09 | -0.45 - 4.63 | 11.5 | 1 | 0.001 | 91.3 | 3.086 | 1.62 | 0.106 |
| *chlorpromazine* |  |  |  |  |  |  |  |  |  |
| ≤6 wk (st=1; n=37) | 2.81 | 1.96 - 3.66 | 0 | 0 |  |  | 0.000 | 6.45 | <0.001 |
| 16-38 wk (st=1; n=19) | 4.90 | 2.04 - 7.76 | 0 | 0 |  |  | 0.000 | 3.36 | 0.001 |
| >38 wk (st=1; n=169) | 1.91 | 1.20 - 2.62 | 0 | 0 |  |  | 0.000 | 5.31 | <0.001 |
| *clozapine* |  |  |  |  |  |  |  |  |  |
| ≤6 wk (st=5; n=315) | 4.27 | 2.01 - 6.53 | 78.21 | 4 | <0.001 | 94.9% | 0.000 | 3.71 | <0.001 |
| 6-16 wk (st=7; n=671) | 3.36 | 1.82 - 4.90 | 55.94 | 6 | <0.001 | 89.3% | 0.000 | 4.28 | <0.001 |
| 16 -38 wk (st=10; n=684) | 3.17 | 0.22 - 6.13 | 700.04 | 9 | <0.001 | 98.7% | 0.035 | 2.1 | 0.035 |
| >38 wk (st=7; n=553) | 7.34 | 4.48 - 10.19 | 437.23 | 6 | <0.001 | 98.6% | 0.000 | 5.04 | <0.001 |
| *FGA* |  |  |  |  |  |  |  |  |  |
| ≤6 wk (st=2; n=119) | 1.38 | 0.91 - 1.84 | 0.4 | 1 | 0.527 | 0% | 0.000 | 5.78 | <0.001 |
| 6-16 wk (st=2; n=87) | -0.49 | -1.67 - 0.70 | 1.11 | 1 | 0.291 | 10.3% | 0.127 | 0.81 | 0.419 |
| 16-38 wk (st=2; n=87) | 2.46 | 1.9 - 3.02 | 0.45 | 1 | 0.501 | 0% | 0.000 | 8.65 | <0.001 |
| >38 wk (st=5; n=359) | 4.86 | 3.62 - 6.10 | 14.26 | 4 | 0.007 | 71.9% | 1.286 | 7.68 | <0.001 |
| *haloperidol* |  |  |  |  |  |  |  |  |  |
| ≤6 wk (st=10; n=2135) | 0.71 | 0.17 – 1.26 | 129.95 | 9 | <0.001 | 93.1% | 0.697 | 2.59 | 0.010 |
| 6-16wk (st=14; n=930) | 0.82 | 0.12 - 1.52 | 182.91 | 14 | <0.001 | 92.3% | 1.489 | 2.28 | 0.023 |
| 16-38 wk (st=4; n=363) | 2.32 | -1.01 - 5.65 | 94.18 | 3 | <0.001 | 96.8% | 10.680 | 1.36 | 0.172 |
| >38 wk (st=11; n=1104) | 2.93 | 1.40 – 4.47 | 123.99 | 10 | <0.001 | 91.9% | 5.414 | 3.73 | <0.000 |
| Lurasidone |  |  |  |  |  |  |  |  |  |
| ≤6 wk (st=1, n=150) | 0,27 | -0.01 - 0.55 | 0 | 0 |  | 0% | 0.000 |  |  |
| *melperone* |  |  |  |  |  |  |  |  |  |
| ≤6 wk (st=1; n=34) | 3.30 | -4.44 - 11.04 | 0 | 0 |  | 0% | 0.000 | 0.84 | 0.403 |
| 8-16 wk (st=1; n=34) | 3.20 | -1.41 - 7.81 |  |  |  |  | 0.000 | 1.36 | 0.173 |
| *olanzapine* |  |  |  |  |  |  |  |  |  |
| ≤6 wk (st=48; n=5931) | 2.55 | 2.20 - 2.88 | 956.80 | 49 | <0.001 | 94.9% | 1.305 | 14.37 | <0.001 |
| 6-16 wk (st=61; n=8601) | 3.12 | 2.67 - 3.58 | 3577.6 | 60 | <0.001 | 98.3% | 2.793 | 13.52 | <0.001 |
| 16-38 wk (st=42; n=8771) | 2.90 | 2.17 - 3.62 | 20902.88 | 41 | <0.001 | 99.8% | 5.284 | 7.82 | <0.001 |
| >38 wk (st=27; n=2974) | 4.63 | 3.27 - 5.98 | 2375.63 | 26 | <0.001 | 98.9% | 11.799 | 6.7 | <0.001 |
| *paliperidone* |  |  |  |  |  |  |  |  |  |
| ≤6 wk (st=15; n=2251) | 0.96 | 0.69 - 1.22 | 59.3 | 14 | <0.001 | 76.4% | 0.199 | 7.13 | <0.001 |
| 6-16 wk (st=2; n=848) | 1.08 | 0.84 - 1.31 | 0.12 | 1 | 0.726 | 0 | 0.000 | 8.99 | <0.001 |
| >38 wk (st=2; n=895) | 0.99 | 0.67 - 1.31 | 0.37 | 1 | 0.54 | 0% | 0.000 | 6.09 | <0.001 |
| *perphenazine* |  |  |  |  |  |  |  |  |  |
| 16-38 wk (st=1; n=257) | -0.91 | -0.97 - -0.85 | 0 | 0 |  |  | 0.000 | 29.18 | <0.001 |
| >38 wk (st=2; n=17) | 1.60 | 0.10 - 3.10 | 0.53 | 1 | 0.465 | 0% | 0.000 | 2.09 | 0.037 |
| *quetiapine* |  |  |  |  |  |  |  |  |  |
| ≤6 wk (st=8; n=301) | 1.54 | 0.54 - 2.54 | 65.91 | 7 | <0.001 | 89.4% | 1.636 | 3.02 | 0.003 |
| 6-16 wk (st=21; n=1871) | 1.62 | 1.25 - 2 | 165.08 | 20 | <0.001 | 87.9% | 0.450 | 8.55 | <0.001 |
| 16-38 wk (st=14; n=1805) | 1.05 | 0.53 - 1.56 | 987.2 | 13 | <0.001 | 98.7% | 0.551 | 3.98 | <0.001 |
| >38 wk (st=6; n=923) | 1.55 | 0.48 - 2.62 | 45.87 | 5 | <0.001 | 91.8% | 1.299 | 2.84 | 0.005 |
| *risperidone* |  |  |  |  |  |  |  |  |  |
| ≤6 wk (st=18; n=1069) | 1.84 | 1.44 - 2.23 | 124.86 | 17 | <0.001 | 86.4% | 0.553 | 9.06 | 0.001 |
| 6-16 wk (st=29; n=4917) | 1.64 | 1.29 - 1.99 | 696.02 | 28 | <0.001 | 96% | 0.727 | 9.26 | <0.001 |
| 16-38 wk (st=25; n=10771) | 1.95 | 1.56 - 2.35 | 2517.56 | 24 | <0.001 | 99% | 0.730 | 9.63 | <0.001 |
| >38 wk (st=26; n=4382) | 2.58 | 1.98 - 3.17 | 305.39 | 25 | <0.001 | 91.8% | 1.911 | 8.52 | <0.001 |
| *SGA* |  |  |  |  |  |  |  |  |  |
| ≤6 wk (st=1; n= 13) | 2.40 | 1.97 - 2.84 | 0 | 0 |  |  | 0.000 | 10.82 | <0.001 |
| 6-16 wk (st=1; n=13) | 6.10 | 5.61 - 6.59 |  |  |  |  | 0.000 | 24.44 | <0.001 |
| >38 (st=1; n=108) | 11.50 | 9.37 - 13.63 | 0 | 0 |  |  | 0.000 | 10.58 | <0.001 |
| *sertindole* |  |  |  |  |  |  |  |  |  |
| 6-16 wk (st=2; n=302) | 3.02 | 1.25 - 4.78 | 12.46 | 1 | <0.001 | 92% | 1.490 | 3.35 | 0.001 |
| >38 wk (st=1; n=8) | 0.60 | -9.59 - 10.79 | 0 | 0 |  |  | 0.000 | 0.12 | 0.908 |
| *ziprasidone* |  |  |  |  |  |  |  |  |  |
| ≤6 wk (st=4; n= 292) | 0.68 | 0.36 - 1.00 | 3.76 | 3 | 0.153 | 51.7% | 0.102 | 4.33 | <0.001 |
| 6-16 wk (st=8; n=687) | -0.23 | -1.21 - 0.76 | 57.28 | 7 | <0.001 | 87.8% | 1.552 | 0.45 | 0.653 |
| 16-38 wk (st=7; n=712) | -0.8 | -1.36 - -0.24 | 614.21 | 6 | <0.001 | 99% | 0.467 | 2.79 | 0.005 |
| >38 wk (st=1; n=32) | 0.25 | -0.03 - 0.53 | 0 | 0 |  |  | 0.000 | 1.77 | 0.077 |
| *placebo* |  |  |  |  |  |  |  |  |  |
| ≤6 wk (st=27; n=2424) | -0.12 | -0.36 - 0.13 | 586.43 | 26 | <0.001 | 95.6% | 0.326 | 0.95 | 0.341 |
| 6-16 wk (st=19: n=1665) | 0.11 | -0.02 - 0.24 | 361.47 | 21 | <0.001 | 94.2% | 0.037 | 1.66 | 0.097 |
| 16-38 wk (st=5: n=561) | -0.38 | -0.91 - 0.16 | 249.23 | 6 | <0.001 | 97.6% | 0475 | 1.38 | 0.169 |
| >38 wk (st=6: n=370) | -0.97 | -1.97 - 0.05 | 18.93 | 5 | 0.002 | 73.6% | 0.958 | 1.86 | 0.63 |

Total weight change of all studies identifeid per antipschotic per exposure category. Between brackets st=... indicates the number of studies included and n=... is number of particpants in the studie If one of the periods is not presented for a specific antipsychotic no data were available (e.g. amisulperide > 38wk). FGA: First Generation Antipsychotics, SGA: Second Generation Antipsychotics. Effect sizes are in kg. 95%CI = 95% Confidence Interval. I^2^: the variation in ES attributable to heterogeneity, Z: Significance test(s) of ES=0
