# Supplementary material for: Almost All Antipsychotics Result in Weight Gain: A Meta-Analysis
Source: PLoS One. 2014 Apr 24;9(4):e94112. doi: 10.1371/journal.pone.0094112 (PMC3998960; doi:10.1371/journal.pone.0094112)
Supplement: Table S3 — Weight changes in AP naives per exposure category. (DOCX) [file pone.0094112.s013.docx]

Table S3 Weight changes in drug naives per exposure category

| **Drug naive weight change** | **Effect size** | **95%CI** | **Heterogeneity** | **df** | **p** | **I^2^** | **Tau^2^** | **Significance test Z** | **p** |
| --- | --- | --- | --- | --- | --- | --- | --- | --- | --- |
| *aripiprazole* |  |  |  |  |  |  |  |  |  |
| ≤6 wk (st=1; n=154) | 0.82 | -0.12 – 1.76 | 0 | 0 |  |  | 0.000 | 1.71 | 0.042 |
| 6-16 wk (st=3;n=208) | 0.94 | 0.53 – 1.34 | 1.27 | 3 | 0.737 | 0 | 0.000 | 4.54 | <0.000 |
| >38wk (st=2; n=210) | 1.09 | 0.26 – 1.92 | 0.76 | 1 | 0.365 | 0 | 0.000 | 2.57 | 0.010 |
| *clozapine* |  |  |  |  |  |  |  |  |  |
| ≤6wk (st=1; n=14) | 6.52 | 4.73 – 8.31 | 0 | 0 |  |  | 0.000 | 7.15 | <0.001 |
| >38wk (st=1; n=177) | 2.99 | 2.11 – 3.87 | 0 | 0 |  |  | 0.000 | 6.64 | <0.001 |
| *FGA* |  |  |  |  |  |  |  |  |  |
| 16-38 wk (st=1; n=42) | 2 | 0.55 – 3.45 | 0 | 0 |  |  | 0.000 | 2.70 | 0.007 |
| >38 wk (st=3; n=89) | 5.20 | 3.56 – 6.82 | 0 | 0 |  |  | 0.000 | 6.31 | <0.001 |
| *haloperidol* |  |  |  |  |  |  |  |  |  |
| 6-16wk (st=2; n=110) | 3.75 | 2.83 – 4.66 | 0.02 | 1 | 0.291 | 10.3% | 0.000 | 8.03 | <0.001 |
| >38wk (st=3; n=89) | 7.42 | 2.65 – 12.20 | 17.62 | 2 | <0.001 | 88.6% | 15.129 | 3.05 | 0.002 |
| *olanzapine* |  |  |  |  |  |  |  |  |  |
| ≤6wk (st=11; n=365) | 3.42 | 2.27 – 4.58 | 297.91 | 10 | <0.001 | 96.6% | 3.417 | 5.79 | <0.001 |
| 6-16wk (st=8; n=256) | 5.43 | 3.46 – 7.39 | 161.53 | 7 | <0.001 | 95.7% | 7.377 | 5.41 | <0.001 |
| 16-38 (st=6; n=827) | 1.92 | 0.73 – 3.11 | 1586.74 | 12 | <0.001 | 98.6% | 1.842 | 3.17 | 0.002 |
| >38wk (st=4: n=242) | 11.36 | 5.03 – 17.69 | 79.07 | 3 | <0.001 | 96.2% | 37.54 | 3.52 | <0.001 |
| *quetiapine* |  |  |  |  |  |  |  |  |  |
| ≤6wk (st=4; n=57) | 1.91 | 0.04 – 3.77 | 20.98 | 3 | <0.001 | 85.7% | 2.985 | 2.01 | 0.045 |
| 6-16wk (st=2; n=31 ) | 2.56 | -0.37 – 5.49 | 4.09 | 1 | 0.43 | 75.6% | 3.399 | 1.72 | 0.086 |
| 16-38wk (st=1; n=94) | 0.18 | 0.13 – 0.24 | 0 | 0 |  |  | 0.000 | 6.46 | <0.001 |
| >38wk (st=1; n=45) | 1.54 | 0.56 – 2.52 | 0 | 0 |  |  | 0.000 | 3.08 | 0.002 |
| *risperidone* |  |  |  |  |  |  |  |  |  |
| ≤6wk (st=3; n=62) | 2.68 | 0.82 – 4.54 | 15.7 | 2 | <0.001 | 87.3 | 2.200 | 2.83 | 0.005 |
| 6-16wk (st=4; n=110) | 3.20 | 1.31 – 5.10 | 31.78 | 3 | <0.001 | 90.6% | 3.023 | 3.31 | 0.001 |
| 16-38wk (st=3; n=288) | 2.05 | 0.23 – 3.86 | 40.37 | 2 | <0.001 | 95% | 2.037 | 2.21 | 0.027 |
| >38wk (st=3; n=300) | 6.26 | 0.88 – 11.63 | 45.11 | 2 | <0.001 | 95.6% | 21.290 | 2.28 | 0.022 |
| *placebo* |  |  |  |  |  |  |  |  |  |
| ≤6wk (st=6; n=293) | 0 | -0.61 – 0.62 | 33.92 | 5 | <0.001 | 85.3% | 0.459 | 0.01 | 0.994 |
| 6-16wk (st=5; n=199) | 0.14 | -0.18 – 0.45 | 2.3 | 4 | 0.68 | 0% | 0.000 | 0.85 | 0.398 |
| 16-38wk (st=2; n=151) | -0.41 | -0.43 – -0.39 | 0.02 | 1 | 0.878 | 0% | 0.000 | 34.91 | <0.001 |
| >38wk (st=4; n=120) | -0.12 | -2.42 – 2.18 | 5.53 | 2 | 0.063 | 63.9% | 2.399 | 0.1 | 0.917 |
