# Supplementary material for: Almost All Antipsychotics Result in Weight Gain: A Meta-Analysis
Source: PLoS One. 2014 Apr 24;9(4):e94112. doi: 10.1371/journal.pone.0094112 (PMC3998960; doi:10.1371/journal.pone.0094112)
Supplement: Table S4 — Change of BMI per exposure category. (DOCX) [file pone.0094112.s014.docx]

Table S4 Change of BMI per exposure category

| **BMI change per period** | **Effect size** | **95%CI** | **Heterogeneity** | **df** | **p** | **I^2^** | **Tau^2^** | **Significance test Z** | **p** |
| --- | --- | --- | --- | --- | --- | --- | --- | --- | --- |
| *amisulpride* |  |  |  |  |  |  |  |  |  |
| 6-16wk (st=2; n=265) | 0.83 | -0.25 – 1.91 | 7.46 | 1 | 0.006 | 86.6% | 0.524 | 1.51 | 0.131 |
| 16-38wk (st=2; n=298) | -0.75 | -3.20 – 1.70 | 40.53 | 1 | <0.001 | 97.5% | 3.048 | 0.6 | 0.55 |
| *aripiprazole* |  |  |  |  |  |  |  |  |  |
| 6-16wk (st=1;n=22) | -1.1 | -1.77 – -0.42 | 0 | 0 |  |  | 0.000 | 3.19 | 0.001 |
| 16-38wk (st=3; n=218) | -0.78 | -1.46 – -0.10 | 10.78 | 2 | 0.005 | 81.4% | 0.285 | 2.26 | 0.024 |
| >38wk (st=1; n=226) | 0.4 | 0.13 – 0.67 | 0 | 0 |  |  | 0.000 | 2.95 | 0.003 |
| *clozapine* |  |  |  |  |  |  |  |  |  |
| ≤6wk (st=7; n=337) | 1.54 | 0.76 – 2.33 | 131.78 | 6 | <0.001 | 95.4% | 1.007 | 3.85 | <0.001 |
| 6-16wk (st=8; n=699) | 1.13 | 0.68 – 1.57 | 117.77 | 7 | <0.001 | 94.1% | 0.356 | 4.93 | <0.001 |
| 16-38 wk (st=3; n=308) | 1.54 | -0.6 – 3.67 | 513.42 | 2 | <0.001 | 99.6% | 3.532 | 1.41 | 0.158 |
| >38wk (st=4; n=279) | 3.21 | 1.54 – 4.88 | 103.35 | 3 | <0.001 | 97.1% | 2.512 | 3.77 | <0.001 |
| *FGA* |  |  |  |  |  |  |  |  |  |
| ≤6wk (st=2; n=119) | 0.42 | 0.25 – 0.58 | 0.89 | 1 | 0.346 | 0% | 0.000 | 5 | <0.001 |
| 6-16wk (st=4; n=893) | 0.36 | -0.62 – 1.33 | 3.82 | 1 | 0.051 | 73.8% | 0.369 | 0.72 | <0.001 |
| 16-38wk (st=4; n=857) | 0.7 | 0.46 – 0.94 | 0 | 1 | 1 | 0% | 0.000 | 5.67 | <0.001 |
| >38wk (st=6; n=484) | 1.71 | 1.44 – 1.98 | 10.87 | 5 | 0.054 | 54% | 0.059 | 12.22 | <0.001 |
| *haloperidol* |  |  |  |  |  |  |  |  |  |
| ≤6wk (st=1; n=659) | -0.8 | -0.20 – -0.68 | 0 | 0 |  |  | 0.000 | 12.6 | <0.001 |
| 6-16wk (st=5; n=184) | 0.36 | -0.62 – 1.33 | 37.06 | 4 | <0.001 | 89.2% | 0.478 | 1.3 | 0.195 |
| >38wk (st=4; n=97) | 2.63 | 1.42 – 3.85 | 19.53 | 3 | <0.001 | 84.6% | 1.234 | 4.25 | <0.001 |
| *melperone* |  |  |  |  |  |  |  |  |  |
| ≤6wk (st=1; n=34) | 0.8 | -0.68 – 2.28 | 0 | 0 |  |  | 0.000 | 1.06 | 0.288 |
| 6-16wk (st=1; n=34) | 0.8 | -0.50 – 2.10 | 0 | 0 |  |  | 0.000 | 1.21 | 0.226 |
| *olanzapine* |  |  |  |  |  |  |  |  |  |
| ≤6wk (st=16; n=1965) | 1.17 | 0.88 – 1.47 | 486.55 | 15 | <0.001 | 96.9% | 0.326 | 7.76 | <0.001 |
| 6-16wk (st=27; n=5425) | 1.17 | 0.83 – 1.51 | 7556.54 | 26 | <0.001 | 99.7% | 0.752 | 6.78 | <0.001 |
| 16-38wk (st=15; n=5914 | 1.42 | 1.08 – 1.76 | 2127.26 | 14 | <0.001 | 99.3% | 0.399 | 8.17 | <0.001 |
| >38wk (st=8; n=370) | 3.29 | 1.19 – 5.39 | 3840.35 | 15 | <0.001 | 99.8% | 8.648 | 3.07 | 0.002 |
| *paliperidone* |  |  |  |  |  |  |  |  |  |
| ≤6wk (st=6; n=733) | 0.28 | 0.15 – 0.42 | 20.99 | 5 | <0.001 | 76.2% | 0.023 | 3.99 | <0.001 |
| >38wk (st=1; n=507) | 0.4 | 0.23 – 0.57 | 0 | 0 |  |  | 0.000 | 4.6 | <0.001 |
| *Quetiapine* |  |  |  |  |  |  |  |  |  |
| ≤6wk (st=4; n=60) | 0.62 | 0.32 – 0.92 | 2.49 | 3 | 0.476 | 0% | 0.000 | 4.11 | <0.001 |
| 6-16wk (st=8; n=935) | 0.85 | 0.18 – 1.53 | 788.63 | 7 | <0.001 | 99.1% | 0.899 | 2.47 | 0.013 |
| 16-38wk (st=4; n=929) | 0.53 | -0.31 – 1.38 | 1312.52 | 3 | <0.001 | 99.8% | 0.736 | 1.24 | 0.216 |
| >38wk (st=4; n=734) | 0.6 | -0.53 – 1.78 | 248.12 | 3 | <0.001 | 98.8% | 1.268 | 1.04 | 0.297 |
| *risperidone* |  |  |  |  |  |  |  |  |  |
| ≤6wk (st=3; n=54) | 0.39 | 0.25 – 0.51 | 1.73 | 2 | 0.421 | 0% | 0.000 | 5.41 | <0.001 |
| 6-16wk (st=14; n=2489) | 0.66 | 0.29 – 1.03 | 2407.75 | 13 | <0.001 | 99.5% | 0.474 | 3.5 | <0.001 |
| 16-38wk (st=13; n=7778) | 0.6 | 0.44 – 0.76 | 945.39 | 12 | <0.001 | 98.7% | 0.070 | 7.31 | <0.001 |
| >38wk (st=16; n=3014) | 1.42 | 0.92 – 1.91 | 815.51 | 15 | <0.001 | 98.2% | 0.896 | 5.62 | <0.001 |
| *SGA* |  |  |  |  |  |  |  |  |  |
| ≤6wk (st=1; n=13) | 0.3 | -0.17 – 0.77 | 0 | 0 |  |  | 0.000 | 1.26 | 0.209 |
| 6-16wk (st=1; n=13) | 1.4 | 0.98 – 1.82 | 0 | 0 |  |  | 0.000 | 6.49 | <0.001 |
| 16-32 (st=1; n=16) | 3.7 | 2.98 – 4.42 | 0 | 0 |  |  | 0.000 | 10.02 | <0.001 |
| > 38 wk (st=1; n=108) | 3.8 | 3.44 – 4.16 | 0 | 0 |  |  | 0.000 | 20.62 | <0.001 |
| *ziprasidone* |  |  |  |  |  |  |  |  |  |
| ≤6wk (st=3; n= 167) | -0.28 | -0.92 – 0.36 | 19.46 | 2 | <0.001 | 89.7% | 0.331 | 0.59 | 0.557 |
| 6-16wk (st=2; n=243) | -0.76 | -1.54 – 0.21 | 7.8 | 1 | 0.005 | 87.2% | 0.279 | 1.91 | 0.056 |
| 16-32wk (st=1; n=55) | -0.5 | -0.69 – -0.32 | 0 | 0 |  |  | 0.000 | 5.30 | <0.001 |
| *placebo* |  |  |  |  |  |  |  |  |  |
| ≤6wk (st=3; n=257) | -0.3 | -0.43 – -0.17 | 0 | 2 | 1 | 0% | 0.000 | 4.48 | <0.001 |
| 6-16wk (st=1; n=10) | 0.2 | -0.31 – 0.71 | 0 | 0 |  |  | 0.000 | 0.77 | 0.438 |
| 16-38wk (st=1; n=209) | -0.44 | -1.08 – 0.21 | 3.06 | 1 | 0.8 | 67.3% | 0.176 | 1.32 | 0.188 |
| >38wk (st=1; n=30) | 0.41 | -0.14 – 0.95 | 0.01 | 1 | 0% | 1.47 | 0.000 | 1.47 | 0.141 |
