# Supplementary material for: Almost All Antipsychotics Result in Weight Gain: A Meta-Analysis
Source: PLoS One. 2014 Apr 24;9(4):e94112. doi: 10.1371/journal.pone.0094112 (PMC3998960; doi:10.1371/journal.pone.0094112)
Supplement: Table S5 — Change of BMI in AP naives per exposure category. (DOCX) [file pone.0094112.s015.docx]

Table S5. Change of BMI in AP naives per exposure category

| **BMI AP naive per period** | **Effect Size** | **95%CI** | **Heterogeneity** | **df** | **p** | **I^2^** | **Tau^2^** | **Significance test Z** | **p** |
| --- | --- | --- | --- | --- | --- | --- | --- | --- | --- |
| *Clozapine* |  |  |  |  |  |  |  |  |  |
| ≤6wk (st=1; n=14) | 3.80 | 3.13 – 4.47 | 0 | 0 |  |  | 0.000 | 11.16 | <0.001 |
| 6 - 16wk (st=1; n=30) | 1.49 | 1.42 – 1.56 | 0 | 0 |  |  | 0.000 | 40.81 | <0.001 |
| *FGA* |  |  |  |  |  |  |  |  |  |
| 16-38wk (st=1; n=42) | 0.70 | 0.19 – 1.21 | 0 | 0 |  |  | 0.000 | 2.67 | <0.001 |
| >38wk (st=3; n=122) | 1.60 | 1.29 – 1.92 | 0 | 0 |  |  | 0.000 | 9.96 | <0.001 |
| *haloperidol* |  |  |  |  |  |  |  |  |  |
| 6-16wk (st= 2, n=110) | 1.09 | 0.64 – 1.53 | 2.44 | 1 | 0.118 | 59% | 0.063 | 4.81 | <0.001 |
| >38 wk (st=3; 89) | 2.62 | 1.17 – 4.07 | 19.53 | 2 | <0.001 | 89.8% | 1.472 | 3.53 | <0.001 |
| *olanzapine* |  |  |  |  |  |  |  |  |  |
| ≤6wk (st=5; n=135) | 1.92 | 0.85 – 3 | 333.19 | 4 | <0.001 | 98.8% | 1.467 | 3.52 | <0.001 |
| 6-16wk (st=4; n=183) | 1.29 | 2.84 – 4.06 | 205.62 | 3 | <0.001 | 98.5% | 0.599 | 5.22 | <0.001 |
| 16-38wk (st=2; n=646) | 1.48 | 0.70 – -0.09 | 118.67 | 1 | <0.001 | 99.2% | 0.317 | 1.74 | 0.082 |
| >38wk (st=3; n=93) | 4.41 | 3.78 – 5.05 | 2.68 | 2 | 0.262 | 25.4% | 0.084 | 13.64 | <0.001 |
| *quetiapine* |  |  |  |  |  |  |  |  |  |
| ≤6 wk (st=3; n=45) | 0.57 | 0.26 – 0.89 | 1.65 | 2 | 0.437 | 0% | 0.000 | 3.55 | <0.001 |
| 6-16wk (st=2; n=31) | 1.27 | -0.2 – 2.74 | 14.53 | 1 | <0.001 | 93.1% | 1.048 | 1.7 | 0.09 |
| 16-38wk (st=1;n=94) | 0.20 | 0.18 – 0.22 | 0 | 0 |  |  | 0.000 | 19.39 | <0.001 |
| *risperidone* |  |  |  |  |  |  |  |  |  |
| ≤6wk (st=1;n=30) | 0.48 | 0.28 – 0.68 | 0 | 0 |  |  | 0.000 | 4.8 | <0.001 |
| 6-16wk (st=3; n=106) | 0.96 | 0.19 – 1.73 | 106.38 | 2 | <0.001 | 98.1% | 0.451 | 2.43 | 0.015 |
| 16-38wk (st=2; n=277) | 0.48 | 0.09 –0.87 | 11.9 | 1 | 0.001 | 91.6% | 0.073 | 2.43 | 0.015 |
| >38wk (st=3; n=151) | 3.49 | 2.27 – 4.71 | 10.33 | 2 | 0.006 | 80.6% | 0.932 | 5.62 | <0.001 |
| *SGA* |  |  |  |  |  |  |  |  |  |
| 16-38wk (st=1;n=16 | 3.70 | 2.98 – 4.42 | 0 | 0 |  |  | 0.000 | 10.02 | <0.001 |
| >38wk (st=1; n-108 | 3.80 | 3.44 – 4.16 | 0 | 0 |  |  | 0.000 | 20.62 | <0.001 |
| Sulpiride |  |  |  |  |  |  |  |  |  |
| 6-16wk (st=1; n=29) | 0.66 | 0.62 – 0.70 | 0 | 0 |  |  | 0.000 | 29.62 | <0.001 |
| *Ziprasidone* |  |  |  |  |  |  |  |  |  |
| ≤6wk (st=1; n=14) | -0.7 | -1.14 – -0.27 | 0 | 0 |  |  | 0.000 | 3.16 | 0.002 |
| *Placebo* |  |  |  |  |  |  |  |  |  |
| ≤6wk (st=1; n=11) | -0.3 | -1 – 0.40 | 0 | 0 |  |  | 0.000 | 0.84 | 0.4 |
| 6-16wk (st=1;n=10) | 0.2 | -0.31 – 0.71 | 0 | 0 |  |  | 0.000 | 0.77 | 0.44 |
| 16-38wk (st=1; n=142) | -0.2 | -0.22 – -0.18 | 0 | 0 |  |  | 0.000 | 23.83 | <0.001 |
| >38wk (st=2; n=37) | 0,95 | 0.41 – -0.14 | 0 | 0 |  |  | 0.000 | 1.47 | 0.141 |
