# Supplementary material for: Almost All Antipsychotics Result in Weight Gain: A Meta-Analysis
Source: PLoS One. 2014 Apr 24;9(4):e94112. doi: 10.1371/journal.pone.0094112 (PMC3998960; doi:10.1371/journal.pone.0094112)
Supplement: Table S6 — Proportion (7%) of weight gain per exposure category. (DOCX) [file pone.0094112.s016.docx]

Table S6. Proportion (7%) of weight gain per exposure category

| **7% weight gain** | **%** | **95%CI** | **Heterogeneity** | **df** | **p** | **I^2^** | **Tau^2^** | **Significance test Z** | **p** |
| --- | --- | --- | --- | --- | --- | --- | --- | --- | --- |
| *Amisulpride* |  |  |  |  |  |  |  |  |  |
| 6-16 wk (st=5; n=378) | 4 | 1 – 18 | 2.96 | 4 | 0.565 | 0% | 0.000 | 12.15 | <0.001 |
| 16-38 wk (st=1; n=152) | 18 | 13 - 25 | 0 | 0 |  |  | 0.000 | 7.18 | <0.001 |
| >38 wk (st=1; n=256) | 16 | 12 - 21 | 0 | 0 |  |  | 0.000 | 9.80 | <0.001 |
| *Aripiprazole* |  |  |  |  |  |  |  |  |  |
| ≤6 wk (st=11; n=1199) | 5 | 3 - 8 | 31.15 | 10 | 0.001 | 67.9% | 0.445 | 11.49 | <0.001 |
| 6-16 wk (n=12; 1802) | 7 | 5 - 10 | 42.49 | 11 | <0.001 | 74.1% | 0.307 | 13.29 | <0.001 |
| 16-38 wk (st=7; n=1137) | 12 | 8 - 17 | 32.12 | 6 | <0.001 | 81.3% | 0.276 | 8.75 | <0.001 |
| >38 wk (st=6; n=619) | 21 | 12 - 33 | 42.91 | 5 | <0.001 | 88.3% | 0.476 | 4.30 | <0.001 |
| *Asenapine* |  |  |  |  |  |  |  |  |  |
| ≤6 wk (st=3;n=438) | 6 | 4 - 9 | 0.38 | 2 | 0.827 | 0% | 0.000 | 13.63 | <0.001 |
| 16-38 wk (st=1; n=194) | 4 | 2 - 7 | 0 | 0 |  |  | 0.000 | 8.57 | <0.001 |
| >38 wk (st=2; n=987) | 23 | 6 - 57 | 37.98 | 1 | <0.001 | 97.4% | 1.175 | 1.58 | 0.113 |
| Chlorpromazine |  |  |  |  |  |  |  |  |  |
| ≤6 wk (st=2; n=191) | 18 | 10 - 29 | 2.3 | 1 | 0.130 | 56.4% | 0.130 | 4.64 | <0.001 |
| >38 wk (st=1; n=169) | 3 | 24 - 37 | 0 | 0 |  |  | 0.000 | 5.05 | <0.001 |
| Clozapine |  |  |  |  |  |  |  |  |  |
| ≤6 wk (st=1; n=40) | 28 | 16 - 44 | 0 | 0 |  |  | 0.000 | 2.68 | 0.007 |
| 6-16 wk (st=1; n=57) | 52 | 39 - 65 | 0 | 0 |  |  | 0.000 | 0.30 | 0.763 |
| 16-38 wk (st=3; n= 126) | 43 | 14 - 79 | 28.62 | 2 | <0.001 | 93% | 1.787 | 0.34 | 0.735 |
| >38 wk (st=3; n=491) | 48 | 25 - 72 | 43.93 | 2 | <0.001 | 95.4% | 0.761 | 0.17 | 0.863 |
| *FGA* |  |  |  |  |  |  |  |  |  |
| ≤6 wk (st=1; n=45) | 7 | 2 - 19 | 0 | 0 |  |  | 0.000 | 4.42 | <0.001 |
| 16-38 wk (st=1; n=45) | 23 | 13 - 38 | 0 | 0 |  |  | 0.000 | 3.38 | 0.001 |
| >38 wk (st=6; n=1138) | 25 | 16 - 36 | 56.66 | 5 | <0.001 | 91.2% | 0.391 | 4.01 | <0.001 |
| *Haloperidol* |  |  |  |  |  |  |  |  |  |
| ≤6 wk (st=3; n=884) | 4 | 2 - 11 | 11.18 | 2 | 0.004 | 82.1% | 0.543 | 6.40 | <0.001 |
| 6-16 wk (st=5; n=397) | 9 | 5 - 15 | 9.14 | 4 | 0.058 | 56.3% | 0.222 | 8.13 | <0.001 |
| >38 wk (st=5; n=341) | 4 | 2 - 64 | 53.31 | 4 | <0.001 | 92.5% | 1.170 | 0.80 | 0.421 |
| *Lurasidone* |  |  |  |  |  |  |  |  |  |
| ≤6 wk (st=1; n=90) | 7 |  | 0 | 0 |  |  | 0.000 |  |  |
| *Olanzapine* |  |  |  |  |  |  |  |  |  |
| ≤6 wk (st=11; n=2264) | 25 | 17 - 34 | 100.6 | 10 | <0.001 | 90% | 0.470 | 4.89 | <0.001 |
| 6-16 wk (st=30; 3802) | 27 | 23 - 32 | 231.74 | 29 | <0.001 | 87.5% | 0.313 | 8.77 | <0.001 |
| 16-38 wk (st=18; n=3629) | 2 | 15 - 27 | 298.75 | 17 | <0.001 | 94.3% | 0.504 | 7.74 | <0.001 |
| >38 wk (st=22; n=8497) | 41 | 36 - 47 | 367.76 | 21 | <0.001 | 94.3% | 0.248 | 2.98 | 0.003 |
| *Paliperidone* |  |  |  |  |  |  |  |  |  |
| ≤6 wk (st=8;n=957) | 9 | 6 - 13 | 28.05 | 7 | <0.001 | 75% | 0.318 | 9.96 | <0.001 |
| 16-38 wk (st=1; n=104) | 2 | 13 - 29 | 0 | 0 |  |  | 0.000 | 5.65 | <0.001 |
| >38 wk (st=2; n=895) | 14 | 12 - 17 | 0.72 | 1 | 0.395 | 0% | 0.000 | 18.77 | <0.001 |
| *Perphenazine* |  |  |  |  |  |  |  |  |  |
| 16-38 wk (st=1; n=257) | 12 | 9 - 17 | 0 | 0 |  |  | 0.000 | 10.38 | <0.001 |
| *>*38 wk (st=2; n=17) | 24 | 4 - 74 | 2.60 | 1 | 0.107 | 61.6% | 1.487 | 1.03 | 0.303 |
| *Quetiapine* |  |  |  |  |  |  |  |  |  |
| ≤6 wk (st=5; n=1010) | 3 | 1 - 4 | 6.81 | 4 | 0.146 | 41.2% | 0.162 | 12.78 | <0.001 |
| 6-16 wk (st=16; n=1476) | 13 | 9 - 18 | 67.78 | 15 | <0.001 | 77.8% | 0.380 | 10.33 | <0.001 |
| 16-38 wk (st=9; n=937) | 16 | 12 - 23 | 32.34 | 8 | <0.001 | 75.3% | 0.264 | 7.78 | <0.001 |
| >38 wk (st=5; n=1147) | 19 | 1 - 35 | 70.17 | 4 | <0.001 | 94.3% | 0.773 | 3.47 | 0.001 |
| *risperidone* |  |  |  |  |  |  |  |  |  |
| ≤6 wk (st=5; n=241) | 15 | 9 - 25 | 9.83 | 4 | 0.043 | 59.3% | 0.281 | 5.42 | <0.001 |
| 6-16 wk (st=12; 1274) | 11 | 7 - 16 | 52.65 | 11 | <0.001 | 79.1% | 0.407 | 9.55 | <0.001 |
| 16-38 wk (st=10; n=1435) | 18 | 13 - 24 | 48.65 | 9 | <0.001 | 81.5% | 0.250 | 8.15 | <0.001 |
| >38 wk (st=22; n=5428) | 28 | 23 - 34 | 295.50 | 21 | <0.001 | 92.9% | 0.330 | 7.09 | <0.001 |
| *SGA* |  |  |  |  |  |  |  |  |  |
| 16- 38 wk (st=1; n=166) | 21 | 15 - 28 | 0 | 0 |  |  | 0.000 | 6.95 | <0.001 |
| >38 wk (st=1; n=108) | 72 | 63 - 8 | 0 | 0 |  |  | 0.000 | 4.41 | <0.001 |
| *ziprasidone* |  |  |  |  |  |  |  |  |  |
| ≤6 wk (st=2; n=277) | 2 | 0 - 4 | 1.93 | 1 | 0.165 | 48.1% | 3.777 | 2.29 | 0.022 |
| 6-16 wk (st=5; n=480) | 7 | 5 - 1 | 3.91 | 4 | 0.419 | 0% | 0.000 | 13.96 | <0.001 |
| 16-38 wk (st=4; n=474) | 8 | 3 - 21 | 25.34 | 3 | <0.001 | 88.2% | 1.036 | 4.37 | <0.001 |
| Placebo |  |  |  |  |  |  |  |  |  |
| ≤6 wk (st=12; n=1405) | 4 | 3 - 5 | 18.56 | 11 | 0.069 | 40.7% | 0.186 | 16.07 | <0.001 |
| 6-16 wk (st=15; n=1919) | 4 | 2 - 8 | 31.21 | 14 | 0.005 | 55.1% | 0.261 | 16.83 | <0.001 |
| 16-38 wk st=4; n=586) | 4 | 3 - 6 | 15.54 | 3 | 0.001 | 80.7% | 0.903 | 5.87 | <0.001 |
| >38 wk (st=3; n=299) | 4 | 1 - 10 | 4.45 | 2 | 0.108 | 55.1% | 0.433 | 6.13 | <0.001 |
