# Supplementary material for: Almost All Antipsychotics Result in Weight Gain: A Meta-Analysis
Source: PLoS One. 2014 Apr 24;9(4):e94112. doi: 10.1371/journal.pone.0094112 (PMC3998960; doi:10.1371/journal.pone.0094112)
Supplement: Table S7 — Proportion (7%) of weight increase in AP naives per exposure category. (DOCX) [file pone.0094112.s017.docx]

Table S7. Proportion (7%) of weight increase in AP naives per exposure category

| **7% weight increase AP naive per period** | **%** | **95%CI** | **Hetero-geneity** | **df** | **p** | **I^2^** | **Tau^2^** | **Significance**  **test Z** | **p** |
| --- | --- | --- | --- | --- | --- | --- | --- | --- | --- |
| *Aripiprazole* |  |  |  |  |  |  |  |  |  |
| ≤6wk (st=1; n=154) | 1 | 0 - 5 | 0 | 0 |  |  | 0.000 | 5.82 | <0.001 |
| 6-16 wk (st=1; n=154) | 15 | 1 -21 | 0 | 0 |  |  | 0.000 | 7.74 | <0.001 |
| >38 wk (st=3; 364) | 26 | 13 - 45 | 22.8 | 2 | <0.001 | 91.2% | 0.499 | 2.45 | 0.014 |
| *Haloperidol* |  |  |  |  |  |  |  |  |  |
| >38 wk (st=2; n=76) | 71 | 25 - 95 | 12.44 | 1 | <0.001 | 92% | 1.963 | 0.88 | 0.378 |
| *Olanzapine* |  |  |  |  |  |  |  |  |  |
| ≤6wk (st=3; n=50) | 55 | 24 - 83 | 9.37 | 2 | 0.009 | 78.7% | 1.167 | 0.31 | 0.756 |
| 16-38wk (st=1; n=100) | 11 | 06 - 19 | 0 | 0 |  |  | 0.000 | 6.54 | <0.001 |
| >38wk (st=1; n=214) | 7 | 49 - 85 | 9.66 | 2 | 0.008 | 79.3% | 0.441 | 1.84 | 0.066 |
| *Quetiapine* |  |  |  |  |  |  |  |  |  |
| 6-16wk (st=1; n=17) | 12 | 03 - 37 | 0 | 0 |  |  | 0.000 | 2.68 | 0.007 |
| 16-38wk (st=1; n=94) | 6 | 3 - 13 | 0 | 0 |  |  | 0.000 | 6.34 | <0.001 |
| >38wk (st=1; n=145) | 25 | 19 - 33 | 0 | 0 |  |  | 0.000 | 5.73 | <0.001 |
| *Risperidone* |  |  |  |  |  |  |  |  |  |
| ≤6wk (st=1; n=19) | 38 | 19 - 6 | 0 | 0 |  |  | 0.000 | 1.08 | 0.281 |
| 16-38wk (st=1; n=85) | 11 | 5 - 2 | 0 | 0 |  |  | 0.000 | 6.03 | <0.001 |
| >38wk (st=3; n=300) | 55 | 36 - 72 | 14.71 | 2 | 0.001 |  | 0.370 | 0.48 | 0.628 |
| *Placebo* |  |  |  |  |  |  |  |  |  |
| ≤6wk (st=1; n=165) | 1 | 0 - 5 | 0 | 0 |  |  |  | 6.03 | <0.001 |
| 16-38wk (st=1; n=142) | 3 | 1 - 8 | 0 | 0 |  |  |  | 7.07 | <0.001 |
| >38wk (st=2; n=37) | 28 | 15 - 46 | 0 | 0 |  |  |  | 2.79 | 0.005 |
