# Supplementary material for: Almost All Antipsychotics Result in Weight Gain: A Meta-Analysis
Source: PLoS One. 2014 Apr 24;9(4):e94112. doi: 10.1371/journal.pone.0094112 (PMC3998960; doi:10.1371/journal.pone.0094112)
Supplement: Table S8 — Proportion of 7% weight loss per exposure category. (DOCX) [file pone.0094112.s018.docx]

Table S8. Proportion of 7% weight loss per exposure category

| **7% weight reduction** | **%** | **95%CI** | **Heterogeneity** | **df** | **p** | **I^2^** | **Tau^2^** | **Significance test Z** | **p** |
| --- | --- | --- | --- | --- | --- | --- | --- | --- | --- |
| *Amisulpride* |  |  |  |  |  |  |  |  |  |
| 6-16wk (st=2; n=105) | 7 | 4 - 14 | 0.63 | 1 | 0.427 | 0% | 0.000 | 6.64 | <0.001 |
| *Aripiprazole* |  |  |  |  |  |  |  |  |  |
| ≤6 wk (st=1; n=932) | 8 | 7 - 1 | 0 | 0 |  |  | 0.000 | 20.23 | <0.001 |
| 6-16 wk (st=5; n=898) | 7 | 4 - 11 | 13.60 | 4 | 0.009 | 70.6% | 0.230 | 9.74 | <0.001 |
| 16-38wk 9st=1; n=284) | 16 | 12 - 21 | 0 | 0 |  |  | 0.000 | 10.28 | <0.001 |
| >38wk (st=1; n=78) | 2 | 0 - 9 | 0 | 0 |  |  | 0.000 | 4.81 | <0.001 |
| *Asenapine* |  |  |  |  |  |  |  |  |  |
| ≤ 6wk (st=1; n=194) | 2 | 1 - 5 | 0 | 0 |  |  | 0.000 | 7.41 | <0.001 |
| 16-38wk (st=1; n=194) | 3 | 1 - 7 | 0 | 0 |  |  | 0.000 | 8.36 | <0.001 |
| >38wk (st=1; n-908) | 2 | 1 - 3 | 0 | 0 |  |  | 0.000 | 16.42 | <0.001 |
| *Olanzapine* |  |  |  |  |  |  |  |  |  |
| ≤6wk (st=4; n=645) | 1 | 1 - 3 | 1.48 | 3 | 0.686 | 0% | 0.000 | 11.60 | <0.001 |
| 6-16wk (st=1; n=62) | 3 | 1 - 11 | 0 | 0 |  |  | 0.000 | 4.54 | <0.001 |
| 16-38wk (st=4; n=770) | 6 | 4 - 9 | 6 | 3 | 0.112 |  | 0.097 | 12.35 | <0.001 |
| >38wk (st=2; n=611) | 4 | 2 - 9 | 3.10 | 1 | 0.079 |  | 0.178 | 8.54 | <0.001 |
| *Paliperidone* |  |  |  |  |  |  |  |  |  |
| ≤6wk (st=6; n=733) | 2 | 1 - 4 | 0.47 | 5 | 0.993 | 0% | 0.000 | 14.96 | <0.001 |
| *Risperidone* |  |  |  |  |  |  |  |  |  |
| 6-16wk (st=2; n=192) | 3 | 1 - 7 | 0.48 | 1 | 0.488 | 0% | 0.000 | 8.05 | <0.001 |
| >38wk 9st=2; n=518) | 9 | 4 - 17 | 5.40 | 1 | 0.020 | 67.7% | 0.235 | 6.20 | <0.001 |
| *Ziprasidone* |  |  |  |  |  |  |  |  |  |
| 6-16 wk (st=5; n=364) | 1 | 5 - 17 | 10.89 | 4 | 0.028 | 63.3% | 0.322 | 6.96 | <0.001 |
| *Placebo* |  |  |  |  |  |  |  |  |  |
| ≤6 wk (st=4; n=766) | 5 | 2 - 11 | 15.27 | 3 | 0.002 | 80.3% | 0.533 | 7.07 | <0.001 |
| 6-16 wk (st=2; n=274) | 9 | 6 - 13 | 0.58 | 1 | 0.446 | 0% | 0.000 | 10.91 | <0.001 |
| 16-38 wk(st=1; n=192) | 10 | 6 - 13 | 0 | 0 |  |  | 0.000 | 9.15 | <0.001 |
| >38 wk (st=1; n=83) | 17 | 1 - 27 | 0 | 0 |  |  | 0.000 | 5.43 | <0.001 |
